# Synthesis of bridged tricyclo[5.2.1.0^1,5^]decanes via nickel-catalyzed asymmetric domino cyclization of enynones

**DOI:** 10.1038/s41467-020-15837-1

**Published:** 2020-04-20

**Authors:** Jiachang Chen, Yiming Wang, Zhengtian Ding, Wangqing Kong

**Affiliations:** The Center for Precision Synthesis (CPS), Institute for Advanced Studies (IAS), Wuhan University, 430072 Wuhan, People’s Republic of China

**Keywords:** Asymmetric catalysis, Homogeneous catalysis, Synthetic chemistry methodology

## Abstract

The restricted availability, expense and toxicity of precious metal catalysts such as rhodium and palladium challenge the sustainability of synthetic chemistry. As such, nickel catalysts have garnered increasing attention as replacements for enyne cyclization reactions. On the other hand, bridged tricyclo[5.2.1.0^1,5^]decanes are found as core structures in many biologically active natural products; however, the synthesis of such frameworks with high functionalities from readily available precursors remains a significant challenge. Herein, we report a nickel-catalyzed asymmetric domino cyclization reaction of enynones, providing rapid and modular synthesis of bridged tricyclo[5.2.1.0^1,5^]decane skeletons with three quaternary stereocenters in good yields and remarkable high levels of regio- and enantioselectivities (92–99% ee).

## Abstract (web summary)

Tricyclo[5.2.1.0^1,5^]decanes are often found in bioactive natural products; however, their synthesis poses significant challenges. Here, the authors report a nickel-catalyzed asymmetric domino cyclization of enynones, providing a rapid and modular synthesis of bridged tricyclo[5.2.1.0^1,5^]decane skeletons.

## Introduction

**Fig. 1:**
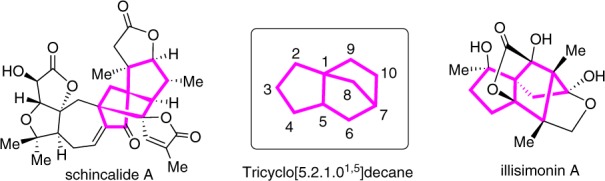
Representative examples of bioactive compounds. Bridged tricyclo[5.2.1.0^1,5^]decane scaffolds are structural cores in many natural products.

With the growing concerns about environmental sustainability, the development of elegant methodologies for efficient and concise synthesis of complex bioactive natural and pharmaceutical products in a step-, atom- and redox-economic manner has received widespread attention. One of the most effective approaches to achieve such a goal is to develop catalytic asymmetric domino reactions in which multiple bond-making events occur in one-pot, and complex chiral compounds with multi-stereogenic centers are generated from easily accessible precursors^1,2^. Consequently, significant efforts have been directed towards the development of asymmetric domino reactions, where the focus has been on the efficient synthesis of biologically important and highly functionalized chiral carbo- and heterocyclic compounds. In this context, the bridged tricyclo[5.2.1.0^1,5^]decanes are found as core structures in many bioactive natural products, including Schincalide A^3^ and Illisimonin A^4^ (Fig. 1). Despite their importance, the bridged tricyclo[5.2.1.0^1,5^]decane remain an elusive skeleton, and the development of which has been clearly underexploited^5–7^. Only recently, Rychnovsky’s group reported the first total synthesis of Illisimonin A, in which the key tricyclic core was constructed by Diels-Alder reaction, and enantioselective control of this transformation has not yet been achieved^8^. The limited output for these challenging molecules may be due to the difficulty in asymmetric synthesis of the bridged tricyclo[5.2.1.0^1,5^]decane core^9–12^, which hinders any further study on their potential bioactive properties. Therefore, a general approach that enables the modular and enantioselective synthesis of this key skeleton is highly desired and sought-after.

**Fig. 2:**
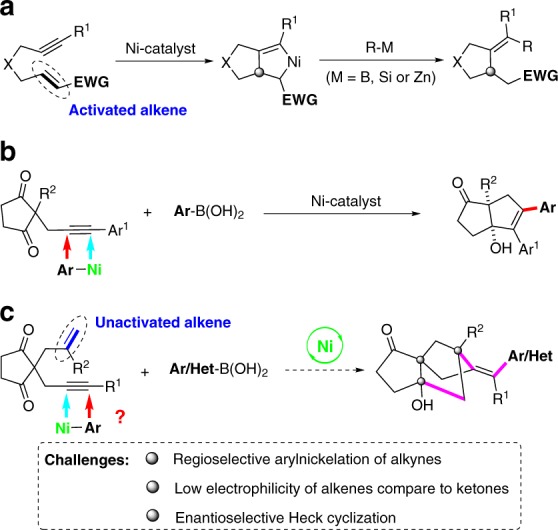
Reaction design. **a** Ni-catalyzed coupling cyclization of an alkyne and an activated alkene; **b** Ni-catalyzed asymmetric coupling cyclization of an alkyne and a ketone; **c** Working hypothesis for bridged tricyclo[5.2.1.0^1,5^]decanes synthesis via coupling cyclization of an alkyne, an alkene and a ketone.

Transition metal-catalyzed asymmetric domino cyclization of 1,n-enynes represents a powerful synthetic tool to rapidly assemble chiral carbo- and heterocyclic compounds. Traditionally used precious metal catalysts based on Rh, Ir, and Pd, etc., have been widely used in such transformations^13–25^. However, they are very expensive and their reserves are declining, thus limiting their wide-scale industrial applications. As such, increasing attention has been focused on the development and use of earth-abundant and sustainable element, especially nickel catalysts, to replace these highly expensive and scarce metals in 1,n-enyne cyclization. However, the reaction is typically restricted to the use of activated alkenes, and the regioselectivity is controlled by the formation of a five-membered ring nickelacyclic intermediate^26,27^ (Fig. 2a). Recently, Lam et al. developed Ni-catalyzed asymmetric coupling cyclization of aryl-substituted alkynes with ketones^28^ (Fig. 2b). We envisioned that the introduction of a 1,3-cyclopentanedione functionality on the 1,6-enyne moiety might facilitate a domino arylnickelation of alkyne/Heck cyclization with alkene/nucleophilic addition to ketone sequence, and therefore provides an expedient access to biologically important bridged tricyclo[5.2.1.0^1,5^] decanes (Fig. 2c).

However, to realize the above conceptually simple yet attractive reaction, many challenging problems need to be addressed. The first is to control the regioselectivity of the 1,2-addition of the arylnickel species to the alkyne moiety^29–33^. since the regioselectivity we expected is contrary to that reported by Lam^28^. The second is to control the enantioselectivity of the Heck-cyclization process. Another challenge is that ketones are more electrophilic than unactivated alkenes, the direct cyclization of alkynes and ketones may take place preferentially, while unactivated alkenes do not participate in the cyclization process^34–50^.

Herein, we report catalytic enantioselective construction of bridged tricyclo[5.2.1.0^1,5^]decanes with multiple quaternary stereocenters by Ni-catalyzed domino coupling cyclization of an alkyne, an alkene and a ketone in a highly regio- and enantioselective fashion. This domino strategy not only has the advantage of being efficient, simple and starting from easily accessible precursors, but also provides the desired targets with two additional functional sites (a ketone and a fully substituted double bond), which could be easily used for diversity-oriented synthesis. This work documents a practical, catalytic enantioselective (92–99% ee) approach to the most diverse set of bridged tricyclo[5.2.1.0^1,5^]decanes reported to-date.

## Results

### Reaction development

According to the reaction design shown in Fig. 2c, we began with an investigation of the model reaction for the construction of bridged tricyclo[5.2.1.0^1,5^]decanes using the 1,3-cyclopentanedione tethered 1,6-enyne 1a as substrate, which can be easily prepared by a two-step sequence consisting of the reductive Knoevenagel condensation of commercially available cyclopentane-1,3-dione with alkynals^51,52^ followed by allylation with allyl halides. The reaction was first conducted with 10 mol % of Ni(OAc)_2_.4H_2_O and 12 mol % of (*S*)-phenyl-Phox (**L1**) as catalyst in MeCN. As anticipated, the cyclization of **1a** to **4aa** (60%) was the major product of the reaction^28^, in which unactivated alkene was not involved, and only trace amount of the desired **3aa** could be observed (Table 1, entry 1). The formation of **4aa** could not be mitigated and remained the main reaction pathway by using MeOH, DMF or toluene as solvent (Table 1, entries 2–4), which reinforces the notion that the domino cyclization of **1a** to bridged tricyclo[5.2.1.0^1,5^]decane **3aa** would be far from trivial.

**Table 1:** Optimization of reaction conditions^a^.


Entry	Ligand	Solvent	Yield of 4aa (%)^b^	Yield of 3aa (%)^b^	ee of 3aa (%)^c^
1	**L1**	MeCN	60	Trace	–
2	**L1**	MeOH	54	Trace	–
3	**L1**	DMF	12	Trace	–
4	**L1**	Toluene	5	Trace	–
5	**L1**	TFE	12	33	71
6	**L2**	TFE	Trace	41	65
7	**L3**	TFE	12	42	76
8	**L4**	TFE	9	46	75
9	**L5**	TFE	24	26	60
10	**L6**	TFE	20	Trace	–
11	**L7**	TFE	Trace	12	25
12	**L8**	TFE	Trace	Trace	–
13	**L9**	TFE	37	Trace	–
14	**L10**	TFE	<1	77	98

*TFE* 2,2,2-Trifluoroethanol.

^a^Reaction condition: **1a** (0.1 mmol), **2a** (2 equiv), Ni(OAc)_2_.4H_2_O (0.1 equiv), ligand (0.12 equiv), TFE (1 mL) at 100 °C for 48 h.

^b^yields of isolated products.

^c^Determined by HPLC on a chiral stationary phase.

To suppress the formation of undesired **4aa**, it is necessary to activate the double bond. Interestingly, the desired product **3aa** was afforded in 33% yield and 71% ee when the reaction was performed in TFE as solvent (Table 1, entry 5). Subsequently, different chiral Phox-type ligands **L2**-**L4** were screened, the yields and ee values of **3aa** remained moderate (Table 1, entries 6–8). To further improve the yield and enantioselectivity, various bidentate phosphine ligands such as (*S*)-BINAP (**L5**), (*S*)-SegPhos (**L6**), (*S*,*S*)-Me-DuPhos (**L7**), (*S*,*S*)-Ph-BPE (**L8**) and (*R*,*S*)-Ming-Phos (**L9**) were investigated. Unfortunately, neither the yields nor the enantioselectivities were improved (Table 1, entries 9–13). Excitingly, **3aa** was obtained in 77% yield and 98% ee when a conformationally rigid P-stereogenic bis(phospholane) ligand **L10** (*1* *R*,*1’R*,*2* *S*,*2’S*-Duanphos)^53^ was used (Table 1, entry 14).

**Fig. 3:**
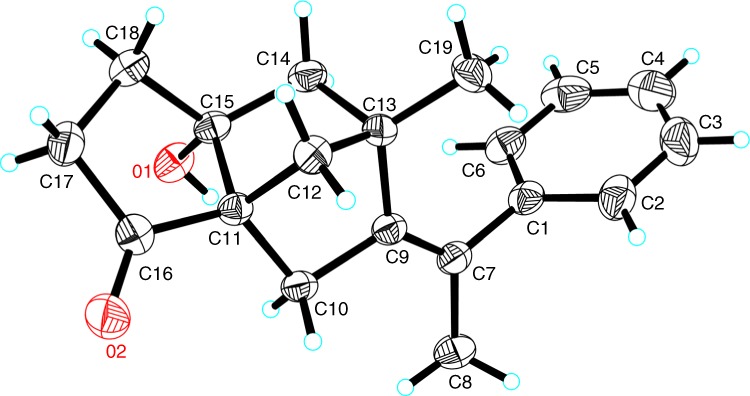
Absolute configuration. ORTEP representation of the product **3aa**.

The *E/Z* configuration of the double bond and the absolute configuration of three newly formed quaternary stereocenters in **3aa** were unambiguously assigned by X-ray crystal crystallography (Fig. 3).

### Substrate scope

**Fig. 4:**
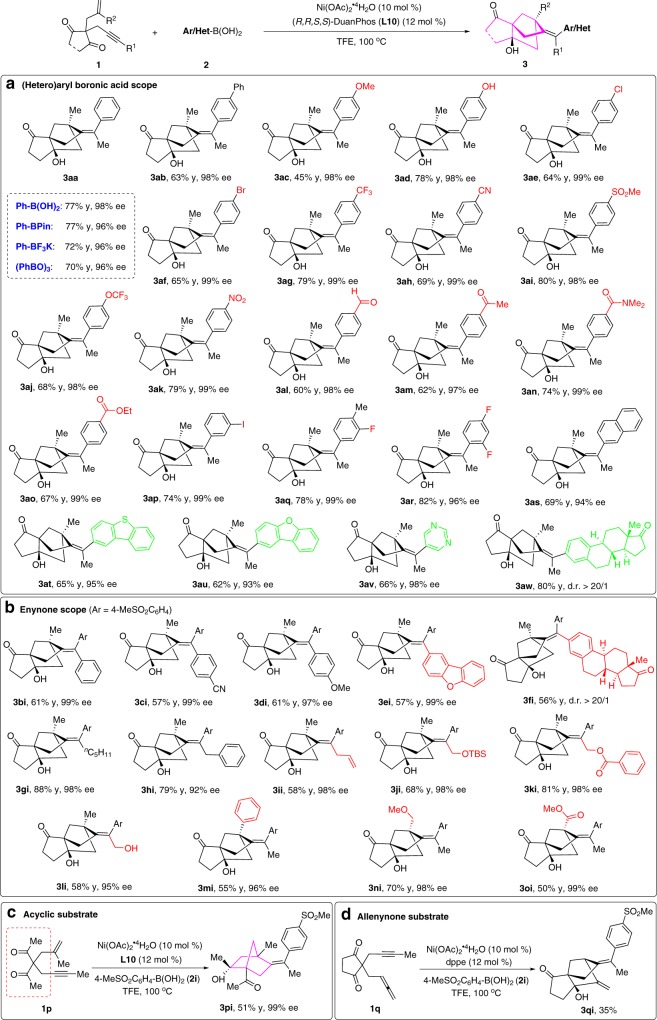
Substrate scope. **a** (Hetero)arylboronic acid scope. **b** Enynone scope. **c** Acyclic substrate. **d** Allenynone substrate.

With optimal reaction conditions in hand, we first investigated the effect of various aryl-boron reagents, such as PhB(OH)_2_, PhBPin (phenylboronic acid pinacol ester), PhBF_3_K and (PhBO)_3_ (phenylboroxine) (Fig. 4a). Interestingly, all these aryl-boron reagents are compatible. Among them, the Ph-B(OH)_2_ performed best comprehensively. The compatibility of the transformation with various (hetero)arylboronic acids was then evaluated in a robustness screening. A variety of *para*-substituted arylboronic acids could undergo tandem cyclization to provide the target bridged tricyclo[5.2.1.0^1,5^]decanes **3aa**-**3ao** in 45–80% yields and 97–99% ee. All of these *meta*- and *ortho*-substituted arylboronic acids proceeded smoothly, providing the corresponding products **3ap**-**3ar** in 74–82% yields and 96–99% ee. Notably, a series of synthetic valuable functional groups such as ether (**3ac**), free hydroxyl (**3ad**), chloride (**3ae**), bromide (**3af**), trifluoromethyl (**3ag**), cyano (**3ah**), sulfonyl (**3ai**), trifluoromethoxyl (**3aj**), nitro (**3ak**), aldehyde (**3al**), ketone (**3am**), amide (**3an**), ester (**3ao**), iodide (**3ap**) and fluoride (**3aq** and **3ar**) were all well-tolerated. In addition, various (hetero)arylboronic acids was also investigated. Naphthalene (**3as**), dibenzothiophene (**3at**) and dibenzofuran (**3au**) were successfully incorporated into the desired products in 62–69% yields and 93–95% ee. Remarkably, pyrimidine was perfectly accommodated to furnish **3av** in 66% yield with 98% ee, which exhibits a wide variety of biological activities^54^. Another interesting feature is that estrone could also be engaged in this route to afford the desired product **3aw** in good yield and high diastereoselectivity (80% yield, >20/1 d.r.), thus demonstrating the robustness and generality of this methodology for the modification of complex biologically active molecules. However, no reaction was observed using an alkyl or alkenyl boronic acids.

Next, the substrate scope of enynone **1** was explored in reactions with (4-(methylsulfonyl)phenyl)boronic acid **2i**, which furnished products **3bi**-**3oi** in synthetic useful yields and 92–99% ee (Fig. 4b). We first studied the influence of the substituents at the alkyne terminus (R^1^). The aryl group having an electron-donating or electron-withdrawing group at the alkyne terminus was found to be compatible, leading to the corresponding products **3bi**-**3di** in 57-61% yields and 97–99% ee. A dibenzofuran substituent which is very useful structural unit in organofunctional materials, could be successfully incorporated into the product **3ei** (57% yield, 99% ee). Moreover, estrone substituted substrate could also be employed in this type of reaction sequence to provide the desired **3fi** in high diastereoselectivity (>20/1 d.r.). The reaction is not limited to aryl group at the alkyne terminus, and alkyl-substituted alkynes such as *n*-pentyl, benzyl, allyl and those functionalized with CH_2_OTBS and CH_2_OBz were also suitable substrates. It is noteworthy that **1** **l** bearing a free hydroxyl group could be efficiently converted to **3li** in 58% yield with 95% ee. However, terminal alkyne provides complex mixtures of unidentified products. Then, we investigated the influence of substituents on the alkene moiety (R^2^). The introduction of a phenyl group at C_2_ of the propene moiety did not preclude the transformation, producing **3** **mi** in 55% yield with 96% ee. A methoxymethyl group was performed uniformly to give **3ni** in 70% yield and 98% ee at this position. Interestingly, substrate **1o** bearing an ester group on the double bond was also amenable to this transformation, giving the desired **3oi** in outstanding enantioselectivity (99% ee).

This transformation is not restricted to cyclic 1,3-diketone tethers, as substrate **1p** containing acyclic 1,3-diketone tethered 1,6-enyne is also highly effective. As shown in Fig. 4c, the highly functionalized bicyclo[2,2,1]heptane derivative **3pi** bearing three quaternary stereocenters could also be efficiently constructed in 99% ee. It is worth noting that this bicyclo[2.2.1]heptane ring system is also a very important skeleton found in many pharmacologically active molecules^55–57^.

We further prepared 1,3-cyclopentanedione tethered 1,6-diene substrate, however, the expected product was not obtained. Interestingly, substrate **1q** possessing allenyne tether could undergo an analogous cyclization reaction to afford the tricyclo[5.2.1.0^1,5^]decane **3qi** (Fig. 4d).

### Synthetic applications

**Fig. 5:**
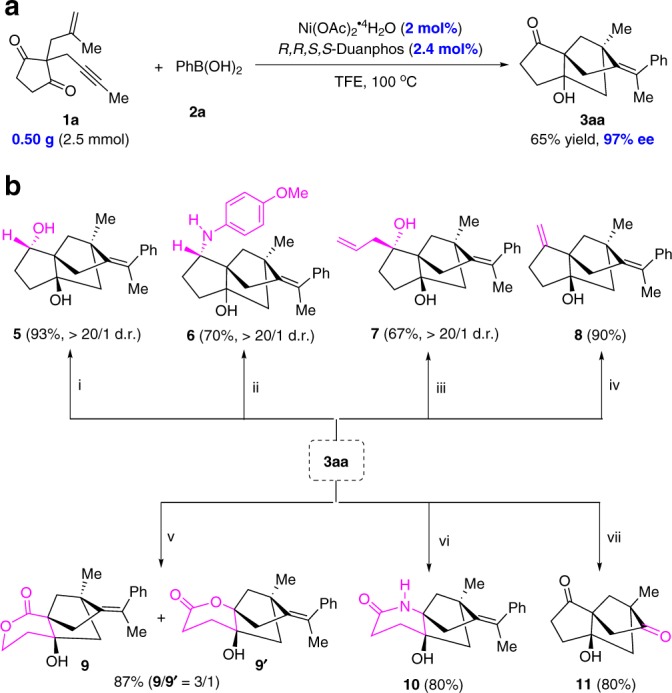
Half-gram scale reaction and synthetic manipulations. **a** Half-gram scale reaction. **b** Synthetic applications. (i) NaBH_4_, EtOH, 0 °C~ rt; (ii) 4-methoxyaniline (3 equiv), NaBH_3_CN, MeOH, rt; (iii) allylMgBr, THF, −78 °C-rt; (iv) PPh_3_MeBr, ^*t*^BuOK, THF, 0 °C-rt; (v) *m*-CPBA, NaHCO_3_, DCM, rt; (vi) NaN_3_, TFA/H_2_O = 4/1, 70 °C; (vii) O_3_/PPh_3_, DCM, −78 °C-rt. *m*-CPBA *m*-chloroperoxybenzoic acid, TFA trifluoroacetic acid.

We carried out a 0.5 g scale reaction of **1a** and found that the chiral Ni-catalyst loading as low as 2.0 mol % was sufficient to provide **3aa** in 65% yield with 97% ee, thus revealing the practical applicability of this Ni-catalyzed domino reaction (Fig. 5). To further demonstrate the synthetic benefit of our domino cyclization, Post-modifications on the tricyclo[5.2.1.0^1,5^]decane skeleton were performed (Fig. 5). Compound **3aa** could undergo a diastereoselective reduction by NaBH_4_ to form the corresponding alcohol **5** in 93% yield. Under reductive amination conditions with NaBH(OAc)_3_ and 4-methoxyaniline, **3aa** was converted into amine **6** in 70% yield with >20/1 diastereoselectivity. A further diastereoselective 1,2-addition of allylMgBr to **3aa** afforded the allyl alcohol **7** in 67% yield. Moreover, olefination of the ketone moiety of **3aa** with PPh_3_MeBr via the Wittig reaction gave a new alkene **8** in 90% yield. We further took advantage of the ketone moiety to generate the bridged tricyclic lactones **9** and **9’** with a ratio of 3:1 through the Baeyer-Villiger oxidation in the presence of *m-*CPBA. Interestingly, bridged tricyclic lactam **10** could also be selectively obtained in 80% yield via the Schmidt reaction with NaN_3_. Finally, the tetrasubstituted double bond was cleaved by ozonolysis with O_3_ to give optically pure tricyclic diketone **11** in 80% yield.

### Mechanistic investigation

**Fig. 6:**
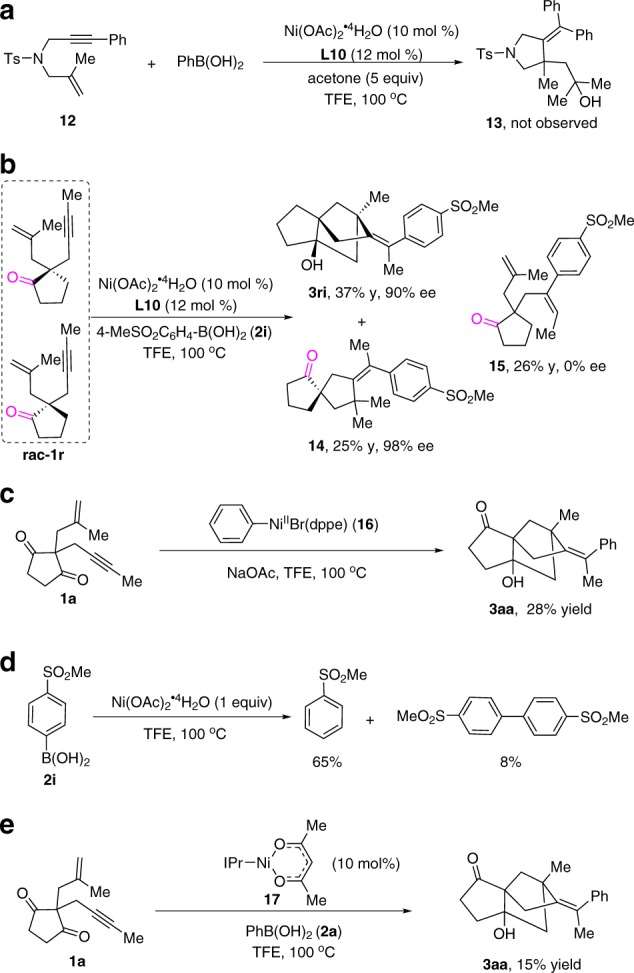
Mechanistic studies. **a** Three-component reaction with acetone. **b** The domino reaction used mono-carbonyl enyne substrate. **c** The use of stoichiometric aryl-Ni(II) complex in the reaction. **d** The stoichiometric reaction of Ni(OAc)_2_.4H_2_O with boronic acid. **e** The use of Ni(I) complex in the reaction.

We conducted preliminary mechanistic experiments to provide insight about the reaction mechanism. The reaction of enyne **12** with PhB(OH)_2_ in the presence of acetone resulted in a complex of mixture, the expected product **13** formed through enyne cyclization/intermolecular nucleophilic addition to acetone, was not observed (Fig. 6a). In addition, the reaction of mono-carbonyl enyne **1r** under our standard reaction conditions led to the formation of significant amount of the regioisomeric arylation product **15** (26% yield, Fig. 6b). These results indicate that these two ketone carbonyl groups on the substrate are very crucial. No enantiomeric excess was detected for product **15**, suggesting that the 1,2-addition of arylnickel species to alkyne might not be the turnover-limiting step. The formation of product **3ri** and **14** was likely a kinetic resolution process, where one of the enantiomer of racemic **1r** was transformed to the **3ri** in 37% yield with 90% ee, while the other was converted to **14** in 25% yield with 98% ee. These results clearly demonstrate that the Heck-cyclization process is the enantioselective-determining step of this transformation.

In order to further understand the reaction mechanism and catalytically active species of this transformation, aryl-Ni(II) complex **16** was prepared^6,58,59^, and the stoichiometric reaction of **16** with **1a** afforded **3aa** in 28% yield (Fig. 6c), implying that aryl-Ni(II) species is involved in the catalytic cycle. We performed a stoichiometric reaction of Ni(OAc)_2_.4H_2_O (1 equiv) and **2i** (1 equiv) in TFE, it was found that in addition to a deboronated product (65%), a biaryl product was also detected (Fig. 6d). This result indicates that Ni(II) was reduced to Ni(0) by reductive elimination of the corresponding diarylnickel(II) intermediate. Therefore, Ni(I) species may be generated by the disproportionation reaction between Ni(II) and Ni(0) species. IPrNi(acac) **17** was synthesized following the previously reported procedure^60^. This Ni(I) complex was also found to catalyze the domino cyclization of **1a** with PhB(OH)_2_ to afford **3aa** (Fig. 6e), thus indicating that an alternative mechanism involving aryl-Ni(I) species cannot be ruled out.

### Proposed reaction mechanism

**Fig. 7:**
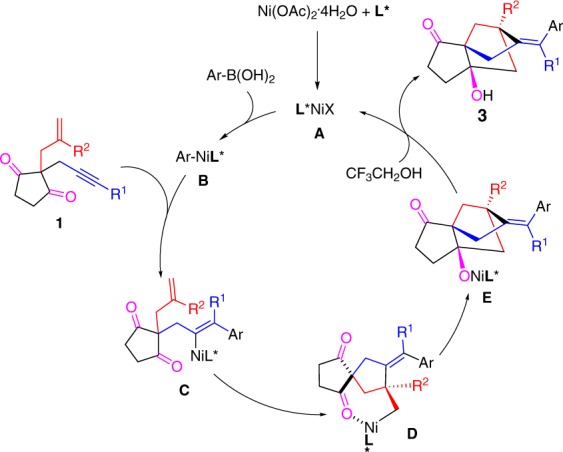
Reaction mechanism. Proposed catalytic cycle.

On the basis of the above results, a possible catalytic cycle for this transformation is proposed in Fig. 7. Transmetallation of arylboronic acid with the chiral nickel species **A** (X could be acetate, hydroxide or 2,2,2-trifluoroethoxide) gives the arylnickel complex **B**. The 1,2-addition of arylnickel species to the triple bond to form an alkenylnickel intermediate **C**. An intramolecular migratory insertion of alkenylnickel **C** into double bond affords the σ-alkylnickel intermediate **D**^61–64^. A subsequent nucleophilic cyclization of the resulting σ-alkylnickel species **D** onto one of the ketones leads to nickel alkoxide species **E**, followed by protonolysis to regenerate the catalytical active nickel catalyst and release the desired tricyclo[5.2.1.0^1,5^]decane product **3**. The oxidation state of nickel catalyst is still unclear, both Ni(I) and Ni(II) are possible.

## Discussion

In summary, a nickel-catalyzed asymmetric domino cyclization of 1,3-diketone tethered 1,6-enynes has been successfully developed, providing a general synthesis of optically pure bridged tricyclo[5.2.1.0^1,5^]decanes with three quaternary stereocenters in good yields and remarkable high levels of enantioselectivities (92–99% ee). This reaction is initiated by a regioselective 1,2-addition of arylboronic acid to the alkyne, followed by an enantioselective Heck-cyclization with unactivated alkene and nucleophilic cyclization of the resulting σ-alkylnickel species to the ketone group. Preliminary mechanistic studies indicate that either Ni(I) or Ni(II) species may be involved in the catalytic cycle.

## Methods

### Procedure for enantioselective synthesis of bridged tricyclo[5.2.1.0^1,5^]decanes

To an oven-dried sealed tube equipped with a PTFE-coated stir bar was charged with Ni(OAc)_2_.4H_2_O (0.01 mmol, 10 mol %), *1* *R*,*1’R*,*2* *S*,*2’S*-Duanphos (0.012 mmol, 12 mol %) and TFE (1 mL). This reaction mixture was stirred at room temperature for 15 minutes in an argon-filled glovebox. Enynone **1** (0.1 mmol) and (hetero)arylboronic acid **2** (0.2 mmol, 2 equiv) was then added. The sealed tube was sealed and removed from the glovebox. Then the mixture was stirred at 100 °C until the reaction was complete (monitored by TLC). The resulting mixture was concentrated under reduced pressure and purified by column chromatography on silica gel, eluting with petroleum ether/ethyl acetate 5/1~1/1 (v/v) to afford the corresponding product **3**.

## Supplementary information

Supplementary information

## Data Availability

The authors declare that all the data supporting the findings of this work are available within the article and its Supplementary Information files or from the corresponding author upon request. The X-ray crystallographic coordinates for structures reported in this study have been deposited at the Cambridge Crystallographic Data Centre (CCDC), under deposition numbers 1963288 (**3aa**). These data can be obtained free of charge from The Cambridge Crystallographic Data Centre via http://www.ccdc.cam.ac.uk/data_request/cif.
