# Supplementary material for: Synthesis of bridged tricyclo[5.2.1.01,5]decanes via nickel-catalyzed asymmetric domino cyclization of enynones
Source: Nat Commun. 2020 Apr 20;11:1882. doi: 10.1038/s41467-020-15837-1 (PMC7171102; doi:10.1038/s41467-020-15837-1)
Supplement: Supplementary file 1 — Supplementary information [file 41467_2020_15837_MOESM1_ESM.pdf]

Supplementary Information for

Synthesis of Bridged Tricyclo[5.2.1.0^{1,5}]decanes via Nickel-Catalyzed Asymmetric Domino Cyclization of Enynones

Chen *et al.*

Supplementary Methods

General information: ^{1}H and ^{13}C NMR data were recorded with Bruker ADVANCE III (400 MHz) or JNM-ECZ400S/L1 (400 MHz) spectrometers. Chemical shifts are given in ppm. The spectra are calibrated to the residual ^{1}H and ^{13}C signals of the solvents. Multiplicities are abbreviated as follows: singlet (s), doublet (d), triplet (t), quartet (q), doublet-doublet (dd), quintet (quint), septet (sept), multiplet (m), and broad (b). ^{19}F NMR spectra were recorded using $CFCl_3$ as internal standard. Gas chromatography were determined with a SHIMADZU Nexis GC 2030 gas chromatography instrument with a FID detector. High-resolution mass spectra (HRMS) were recorded on DIONEX UltiMate 3000 & Bruker Compact TOF mass spectrometer. Enantiomeric excesses were determined with a SHIMADZU LC-20ADXR system using chiral stationary phase columns (DAICEL) by comparing the samples with the corresponding racemic samples. Column and elution details were specified in each entry.

Unless otherwise stated, starting materials were purchased from commercial suppliers (Adamas-beta®, Alfa, Aldrich and so on). Mn powder (Alfa Aesar-140+325 mesh, 99.6% metals basis). All reactions dealing with air- or moisture-sensitive compounds were performed in the argon-filled glove box or by standard Schlenk techniques in oven-dried reaction vessels under argon atmosphere. Solvents were purchased in HPLC quality, degassed by purging thoroughly with argon and dried over activated molecular sieves of appropriate size. More sensitive compounds were stored in a desiccator or in a glove-box if required. Reactions were monitored by thin layer chromatography (TLC) using glass 0.25 mm silica gel plates. Compounds were visualized by UV-light at 254 nm and by dipping the plates in an aqueous potassium permanganate solution followed by heating. Flash column chromatography was performed over silica gel (200-400 mesh).

Supplementary Table 1. Optimization of reaction conditions[a]

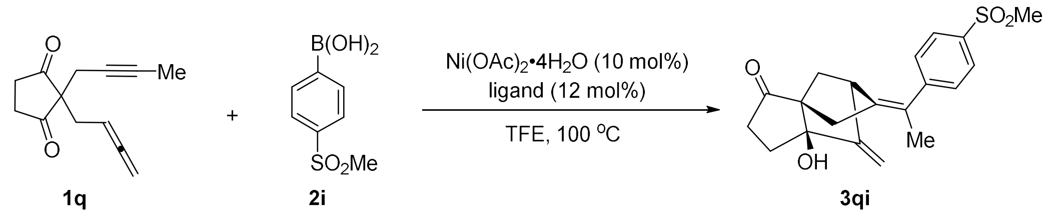

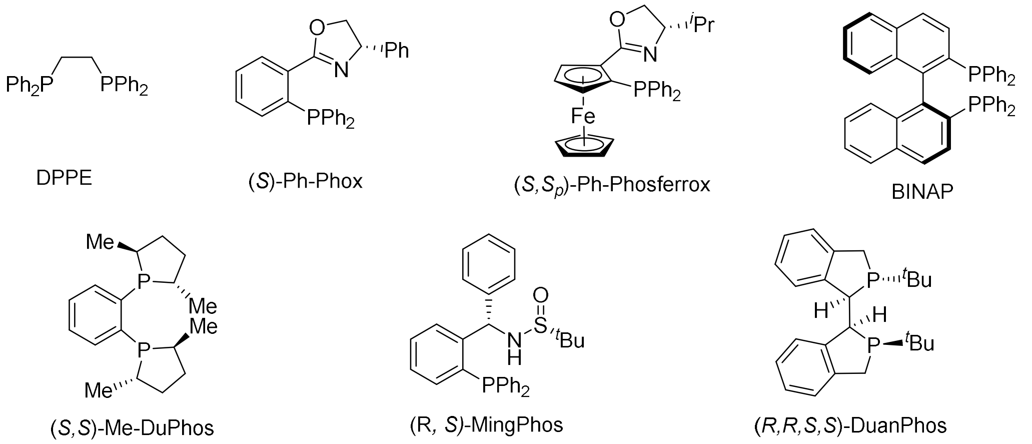

entry	ligand	yield of **3qi** (%)[b]
1	DPPE	35
2	(*S*)-Ph-Phox	no reaction
3	(*S,Sp*)-Ph-Phosferrox	no reaction
4	BINAP	no reaction
5	(*S,S*)-Me-DuPhos	trace
6	(*R, S*)-MingPhos	no reaction
7	(*R,R,S,S*)-DuanPhos	trace

[a] Reaction condition: **1q** (0.1 mmol), **2i** (2 equiv), $Ni(OAc)_2\cdot 4H_2O$ (0.1 equiv), ligand (0.12 equiv), TFE (1 mL) at 100 °C for 48 h. [b] yields of isolated products. TFE = 2,2,2-Trifluoroethanol.

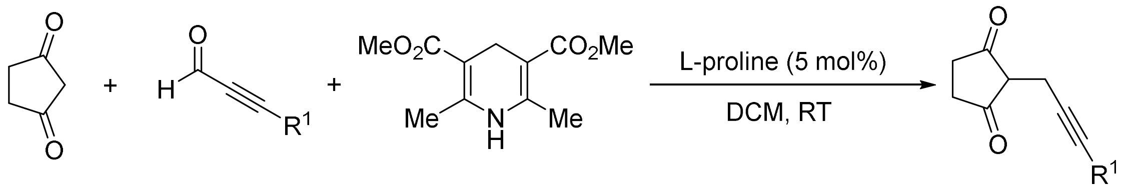

Supplementary Figure 1. Propargylation of cyclopentane-1,3-dione

Procedure for the propargylation of cyclopentane-1,3-dione:[1,2] To a solution of cyclopentane-1,3-dione (1 equiv), alkynaldehyde (1.5 equiv) and Hantzsch ester (1 equiv) in DCM (0.3 M) was added L-proline (0.05 equiv). The reaction mixture was stirred at room temperature for 24 hours until the reaction was complete (monitored by TLC). The reaction mixture was then concentrated in vacuo and purified by column chromatography on silica gel, eluting with petroleum ether/ethyl acetate 20/1~2/1 (v/v) to afford the desired propargylic cyclopentane-1,3-dione.

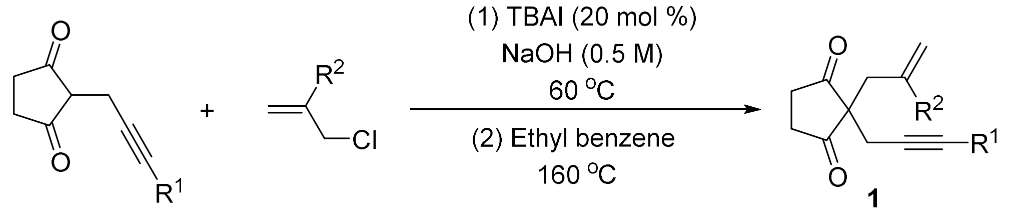

1

Supplementary Figure 2. Allylation of cyclopentane-1,3-dione

Procedure for the allylation of cyclopentane-1,3-dione: To a solution of propargylic cyclopentane-1,3-dione (1 equiv) and TBAI (1 equiv) in 0.5 N NaOH aqueous solution (0.5 M) was added allyl chloride (2 equiv). The mixture was heated at 60 °C for 24 hours until propargylic cyclopentane-1,3-dione was completely consumed (monitored by TLC). The reaction mixture was then extracted with ethyl acetate. The combined organic layer was washed with H_2O, dried over anhydrous Na_2SO_4, and concentrated under reduced pressure. The resulting crude O-allylation product was dissolved in ethyl benzene (0.5 M) and heated at 160 °C for 24 hours until the reaction was complete (monitored by TLC). The mixture was then concentrated under reduced pressure and purified by silica-gel column chromatography, eluting with petroleum ether/ethyl acetate 10/1~2/1(v/v) to provide the corresponding substrate **1**.

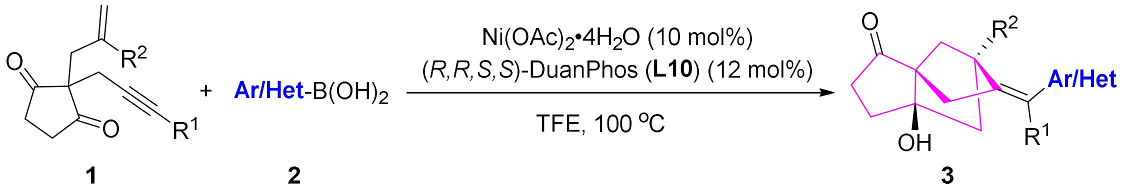

Supplementary Figure 3. Synthesis of bridged tricyclo[5.2.1.0^{1,5}]decanes.

General procedure for the enantioselective synthesis of bridged tricyclo[5.2.1.0^{1,5}]decanes: An oven-dried sealed tube equipped with a PTFE-coated stir bar was charged with $Ni(OAc)_2 \cdot 4H_2O$ (0.01 mmol, 2.5 mg, 10 mol%), *1R,1'R,2S,2'S*-Duanphos (**L10**) (0.012 mmol, 4.6 mg, 12 mol%) and TFE (1 mL). This reaction mixture was stirred at room temperature for 15 minutes in an argon-filled glovebox. Substrate **1** (0.1 mmol) and (hetero)aryl boronic acid **2** (0.2 mmol, 2 equiv) was then added. The sealed tube was sealed and removed from the glovebox. Then the mixture was stirred at 100 °C until the reaction was complete (monitored by TLC). The resulting mixture was concentrated under reduced pressure and purified by column chromatography on silica gel, eluting with petroleum ether/ethyl acetate 5/1~1/1 (v/v) to afford the corresponding product **3**.

3a-hydroxy-4-methyl-6a-(2-methylallyl)-5-phenyl-3,3a,6,6a-tetrahydropentalen-1(2*H*)-one (**4aa**)(Table 1, entry 1)

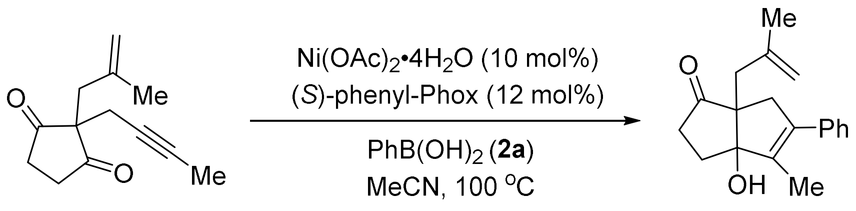

Supplementary Figure 4. Synthesis of 4aa

Procedure for the synthesis of 4aa: An oven-dried sealed tube equipped with a PTFE-coated stir bar was charged with $Ni(OAc)_2 \cdot 4H_2O$ (0.01 mmol, 2.5 mg, 10 mol%), (*S*)-phenyl-Phox (0.012 mmol, 4.8 mg, 12 mol%) and MeCN (1 mL). This reaction mixture was stirred at room temperature for 15 minutes in an argon-filled glovebox. Substrate **1a** (0.1 mmol, 20.0 mg) and $PhB(OH)_2$ **2a** (0.2 mmol, 24.5 mg) was then added. The sealed tube was sealed and removed from the glovebox. Then the reaction was stirred at 100 °C until the reaction was complete (monitored by TLC). The resulting mixture was concentrated under reduced pressure and purified by column chromatography on silica gel, eluting with petroleum ether/ethyl acetate 5/1~2/1 (v/v) to afford the product **4aa** (17.0 mg, 60% yield). ^1H NMR (400 MHz, $CDCl_3$) δ 7.37-7.30 (m, 2H), 7.29-7.21 (m, 3H), 4.89-4.85 (m, 1H), 4.84-4.81 (m, 1H), 2.99-2.75 (m, 2H), 2.56-2.33 (m, 4H), 2.15-2.00 (m, 2H), 1.89-1.85 (m, 1H), 1.61 (t, *J* = 4.0 Hz, 3H), 1.79 (s, 3H); ^{13}C NMR (101 MHz, $CDCl_3$) δ 220.7, 143.1, 139.9, 136.7, 135.6, 128.2, 127.7, 127.4, 114.8, 92.9, 92.9, 77.3, 77.0, 76.7, 59.0, 44.5, 39.3, 36.7, 29.6, 24.1, 10.6.

(*3aS,6R,7aR,Z*)-7a-hydroxy-6-methyl-5-(1-phenylethylidene)hexahydro-3a,6-methanoinden-3(2*H*)-one (**3aa**)

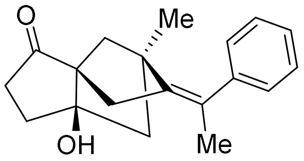

Chemical Formula: $C_{19}H_{22}O_2$
Exact Mass: 282.1620

3aa was prepared according to general procedure using **1a** (0.1 mmol, 20.4 mg) and **2a** and was purified by silica gel column chromatography (petroleum ether/ethyl acetate = 10/1~2/1) to obtain **3aa** (21.7 mg, 77% yield). ^{1}H NMR (400 MHz, $CDCl_3$) δ 7.29-7.17 (m, 3H), 7.14-7.04 (m, 2H), 2.76-2.68 (m, 1H), 2.65-2.47 (m, 3H), 2.31-2.20 (m, 1H), 2.15-2.05 (m, 1H), 2.01 (d, *J* = 12.7 Hz, 1H), 1.92 (s, 3H), 1.81 (dd, *J* = 12.7, 3.5 Hz, 1H), 1.73 (bs, 1H), 1.67 (dd, *J* = 9.7, 3.5 Hz, 1H), 1.61 (dd, *J* = 9.6, 3.6 Hz, 1H), 0.56 (s, 3H);

^{13}C NMR (101 MHz, $CDCl_3$) δ 217.5, 144.1, 138.3, 128.9, 128.4, 127.7, 126.3, 86.7, 64.4, 55.6, 54.0, 52.9, 38.2, 35.8, 33.5, 23.3, 20.3;

HRMS: (ESI) calcd for $C_{19}H_{23}O_2^+$[M+H]$^+$ 283.1693; found 283.1704.

The enantiomeric purity was established by HPLC analysis using a chiral column: AD-H column, 30 °C, *n*-Hexane/*i*-Propanol = 85/15 as eluent, 254 nm, 1 mL/min. tR = 4.6 min (minor), 5.3 min (major).

Optical Rotation: $[\alpha]_D^{23}$ -281.0 (c 0.3, ^{i}PrOH) for 98% ee.

The absolute configuration of **3aa** was determined by X-ray crystallographic analysis.

〈色谱图〉

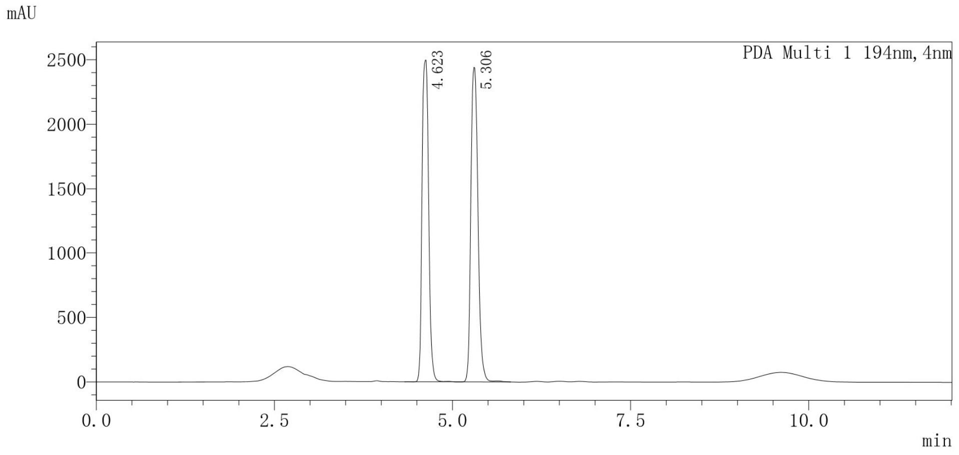

〈峰表〉

PDA Ch1 194nm

峰号	保留时间	面积	高度	浓度	浓度单位	标记	化合物名
1	4.623	16035535	2496552	0.000		M	
2	5.306	16926613	2441453	0.000		M	
总计		32962148	4938005				

peak number → 峰号; retention time → 保留时间; area → 面积; height → 高度

Supplementary Figure 5. HPLC spectrum of 3aa

〈色谱图〉

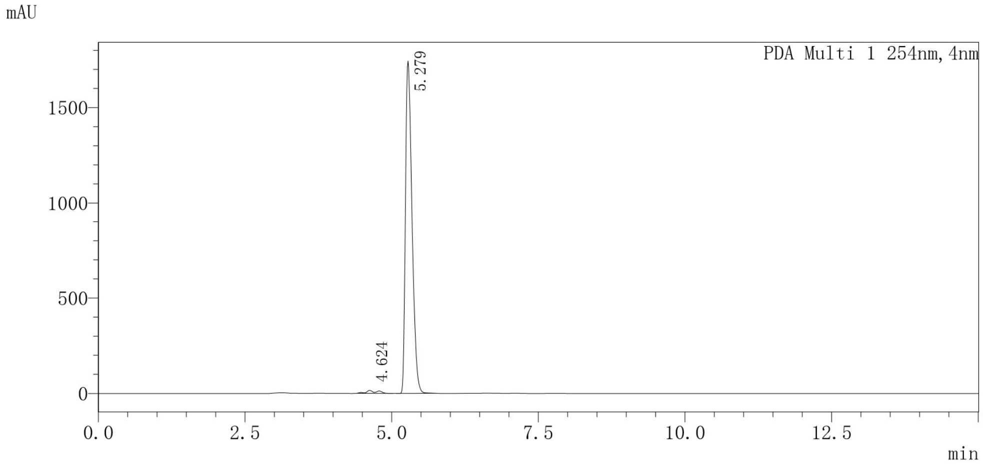

〈峰表〉

PDA Ch1 254nm

峰号	保留时间	面积	高度	浓度	浓度单位	标记	化合物名
1	4.624	202410	15492	0.000		M	
2	5.279	13540819	1743870	0.000		M	
总计		13743229	1759362				

peak number → 峰号; retention time → 保留时间; area → 面积; height → 高度

Supplementary Figure 6. HPLC spectrum of (*3aS,6R,7aR,Z*)-3aa

(*3aS,6R,7aR,Z*)-5-(1-([1,1'-biphenyl]-4-yl)ethylidene)-7a-hydroxy-6-methylhexahydro-3a,6-methanoinden-3(2*H*)-one (**3ab**)

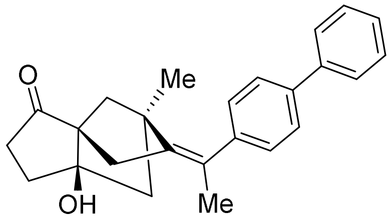

Chemical Formula: $C_{25}H_{26}O_2$
Exact Mass: 358.1933

3ab was prepared according to general procedure using **1a** (0.1 mmol, 20.4 mg) and **2b** and was purified by silica gel column chromatography (petroleum ether/ethyl acetate = 10/1~2/1) to obtain **3ab** (22.6 mg, 63% yield). ^{1}H NMR (400 MHz, $CDCl_3$) δ 7.64-7.59 (m, 2H), 7.53 (d, *J* = 8.0 Hz, 2H), 7.46-7.41 (m, 2H), 7.36-7.30 (m, 1H), 7.19 (d, *J* = 8.0 Hz, 2H), 2.80-2.50 (m, 4H), 2.33-2.23 (m, 1H), 2.18-2.02 (m, 2H), 1.97 (s, 3H), 1.90-1.84 (m, 1H), 1.73-1.61 (m, 3H), 0.66 (s, 3H);

^{13}C NMR (101 MHz, $CDCl_3$) δ 217.5, 143.1, 140.8, 139.1, 138.7, 128.9, 128.7, 128.4, 127.1, 126.9, 126.4, 86.7, 64.4, 55.6, 54.1, 52.9, 38.2, 35.9, 33.6, 23.2, 20.4;

HRMS: (ESI) calcd for $C_{25}H_{27}O_2^+$[M+H]$^+$ 359.2006; found 359.2006.

The enantiomeric purity was established by HPLC analysis using a chiral column: AD-H column, 30 °C, *n*-Hexane/*i*-Propanol = 85/15 as eluent, 254 nm, 1 mL/min. tR = 6.0 min (minor), 7.1 min (major).

Optical Rotation: $[\alpha]_D^{25}$ -53.8 (c 0.8, ^{i}PrOH) for 98% ee.

Absolute stereochemistry was determined through analogy with **3aa**.

〈色谱图〉

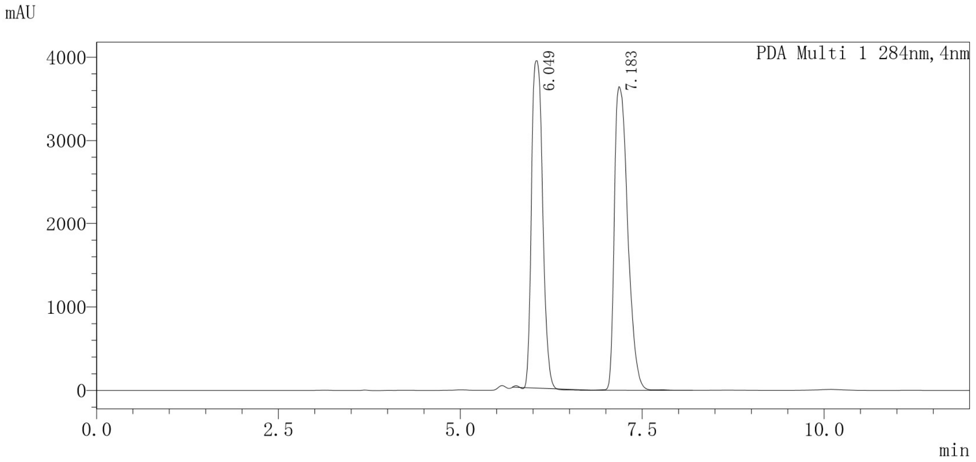

〈峰表〉

PDA Ch1 284nm

峰号	保留时间	面积	高度	浓度	浓度单位	标记	化合物名
1	6.049	41623584	3929980	0.000		M	
2	7.183	46657286	3644865	0.000		M	
总计		88280870	7574846				

peak number
area
height
retention time

Supplementary Figure 7. HPLC spectrum of 3ab

〈色谱图〉

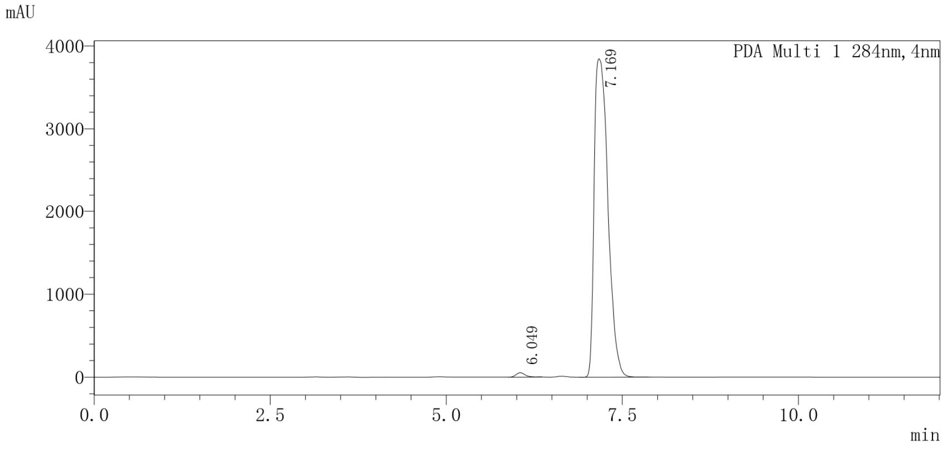

〈峰表〉

PDA Ch1 284nm

峰号	保留时间	面积	高度	浓度	浓度单位	标记	化合物名
1	6.049	477595	53729	0.000		M	
2	7.169	52236500	3847336	0.000		M	
总计		52714095	3901065				

peak number
area
height
retention time

Supplementary Figure 8. HPLC spectrum of (*3aS,6R,7aR,Z*)-3ab

(*3aS,6R,7aR,Z*)-7a-hydroxy-5-(1-(4-methoxyphenyl)ethylidene)-6-methylhexahydro-3a,6-methanoinden-3(2*H*)-one (**3ac**)

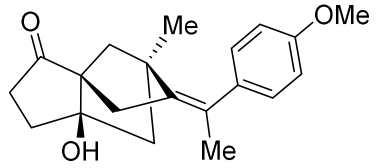

Chemical Formula: $C_{20}H_{24}O_3$
Exact Mass: 312.1725

3ac was prepared according to general procedure using **1a** (0.1 mmol, 20.4 mg) and **2c** and was purified by silica gel column chromatography (petroleum ether/ethyl acetate = 20/1~1/1) to obtain **3ac** (14.0 mg, 45% yield). ^{1}H NMR (400 MHz, $CDCl_3$) δ 7.02 (d, *J* = 8.4 Hz, 2H), 6.81 (d, *J* = 8.4 Hz, 2H), 3.80 (s, 3H), 2.73-2.49 (m, 4H), 2.30-2.23 (m, 1H), 2.16-2.08 (m, 1H), 2.02 (m, 1H), 1.90 (s, 3H), 1.83-1.79 (m, 1H), 1.70-1.60 (m, 3H), 0.61 (s, 3H);

^{13}C NMR (101 MHz, $CDCl_3$) δ 217.6, 158.1, 138.3, 136.4, 129.4, 128.4, 113.1, 86.7, 64.4, 55.6, 55.2, 54.1, 52.9, 38.2, 35.8, 33.6, 23.4, 20.3;

HRMS: (ESI) calcd for $C_{20}H_{25}O_3^+$[M+H]$^+$ 313.1798; found 313.1798.

The enantiomeric purity was established by HPLC analysis using a chiral column: AD-H column, 30 °C, *n*-Hexane/*i*-Propanol = 85/15 as eluent, 254 nm, 1 mL/min. tR = 6.6 min (minor), 7.2 min (major).

Optical Rotation: $[\alpha]_D^{25}$ -98.5 (c 0.5, ^{i}PrOH) for 98% ee.

Absolute stereochemistry was determined through analogy with **3aa**.

〈色谱图〉
mAU

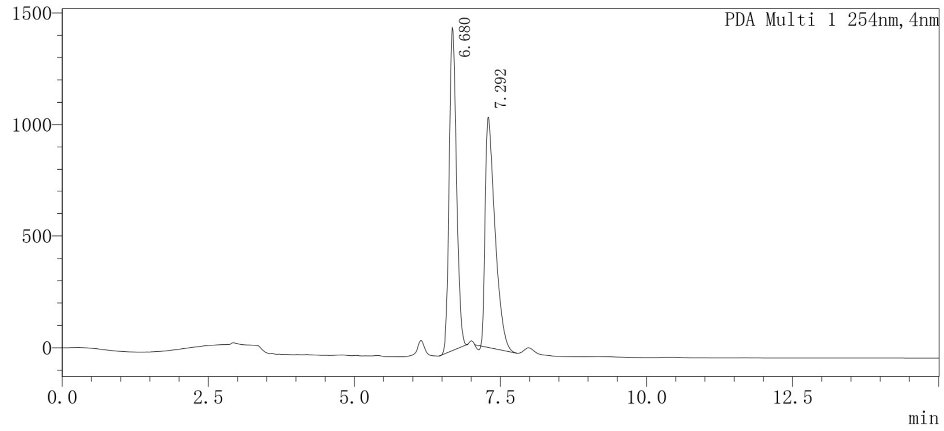

〈峰表〉
PDA Ch1 254nm

峰号	保留时间	面积	高度	浓度	浓度单位	标记	化合物名
1	6.680	12584648	1448264	0.000		M	
2	7.292	12048629	1032393	0.000		M	
总计		24633277	2480657				

peak number
retention time
area
height

Supplementary Figure 9. HPLC spectrum of 3ac

〈色谱图〉
mAU

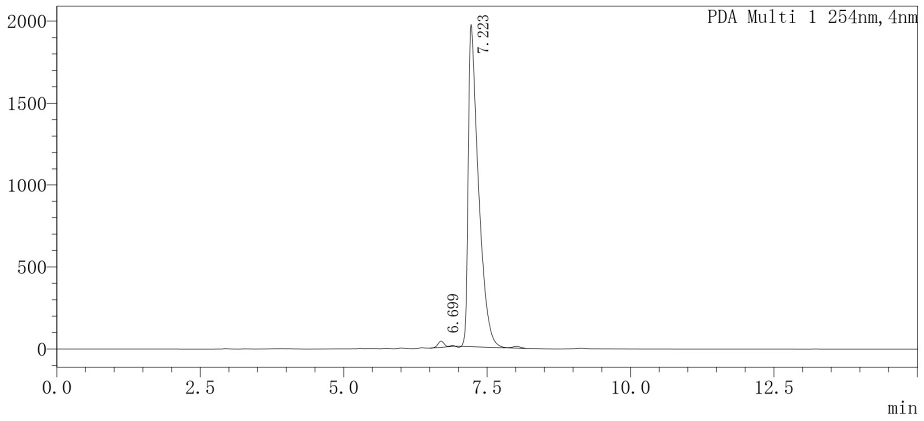

〈峰表〉
PDA Ch1 254nm

峰号	保留时间	面积	高度	浓度	浓度单位	标记	化合物名
1	6.699	307143	37463	0.000		M	
2	7.223	24429724	1966077	0.000		M	
总计		24736867	2003541				

peak number
retention time
area
height

Supplementary Figure 10. HPLC spectrum of (*3aS,6R,7aR,Z*)-3ac

(*3aS,6R,7aR,Z*)-7a-hydroxy-5-(1-(4-hydroxyphenyl)ethylidene)-6-methylhexahydro-3a,6-methanoinden-3(2*H*)-one (**3ad**)

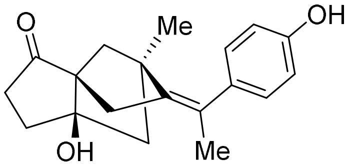

Chemical Formula: $C_{19}H_{22}O_3$
Exact Mass: 298.1569

3ad was prepared according to general procedure using **1a** (0.1 mmol, 20.4 mg) and **2d** and was purified by silica gel column chromatography (petroleum ether/ethyl acetate = 10/1~1/1) to obtain **3ad** (23.2 mg, 78% yield). ^{1}H NMR (400 MHz, CD_3CN) δ 6.96 (d, *J* = 8.6 Hz, 2H), 6.72 (d, *J* = 8.6 Hz, 2H), 3.17 (s, 1H), 2.56-2.49 (m, 2H), 2.47-2.40 (m, 2H), 2.17-2.08 (m, 2H), 1.94 (dd, *J* = 5.0, 2.5 Hz, 2H), 1.85 (s, 3H), 1.80-1.72 (m, 2H), 1.43 (dd, *J* = 9.6, 3.6 Hz, 1H), 0.58 (s, 3H);

^{13}C NMR (151 MHz, CD_3CN) δ 218.8, 156.7, 140.5, 137.3, 131.0, 128.6, 115.7, 87.3, 65.6, 55.6, 55.1, 53.7, 38.9, 36.9, 34.7, 23.9, 21.1;

HRMS: (ESI) calcd for $C_{19}H_{23}O_3^+$[M+H]$^+$ 299.1642; found 299.1642.

The enantiomeric purity was established by HPLC analysis using a chiral column: OD-H column, 30 °C, *n*-Hexane/*i*-Propanol = 85/15 as eluent, 254 nm, 1 mL/min. tR = 8.5 min (major), 10.4 min (minor).

Optical Rotation: $[\alpha]_D^{25}$ -47.3 (c 0.6, ^{i}PrOH) for 98% ee.

Absolute stereochemistry was determined through analogy with **3aa**.

〈色谱图〉

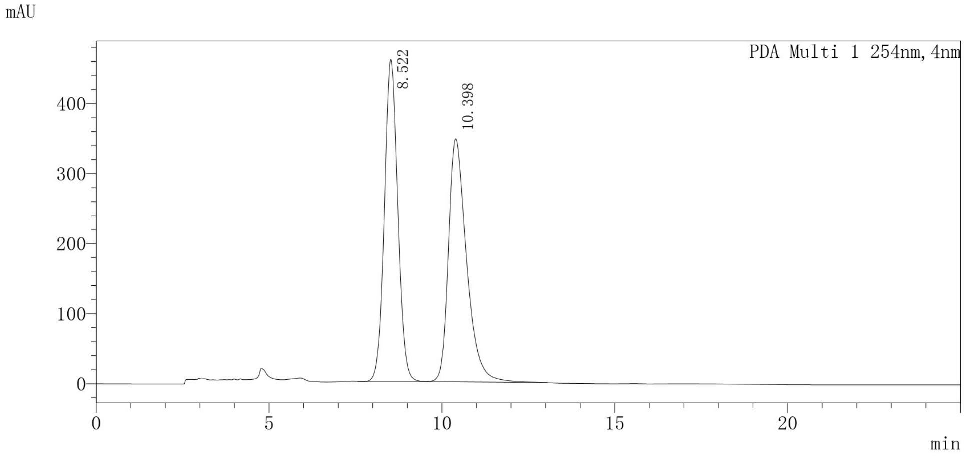

〈峰表〉

PDA Ch1 254nm

峰号	保留时间	面积	高度	浓度	浓度单位	标记	化合物名
1	8.522	12353089	459821	0.000		M	
2	10.398	12658542	347075	0.000		M	
总计		25011631	806896				

peak number　retention time　area　height

Supplementary Figure 11. HPLC spectrum of 3ad

〈色谱图〉

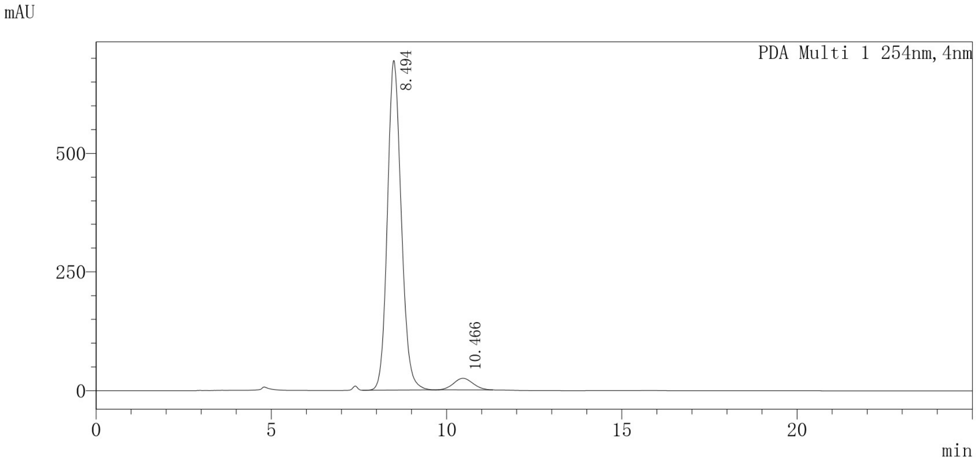

〈峰表〉

PDA Ch1 254nm

峰号	保留时间	面积	高度	浓度	浓度单位	标记	化合物名
1	8.494	19037901	694184	0.000		M	
2	10.466	933808	24381	0.000		M	
总计		19971709	718565				

peak number　retention time　area　height

Supplementary Figure 12. HPLC spectrum of (*3aS,6R,7aR,Z*)-3ad

(*3aS,6R,7aR,Z*)-5-(1-(4-chlorophenyl)ethylidene)-7a-hydroxy-6-methylhexahydro-3a,6-methanoinden-3(2*H*)-one (**3ae**)

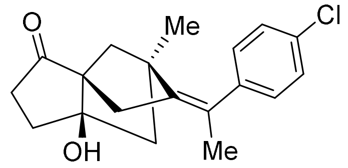

Chemical Formula: $C_{19}H_{21}ClO_2$
Exact Mass: 316.1230

3ae was prepared according to general procedure using **1a** (0.1 mmol, 20.4 mg) and **2e** and was purified by silica gel column chromatography (petroleum ether/ethyl acetate = 20/1~2/1) to obtain **3ae** (20.2 mg, 64% yield). ^1H NMR (400 MHz, $CDCl_3$) δ 7.24 (d, *J* = 8.2 Hz 2H), 7.04 (d, *J* = 8.2 Hz, 2H), 2.72-2.48 (m, 4H), 2.30-2.22 (m, 1H), 2.17-2.07 (m, 1H), 2.04-1.99 (m, 1H), 1.89 (s, 3H), 1.88-1.81 (m, 1H), 1.81-1.76 (m, 1H), 1.70-1.66 (m, 1H), 1.63-1.59 (m, 1H), 0.60 (s, 3H);

^{13}C NMR (101 MHz, $CDCl_3$) δ 217.4, 142.5, 139.3, 132.1, 129.8, 127.9, 127.5, 86.6, 64.3, 55.4, 54.0, 52.8, 38.1, 35.9, 33.6, 23.1, 20.5;

HRMS: (ESI) calcd for $C_{19}H_{22}ClO_2^+$[M+H]$^+$ 317.1303; found 317.1306.

The enantiomeric purity was established by HPLC analysis using a chiral column: AD-H column, 30 °C, *n*-Hexane/*i*-Propanol = 85/15 as eluent, 254 nm, 1 mL/min. tR = 5.5 min (minor), 6.7 min (major).

Optical Rotation: $[\alpha]_D^{25}$ -70.6 (c 0.7, ^{i}PrOH) for 99% ee.

Absolute stereochemistry was determined through analogy with **3aa**.

<色谱图>

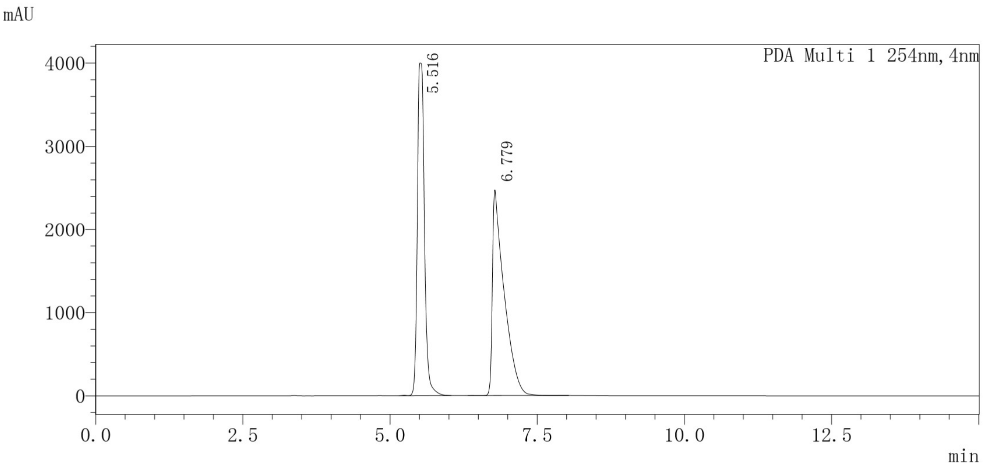

<峰表>

PDA Ch1 254nm

峰号	保留时间	面积	高度	浓度	浓度单位	标记	化合物名
1	5.516	33301232	3997668	0.000		M	
2	6.779	34167350	2475662	0.000		M	
总计		67468582	6473330				

peak number
area
height
retention time

Supplementary Figure 13. HPLC spectrum of 3ae

<色谱图>

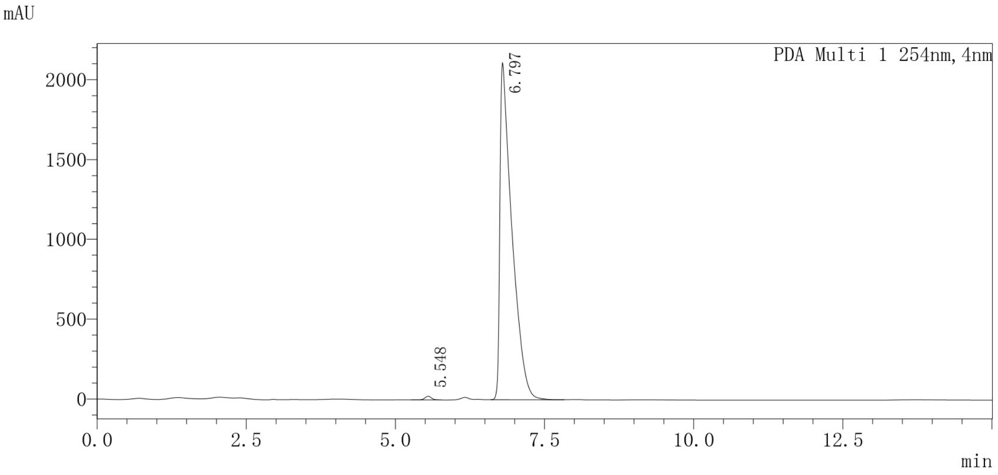

<峰表>

PDA Ch1 254nm

峰号	保留时间	面积	高度	浓度	浓度单位	标记	化合物名
1	5.548	186791	23482	0.000		M	
2	6.797	29482003	2111477	0.000		M	
总计		29668794	2134959				

peak number
area
height
retention time

Supplementary Figure 14. HPLC spectrum of (*3aS,6R,7aR,Z*)-3ae

(*3aS,6R,7aR,Z*)-5-(1-(4-bromophenyl)ethylidene)-7a-hydroxy-6-methylhexahydro-3a,6-methanoinden-3(2*H*)-one (**3af**)

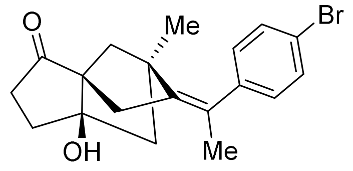

Chemical Formula: $C_{19}H_{21}BrO_2$
Exact Mass: 360.0725

3af was prepared according to general procedure using **1a** (0.1 mmol, 20.4 mg) and **2f** and was purified by silica gel column chromatography (petroleum ether/ethyl acetate = 20/1~2/1) to obtain **3af** (23.4 mg, 65% yield). ^{1}H NMR (400 MHz, $CDCl_3$) δ 7.40 (d, *J* = 8.4 Hz, 2H), 6.99 (d, *J* = 8.2 Hz, 2H), 2.74-2.43 (m, 4H), 2.30-2.20 (m, 1H), 2.17-2.06 (m, 1H), 2.05-1.98 (m, 1H), 1.89 (s, 3H), 1.83 (bs, 1H), 1.78 (dd, *J* = 12.7, 3.5 Hz, 1H), 1.68 (dd, *J* = 9.8, 3.4 Hz, 1H), 1.61 (dd, *J* = 9.8, 3.5 Hz, 1H), 0.60 (s, 3H);

^{13}C NMR (101 MHz, $CDCl_3$) δ 217.4, 143.0, 139.4, 130.9, 130.2, 127.4, 120.2, 86.6, 64.3, 55.3, 54.0, 52.8, 38.1, 35.9, 33.6, 23.1, 20.5;

HRMS: (ESI) calcd for $C_{19}H_{21}BrO_2Na^+$[M+Na]$^+$ 383.0617; found 383.0606.

The enantiomeric purity was established by HPLC analysis using a chiral column: AD-H column, 30 °C, *n*-Hexane/*i*-Propanol = 85/15 as eluent, 254 nm, 1 mL/min. tR = 5.6 min (minor), 6.8 min (major).

Optical Rotation: $[\alpha]_D^{25}$ -95.5 (c 0.8, ^{i}PrOH) for 99% ee.

Absolute stereochemistry was determined through analogy with **3aa**.

<色谱图>

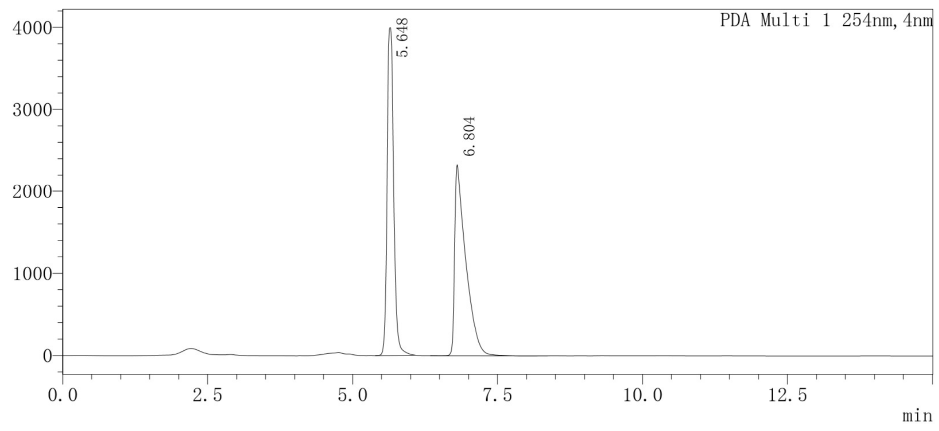

<峰表>

PDA Ch1 254nm

峰号	保留时间	面积	高度	浓度	浓度单位	标记	化合物名
1	5.648	31272638	3996024	0.000		M	
2	6.804	31045425	2330970	0.000		M	
总计		62318064	6326994				

peak number area height

retention time

Supplementary Figure 15. HPLC spectrum of 3af

<色谱图>

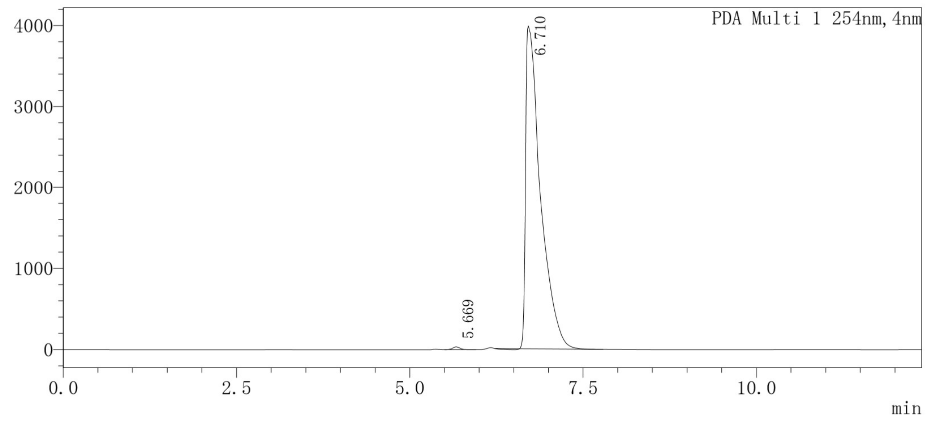

<峰表>

PDA Ch1 254nm

峰号	保留时间	面积	高度	浓度	浓度单位	标记	化合物名
1	5.669	240360	33180	0.000		M	
2	6.710	58980583	3985120	0.000		M	
总计		59220943	4018300				

peak number area height

retention time

Supplementary Figure 16. HPLC spectrum of (*3aS,6R,7aR,Z*)-3af

(*3aS,6R,7aR,Z*)-7a-hydroxy-6-methyl-5-(1-(4-(trifluoromethyl)phenyl)ethylidene) hexahydro-3a,6-methanoinden-3(2*H*)-one (**3ag**)

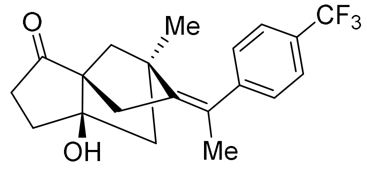

Chemical Formula: $C_{20}H_{21}F_3O_2$
Exact Mass: 350.1494

3ag was prepared according to general procedure using **1a** (0.1 mmol, 20.4 mg) and **2g** and was purified by silica gel column chromatography (petroleum ether/ethyl acetate = 20/1~2/1) to obtain **3ag** (27.7 mg, 79% yield). ^{1}H NMR (400 MHz, $CDCl_3$) δ 7.53 (d, *J* = 8.0 Hz, 2H), 7.23 (d, *J* = 7.7 Hz, 2H), 2.76-2.46 (m, 4H), 2.31-2.21 (m, 1H), 2.20-2.07 (m, 1H), 2.02 (d, *J* = 12.7 Hz, 1H), 1.99-1.84 (bs, 1H), 1.91 (s, 3H), 1.80 (dd, *J* = 12.7, 3.4 Hz, 1H), 1.70 (dd, *J* = 9.8, 3.1 Hz, 1H), 1.62 (dd, *J* = 9.8, 3.5 Hz, 1H), 0.56 (s, 3H);

^{13}C NMR (101 MHz, $CDCl_3$) δ 217.4, 147.91 (d, *J* = 1.6 Hz), 139.9, 128.8, 128.6 (d, *J* = 32.1 Hz), 127.4, 124.7 (q, *J* = 3.2, 2.3 Hz), 124.2 (q, *J* = 272.0 Hz), 86.5, 64.3, 55.3, 54.0, 52.8, 38.1, 36.0, 33.6, 23.0, 20.5;

^{19}F NMR (376 MHz, $CDCl_3$) δ -62.2 (s);

HRMS: (ESI) calcd for $C_{20}H_{22}F_3O_2^+$[M+H]$^+$ 351.1566; found 351.1566.

The enantiomeric purity was established by HPLC analysis using a chiral column: AD-H column, 30 °C, *n*-Hexane/*i*-Propanol = 85/15 as eluent, 254 nm, 1 mL/min. tR = 5.2 min (minor), 6.3 min (major).

Optical Rotation: $[\alpha]_D^{25}$ -47.2 (c 0.9, ^{i}PrOH) for 99% ee.

Absolute stereochemistry was determined through analogy with **3aa**.

〈色谱图〉

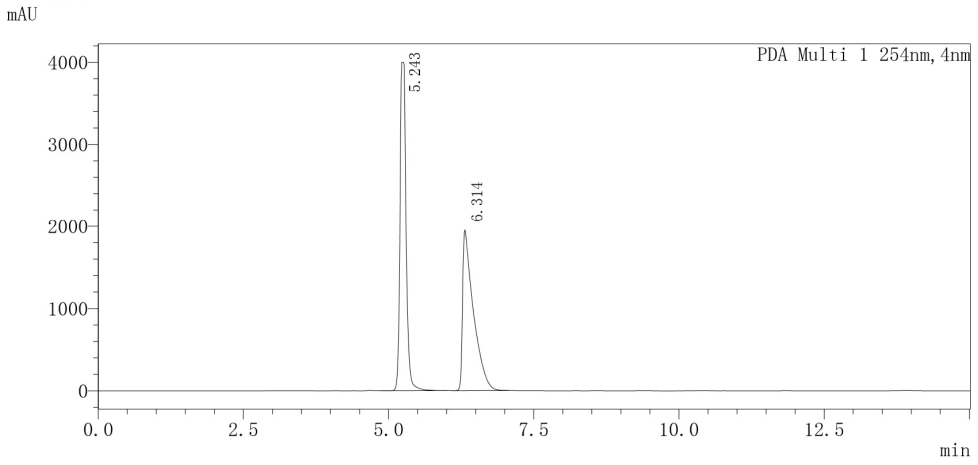

〈峰表〉

PDA Ch1 254nm

峰号	保留时间	面积	高度	浓度	浓度单位	标记	化合物名
1	5.243	28978185	3998214	0.000		M	
2	6.314	25283057	1954294	0.000		M	
总计		54261242	5952508				

peak number
retention time
area
height

Supplementary Figure 17. HPLC spectrum of 3ag

〈色谱图〉

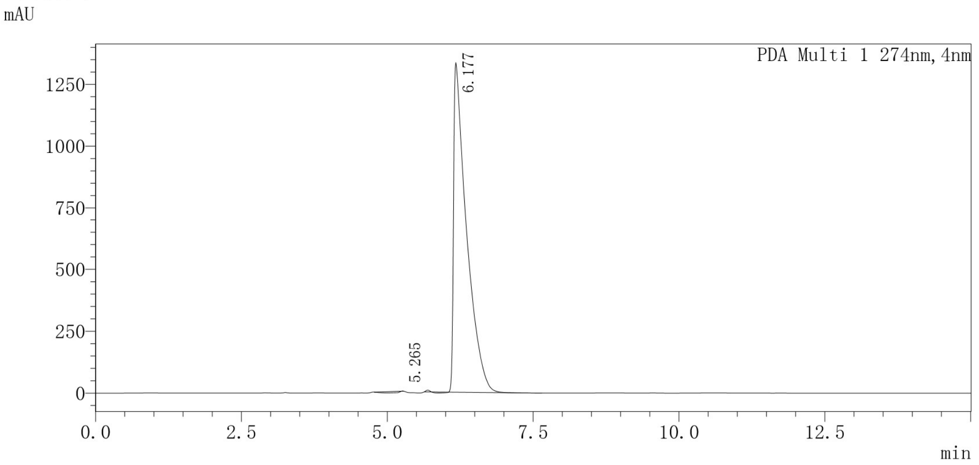

〈峰表〉

PDA Ch1 274nm

峰号	保留时间	面积	高度	浓度	浓度单位	标记	化合物名
1	5.265	-109703	529	0.000		M	
2	6.177	20504322	1334869	0.000		M	
总计		20394619	1335399				

peak number
retention time
area
height

Supplementary Figure 18. HPLC spectrum of (*3aS,6R,7aR,Z*)-3ag

4-((*Z*)-1-((*3aS,6R,7aR*)-7a-hydroxy-6-methyl-3-oxohexahydro-3a,6-methanoinden-5(4*H*)-ylidene)ethyl)benzonitrile (**3ah**)

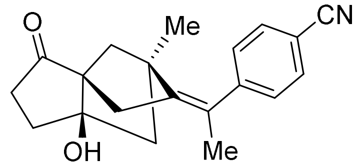

Chemical Formula: $C_{20}H_{21}NO_2$
Exact Mass: 307.1572

3ah was prepared according to general procedure using **1a** (0.1 mmol, 20.4 mg) and **2h** and was purified by silica gel column chromatography (petroleum ether/ethyl acetate = 20/1~2/1) to obtain **3ah** (21.2 mg, 69% yield). ^{1}H NMR (400 MHz, $CDCl_3$) δ 7.57 (d, *J* = 8.0 Hz, 2H), 7.23 (d, *J* = 8.0 Hz, 2H), 2.73-2.49 (m, 4H), 2.30-2.22 (m, 1H), 2.19-2.08 (m, 1H), 2.05-2.00 (m, 1H), 1.98-1.85 (bs, 1H), 1.91 (s, 3H), 1.81-1.76 (m, 1H), 1.71-1.68 (m, 1H), 1.64-1.59 (m, 1H), 0.56 (s, 3H);

^{13}C NMR (101 MHz, $CDCl_3$) δ 217.1, 149.3, 140.7, 131.6, 129.3, 126.9, 118.9, 110.1, 86.4, 64.2, 55.1, 54.0, 52.7, 38.1, 36.0, 33.6, 22.8, 20.5;

HRMS: (ESI) calcd for $C_{20}H_{22}NO_2^+$[M+H]$^+$ 308.1645; found 308.1643.

The enantiomeric purity was established by HPLC analysis using a chiral column: AD-H column, 30 °C, *n*-Hexane/*i*-Propanol = 85/15 as eluent, 254 nm, 1 mL/min. tR = 8.1 min (minor), 10.9 min (major).

Optical Rotation: $[\alpha]_D^{25}$ -52.5 (c 0.7, ^{i}PrOH) for 99% ee.

Absolute stereochemistry was determined through analogy with **3aa**.

<色谱图>

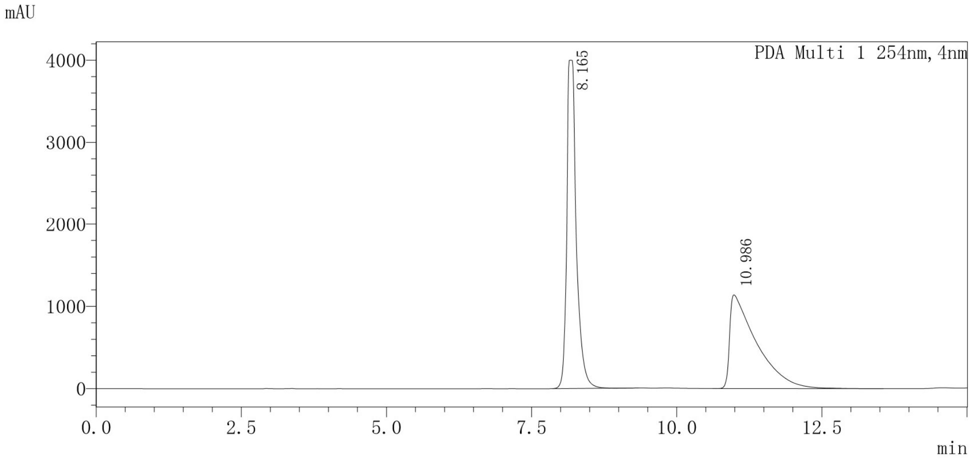

<峰表>

PDA Ch1 254nm

峰号	保留时间	面积	高度	浓度	浓度单位	标记	化合物名
1	8.165	45573883	3997409	0.000		M	
2	10.986	36711012	1138400	0.000		M	
总计		82284895	5135809				

peak number　area　height

retention time

Supplementary Figure 19. HPLC spectrum of 3ah

<色谱图>

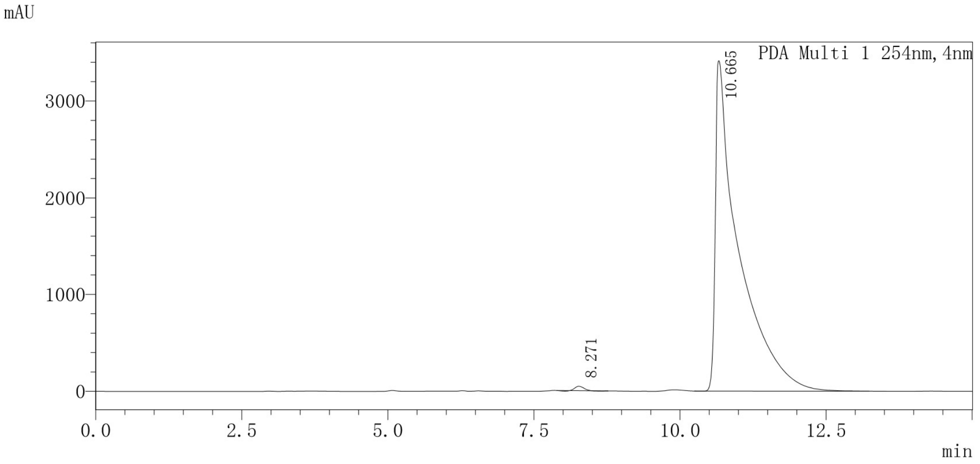

<峰表>

PDA Ch1 254nm

峰号	保留时间	面积	高度	浓度	浓度单位	标记	化合物名
1	8.271	428026	45839	0.000		M	
2	10.665	95222282	3414286	0.000		M	
总计		95650308	3460125				

peak number　area　height

retention time

Supplementary Figure 20. HPLC spectrum of (*3aS,6R,7aR,Z*)-3ah

($3aS,6R,7aR,Z$)-7a-hydroxy-6-methyl-5-(1-(4-(methylsulfonyl)phenyl)ethylidene) hexahydro-3a,6-methanoinden-3(2*H*)-one (**3ai**)

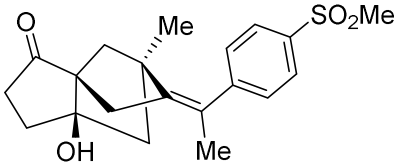

3ai was prepared according to general procedure using **1a** (0.1 mmol, 20.4 mg) and **2i** and was purified by silica gel column chromatography (petroleum ether/ethyl acetate = 20/1~2/1) to obtain **3ai** (28.8 mg, 80% yield). ^{1}H NMR (400 MHz, $CDCl_3$) δ 7.83 (d, J = 8.1 Hz, 2H), 7.32 (d, J = 8.1 Hz, 2H), 3.05 (s, 3H), 2.68 (s, 2H), 2.66-2.47 (m, 2H), 2.30-2.22 (m, 1H), 2.18-2.07 (m, 2H), 2.01 (d, J = 12.7 Hz, 1H), 1.91 (s, 3H), 1.79 (dd, J = 12.7, 3.5 Hz, 1H), 1.69 (d, J = 9.7 Hz, 1H), 1.60 (dd, J = 9.8, 3.5 Hz, 1H), 0.54 (s, 3H);

^{13}C NMR (101 MHz, $CDCl_3$) δ 217.3, 150.3, 140.9, 138.3, 129.5, 126.9, 126.6, 86.3, 64.2, 55.0, 54.0, 52.7, 44.5, 38.1, 36.0, 33.6, 22.9, 20.6;

HRMS: (ESI) calcd for $C_{20}H_{25}O_4S^+$[M+H]$^+$ 361.1468; found 361.1467.

The enantiomeric purity was established by HPLC analysis using a chiral column: OJ-H column, 30 °C, *n*-Hexane/*i*-Propanol = 60/40 as eluent, 254 nm, 1 mL/min. tR = 8.5 min (minor), 10.8 min (major).

Optical Rotation: $[\alpha]_D^{25}$ -39.4 (c 1.0, ^{i}PrOH) for 98% ee.

Absolute stereochemistry was determined through analogy with **3aa**.

<色谱图>

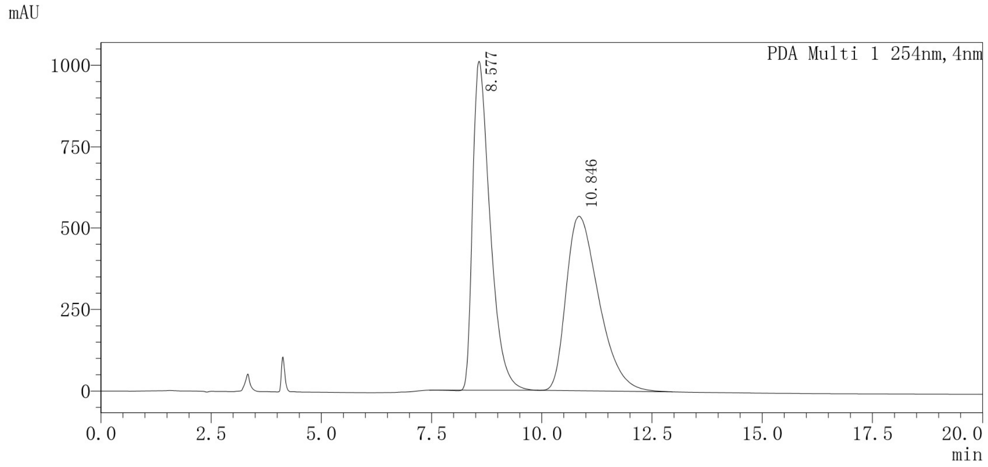

<峰表>

PDA Ch1 254nm

峰号	保留时间	面积	高度	浓度	浓度单位	标记	化合物名
1	8.577	27868595	1009921	0.000		M	
2	10.846	27677789	536469	0.000		M	
总计		55546384	1546390				

peak number
area
height
retention time

Supplementary Figure 21. HPLC spectrum of 3ai

<色谱图>

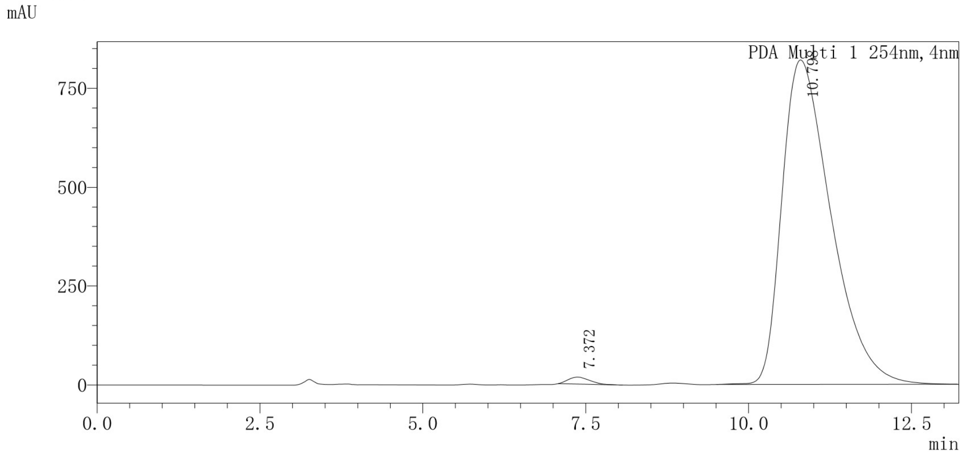

<峰表>

PDA Ch1 254nm

峰号	保留时间	面积	高度	浓度	浓度单位	标记	化合物名
1	7.372	396083	17462	0.000		M	
2	10.798	42922601	819887	0.000		M	
总计		43318683	837349				

peak number
area
height
retention time

Supplementary Figure 22. HPLC spectrum of (*3aS,6R,7aR,Z*)-3ai

(*3aS,6R,7aR,Z*)-7a-hydroxy-6-methyl-5-(1-(4-(trifluoromethoxy)phenyl)ethylidene) hexahydro-3a,6-methanoinden-3(2*H*)-one (**3aj**)

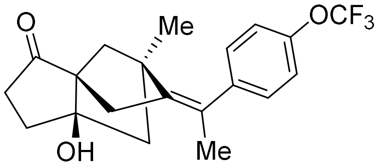

Chemical Formula: $C_{20}H_{21}F_3O_3$
Exact Mass: 366.1443

3aj was prepared according to general procedure using **1a** (0.1 mmol, 20.4 mg) and **2j** and was purified by silica gel column chromatography (petroleum ether/ethyl acetate = 20/1~2/1) to obtain **3aj** (24.5 mg, 68% yield). ^{1}H NMR (400 MHz, $CDCl_3$) δ 7.12 (bs, 4H), 2.73-2.49 (m, 4H), 2.31-2.22 (m, 1H), 2.17-2.07 (m, 1H), 2.04-1.99 (m, 1H), 1.90 (s, 3H), 1.86 (s, 1H), 1.81-1.76 (m, 1H), 1.71-1.67 (m, 1H), 1.64-1.59 (m, 1H), 0.57 (s, 3H);

^{13}C NMR (101 MHz, $CDCl_3$) δ 217.4, 147.7, 142.8, 139.5, 123.0 (q, *J* = 258 Hz), 129.7, 127.4, 120.2, 86.6, 64.3, 55.3, 54.0, 52.8, 38.1, 35.9, 33.5, 23.2, 20.4;

^{19}F NMR (376 MHz, $CDCl_3$) δ -57.8;

HRMS: (ESI) calcd for $C_{20}H_{22}F_3O_3^+$[M+H]$^+$ 367.1516; found 367.1516.

The enantiomeric purity was established by HPLC analysis using a chiral column: AD-H column, 30 °C, *n*-Hexane/*i*-Propanol = 85/15 as eluent, 254 nm, 1 mL/min. tR = 4.2 min (minor), 4.6 min (major).

Optical Rotation: $[\alpha]_D^{25}$ -88.5 (c 0.8, ^{i}PrOH) for 98% ee.

Absolute stereochemistry was determined through analogy with **3aa**.

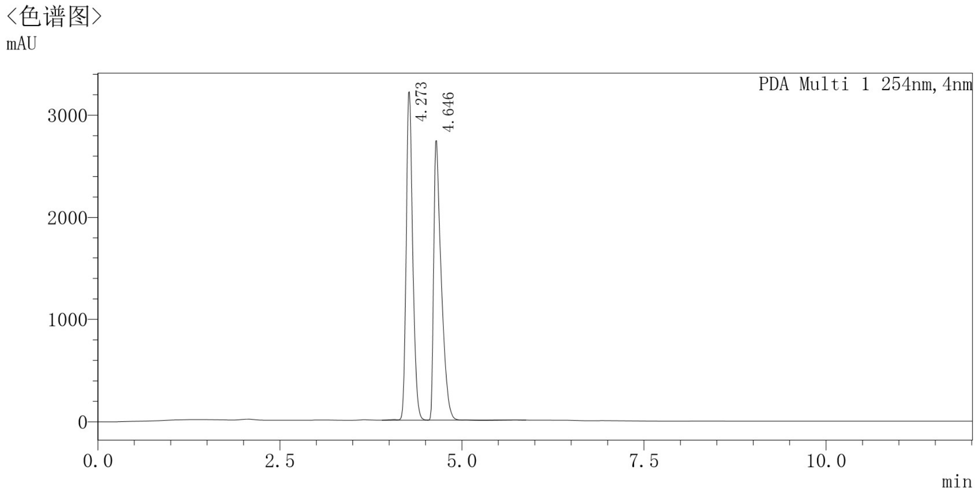

<峰表>

PDA Ch1 254nm

峰号	保留时间	面积	高度	浓度	浓度单位	标记	化合物名
1	4.273	20090746	3214201	0.000		M	
2	4.646	19628405	2731756	0.000		V M	
总计		39719151	5945957				

peak number　retention time　area　height

Supplementary Figure 23. HPLC spectrum of 3aj

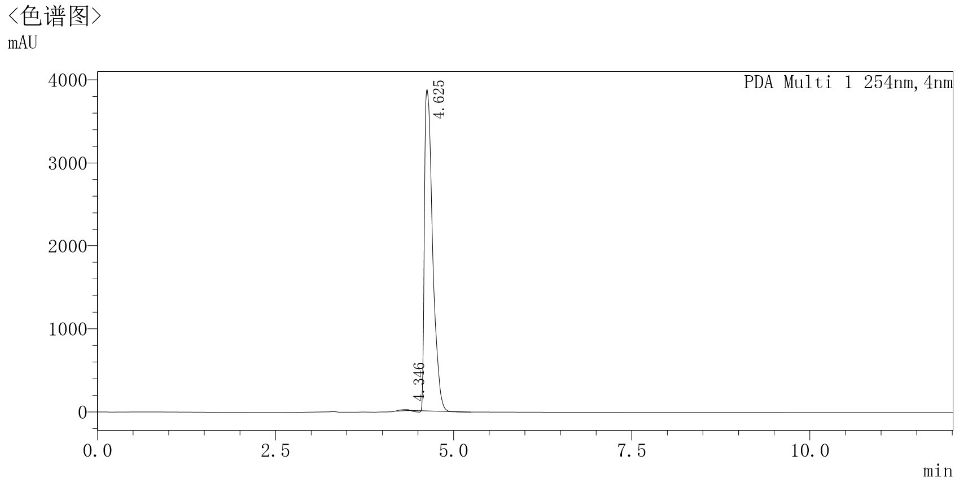

<峰表>

PDA Ch1 254nm

峰号	保留时间	面积	高度	浓度	浓度单位	标记	化合物名
1	4.346	122132	12929	0.000		M	
2	4.625	30744253	3869494	0.000		M	
总计		30866385	3882423				

peak number　retention time　area　height

Supplementary Figure 24. HPLC spectrum of (*3aS,6R,7aR,Z*)-3aj

(*3aS,6R,7aR,Z*)-7a-hydroxy-6-methyl-5-(1-(4-nitrophenyl)ethylidene)hexahydro-3a,6-methanoinden-3(2*H*)-one (**3ak**)

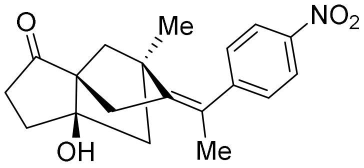

Chemical Formula: $C_{19}H_{21}NO_4$
Exact Mass: 327.1471

3ak was prepared according to general procedure using **1a** (0.1 mmol, 20.4 mg) and **2k** and was purified by silica gel column chromatography (petroleum ether/ethyl acetate = 10/1~1/1) to obtain **3ak** (25.8 mg, 79% yield). ^{1}H NMR (400 MHz, $CDCl_3$) δ 8.15 (d, *J* = 8.9 Hz, 2H), 7.29 (d, *J* = 8.3 Hz, 2H), 2.73-2.67 (m, 2H), 2.67-2.48 (m, 2H), 2.27 (ddd, *J* = 14.0, 8.9, 1.9 Hz, 1H), 2.21-2.09 (m, 1H), 2.04 (d, *J* = 12.7 Hz, 1H), 1.93 (s, 3H), 1.85 (bs, 1H), 1.81 (dd, *J* = 12.7, 3.6 Hz, 1H), 1.71 (dd, *J* = 10.1, 2.1 Hz, 1H), 1.63 (dd, *J* = 9.8, 3.6 Hz, 1H), 0.58 (s, 3H);

^{13}C NMR (101 MHz, $CDCl_3$) δ 217.0, 151.4, 146.4, 141.2, 129.4, 126.6, 123.1, 86.4, 64.2, 55.1, 54.1, 52.7, 38.1, 36.0, 33.7, 22.8, 20.6;

HRMS: (ESI) calcd for $C_{19}H_{22}NO_4^+$[M+H]$^+$ 328.1543; found 328.1544.

The enantiomeric purity was established by HPLC analysis using a chiral column: AD-H column, 30 °C, *n*-Hexane/*i*-Propanol = 80/20 as eluent, 254 nm, 1 mL/min. tR = 5.8 min (minor), 6.6 min (major).

Optical Rotation: $[\alpha]_D^{25}$ -106.4 (c 0.9, ^{i}PrOH) for 99% ee.

Absolute stereochemistry was determined through analogy with **3aa**.

〈色谱图〉

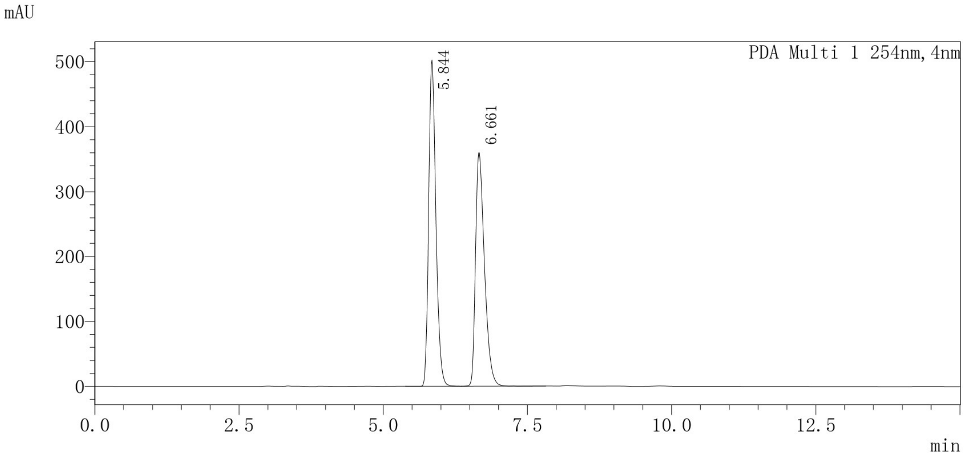

〈峰表〉

PDA Ch1 254nm

峰号	保留时间	面积	高度	浓度	浓度单位	标记	化合物名
1	5.844	4352175	502404	0.000		M	
2	6.661	3863500	359765	0.000		M	
总计		8215675	862168				

peak number, retention time, area, height

Supplementary Figure 25. HPLC spectrum of 3ak

〈色谱图〉

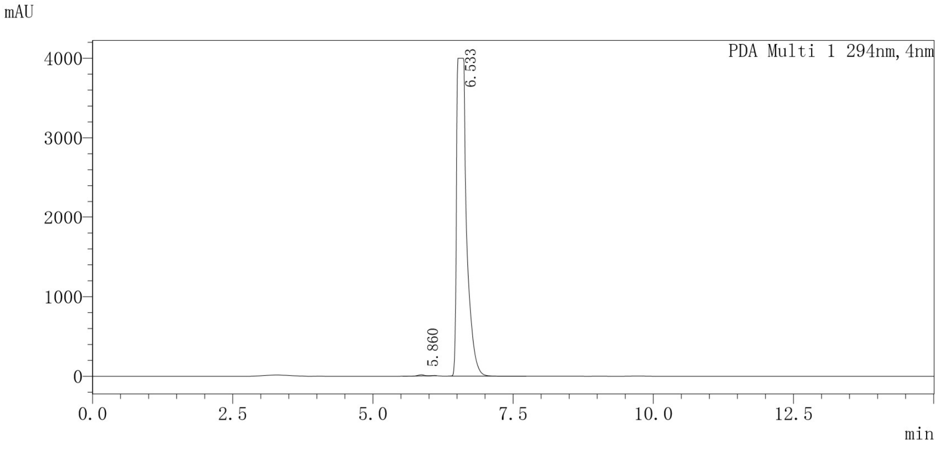

〈峰表〉

PDA Ch1 294nm

峰号	保留时间	面积	高度	浓度	浓度单位	标记	化合物名
1	5.860	90566	14574	0.000		M	
2	6.533	49488676	3997769	0.000		M	
总计		49579241	4012343				

peak number, retention time, area, height

Supplementary Figure 26. HPLC spectrum of (*3aS,6R,7aR,Z*)-3ak

4-((*Z*)-1-((*3aS,6R,7aR*)-7a-hydroxy-6-methyl-3-oxohexahydro-3a,6-methanoinden-5(4*H*)-ylidene)ethyl)benzaldehyde (**3al**)

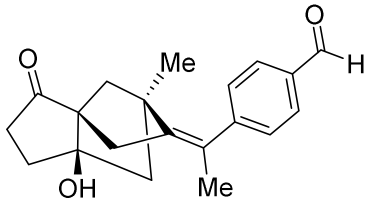

Chemical Formula: $C_{20}H_{22}O_3$
Exact Mass: 310.1569

3al was prepared according to general procedure using **1a** (0.1 mmol, 20.4 mg) and **2l** and was purified by silica gel column chromatography (petroleum ether/ethyl acetate = 20/1~2/1) to obtain **3al** (18.6 mg, 60% yield). ^{1}H NMR (400 MHz, $CDCl_3$) δ 9.98 (s, 1H), 7.79 (d, *J* = 7.8 Hz, 2H), 7.29 (d, *J* = 7.8 Hz, 2H), 2.72-2.49 (m, 4H), 2.31-2.22 (m, 1H), 2.18-2.10 (m, 1H), 2.09-2.04 (m, 1H), 2.01 (s, 1H), 1.93 (s, 3H), 1.83 (dd, *J* = 12.7, 3.5 Hz, 1H), 1.70 (dd, *J* = 9.8, 3.2 Hz, 1H), 1.62 (dd, *J* = 9.8, 3.6 Hz, 1H), 0.57 (s, 3H);

^{13}C NMR (101 MHz, $CDCl_3$) δ 217.3, 192.0, 151.0, 140.2, 134.6, 129.3, 129.2, 127.5, 86.4, 64.3, 55.2, 54.1, 52.8, 38.1, 36.0, 33.6, 22.9, 20.4;

HRMS: (ESI) calcd for $C_{20}H_{23}O_3^+$[M+H]$^+$ 311.1642; found 311.1642.

The enantiomeric purity was established by HPLC analysis using a chiral column: AD-H column, 30 °C, *n*-Hexane/*i*-Propanol = 85/15 as eluent, 254 nm, 1 mL/min. tR = 8.6 min (minor), 11.0 min (major).

Optical Rotation: $[\alpha]_D^{25}$ -55.4 (c 0.6, ^{i}PrOH) for 98% ee.

Absolute stereochemistry was determined through analogy with **3aa**.

〈色谱图〉

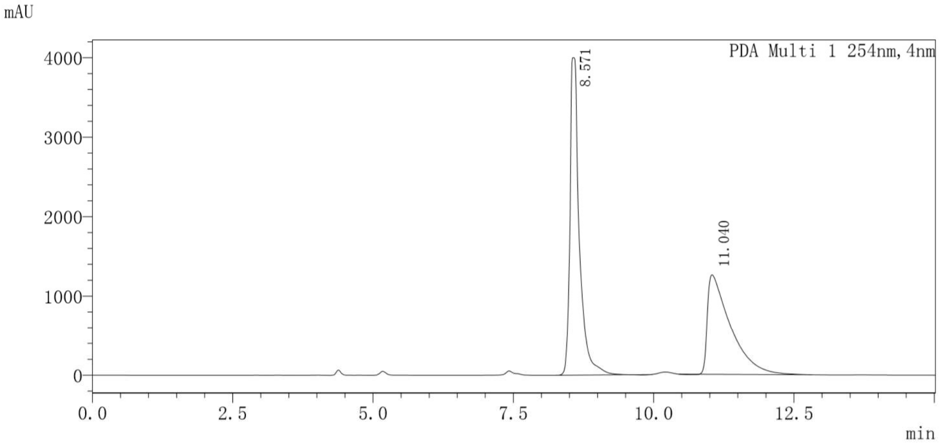

〈峰表〉

PDA Ch1 254nm

峰号	保留时间	面积	高度	浓度	浓度单位	标记	化合物名
1	8.571	46571837	3999000	0.000		M	
2	11.040	36008651	1258042	0.000		M	
总计		82580488	5257042				

peak number　retention time　area　height

Supplementary Figure 27. HPLC spectrum of 3al

〈色谱图〉

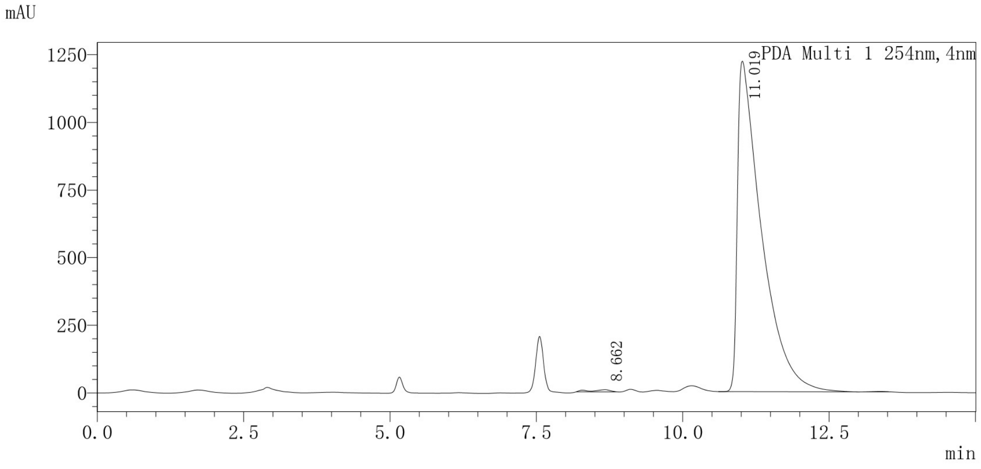

〈峰表〉

PDA Ch1 254nm

峰号	保留时间	面积	高度	浓度	浓度单位	标记	化合物名
1	8.662	192598	8338	0.000		M	
2	11.019	34956013	1221678	0.000		M	
总计		35148612	1230016				

peak number　retention time　area　height

Supplementary Figure 28. HPLC spectrum of (*3aS,6R,7aR,Z*)-3al

(*3aS,6R,7aR,Z*)-5-(1-(4-acetylphenyl)ethylidene)-7a-hydroxy-6-methylhexahydro-3a,6-methanoinden-3(2*H*)-one (**3am**)

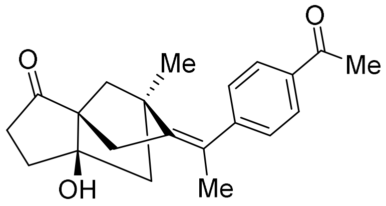

Chemical Formula: $C_{21}H_{24}O_3$
Exact Mass: 324.1725

3am was prepared according to general procedure using **1a** (0.1 mmol, 20.4 mg) and **2m** and was purified by silica gel column chromatography (petroleum ether/ethyl acetate = 10/1~1/1) to obtain **3am** (20.1 mg, 62% yield). ^{1}H NMR (400 MHz, $CDCl_3$) δ 7.87 (d, *J* = 8.4 Hz, 2H), 7.21 (d, *J* = 8.4 Hz, 2H), 2.76-2.60 (m, 3H), 2.59 (s, 3H), 2.57-2.49 (m, 1H), 2.31-2.23 (m, 1H), 2.18-2.09 (m, 1H), 2.08-2.03 (m, 1H), 2.01 (s, 1H), 1.92 (s, 3H), 1.83 (dd, *J* = 12.8, 3.2 Hz, 1H), 1.69 (dd, *J* = 9.8, 3.2 Hz, 1H), 1.62 (dd, *J* = 9.8, 3.2 Hz, 1H), 0.57 (s, 3H);

^{13}C NMR (101 MHz, $CDCl_3$) δ 217.4, 197.9, 149.5, 139.9, 135.2, 128.7, 127.9, 127.6, 86.5, 64.3, 55.3, 54.1, 52.8, 38.1, 35.9, 33.6, 26.5, 22.9, 20.4;

HRMS: (ESI) calcd for $C_{21}H_{25}O_3^+$[M+H]$^+$ 325.1798; found 325.1798.

The enantiomeric purity was established by HPLC analysis using a chiral column: OD-H column, 30 °C, *n*-Hexane/*i*-Propanol = 90/10 as eluent, 254 nm, 1 mL/min. tR = 12.8 min (major), 14.1 min (minor).

Optical Rotation: $[\alpha]_D^{25}$ -72.9 (c 0.7, ^{i}PrOH) for 97% ee.

Absolute stereochemistry was determined through analogy with **3aa**.

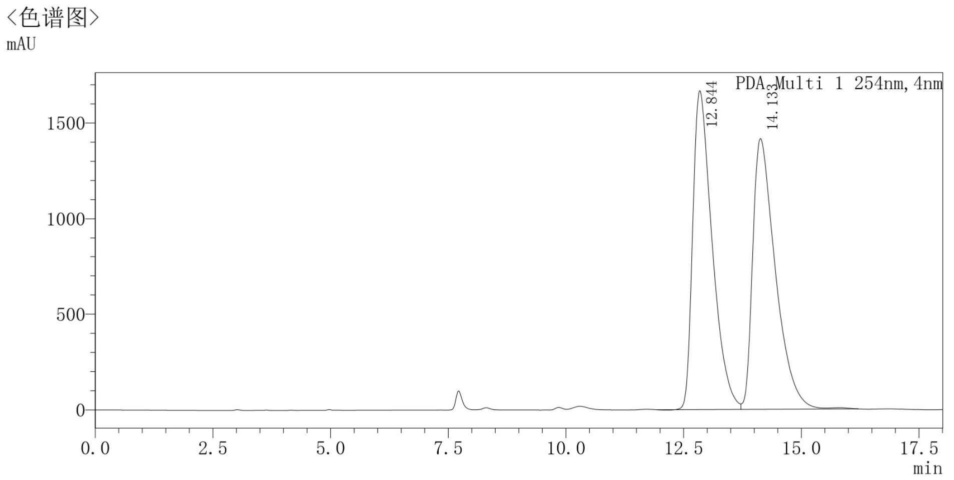

〈峰表〉

PDA Ch1 254nm

峰号	保留时间	面积	高度	浓度	浓度单位	标记	化合物名
1	12.844	47991558	1667063	0.000		M	
2	14.133	47632228	1415901	0.000		V M	
总计		95623786	3082964				

peak number | retention time | area | height

Supplementary Figure 29. HPLC spectrum of 3am

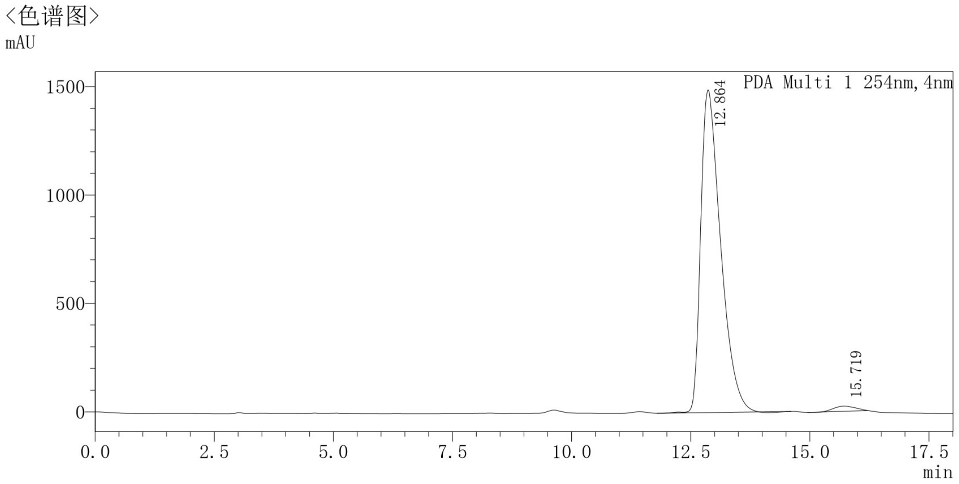

〈峰表〉

PDA Ch1 254nm

峰号	保留时间	面积	高度	浓度	浓度单位	标记	化合物名
1	12.864	43199871	1488265	0.000		M	
2	15.719	738991	23413	0.000		M	
总计		43938862	1511678				

peak number | retention time | area | height

Supplementary Figure 30. HPLC spectrum of (*3aS,6R,7aR,Z*)-3am

4-((*Z*)-1-((*3aS,6R,7aR*)-7a-hydroxy-6-methyl-3-oxohexahydro-3a,6-methanoinden-5(4*H*)-ylidene)ethyl)-*N,N*-dimethylbenzamide (**3an**)

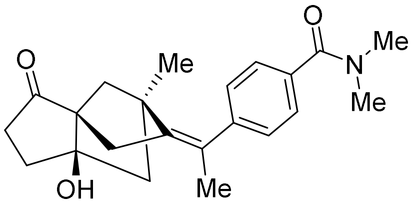

Chemical Formula: $C_{22}H_{27}NO_3$
Exact Mass: 353.1991

3an was prepared according to general procedure using **1a** (0.1 mmol, 20.4 mg) and **2n** and was purified by silica gel column chromatography (petroleum ether/ethyl acetate = 10/1~1/2) to obtain **3an** (26.1 mg, 74% yield). ^{1}H NMR (400 MHz, $CDCl_3$) δ 7.32 (d, *J* = 7.8 Hz, 2H), 7.13 (d, *J* = 7.8 Hz, 2H), 3.09 (s, 3H), 2.98 (s, 3H), 2.70-2.46 (m, 4H), 2.37 (bs, 1H), 2.28-2.21 (m, 1H), 2.14-2.04 (m, 1H), 2.00-1.94 (m, 1H), 1.88 (s, 3H), 1.83-1.77 (m, 1H), 1.69-1.56 (m, 2H), 0.57 (s, 3H);

^{13}C NMR (101 MHz, $CDCl_3$) δ 217.6, 171.6, 145.7, 139.4, 134.0, 128.4, 127.7, 126.6, 86.4, 64.3, 55.2, 54.0, 52.8, 39.7, 38.1, 35.9, 35.4, 33.6, 23.0, 20.5;

HRMS: (ESI) calcd for $C_{22}H_{28}NO_3^+$[M+H]$^+$ 354.2064; found 354.2065.

The enantiomeric purity was established by HPLC analysis using a chiral column: AD-H column, 30 °C, *n*-Hexane/*i*-Propanol = 80/20 as eluent, 254 nm, 1 mL/min. tR = 6.7 min (major), 7.1 min (minor).

Optical Rotation: $[\alpha]_D^{25}$ -61.8 (c 0.9, ^{i}PrOH) for 99% ee.

Absolute stereochemistry was determined through analogy with **3aa**.

<色谱图>

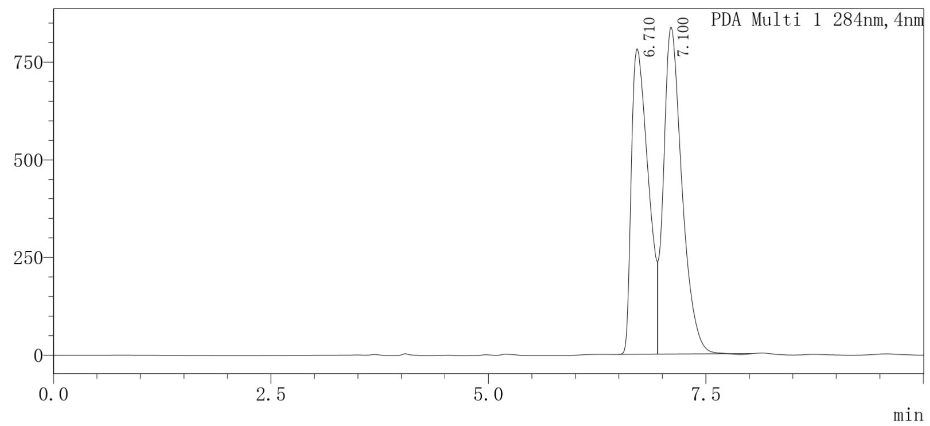

<峰表>

PDA Ch1 284nm

峰号	保留时间	面积	高度	浓度	浓度单位	标记	化合物名
1	6.710	10659530	781908	0.000		M	
2	7.100	12296489	836289	0.000		V M	
总计		22956019	1618197				

peak number

retention time

area

height

Supplementary Figure 31. HPLC spectrum of 3an

<色谱图>

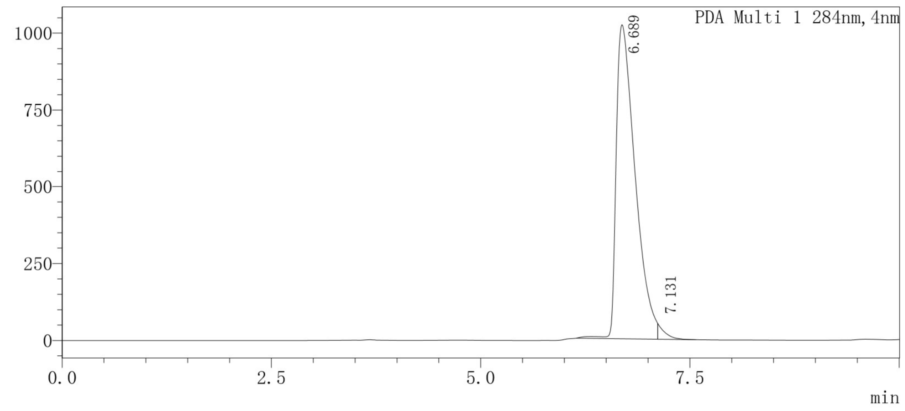

<峰表>

PDA Ch1 284nm

峰号	保留时间	面积	高度	浓度	浓度单位	标记	化合物名
1	6.689	16048843	1021956	0.000		M	
2	7.131	269356	46584	0.000		V M	
总计		16318199	1068541				

peak number

retention time

area

height

Supplementary Figure 32. HPLC spectrum of (*3aS,6R,7aR,Z*)-3an

Ethyl 4-((*Z*)-1-((*3aS,6R,7aR*)-7*a*-hydroxy-6-methyl-3-oxohexahydro-3a,6-methanoinden-5(4*H*)-ylidene)ethyl)benzoate (**3ao**)

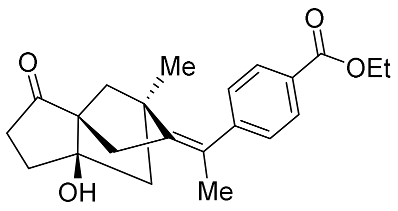

Chemical Formula: $C_{22}H_{26}O_4$
Exact Mass: 354.1831

3ao was prepared according to general procedure using **1a** (0.1 mmol, 20.4 mg) and **2o** and was purified by silica gel column chromatography (petroleum ether/ethyl acetate = 10/1~1/1) to obtain **3ao** (23.7 mg, 67% yield). ^{1}H NMR (400 MHz, $CDCl_3$) δ 7.95 (d, *J* = 7.8 Hz, 2H), 7.18 (d, *J* = 7.8 Hz, 2H), 4.36 (q, *J* = 8.0 Hz, 2H), 2.74-2.48 (m, 4H), 2.31-2.22 (m, 1H), 2.17-2.08 (m, 1H), 2.07 (bs, 1H), 2.03-1.99 (m, 1H), 1.91 (s, 3H), 1.82 (dd, *J* = 12.7, 3.5 Hz, 1H), 1.68 (dd, *J* = 9.8, 3.1 Hz, 1H), 1.61 (dd, *J* = 9.8, 3.4 Hz, 1H), 1.38 (t, *J* = 8.0 Hz, 3H), 0.56 (s, 3H);

^{13}C NMR (101 MHz, $CDCl_3$) δ 217.4, 166.6, 149.1, 139.6, 129.1, 128.5, 128.4, 127.7, 86.5, 64.3, 60.9, 55.3, 54.0, 52.8, 38.1, 35.9, 33.6, 22.9, 20.4, 14.3;

HRMS: (ESI) calcd for $C_{22}H_{27}O_4^+$[M+H]$^+$ 355.1904; found 355.1905.

The enantiomeric purity was established by HPLC analysis using a chiral column: AD-H column, 30 °C, *n*-Hexane/*i*-Propanol = 85/15 as eluent, 254 nm, 1 mL/min. tR = 7.4 min (minor), 7.8 min (major).

Optical Rotation: $[\alpha]_D^{25}$ -50.4 (c 0.8, ^{i}PrOH) for 99% ee.

Absolute stereochemistry was determined through analogy with **3aa**.

〈色谱图〉

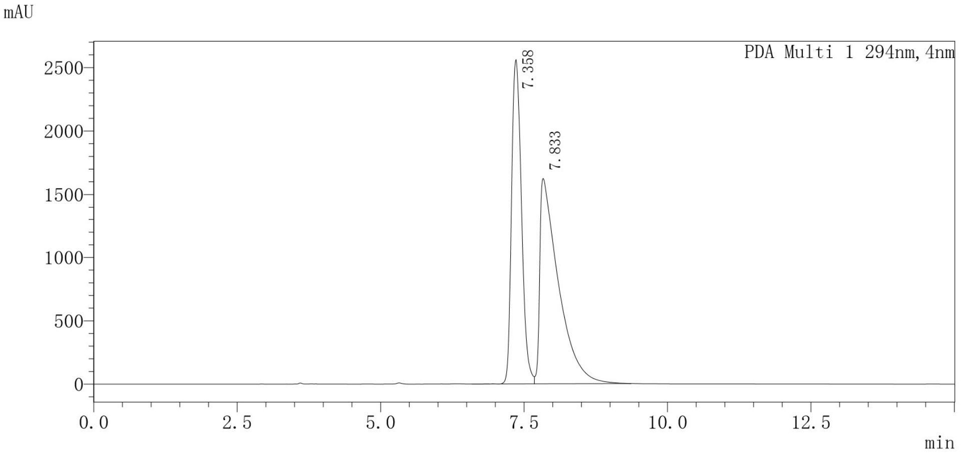

〈峰表〉

PDA Ch1 294nm

峰号	保留时间	面积	高度	浓度	浓度单位	标记	化合物名
1	7.358	30866048	2561520	0.000		M	
2	7.833	36750372	1623997	0.000		V M	
总计		67616419	4185517				

peak number　area　height

retention time

Supplementary Figure 33. HPLC spectrum of 3ao

〈色谱图〉

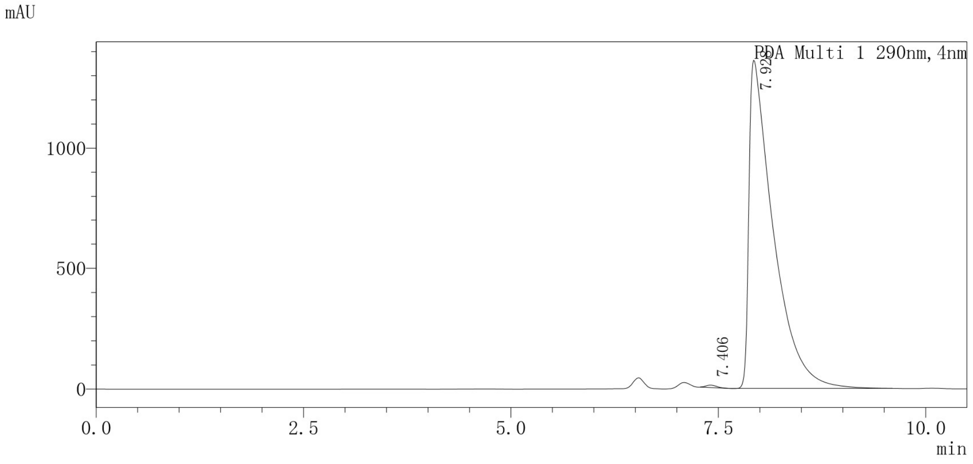

〈峰表〉

PDA Ch1 290nm

峰号	保留时间	面积	高度	浓度	浓度单位	标记	化合物名
1	7.406	91216	10160	0.000		M	
2	7.928	27743403	1361124	0.000		M	
总计		27834619	1371283				

peak number　area　height

retention time

Supplementary Figure 34. HPLC spectrum of (*3aS,6R,7aR,Z*)-3ao

(*3aS,6R,7aR,Z*)-7a-hydroxy-5-(1-(3-iodophenyl)ethylidene)-6-methylhexahydro-3a,6-methanoinden-3(2*H*)-one (**3ap**)

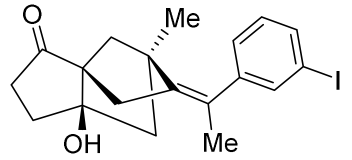

Chemical Formula: $C_{19}H_{21}IO_2$
Exact Mass: 408.0586

3ap was prepared according to general procedure using **1a** (0.1 mmol, 20.4 mg) and **2p** and was purified by silica gel column chromatography (petroleum ether/ethyl acetate = 10/1~2/1) to obtain **3ap** (30.2 mg, 74% yield). ^{1}H NMR (400 MHz, $CDCl_3$) δ 7.58-7.52 (m, 1H), 7.49 (bs, 1H), 7.08 (d, *J* = 7.6 Hz, 1H), 7.01 (t, *J* = 7.6 Hz, 1H), 2.71-2.49 (m, 4H), 2.31-2.21 (m, 1H), 2.17-2.08 (m, 1H), 2.03 (d, *J* = 12.8 Hz, 1H), 1.94-1.82 (m, 1H), 1.89 (s, 3H), 1.79 (dd, *J* = 12.8, 3.6 Hz, 1H), 1.69 (dd, *J* = 9.8, 3.6 Hz, 1H), 1.61 (dd, *J* = 9.8, 3.6 Hz, 1H), 0.61 (s, 3H);

^{13}C NMR (101 MHz, $CDCl_3$) δ 217.4, 146.3, 139.6, 137.3, 135.3, 129.5, 127.7, 127.2, 123.0, 86.5, 64.3, 55.3, 54.1, 52.8, 38.2, 35.9, 33.5, 23.1, 20.5;

HRMS: (ESI) calcd for $C_{19}H_{22}IO_2^+$[M+H]$^+$ 409.0659; found 409.0659.

The enantiomeric purity was established by HPLC analysis using a chiral column: AD-H column, 30 °C, *n*-Hexane/*i*-Propanol = 85/15 as eluent, 254 nm, 1 mL/min. tR = 5.1 min (minor), 6.7 min (major).

Optical Rotation: $[\alpha]_D^{25}$ -54.8 (c 1.0, ^{i}PrOH) for 99% ee.

Absolute stereochemistry was determined through analogy with **3aa**.

<色谱图>

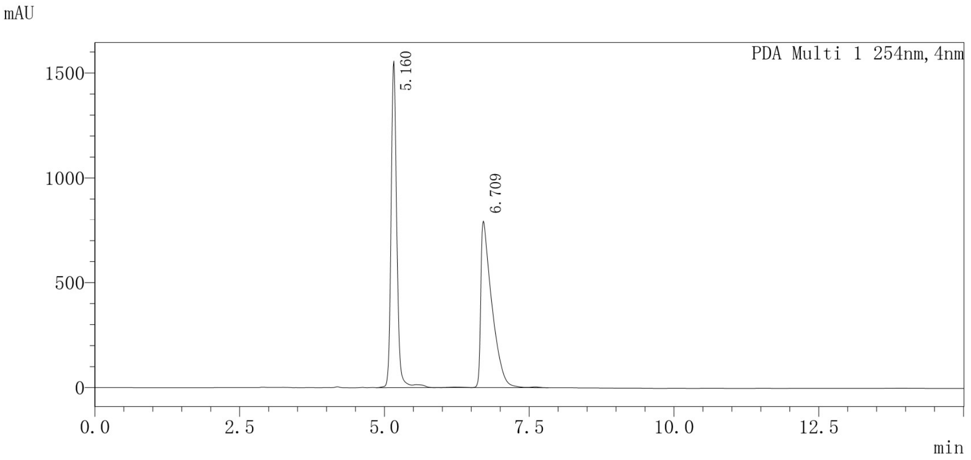

<峰表>

PDA Ch1 254nm

峰号	保留时间	面积	高度	浓度	浓度单位	标记	化合物名
1	5.160	10656002	1557942	0.000		M	
2	6.709	10570534	793341	0.000		M	
总计		21226536	2351282				

peak number　retention time　area　height

Supplementary Figure 35. HPLC spectrum of 3ap

<色谱图>

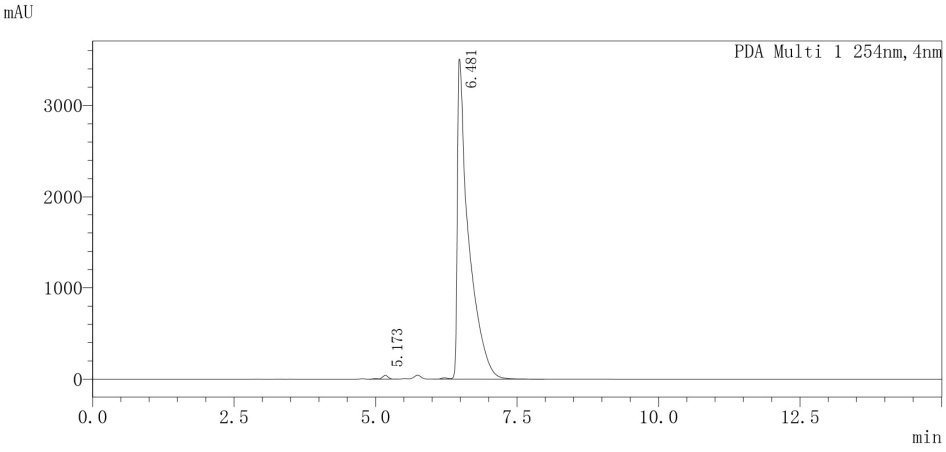

<峰表>

PDA Ch1 254nm

峰号	保留时间	面积	高度	浓度	浓度单位	标记	化合物名
1	5.173	319847	43030	0.000		M	
2	6.481	48854079	3506565	0.000		M	
总计		49173926	3549595				

peak number　retention time　area　height

Supplementary Figure 36. HPLC spectrum of (*3aS,6R,7aR,Z*)-3ap

(*3aS,6R,7aR,Z*)-5-(1-(3-fluoro-4-methylphenyl)ethylidene)-7a-hydroxy-6-methylhexahydro-3a,6-methanoinden-3(2*H*)-one (**3aq**)

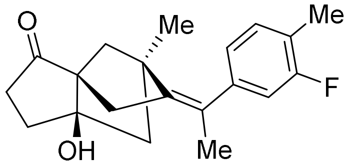

Chemical Formula: $C_{20}H_{23}FO_2$
Exact Mass: 314.1682

3aq was prepared according to general procedure using **1a** (0.1 mmol, 20.4 mg) and **2q** and was purified by silica gel column chromatography (petroleum ether/ethyl acetate = 20/1~2/1) to obtain **3aq** (25.1 mg, 78% yield). ^{1}H NMR (400 MHz, $CDCl_3$) δ 7.11-7.02 (m, 1H), 6.81-6.72 (m, 2H), 2.74-2.47 (m, 4H), 2.31-2.22 (m, 4H), 2.17-2.07 (m, 1H), 2.02 (d, *J* = 12.7 Hz, 1H), 1.90-1.83 (m, 4H), 1.80 (dd, *J* = 12.8, 3.5 Hz, 1H), 1.69 (dd, *J* = 9.8, 3.4 Hz, 1H), 1.61 (dd, *J* = 9.8, 3.5 Hz, 1H), 0.63 (s, 3H);

^{13}C NMR (101 MHz, $CDCl_3$) δ 217.5, 143.5 (d, *J* = 7.5 Hz), 139.0, 130.6, 127.6, 124.9, 124.0, 122.6 (d, *J* = 16.6 Hz), 115.1, 86.6, 64.3, 55.4, 54.0, 52.8, 38.2, 35.9, 33.5, 23.1, 20.3, 14.3;

^{19}F NMR (376 MHz, $CDCl_3$) δ -118.57;

HRMS: (ESI) calcd for $C_{20}H_{24}FO_2^+$[M+H]$^+$ 315.1755; found 315.1755.

The enantiomeric purity was established by HPLC analysis using a chiral column: AD-H column, 30 °C, *n*-Hexane/*i*-Propanol = 85/15 as eluent, 254 nm, 1 mL/min. tR = 4.4 min (minor), 4.9 min (major).

Optical Rotation: $[\alpha]_D^{25}$ -78.1 (c 0.8, ^{i}PrOH) for 99% ee.

Absolute stereochemistry was determined through analogy with **3aa**.

〈色谱图〉

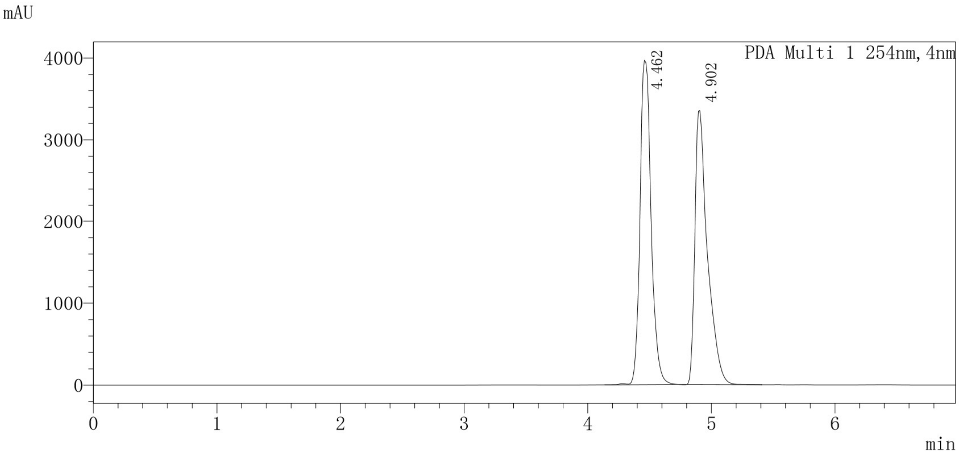

〈峰表〉

PDA Ch1 254nm

峰号	保留时间	面积	高度	浓度	浓度单位	标记	化合物名
1	4.462	24607760	3968914	0.000		M	
2	4.902	23638835	3350706	0.000		M	
总计		48246594	7319620				

peak number　area　height

retention time

Supplementary Figure 37. HPLC spectrum of 3aq

〈色谱图〉

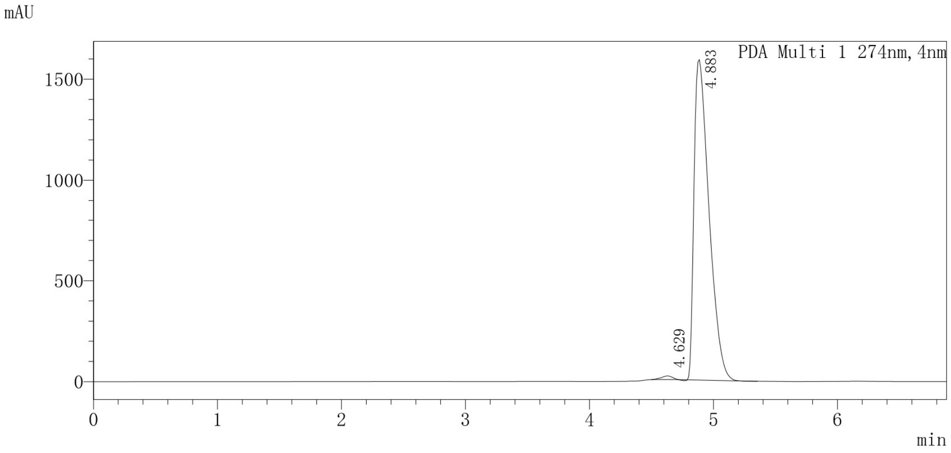

〈峰表〉

PDA Ch1 274nm

峰号	保留时间	面积	高度	浓度	浓度单位	标记	化合物名
1	4.629	113364	17960	0.000		M	
2	4.883	13330328	1589665	0.000		M	
总计		13443691	1607625				

peak number　area　height

retention time

Supplementary Figure 38. HPLC spectrum of (*3aS,6R,7aR,Z*)-3aq

(*3aS,6R,7aR,Z*)-5-(1-(2,4-difluorophenyl)ethylidene)-7a-hydroxy-6-methylhexahydro-3a,6-methanoinden-3(2*H*)-one (**3ar**)

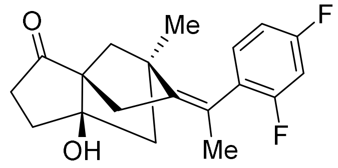

Chemical Formula: $C_{19}H_{20}F_2O_2$
Exact Mass: 318.1431

3ar was prepared according to general procedure using **1a** (0.1 mmol, 20.4 mg) and **2r** and was purified by silica gel column chromatography (petroleum ether/ethyl acetate = 20/1~2/1) to obtain **3ar** (26.1 mg, 82% yield). ^{1}H NMR (400 MHz, $CDCl_3$) δ 7.13-6.96 (m, 1H), 6.86-6.71 (m, 2H), 2.78-2.44 (m, 4H), 2.31-2.19 (m, 1H), 2.18-2.05 (m, 1H), 2.03-1.89 (m, 1H), 1.87 (d, *J* = 4.8 Hz, 3H), 1.85-1.78 (m, 1H), 1.76-1.57(m, 3H), 0.61 (d, *J* = 1.9 Hz, 3H);

^{13}C NMR (101 MHz, $CDCl_3$) δ 217.5, 217.1, 162.6 (dd, *J* = 54.1, 11.5 Hz), 161.0 (dd, *J* = 54.4, 11.5 Hz), 160.2 (dd, *J* = 93.6, 11.9 Hz), 158.6 (dd, *J* = 90.8, 11.8 Hz), 141.6, 141.4, 131.4 (d, *J* = 8.4 Hz), 131.3 (d, *J* = 8.6 Hz), 127.2 (dd, *J* = 17.9, 3.9 Hz), 126.8 (dd, *J* = 18.1, 3.7 Hz), 122.1, 119.8, 110.9 (dd, *J* = 21.0, 3.6 Hz), 110.4 (dd, *J* = 20.7, 3.3 Hz), 103.8 (t, *J* = 25.7 Hz), 103.4 (t, *J* = 25.7 Hz), 86.5, 86.3, 64.30, 64.26, 55.2, 54.1, 54.0, 53.42, 53.40, 52.6, 52.5, 38.2, 38.1, 35.9, 35.8, 33.5, 33.2, 22.9, 21.8, 20.1, 19.2;

^{19}F NMR (376 MHz, $CDCl_3$) δ -110.92 (dq, *J* = 172.5, 8.3 Hz), -112.26 (dp, *J* = 49.6, 7.5 Hz);

HRMS: (ESI) calcd for $C_{19}H_{21}F_2O_2^+$[M+H]$^+$ 319.1504; found 319.1508.

The enantiomeric purity was established by HPLC analysis using a chiral column: AD-H column, 30 °C, *n*-Hexane/*i*-Propanol = 85/15 as eluent, 254 nm, 1 mL/min. tR = 5.1 min (minor), 6.4 min (major).

Optical Rotation: $[\alpha]_D^{25}$ -60.1 (c 0.9, ^{i}PrOH) for 96% ee.

Absolute stereochemistry was determined through analogy with **3aa**.

<色谱图>

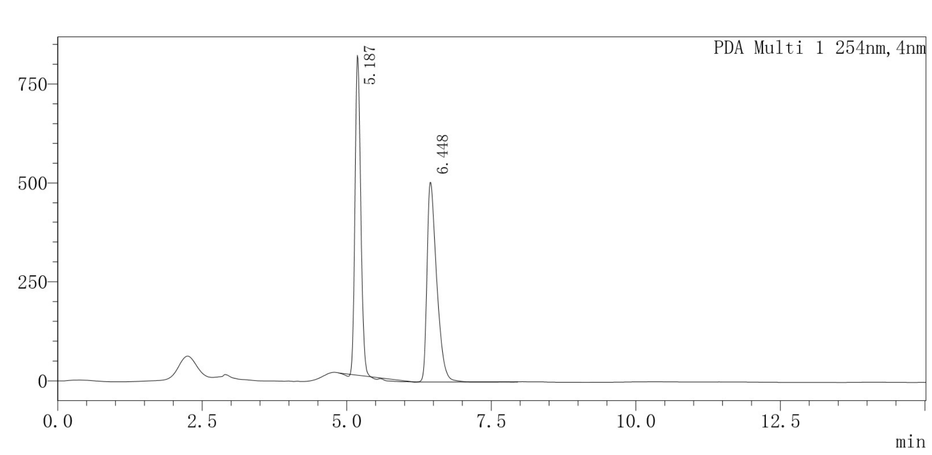

<峰表>

PDA Ch1 254nm

峰号	保留时间	面积	高度	浓度	浓度单位	标记	化合物名
1	5.187	5402478	808476	0.000		M	
2	6.448	5869014	504554	0.000		M	
总计		11271491	1313030				

peak number

retention time

area

height

Supplementary Figure 39. HPLC spectrum of 3ar

<色谱图>

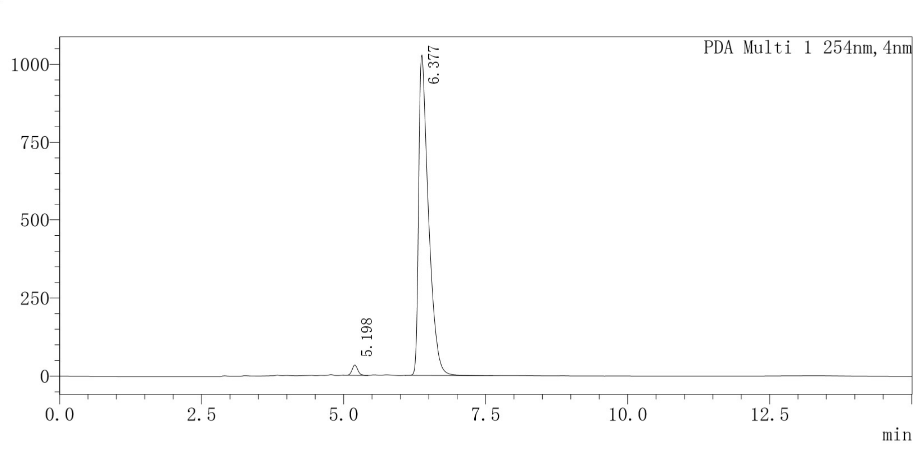

<峰表>

PDA Ch1 254nm

峰号	保留时间	面积	高度	浓度	浓度单位	标记	化合物名
1	5.198	223707	32653	0.000		M	
2	6.377	12379377	1027759	0.000		M	
总计		12603084	1060412				

peak number

retention time

area

height

Supplementary Figure 40. HPLC spectrum of (*3aS,6R,7aR,Z*)-3ar

(*3aS,6R,7aR,Z*)-7a-hydroxy-6-methyl-5-(1-(naphthalen-2-yl)ethylidene)hexahydro-3a,6-methanoinden-3(2*H*)-one (**3as**)

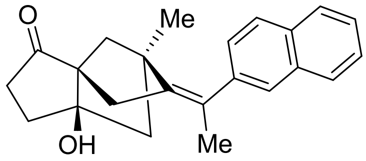

Chemical Formula: $C_{23}H_{24}O_2$
Exact Mass: 332.1776

3as was prepared according to general procedure using **1a** (0.1 mmol, 20.4 mg) and **2s** and was purified by silica gel column chromatography (petroleum ether/ethyl acetate = 20/1~2/1) to obtain **3as** (22.9 mg, 69% yield). ^{1}H NMR (400 MHz, $CDCl_3$) δ 7.84-7.80 (m, 1H), 7.80-7.74 (m, 2H), 7.55 (s, 1H), 7.50-7.41 (m, 2H), 7.28 (dd, *J* = 8.4, 1.5 Hz, 1H), 2.82-2.50 (m, 4H), 2.32-2.24 (m, 1H), 2.20-2.08 (m, 1H), 2.05 (d, *J* = 12.7 Hz, 1H), 2.01 (s, 3H), 1.92 (dd, *J* = 12.7, 3.2 Hz, 1H), 1.78 (s, 1H), 1.70 (dd, *J* = 9.7, 3.3 Hz, 1H), 1.65 (dd, *J* = 9.7, 3.3 Hz, 1H), 0.56 (s, 3H);

^{13}C NMR (101 MHz, $CDCl_3$) δ 217.4, 141.6, 139.0, 133.0, 132.1, 128.7, 127.7, 127.6, 127.3, 126.7, 126.0, 125.5, 86.7, 64.4, 55.6, 54.2, 52.9, 38.2, 35.9, 33.7, 23.3, 20.5;

HRMS: (ESI) calcd for $C_{23}H_{25}O_2^+$[M+H]$^+$ 333.1849; found 333.1844.

The enantiomeric purity was established by HPLC analysis using a chiral column: AD-H column, 30 °C, *n*-Hexane/*i*-Propanol = 90/10 as eluent, 254 nm, 1 mL/min. tR = 7.2 min (minor), 7.5 min (major).

Optical Rotation: $[\alpha]_D^{25}$ -32.3 (c 0.8, ^{i}PrOH) for 94% ee.

Absolute stereochemistry was determined through analogy with **3aa**.

〈色谱图〉

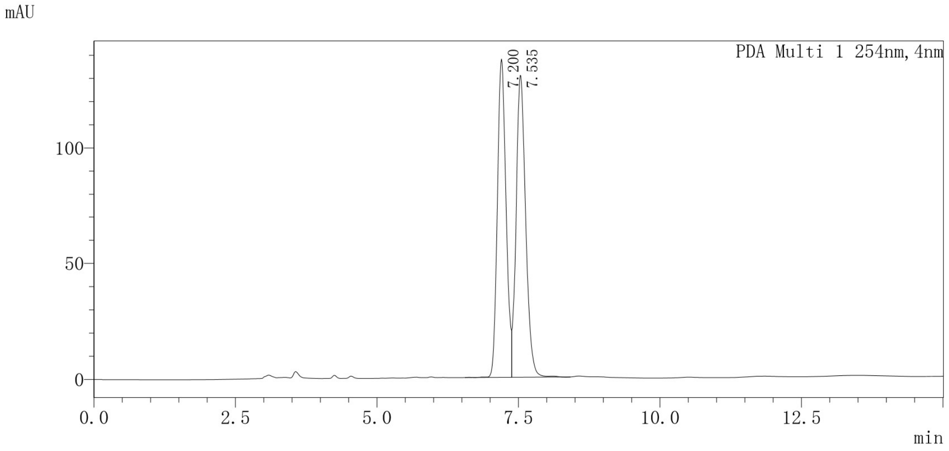

〈峰表〉

PDA Ch1 254nm

峰号	保留时间	面积	高度	浓度	浓度单位	标记	化合物名
1	7.200	1465391	137450	0.000		M	
2	7.535	1521283	130328	0.000		V M	
总计		2986674	267779				

peak number　retention time　area　height

Supplementary Figure 41. HPLC spectrum of 3as

〈色谱图〉

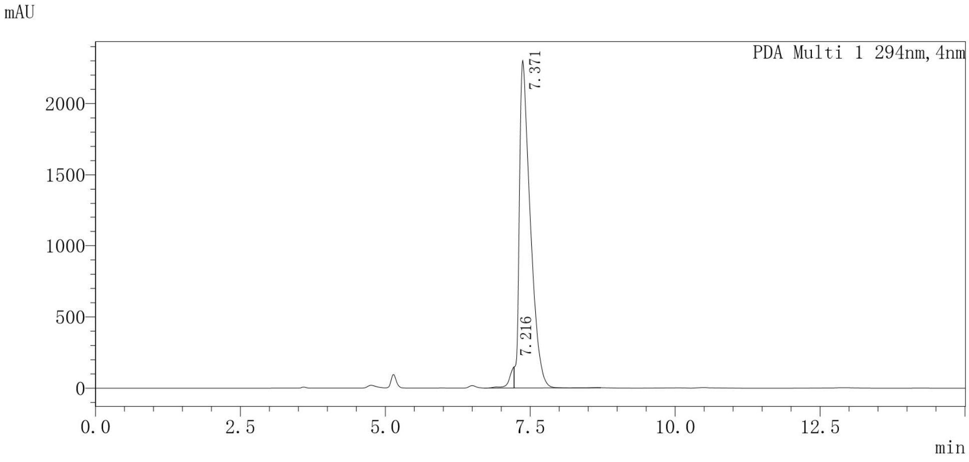

〈峰表〉

PDA Ch1 294nm

峰号	保留时间	面积	高度	浓度	浓度单位	标记	化合物名
1	7.216	868858	144377	0.000		M	
2	7.371	29961555	2304070	0.000		V M	
总计		30830413	2448446				

peak number　retention time　area　height

Supplementary Figure 42. HPLC spectrum of (*3aS,6R,7aR,Z*)-3as

(*3aS,6R,7aR,Z*)-5-(1-(dibenzo[*b,d*]thiophen-2-yl)ethylidene)-7a-hydroxy-6-methylhexahydro-3a,6-methanoinden-3(2*H*)-one (**3at**)

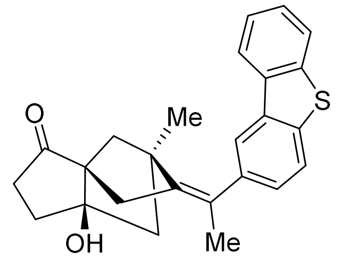

Chemical Formula: $C_{25}H_{24}O_2S$
Exact Mass: 388.1497

3at was prepared according to general procedure using **1a** (0.1 mmol, 20.4 mg) and **2t** and was purified by silica gel column chromatography (petroleum ether/ethyl acetate = 20/1~1/1) to obtain **3at** (25.2 mg, 65% yield). ^{1}H NMR (400 MHz, $CDCl_3$) δ 8.13 (dd, *J* = 6.0, 2.8 Hz, 1H), 7.90 (bs, 1H), 7.94-7.82 (m, 1H), 7.76 (d, *J* = 8.0 Hz, 1H), 7.48-7.43 (m, 2H), 7.24 (d, *J* = 7.2 Hz, 1H), 2.81-2.51 (m, 4H), 2.33-2.24 (m, 1H), 2.18-2.10 (m, 1H), 2.08-1.99 (m, 1H), 2.03 (s, 3H), 1.97-1.83 (m, 2H), 1.70 (dd, *J* = 9.7, 3.2 Hz, 1H), 1.66 (dd, *J* = 9.7, 3.3 Hz, 1H), 0.57 (s, 3H);

^{13}C NMR (101 MHz, $CDCl_3$) δ 217.5, 140.5, 139.8, 139.2, 137.4, 137.3, 135.5, 128.5, 127.6, 126.7, 124.3, 122.9, 122.0, 121.5, 121.2, 86.7, 64.4, 55.6, 54.2, 52.9, 38.2, 35.9, 33.7, 23.6, 20.6;

HRMS: (ESI) calcd for $C_{25}H_{25}O_2S^+$[M+H]$^+$ 389.1570; found 389.1570.

The enantiomeric purity was established by HPLC analysis using a chiral column: AD-H column, 30 °C, *n*-Hexane/*i*-Propanol = 80/20 as eluent, 254 nm, 1 mL/min. tR = 4.5 min (minor), 5.1 min (major).

Optical Rotation: $[\alpha]_D^{25}$ -58.5 (c 0.9, ^{i}PrOH) for 95% ee.

Absolute stereochemistry was determined through analogy with **3aa**.

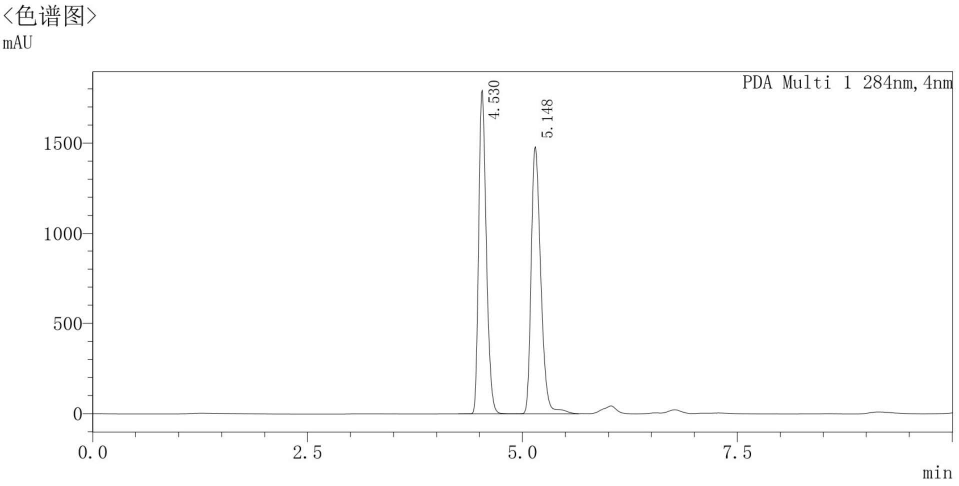

<峰表>

PDA Ch1 284nm

峰号	保留时间	面积	高度	浓度	浓度单位	标记	化合物名
1	4.530	11059761	1795335	0.000		M	
2	5.148	11529979	1481691	0.000		M	
总计		22589740	3277026				

peak number   retention time   area   height

Supplementary Figure 43. HPLC spectrum of 3at

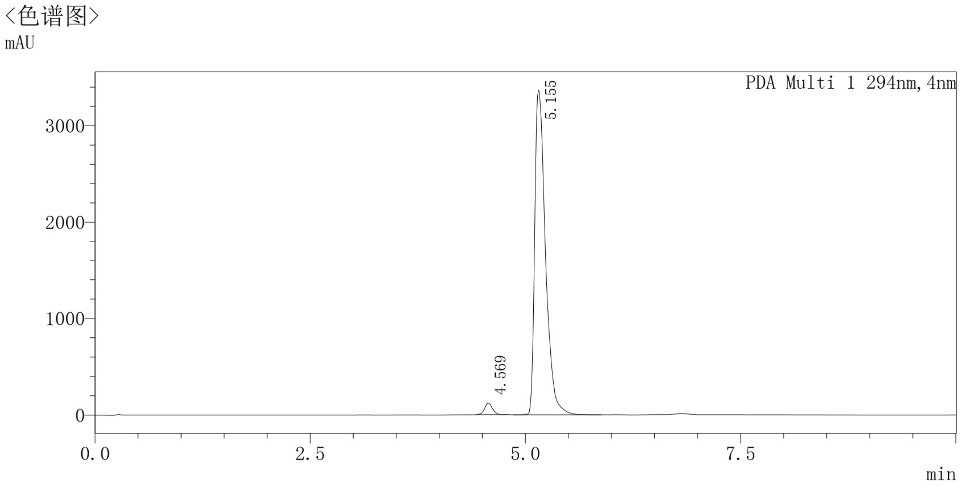

<峰表>

PDA Ch1 294nm

峰号	保留时间	面积	高度	浓度	浓度单位	标记	化合物名
1	4.569	766705	120367	0.000		M	
2	5.155	29280218	3365168	0.000		M	
总计		30046923	3485535				

peak number   retention time   area   height

Supplementary Figure 44. HPLC spectrum of (*3aS,6R,7aR,Z*)-3at

(*3aS,6R,7aR,Z*)-5-(1-(dibenzo[*b,d*]furan-2-yl)ethylidene)-7a-hydroxy-6-methylhexahydro-3a,6-methanoinden-3(2*H*)-one (**3au**)

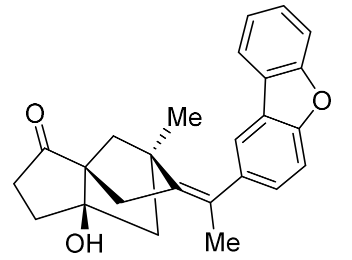

Chemical Formula: $C_{25}H_{24}O_3$
Exact Mass: 372.1725

3au was prepared according to general procedure using **1a** (0.1 mmol, 20.4 mg) and **2u** and was purified by silica gel column chromatography (petroleum ether/ethyl acetate = 20/1~1/1) to obtain **3au** (23.1 mg, 62% yield). ^{1}H NMR (400 MHz, $CDCl_3$) δ 7.95-7.90 (m, 1H), 7.69 (s, 1H), 7.60-7.54 (m, 1H), 7.51-7.42 (m, 2H), 7.34 (td, *J* = 7.4, 1.0 Hz, 1H), 7.21 (d, *J* = 8.2 Hz, 1H), 2.82-2.50 (m, 4H), 2.33-2.24 (m, 1H), 2.18-2.08 (m, 1H), 2.07-1.99 (m, 1H), 2.01 (s, 3H), 1.89 (dd, *J* = 12.7, 3.2 Hz, 1H), 1.82 (s, 1H), 1.73-1.64 (m, 2H), 0.55 (s, 3H);

^{13}C NMR (101 MHz, $CDCl_3$) δ 217.5, 156.5, 154.9, 139.0, 138.8, 128.6, 127.7, 127.1, 124.2, 122.6, 120.5, 120.1, 111.7, 110.8, 86.7, 64.4, 55.6, 54.1, 52.9, 38.2, 35.9, 33.6, 23.7, 20.5;

HRMS: (ESI) calcd for $C_{25}H_{25}O_3^+$[M+H]$^+$ 373.1798; found 373.1785.

The enantiomeric purity was established by HPLC analysis using a chiral column: AD-H column, 30 °C, *n*-Hexane/*i*-Propanol = 80/20 as eluent, 254 nm, 1 mL/min. tR = 4.4 min (minor), 4.9 min (major).

Optical Rotation: $[\alpha]_D^{25}$ -70.5 (c 0.8, ^{i}PrOH) for 93% ee.

Absolute stereochemistry was determined through analogy with **3aa**.

<色谱图>

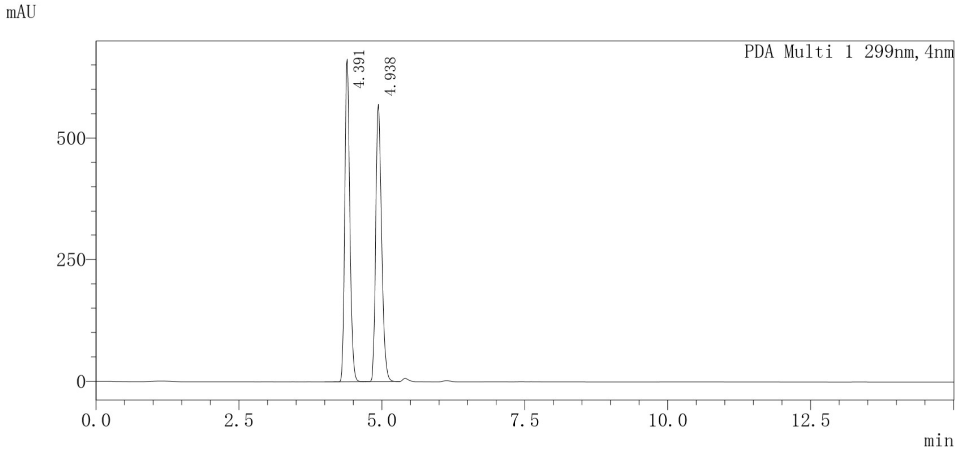

<峰表>

PDA Ch1 299nm

峰号	保留时间	面积	高度	浓度	浓度单位	标记	化合物名
1	4.391	4041613	662903	49.912		M	
2	4.938	4055870	569781	50.088		M	
总计		8097483	1232684				

peak number　area　height

retention time

Supplementary Figure 45. HPLC spectrum of 3au

<色谱图>

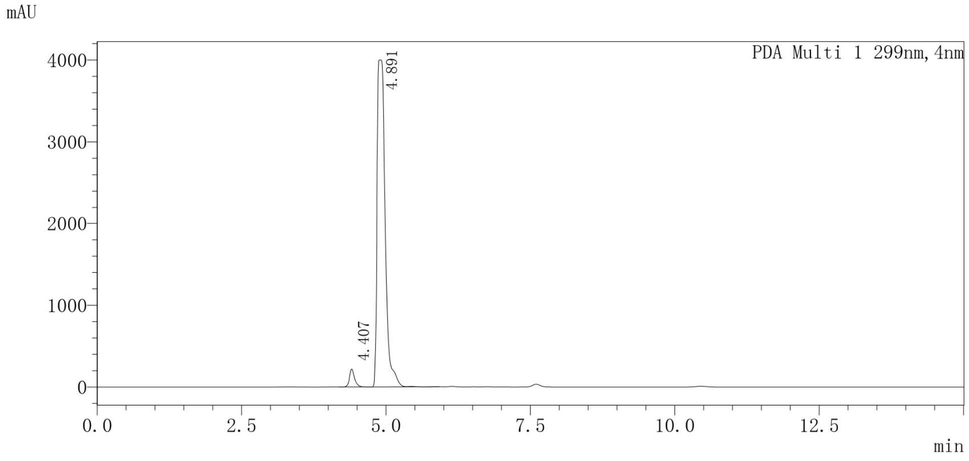

<峰表>

PDA Ch1 299nm

峰号	保留时间	面积	高度	浓度	浓度单位	标记	化合物名
1	4.407	1353779	221275	0.000		M	
2	4.891	38625211	3998636	0.000		M	
总计		39978989	4219911				

peak number　area　height

retention time

Supplementary Figure 46. HPLC spectrum of (*3aS,6R,7aR,Z*)-3au

($3aS,6R,7aR,Z$)-7a-hydroxy-6-methyl-5-(1-(pyrimidin-5-yl)ethylidene)hexahydro-3a,6-methanoinden-3(2*H*)-one (**3av**)

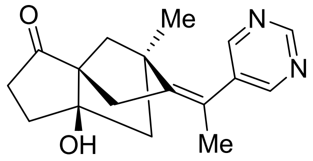

Chemical Formula: $C_{17}H_{20}N_2O_2$
Exact Mass: 284.1525

3av was prepared according to general procedure using **1a** (0.1 mmol, 20.4 mg) and **2v** and was purified by silica gel column chromatography (petroleum ether/ethyl acetate = 10/1~1/1) to obtain **3av** (18.7 mg, 66% yield). ^{1}H NMR (400 MHz, $CDCl_3$) δ 9.08 (s, 1H), 8.53 (s, 2H), 2.72 (s, 2H), 2.70-2.47 (m, 2H), 2.47-2.30 (bs, 1H), 2.30-2.22 (m, 1H), 2.20-2.11 (m, 1H), 2.10-2.05 (m, 1H), 1.95 (t, *J* = 1.2 Hz, 3H), 1.79 (dd, *J* = 12.7, 3.5 Hz, 1H), 1.73 (d, *J* = 9.8 Hz, 1H), 1.65 (dd, *J* = 9.9, 3.6 Hz, 1H), 0.61 (s, 3H);

^{13}C NMR (101 MHz, $CDCl_3$) δ 216.7, 156.7, 156.0, 144.4, 137.4, 124.9, 121.0, 86.1, 64.2, 55.5, 54.2, 52.6, 38.1, 36.2, 33.9, 22.9, 21.4;

HRMS: (ESI) calcd for $C_{17}H_{21}N_2O_2^+$[M+H]$^+$ 285.1598; found 285.1594.

The enantiomeric purity was established by HPLC analysis using a chiral column: AD-H column, 30 °C, *n*-Hexane/*i*-Propanol = 70/30 as eluent, 254 nm, 1 mL/min. tR = 6.7 min (minor), 9.5 min (major).

Optical Rotation: $[\alpha]_D^{25}$ -52.5 (c 0.6, ^{i}PrOH) for 98% ee.

Absolute stereochemistry was determined through analogy with **3aa**.

<色谱图>

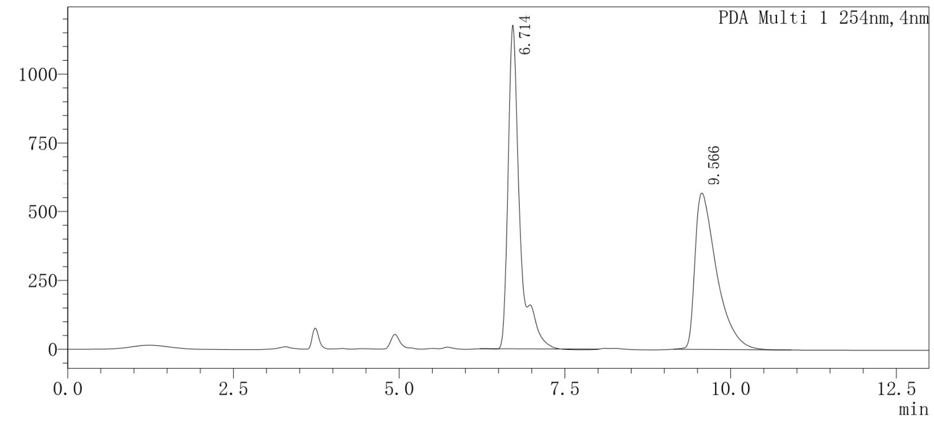

<峰表>

PDA Ch1 254nm

峰号	保留时间	面积	高度	浓度	浓度单位	标记	化合物名
1	6.714	14353531	1175785	52.613		M	
2	9.566	12927970	567803	47.387		M	
总计		27281501	1743588				

peak number

retention time

area

height

Supplementary Figure 47. HPLC spectrum of 3av

<色谱图>

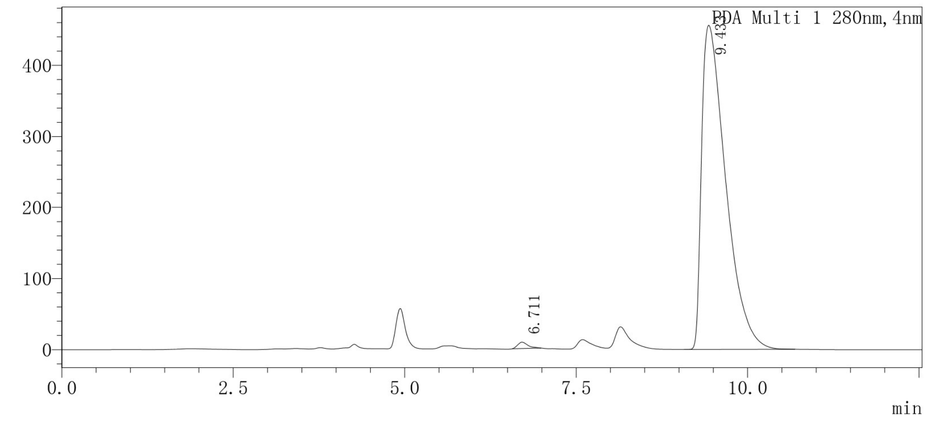

<峰表>

PDA Ch1 280nm

峰号	保留时间	面积	高度	浓度	浓度单位	标记	化合物名
1	6.711	92664	8913	0.824		M	
2	9.433	11149454	455908	99.176		M	
总计		11242118	464821				

peak number

retention time

area

height

Supplementary Figure 48. HPLC spectrum of (*3aS,6R,7aR,Z*)-3av

(*8R,9S,13S,14S*)-3-((*Z*)-1-((*3aS,6R,7aR*)-7a-hydroxy-6-methyl-3-oxohexahydro-3a,6-methanoinden-5(4*H*)-ylidene)ethyl)-13-methyl-6,7,8,9,11,12,13,14,15,16-decahydro-17*H*-cyclopenta[*a*]phenanthren-17-one (**3aw**)

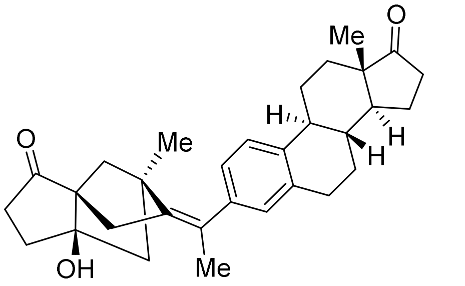

Chemical Formula: $C_{31}H_{38}O_3$
Exact Mass: 458.2821

3aw was prepared according to general procedure using **1a** (0.1 mmol, 20.4 mg) and **2w** and was purified by silica gel column chromatography (petroleum ether/ethyl acetate = 10/1~1/2) to obtain **3aw** (36.6 mg, 80% yield). ^{1}H NMR (400 MHz, $CDCl_3$) δ 7.17 (d, *J* = 7.9 Hz, 1H), 6.88 (d, *J* = 7.9 Hz, 1H), 6.84 (s, 1H), 2.94-2.80 (m, 2H), 2.71 (d, *J* = 15.7 Hz, 1H), 2.67-2.37 (m, 5H), 2.34-2.22 (m, 2H), 2.20-1.92 (m, 7H), 1.90 (s, 3H), 1.83 (dd, *J* = 12.7, 3.4 Hz, 1H), 1.72-1.41 (m, 8H), 0.91 (s, 3H), 0.62 (s, 3H);

^{13}C NMR (101 MHz, $CDCl_3$) δ 221.1, 217.7, 141.4, 138.1, 137.7, 135.6, 128.8, 128.6, 125.9, 124.5, 86.7, 75.0, 64.5, 55.5, 54.0, 52.9, 50.5, 48.0, 44.3, 38.2, 35.8, 33.6, 31.6, 29.3, 26.5, 25.6, 24.8, 23.3, 21.6, 20.5, 13.9;

HRMS: (ESI) calcd for $C_{31}H_{39}O_3^+$[M+H]$^+$ 459.2894; found 459.2881.

Optical Rotation: $[\alpha]_D^{21}$ -29.8 (c 0.9, ^{i}PrOH) for 98% ee.

Absolute stereochemistry was determined through analogy with **3aa**.

(*3aS,6R,7aR,Z*)-7a-hydroxy-6-methyl-5-((4-(methylsulfonyl)phenyl)(phenyl) methylene)hexahydro-3*a*,6-methanoinden-3(2*H*)-one (**3bi**)

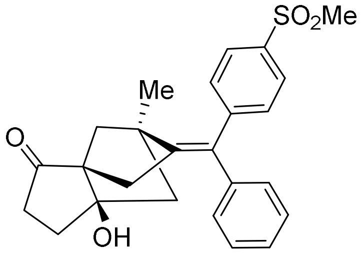

Chemical Formula: $C_{25}H_{26}O_4S$
Exact Mass: 422.1552

3bi was prepared according to general procedure using **1b** (0.1 mmol, 26.6 mg) and **2i** and was purified by silica gel column chromatography (petroleum ether/ethyl acetate = 5/1~1/1) to obtain **3bi** (25.7 mg, 61% yield). ^{1}H NMR (400 MHz, $CDCl_3$) δ 7.88-7.82 (m, 2H), 7.39 (d, *J* = 7.7 Hz, 2H), 7.31-7.26 (m, 2H), 7.22-7.14 (m, 3H), 3.04 (s, 3H), 2.92 (d, *J* = 16.5 Hz, 1H), 2.64-2.42 (m, 3H), 2.30-2.21 (m, 1H), 2.17-2.07 (m, 2H), 1.96 (d, *J* = 12.8 Hz, 1H), 1.84-1.73 (m, 3H), 0.68 (s, 3H);

^{13}C NMR (101 MHz, $CDCl_3$) δ 216.8, 148.3, 144.6, 142.1, 138.6, 133.4, 130.5, 128.6, 128.5, 127.0, 86.1, 64.5, 55.7, 54.4, 53.0, 44.5, 38.2, 35.9, 35.5, 20.2;

HRMS: (ESI) calcd for $C_{25}H_{27}O_4S^+$[M+H]$^+$ 423.1625; found 423.1630.

The enantiomeric purity was established by HPLC analysis using a chiral column: AD-H column, 30 °C, *n*-Hexane/*i*-Propanol = 70/30 as eluent, 254 nm, 1 mL/min. tR = 5.9 min (minor), 6.5 min (major).

Optical Rotation: $[\alpha]_D^{25}$ -83.4 (c 0.8, ^{i}PrOH) for 99% ee.

Absolute stereochemistry was determined through analogy with **3aa**.

<色谱图>

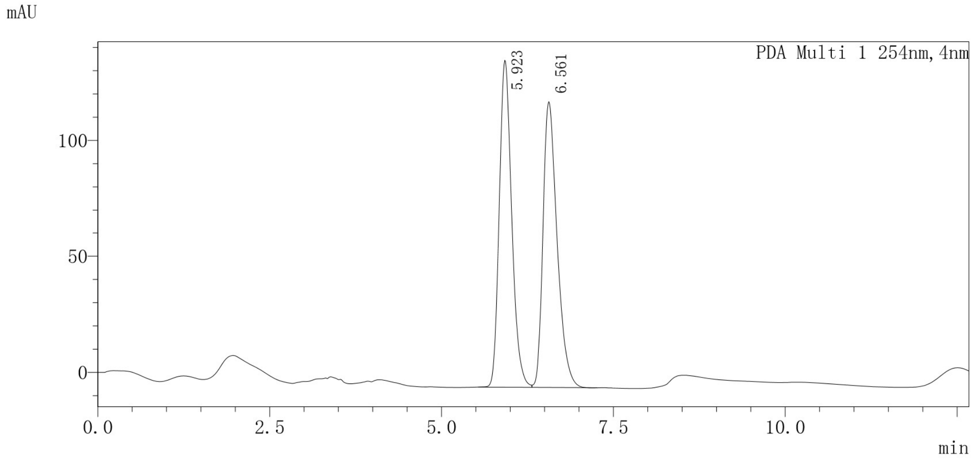

<峰表>

PDA Ch1 254nm

峰号	保留时间	面积	高度	浓度	浓度单位	标记	化合物名
1	5.923	1773559	140917	0.000		M	
2	6.561	1753038	123191	0.000		V M	
总计		3526597	264107				

peak number　retention time　area　height

Supplementary Figure 49. HPLC spectrum of (*3aS,6R,7aR,Z*)-3bi

<色谱图>

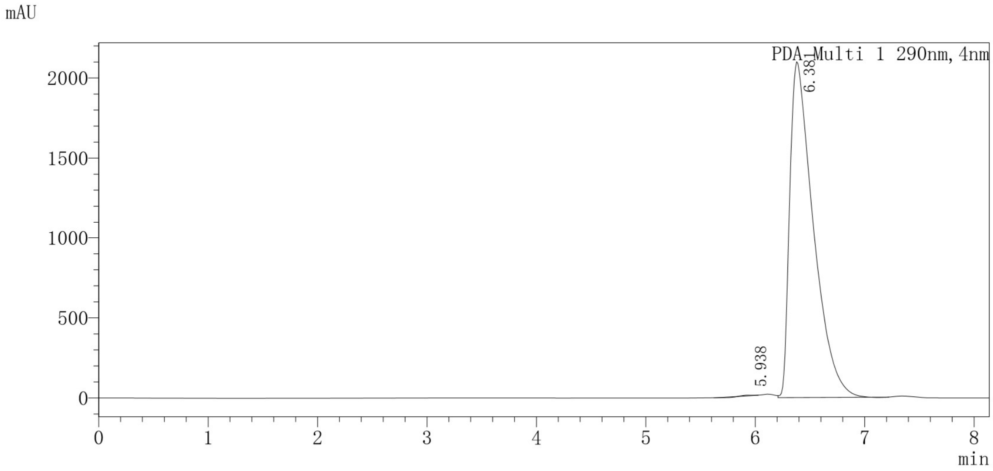

<峰表>

PDA Ch1 290nm

峰号	保留时间	面积	高度	浓度	浓度单位	标记	化合物名
1	5.938	6472	4396	0.000		M	
2	6.381	31378932	2099408	0.000		M	
总计		31385404	2103804				

peak number　retention time　area　height

Supplementary Figure 50. HPLC spectrum of (*3aS,6R,7aR,Z*)-3bi

4-((*Z*)-((*3aS,6R,7aR*)-7a-hydroxy-6-methyl-3-oxohexahydro-3a,6-methanoinden-5(4*H*)-ylidene)(4-(methylsulfonyl)phenyl)methyl)benzonitrile (**3ci**)

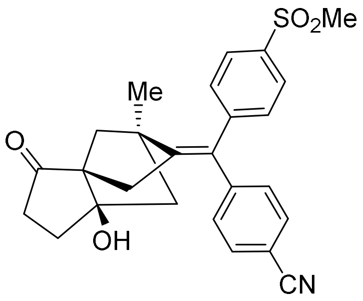

Chemical Formula: $C_{26}H_{25}NO_4S$
Exact Mass: 447.1504

3ci was prepared according to general procedure using **1c** (0.1 mmol, 29.1 mg) and **2i** and was purified by silica gel column chromatography (petroleum ether/ethyl acetate = 5/1~1/1) to obtain **3ci** (25.5 mg, 57% yield). ^{1}H NMR (400 MHz, $CDCl_3$) δ 7.88 (d, *J* = 8.6 Hz, 2H), 7.56 (d, *J* = 8.6 Hz, 2H), 7.38 (d, *J* = 7.4 Hz, 2H), 7.28 (d, *J* = 8.6 Hz, 2H), 3.06 (s, 3H), 2.85 (d, *J* = 16.4 Hz, 1H), 2.66-2.42 (m, 3H), 2.29-2.21 (m, 1H), 2.20-2.09 (m, 2H), 2.00-1.85 (m, 2H), 1.84-1.75 (m, 2H), 0.67 (s, 3H);

^{13}C NMR (101 MHz, $CDCl_3$) δ 216.3, 147.2, 146.9, 146.7, 139.3, 132.4, 131.8, 130.6, 129.5, 127.2, 118.6, 110.6, 85.8, 64.4, 55.5, 54.8, 52.8, 44.5, 38.1, 36.2, 35.6, 20.1;

HRMS: (ESI) calcd for $C_{26}H_{25}NO_4SNa^+$[M+Na]$^+$ 470.1397; found 470.1374.

The enantiomeric purity was established by HPLC analysis using a chiral column: AD-H column, 30 °C, *n*-Hexane/*i*-Propanol = 70/30 as eluent, 254 nm, 1 mL/min. tR = 9.4 min (major), 17.0 min (minor).

Optical Rotation: $[\alpha]_D^{25}$ -49.6 (c 0.8, ^{i}PrOH) for 99% ee.

Absolute stereochemistry was determined through analogy with **3aa**.

〈色谱图〉

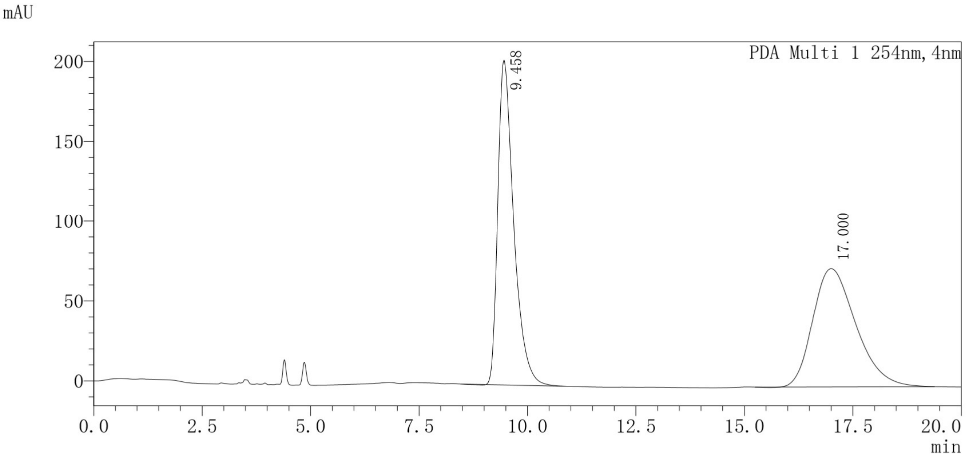

〈峰表〉

PDA Ch1 254nm

峰号	保留时间	面积	高度	浓度	浓度单位	标记	化合物名
1	9.458	5179912	203285	0.000		M	
2	17.000	5104875	73997	0.000		M	
总计		10284787	277283				

peak number　retention time　area　height

Supplementary Figure 51. HPLC spectrum of 3ci

〈色谱图〉

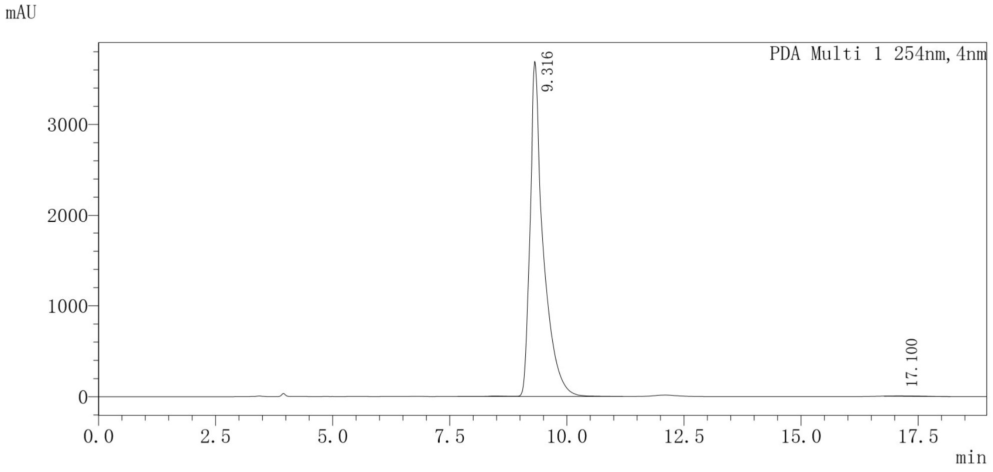

〈峰表〉

PDA Ch1 254nm

峰号	保留时间	面积	高度	浓度	浓度单位	标记	化合物名
1	9.316	72534864	3691883	0.000		M	
2	17.100	131693	3142	0.000		M	
总计		72666557	3695025				

peak number　retention time　area　height

Supplementary Figure 52. HPLC spectrum of (*3aS,6R,7aR,Z*)-3ci

(*3aS,6R,7aR,Z*)-7a-hydroxy-5-((4-methoxyphenyl)(4-(methylsulfonyl)phenyl) methylene)-6-methylhexahydro-3a,6-methanoinden-3(2*H*)-one (**3di**)

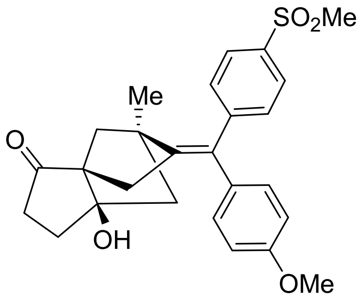

Chemical Formula: $C_{26}H_{28}O_5S$
Exact Mass: 452.1657

3di was prepared according to general procedure using **1d** (0.1 mmol, 29.6 mg) and **2i** and was purified by silica gel column chromatography (petroleum ether/ethyl acetate = 5/1~1/1) to obtain **3di** (27.6 mg, 61% yield). ^{1}H NMR (400 MHz, $CDCl_3$) δ 7.84 (d, *J* = 8.7 Hz, 2H), 7.37 (d, *J* = 7.7 Hz, 2H), 7.07 (d, *J* = 8.8 Hz, 2H), 6.80 (d, *J* = 8.8 Hz, 2H), 3.76 (s, 3H), 3.04 (s, 3H), 2.95 (d, *J* = 16.5 Hz, 1H), 2.66-2.43 (m, 3H), 2.30-2.19 (m, 1H), 2.17-2.05 (m, 2H), 1.95 (d, *J* = 12.7 Hz, 1H), 1.85 (bs, 1H), 1.78 (s, 2H), 0.66 (s, 3H);

^{13}C NMR (101 MHz, $CDCl_3$) δ 216.9, 158.4, 148.7, 144.0, 138.5, 134.5, 133.0, 130.5, 129.9, 126.9, 113.8, 86.1, 64.5, 55.9, 55.2, 54.4, 53.1, 44.5, 38.2, 35.9, 35.7, 20.2;

HRMS: (ESI) calcd for $C_{26}H_{29}O_5S^+$[M+H]$^+$ 453.1730; found 453.1726.

The enantiomeric purity was established by HPLC analysis using a chiral column: AD-H column, 30 °C, *n*-Hexane/*i*-Propanol = 80/20 as eluent, 254 nm, 1 mL/min. tR = 14.4 min (major), 15.5 min (minor).

Optical Rotation: $[\alpha]_D^{25}$ -197.6 (c 0.9, ^{i}PrOH) for 97% ee.

Absolute stereochemistry was determined through analogy with **3aa**.

〈色谱图〉

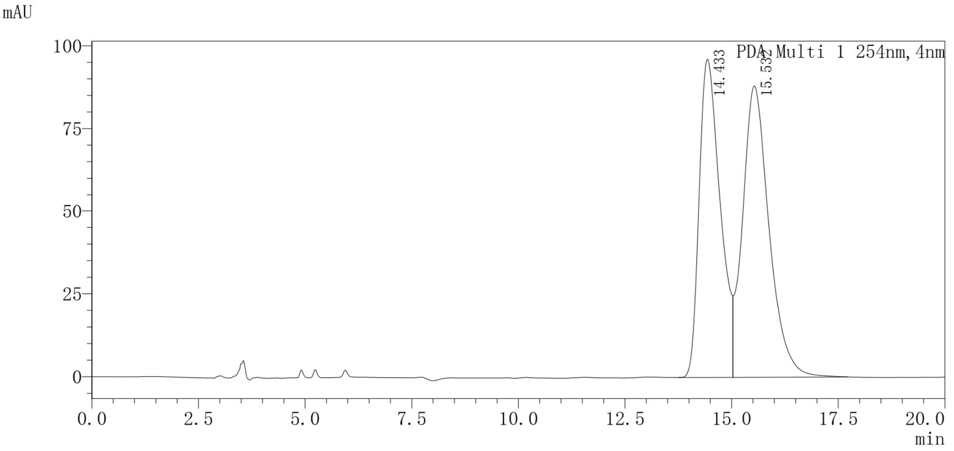

〈峰表〉

PDA Ch1 254nm

峰号	保留时间	面积	高度	浓度	浓度单位	标记	化合物名
1	14.433	3300446	96182	0.000			
2	15.532	3800681	88014	0.000		V	
总计		7101127	184196				

peak number · retention time · area · height

Supplementary Figure 53. HPLC spectrum of 3di

〈色谱图〉

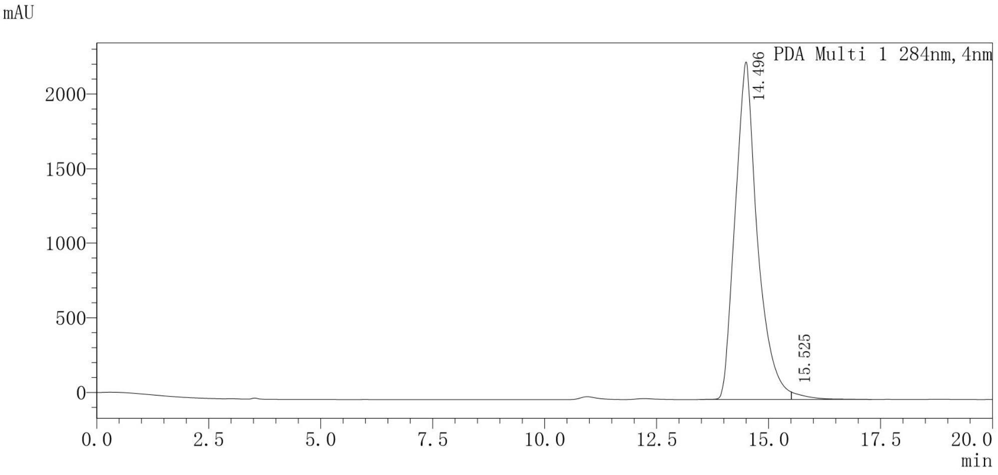

〈峰表〉

PDA Ch1 284nm

峰号	保留时间	面积	高度	浓度	浓度单位	标记	化合物名
1	14.496	78258990	2262297	0.000		M	
2	15.525	1028324	48048	0.000		V M	
总计		79287314	2310345				

peak number · retention time · area · height

Supplementary Figure 54. HPLC spectrum of (*3aS,6R,7aR,Z*)-3di

(*3aS,6R,7aR,E*)-5-(dibenzo[*b,d*]furan-2-yl(4-(methylsulfonyl)phenyl)methylene)-7a-hydroxy-6-methylhexahydro-3a,6-methanoinden-3(2*H*)-one (**3ei**)

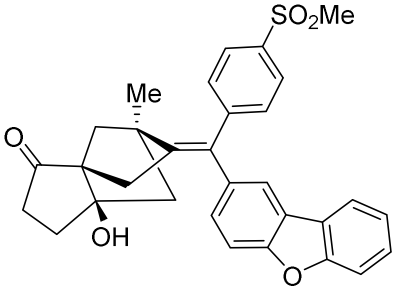

Chemical Formula: $C_{31}H_{28}O_5S$
Exact Mass: 512.1657

3ei was prepared according to general procedure using **1e** (0.1 mmol, 35.6 mg) and **2i** and was purified by silica gel column chromatography (petroleum ether/ethyl acetate = 5/1~1/1) to obtain **3ei** (29.2 mg, 57% yield). ^{1}H NMR (400 MHz, $CDCl_3$) δ 7.89 (dd, *J* = 12.0, 7.6 Hz, 3H), 7.74 (d, *J* = 1.2 Hz, 1H), 7.54 (d, *J* = 8.0 Hz, 1H), 7.51-7.41 (m, 4H), 7.33 (td, *J* = 7.6, 1.2 Hz, 1H), 7.24 (d, *J* = 2.0 Hz, 1H), 3.06 (s, 3H), 2.99 (d, *J* = 16.4 Hz, 1H), 2.63-2.45 (m, 3H), 2.27 (dd, *J* = 12.4, 7.2 Hz, 1H), 2.20-2.11 (m, 2H), 2.05-1.99 (m, 1H), 1.88-1.80 (m, 2H), 1.63 (s, 1H), 0.74 (s, 3H);

^{13}C NMR (101 MHz, $CDCl_3$) δ 216.6, 156.5, 155.0, 148.5, 144.9, 138.8, 136.9, 133.5, 130.5, 127.9, 127.4, 127.1, 124.5, 123.9, 122.8, 120.8, 111.8, 111.7, 86.2, 64.5, 55.9, 54.5, 53.1, 44.5, 38.3, 36.0, 35.7, 20.2;

HRMS: (ESI) calcd for $C_{31}H_{29}O_5S^+[M+H]^+$ 513.1730; found 513.1728.

The enantiomeric purity was established by HPLC analysis using a chiral column: AD-H column, 30 °C, *n*-Hexane/*i*-Propanol = 85/15 as eluent, 254 nm, 1 mL/min. tR = 34 min (minor), 37 min (major).

Optical Rotation: $[\alpha]_D^{25}$ -50.2 (c 1.0, ^{i}PrOH) for 99% ee.

Absolute stereochemistry was determined through analogy with **3aa**.

<色谱图>

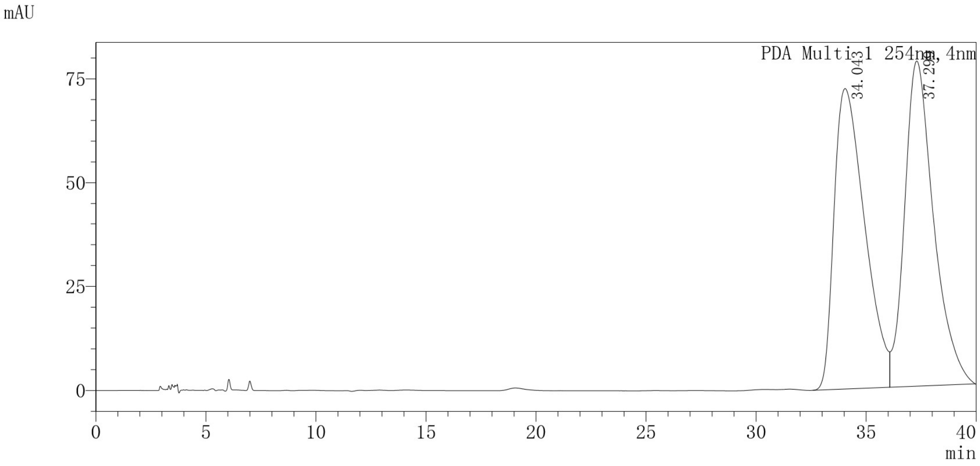

<峰表>

PDA Ch1 254nm

峰号	保留时间	面积	高度	浓度	浓度单位	标记	化合物名
1	34.043	6992287	72323	0.000			
2	37.299	7186030	78272	0.000		V	
总计		14178317	150595				

peak number
retention time
area
height

Supplementary Figure 55. HPLC spectrum of 3ei

<色谱图>

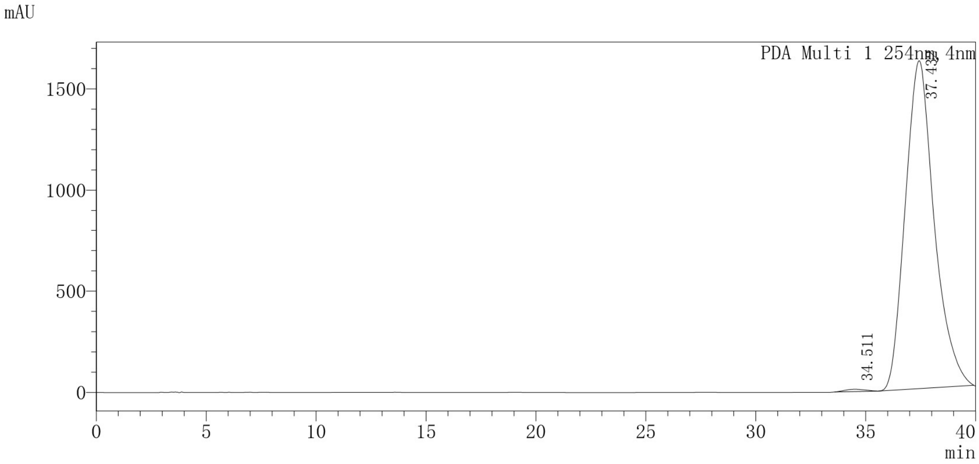

<峰表>

PDA Ch1 254nm

峰号	保留时间	面积	高度	浓度	浓度单位	标记	化合物名
1	34.511	716417	11048	0.000		M	
2	37.432	148371397	1619849	0.000		M	
总计		149087815	1630897				

peak number
retention time
area
height

Supplementary Figure 56. HPLC spectrum of (*3aS,6R,7aR,Z*)-3ei

(*8R,9S,13S,14S*)-3-((*E*)-((*3aS,6R,7aR*)-7a-hydroxy-6-methyl-3-oxohexahydro-3a,6-methanoinden-5(4*H*)-ylidene)(4-(methylsulfonyl)phenyl)methyl)-13-methyl-6,7,8,9,11,12,13,14,15,16-decahydro-17*H*-cyclopenta[*a*]phenanthren-17-one (**3fi**)

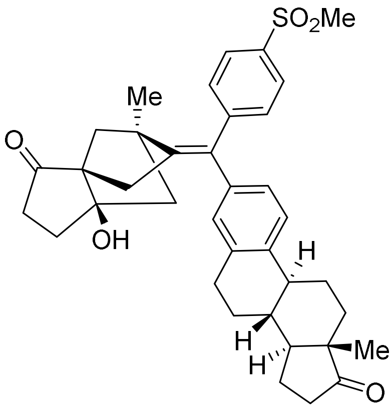

Chemical Formula: $C_{37}H_{42}O_5S$
Exact Mass: 598.2753

3fi was prepared according to general procedure using **1f** (0.1 mmol, 44.2 mg) and **2i** and was purified by silica gel column chromatography (petroleum ether/ethyl acetate = 5/1~1/3) to obtain **3fi** (40.7 mg, 68% yield). ^{1}H NMR (400 MHz, $CDCl_3$) δ 7.84 (d, *J* = 8.5 Hz, 2H), 7.40 (d, *J* = 7.5 Hz, 2H), 7.17 (d, *J* = 8.5 Hz, 1H), 6.93-6.86 (m, 2H), 3.04 (s, 3H), 2.91 (d, *J* = 16.5 Hz, 1H), 2.87-2.80 (m, 2H), 2.64-2.42 (m, 4H), 2.40-2.19 (m, 3H), 2.18-1.87 (m, 8H), 1.78 (s, 2H), 1.66-1.35 (m, 6H), 0.87 (s, 3H), 0.66 (s, 3H);

^{13}C NMR (151 MHz, $CDCl_3$) δ 217.0, 148.4, 144.2, 139.6, 138.5, 138.5, 136.6, 133.3, 130.4, 128.9, 126.9, 126.0, 125.4, 86.1, 64.5, 55.8, 54.4, 53.0, 50.4, 47.9, 44.5, 44.3, 38.3, 38.0, 35.9, 35.8, 35.6, 31.5, 29.4, 26.4, 25.6, 21.5, 20.2, 13.8;

HRMS: (ESI) calcd for $C_{37}H_{43}O_5S^+$[M+H]$^+$ 599.2826; found 599.2828.

Optical Rotation: $[\alpha]_D^{22}$ -174.1 (c 0.8, ^{i}PrOH) for d.r. > 20/1.

Absolute stereochemistry was determined through analogy with **3aa**.

(*3aS,6R,7aR,Z*)-7a-hydroxy-6-methyl-5-(1-(4-(methylsulfonyl)phenyl)hexylidene) hexahydro-3a,6-methanoinden-3(2*H*)-one (**3gi**)

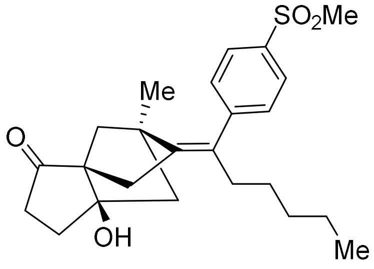

Chemical Formula: $C_{24}H_{32}O_4S$
Exact Mass: 416.2021

3gi was prepared according to general procedure using **1g** (0.1 mmol, 26.0 mg) and **2i** and was purified by silica gel column chromatography (petroleum ether/ethyl acetate = 5/1~2/1) to obtain **3gi** (36.6 mg, 88% yield). ^{1}H NMR (400 MHz, $CDCl_3$) δ 7.85 (d, *J* = 8.0 Hz, 2H), 7.26 (s, 2H), 3.07 (s, 3H), 2.71 (s, 2H), 2.69-2.48 (m, 2H), 2.37-2.20 (m, 2H), 2.20-2.06 (m, 2H), 1.99 (d, *J* = 12.7 Hz, 1H), 1.78-1.57 (m, 4H), 1.27-1.19 (m, 6H), 0.85-0.78 (m, 3H), 0.49 (s, 3H);

^{13}C NMR (101 MHz, $CDCl_3$) δ 217.2, 149.1, 140.6, 138.4, 131.8, 130.1, 126.8, 86.3, 64.2, 55.5, 54.0, 52.5, 44.5, 38.2, 36.4, 36.0, 33.1, 31.6, 26.9, 22.5, 20.6, 14.0;

HRMS: (ESI) calcd for $C_{24}H_{33}O_4S^+$[M+H]$^+$ 417.2094; found 417.2091.

The enantiomeric purity was established by HPLC analysis using a chiral column: OJ-H column, 30 °C, *n*-Hexane/*i*-Propanol = 60/40 as eluent, 254 nm, 1 mL/min. tR = 4.8 min (minor), 6.4 min (major).

Optical Rotation: $[\alpha]_D^{25}$ -30.5 (c 1.2, ^{i}PrOH) for 98% ee.

Absolute stereochemistry was determined through analogy with **3aa**.

〈色谱图〉

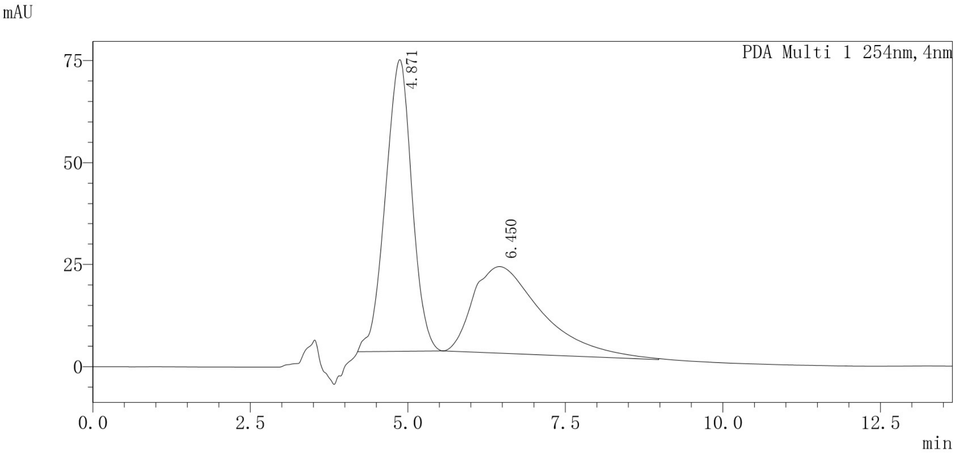

〈峰表〉

PDA Ch1 254nm

峰号	保留时间	面积	高度	浓度	浓度单位	标记	化合物名
1	4.871	2089916	71472	56.400		M	
2	6.450	1615604	21179	43.600		M	
总计		3705520	92651				

peak number
retention time
area
height

Supplementary Figure 57. HPLC spectrum of 3gi

〈色谱图〉

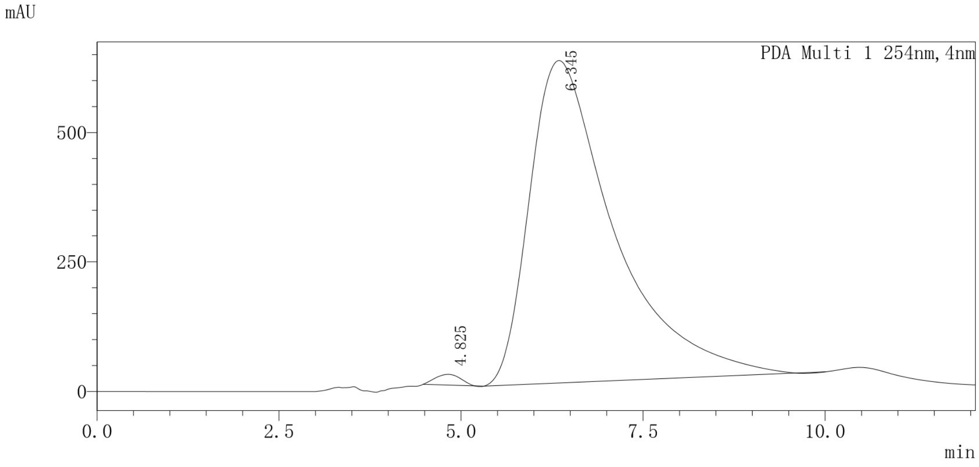

〈峰表〉

PDA Ch1 254nm

峰号	保留时间	面积	高度	浓度	浓度单位	标记	化合物名
1	4.825	496212	20728	0.000		M	
2	6.345	50417747	622604	0.000		M	
总计		50913958	643332				

peak number
retention time
area
height

Supplementary Figure 58. HPLC spectrum of (*3aS,6R,7aR,Z*)-3gi

(*3aS,6R,7aR,Z*)-7a-hydroxy-6-methyl-5-(1-(4-(methylsulfonyl)phenyl)-2-phenylethylidene) hexahydro-3a,6-methanoinden-3(2*H*)-one (**3hi**)

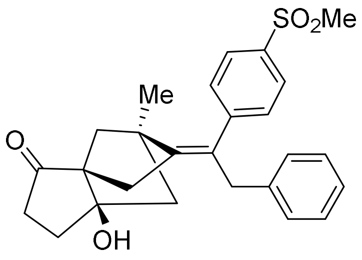

Chemical Formula: $C_{26}H_{28}O_4S$
Exact Mass: 436.1708

3hi was prepared according to general procedure using **1h** (0.1 mmol, 28.0 mg) and **2i** and was purified by silica gel column chromatography (petroleum ether/ethyl acetate = 5/1~2/1) to obtain **3hi** (34.4 mg, 79% yield). ^{1}H NMR (400 MHz, $CDCl_3$) δ 7.74 (d, *J* = 8.5 Hz, 2H), 7.23-7.10 (m, 5H), 7.08-7.02 (m, 2H), 3.68 (d, *J* = 14.7 Hz, 1H), 3.55 (d, *J* = 14.8 Hz, 1H), 3.02 (s, 3H), 2.96 (dd, *J* = 15.7, 2.8 Hz, 1H), 2.86 (d, *J* = 15.7 Hz, 1H), 2.71-2.50 (m, 2H), 2.31-2.10 (m, 2H), 2.03 (d, *J* = 12.7 Hz, 1H), 1.84 (dd, *J* = 12.8, 2.9 Hz, 2H), 1.76-1.68 (m, 2H), 0.53 (s, 3H);

^{13}C NMR (101 MHz, $CDCl_3$) δ 216.9, 148.4, 142.7, 138.5, 138.3, 130.4, 128.6, 128.4, 126.6, 126.2, 86.3, 64.2, 55.4, 54.3, 52.6, 44.5, 42.6, 38.2, 36.2, 33.9, 20.6;

HRMS: (ESI) calcd for $C_{26}H_{29}O_4S^+$[M+H]$^+$ 437.1781; found 437.1771.

The enantiomeric purity was established by HPLC analysis using a chiral column: AD-H column, 30 °C, *n*-Hexane/*i*-Propanol = 80/20 as eluent, 254 nm, 1 mL/min. tR = 9.7 min (minor), 11.9 min (major).

Optical Rotation: $[\alpha]_D^{25}$ -46.7 (c 1.1, ^{i}PrOH) for 92% ee.

Absolute stereochemistry was determined through analogy with **3aa**.

〈色谱图〉

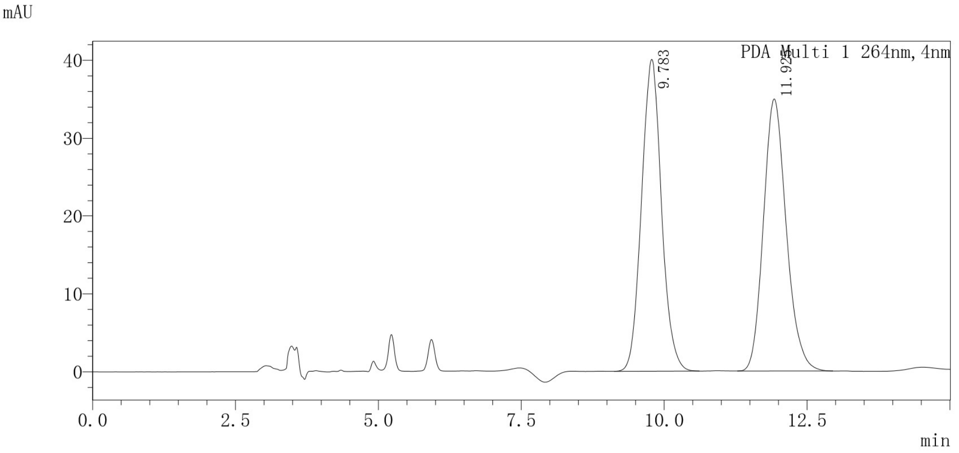

〈峰表〉

PDA Ch1 264nm

峰号	保留时间	面积	高度	浓度	浓度单位	标记	化合物名
1	9.783	973672	40041	0.000			
2	11.925	971667	34949	0.000			
总计		1945339	74989				

peak number　retention time　area　height

Supplementary Figure 59. HPLC spectrum of 3hi

〈色谱图〉

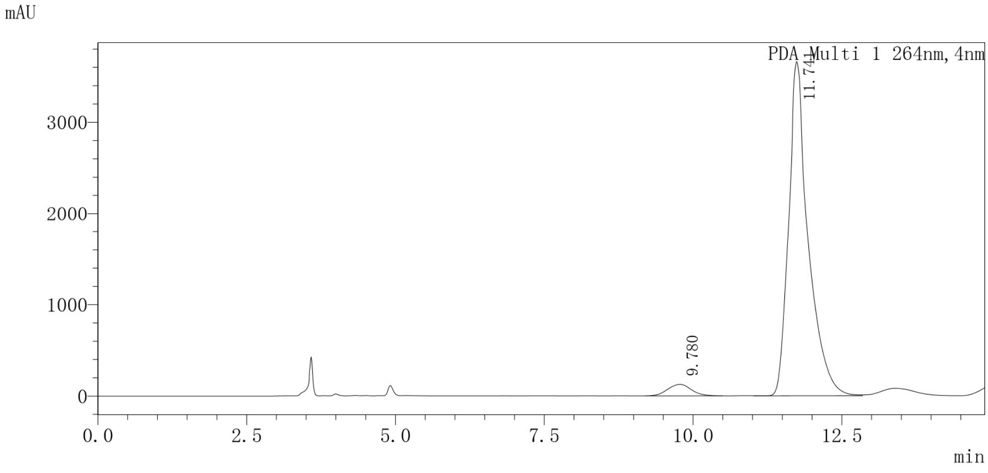

〈峰表〉

PDA Ch1 264nm

峰号	保留时间	面积	高度	浓度	浓度单位	标记	化合物名
1	9.780	3476878	126262	0.000		M	
2	11.741	83823791	3660782	0.000		M	
总计		87300669	3787044				

peak number　retention time　area　height

Supplementary Figure 60. HPLC spectrum of (*3aS,6R,7aR,Z*)-3hi

(*3aS,6R,7aR,Z*)-7a-hydroxy-6-methyl-5-(1-(4-(methylsulfonyl)phenyl)but-3-en-1-ylidene)hexahydro-3a,6-methanoinden-3(2*H*)-one (**3ii**)

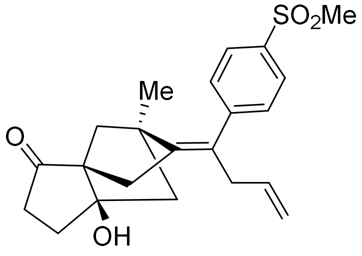

Chemical Formula: $C_{22}H_{26}O_4S$
Exact Mass: 386.1552

3ii was prepared according to general procedure using **1i** (0.1 mmol, 23.0 mg) and **2i** and was purified by silica gel column chromatography (petroleum ether/ethyl acetate = 5/1~1/1) to obtain **3ii** (22.4 mg, 58% yield). ^{1}H NMR (400 MHz, $CDCl_3$) δ 7.85 (d, *J* = 8.7 Hz, 2H), 7.31 (d, *J* = 7.7 Hz, 2H), 5.77-5.61 (m, 1H), 5.02-4.98 (m, 1H), 4.97 (t, *J* = 1.3 Hz, 1H), 3.07 (s, 3H), 3.06-2.92 (m, 2H), 2.79-2.69 (m, 2H), 2.66-2.49 (m, 2H), 2.31-2.21 (m, 1H), 2.17-2.07 (m, 1H), 2.02 (d, *J* = 12.7 Hz, 1H), 1.84-1.75 (m, 2H), 1.70 (dd, *J* = 9.9, 2.6 Hz, 1H), 1.63 (dd, *J* = 9.9, 3.5 Hz, 1H), 0.53 (s, 3H);

^{13}C NMR (101 MHz, $CDCl_3$) δ 216.9, 148.7, 142.6, 138.5, 133.9, 130.1, 129.0, 126.8, 116.1, 86.3, 64.2, 55.3, 54.2, 52.5, 44.5, 41.0, 38.2, 36.0, 33.0, 20.5;

HRMS: (ESI) calcd for $C_{22}H_{27}O_4S^+$[M+H]$^+$ 387.1625; found 387.1610.

The enantiomeric purity was established by HPLC analysis using a chiral column: AD-H column, 30 °C, *n*-Hexane/*i*-Propanol = 80/20 as eluent, 254 nm, 1 mL/min. tR = 8.0 min (minor), 9.3 min (major).

Optical Rotation: $[\alpha]_D^{25}$ -24.3 (c 0.7, ^{i}PrOH) for 98% ee.

Absolute stereochemistry was determined through analogy with **3aa**.

〈色谱图〉

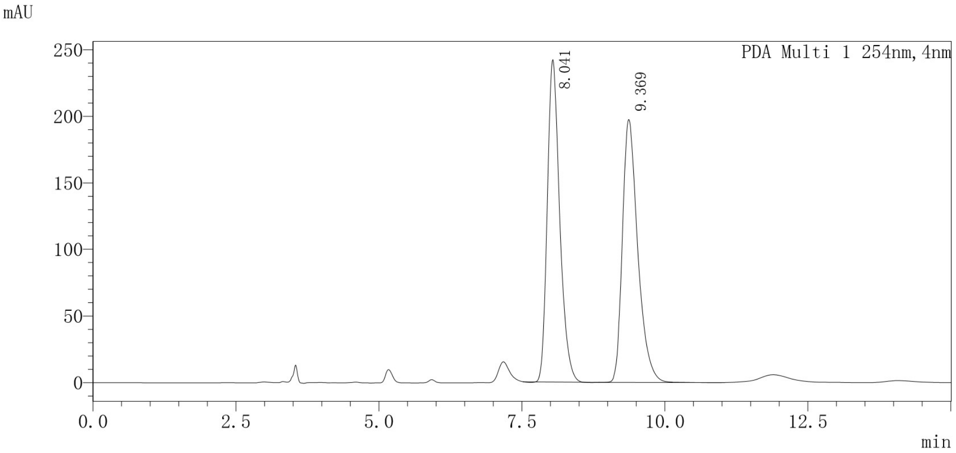

〈峰表〉

PDA Ch1 254nm

峰号	保留时间	面积	高度	浓度	浓度单位	标记	化合物名
1	8.041	3649362	242103	0.000		M	
2	9.369	3688600	197473	0.000		M	
总计		7337963	439577				

peak number　retention time　area　height

Supplementary Figure 61. HPLC spectrum of 3ii

〈色谱图〉

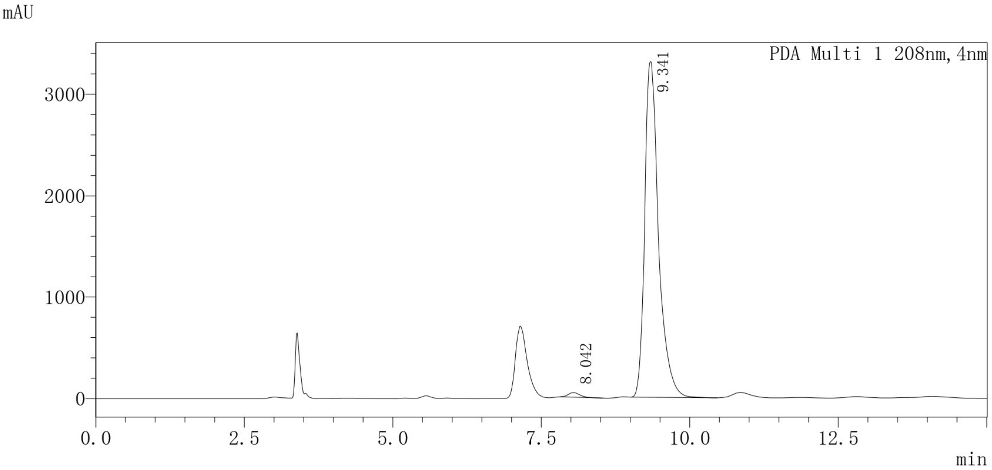

〈峰表〉

PDA Ch1 208nm

峰号	保留时间	面积	高度	浓度	浓度单位	标记	化合物名
1	8.042	609034	45934	0.000		M	
2	9.341	53734310	3310369	0.000		M	
总计		54343343	3356303				

peak number　retention time　area　height

Supplementary Figure 62. HPLC spectrum of (*3aS,6R,7aR,Z*)-3ii

(*3aS,6R,7aR,E*)-5-(2-((tert-butyldimethylsilyl)oxy)-1-(4-(methylsulfonyl)phenyl) ethylidene)-7a-hydroxy-6-methylhexahydro-3a,6-methanoinden-3(2*H*)-one (**3ji**)

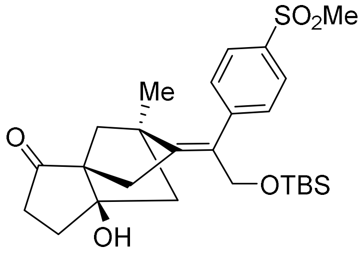

Chemical Formula: $C_{26}H_{38}O_5SSi$
Exact Mass: 490.2209

3ji was prepared according to general procedure using **1j** (0.1 mmol, 33.4 mg) and **2i** and was purified by silica gel column chromatography (petroleum ether/ethyl acetate = 5/1~2/1) to obtain **3ji** (33.3 mg, 68% yield). ^{1}H NMR (400 MHz, $CDCl_3$) δ 7.85 (d, *J* = 8.4 Hz, 2H), 7.36 (d, *J* = 8.4 Hz, 2H), 4.36 (d, *J* = 11.2 Hz, 1H), 4.24 (d, *J* = 11.2 Hz, 1H), 3.03 (s, 3H), 2.83 (dd, *J* = 15.8, 3.4 Hz, 1H), 2.73 (d, *J* = 15.8 Hz, 1H), 2.68-2.47 (m, 3H), 2.31-2.22 (m, 1H), 2.14-1.97 (m, 2H), 1.81 (dd, *J* = 12.8, 3.2 Hz, 1H), 1.70 (dd, *J* = 10.0, 3.6 Hz, 1H), 1.61 (dd, *J* = 10.0, 3.6 Hz, 1H), 0.72 (s, 9H), 0.53 (s, 3H), -0.14 (s, 3H), -0.16 (s, 3H);

^{13}C NMR (101 MHz, $CDCl_3$) δ 217.0, 147.1, 143.9, 138.6, 130.7, 130.4, 126.5, 86.1, 65.3, 64.2, 54.9, 54.1, 52.0, 44.6, 38.2, 35.7, 32.8, 25.7, 20.5, 18.2, -5.6, -5.6;

HRMS: (ESI) calcd for $C_{26}H_{38}O_5SSiNa^+$[M+Na]$^+$ 513.2101; found 513.2080.

The enantiomeric purity was established by HPLC analysis using a chiral column: OD-H column, 30 °C, *n*-Hexane/*i*-Propanol = 93/7 as eluent, 254 nm, 1 mL/min. tR = 20 min (major), 23 min (minor).

Optical Rotation: $[\alpha]_D^{25}$ -74.2(c 1.1, ^{i}PrOH) for 98% ee.

Absolute stereochemistry was determined through analogy with **3aa**.

<色谱图>

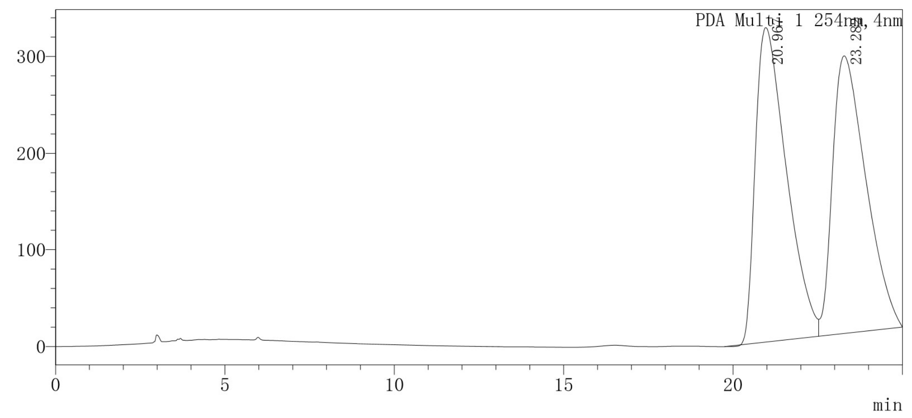

<峰表>

PDA Ch1 254nm

峰号	保留时间	面积	高度	浓度	浓度单位	标记	化合物名
1	20.967	20740307	325318	0.000		M	
2	23.282	19673931	287128	0.000		V M	
总计		40414238	612446				

peak number　retention time　area　height

Supplementary Figure 63. HPLC spectrum of 3ji

<色谱图>

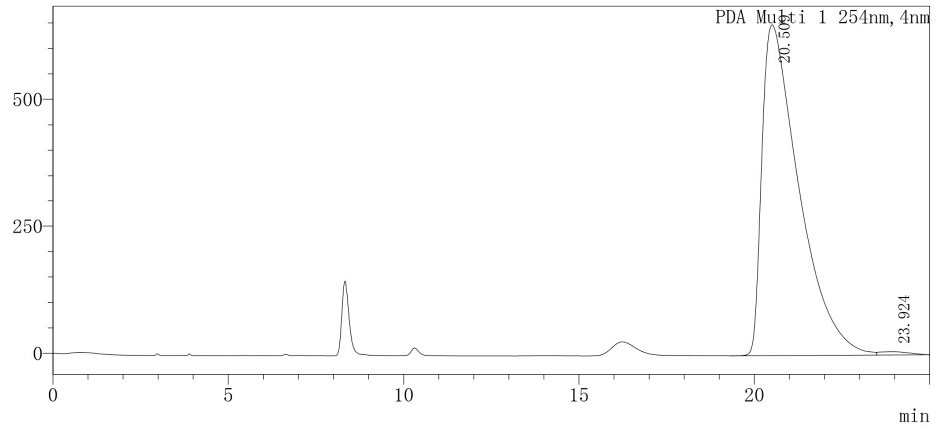

<峰表>

PDA Ch1 254nm

峰号	保留时间	面积	高度	浓度	浓度单位	标记	化合物名
1	20.509	47885382	650947	0.000		M	
2	23.924	379909	6379	0.000		V M	
总计		48265291	657326				

peak number　retention time　area　height

Supplementary Figure 64. HPLC spectrum of (*3aS,6R,7aR,Z*)-3ji

(*E*)-2-((*3aS,6R,7aR*)-7a-hydroxy-6-methyl-3-oxohexahydro-3a,6-methanoinden-5(4*H*)-ylidene)-2-(4-(methylsulfonyl)phenyl)ethyl benzoate (**3ki**)

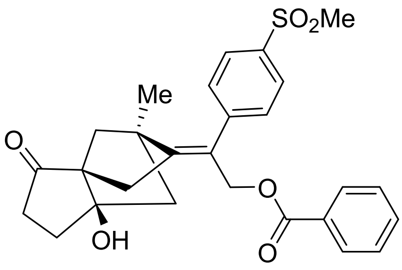

Chemical Formula: $C_{27}H_{28}O_6S$
Exact Mass: 480.1607

3ki was prepared according to general procedure using **1k** (0.1 mmol, 32.4 mg) and **2i** and was purified by silica gel column chromatography (petroleum ether/ethyl acetate = 5/1~1/1) to obtain **3ki** (38.9 mg, 81% yield). ^{1}H NMR (400 MHz, $CDCl_3$) δ 7.92-7.76 (m, 4H), 7.55-7.50 (m, 1H), 7.46-7.24 (m, 4H), 5.20 (d, *J* = 12.0 Hz, 1H), 4.74 (d, *J* = 12.0, 1H), 3.31 (d, *J* = 16.0, 1H), 3.08-2.94 (m, 1H), 3.04 (s, 3H), 2.83 (d, *J* = 16.0 Hz, 1H), 2.75-2.62 (m, 1H), 2.59-2.49 (m, 1H), 2.37-2.28 (m, 1H), 2.10-2.00 (m, 2H), 1.79-1.70 (m, 3H), 0.53 (s, 3H);

^{13}C NMR (101 MHz, $CDCl_3$) δ 216.8, 166.9, 148.5, 145.7, 139.3, 133.3, 129.6, 129.5, 129.5, 128.4, 127.0, 125.9, 86.2, 67.8, 64.3, 56.0, 54.2, 52.1, 44.4, 38.5, 35.3, 34.2, 20.0;

HRMS: (ESI) calcd for $C_{27}H_{29}O_6S^+$[M+H]$^+$ 481.1679; found 481.1678.

The enantiomeric purity was established by HPLC analysis using a chiral column: AD-H column, 30 °C, *n*-Hexane/*i*-Propanol = 70/30 as eluent, 254 nm, 1 mL/min. tR = 7.7 min (major), 11.4 min (minor).

Optical Rotation: $[\alpha]_D^{25}$ -40.4 (c 1.3, ^{i}PrOH) for 98% ee.

Absolute stereochemistry was determined through analogy with **3aa**.

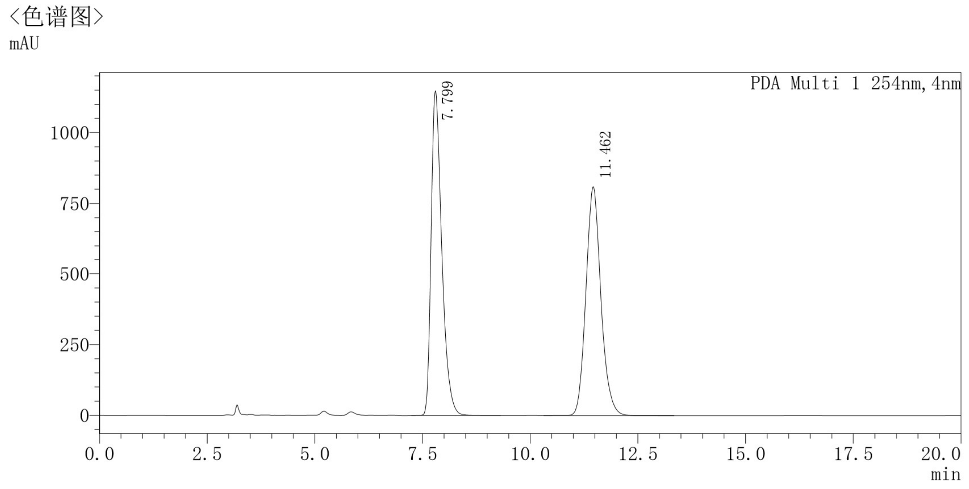

〈峰表〉

PDA Ch1 254nm

峰号	保留时间	面积	高度	浓度	浓度单位	标记	化合物名
1	7.799	19668972	1148097	0.000		M	
2	11.462	19565522	809237	0.000		M	
总计		39234494	1957334				

peak number　retention time　area　height

Supplementary Figure 65. HPLC spectrum of 3ki

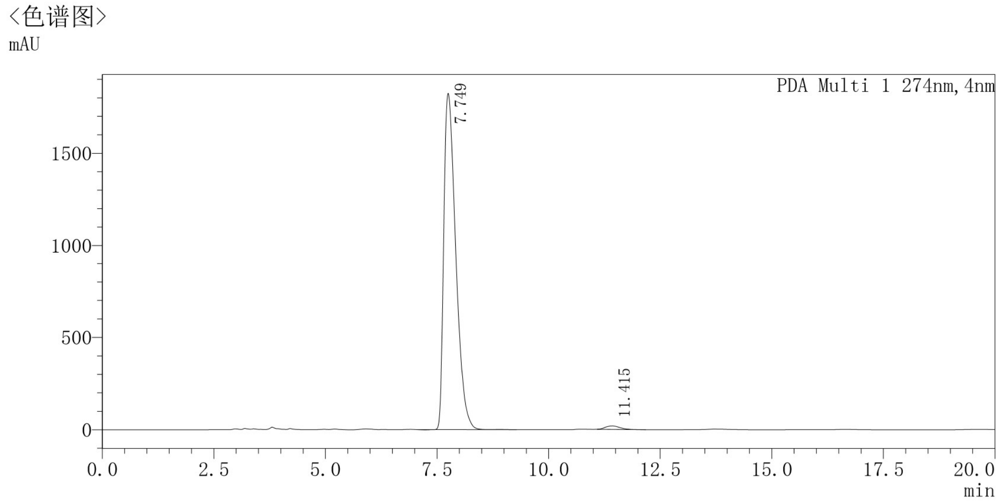

〈峰表〉

PDA Ch1 274nm

峰号	保留时间	面积	高度	浓度	浓度单位	标记	化合物名
1	7.749	34002447	1823510	0.000		M	
2	11.415	435194	19555	0.000		M	
总计		34437641	1843065				

peak number　retention time　area　height

Supplementary Figure 66. HPLC spectrum of (*3aS,6R,7aR,Z*)-3ki

($3aS,6R,7aR,E$)-7a-hydroxy-5-(2-hydroxy-1-(4-(methylsulfonyl)phenyl) ethylidene)-6-methylhexahydro-3a,6-methanoinden-3(2*H*)-one (**3li**)

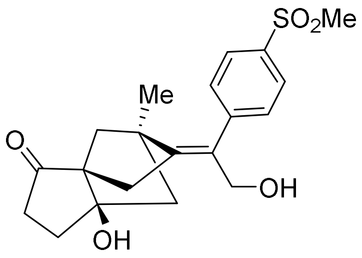

Chemical Formula: $C_{20}H_{24}O_5S$
Exact Mass: 376.1344

3li was prepared according to general procedure using **1l** (0.1 mmol, 22.0 mg) and **2i** and was purified by silica gel column chromatography (petroleum ether/ethyl acetate = 3/1~1/3) to obtain **3li** (21.8 mg, 58% yield). ^{1}H NMR (400 MHz, $CDCl_3$) δ 7.85 (d, *J* = 8.0 Hz, 2H), 7.35 (d, *J* = 8.0 Hz, 2H), 4.57-4.20 (bs, 1H), 4.40 (d, *J* = 11.2 Hz, 1H), 4.05 (d, *J* = 11.2 Hz, 1H), 3.09 (s, 3H), 2.77 (s, 2H), 2.64-2.45 (m, 2H), 2.35-2.25 (m, 1H), 2.16-1.97 (m, 3H), 1.85 (dd, *J* = 13.0, 3.4 Hz, 1H), 1.72 (d, *J* = 9.9 Hz, 1H), 1.59 (dd, *J* = 9.9, 3.2 Hz, 1H), 0.52 (s, 3H);

^{13}C NMR (101 MHz, $CDCl_3$) δ 217.5, 147.1, 146.4, 138.9, 129.8, 126.8, 124.9, 86.5, 65.0, 64.4, 54.3, 54.0, 52.1, 44.4, 38.1, 35.5, 33.1, 20.1;

HRMS: (ESI) calcd for $C_{20}H_{24}O_5SNa^+$[M+Na]$^+$ 399.1237; found 399.1227.

The enantiomeric purity was established by HPLC analysis using a chiral column: AD-H column, 30 °C, *n*-Hexane/*i*-Propanol = 70/30 as eluent, 254 nm, 1 mL/min. tR = 4.8 min (major), 7.0 min (minor).

Optical Rotation: $[\alpha]_D^{25}$ -101.4 (c 0.7, ^{i}PrOH) for 95% ee.

Absolute stereochemistry was determined through analogy with **3aa**.

〈色谱图〉

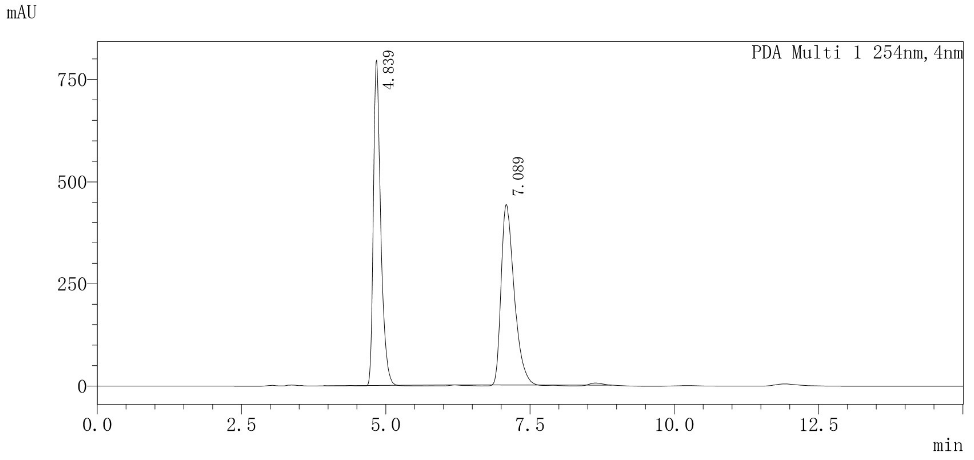

〈峰表〉

PDA Ch1 254nm

峰号	保留时间	面积	高度	浓度	浓度单位	标记	化合物名
1	4.839	6925502	795795	0.000		M	
2	7.089	6918692	442178	0.000		M	
总计		13844194	1237973				

peak number
retention time
area
height

Supplementary Figure 67. HPLC spectrum of 3li

〈色谱图〉

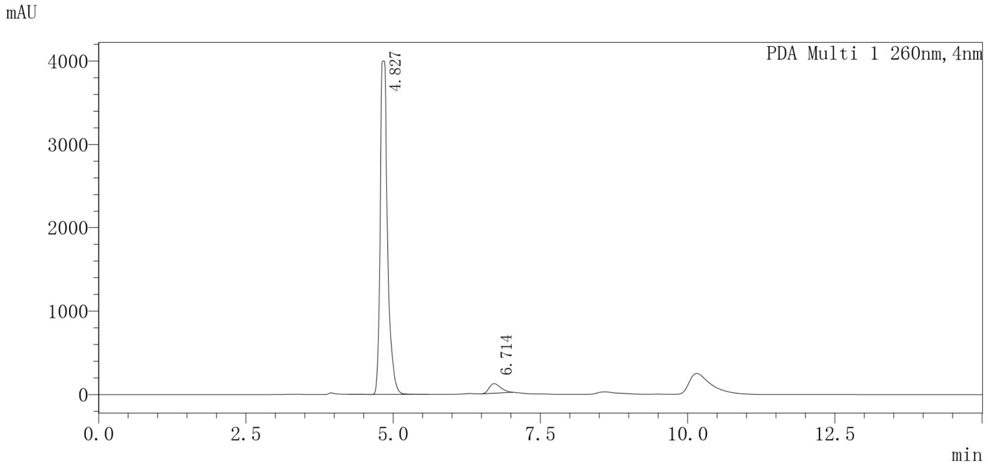

〈峰表〉

PDA Ch1 260nm

峰号	保留时间	面积	高度	浓度	浓度单位	标记	化合物名
1	4.827	34547968	3996646	95.627		M	
2	6.714	1579971	115803	4.373		M	
总计		36127939	4112449				

peak number
retention time
area
height

Supplementary Figure 68. HPLC spectrum of (*3aS,6R,7aR,Z*)-3li

($3aR,6R,7aR,Z$)-7a-hydroxy-5-(1-(4-(methylsulfonyl)phenyl)ethylidene)-6-phenylhexahydro-3a,6-methanoinden-3(2*H*)-one (**3mi**)

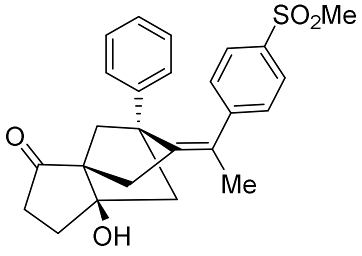

Chemical Formula: $C_{25}H_{26}O_4S$
Exact Mass: 422.1552

3mi was prepared according to general procedure using **1m** (0.1 mmol, 26.6 mg) and **2i** and was purified by silica gel column chromatography (petroleum ether/ethyl acetate = 5/1~1/1) to obtain **3mi** (23.2 mg, 55% yield). ^{1}H NMR (400 MHz, $CDCl_3$) δ 7.38 (d, J = 8.4 Hz, 2H), 7.13-6.50 (m, 7H), 2.96-2.82 (m, 2H), 2.92 (s, 3H), 2.76-2.64 (m, 2H), 2.57 (dd, J = 18.6, 8.4 Hz, 1H), 2.36 (dd, J = 14.0, 8.4 Hz, 1H), 2.25-2.10 (m, 4H), 1.97 (dd, J = 9.6, 3.2 Hz, 1H), 1.92 (s, 3H);

^{13}C NMR (101 MHz, $CDCl_3$) δ 216.6, 148.7, 141.8, 141.6, 137.1, 129.1, 128.6, 127.8, 127.5, 126.3, 125.7, 85.5, 64.2, 60.8, 52.4, 51.8, 44.4, 38.2, 36.2, 33.6, 22.9;

HRMS: (ESI) calcd for $C_{25}H_{27}O_4S^+$[M+H]$^+$ 423.1625; found 423.1609.

The enantiomeric purity was established by HPLC analysis using a chiral column: AD-H column, 30 °C, *n*-Hexane/*i*-Propanol = 70/30 as eluent, 254 nm, 1 mL/min. tR = 7.7 min (major), 9.3 min (minor).

Optical Rotation: $[\alpha]_D^{25}$ -25.9 (c 0.8, ^{i}PrOH) for 96% ee.

Absolute stereochemistry was determined through analogy with **3aa**.

〈色谱图〉

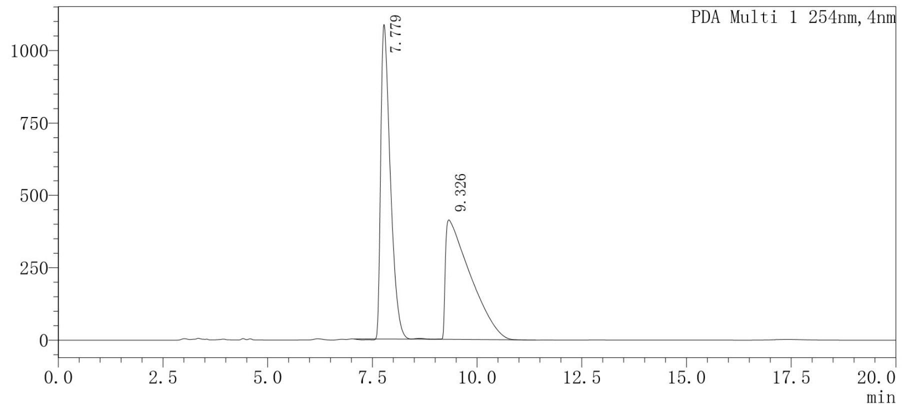

〈峰表〉

PDA Ch1 254nm

峰号	保留时间	面积	高度	浓度	浓度单位	标记	化合物名
1	7.779	17292439	1086148	0.000		M	
2	9.326	17080017	412110	0.000		M	
总计		34372455	1498257				

peak number　retention time　area　height

Supplementary Figure 69. HPLC spectrum of 3mi

〈色谱图〉

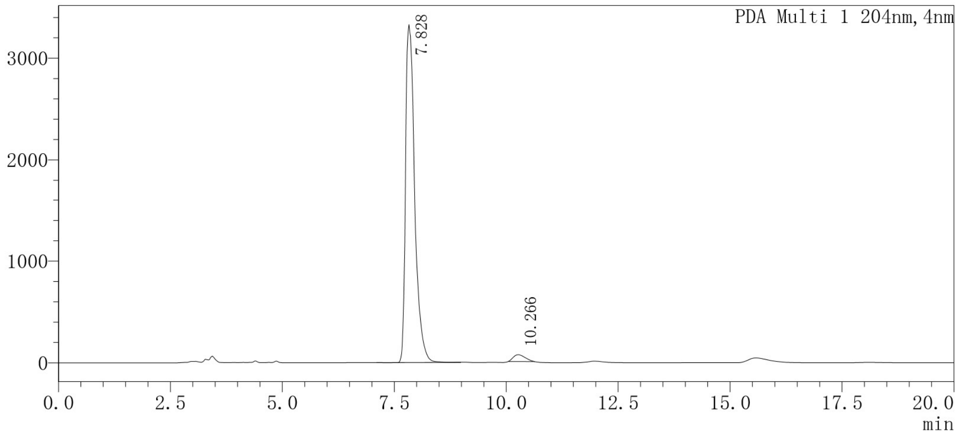

〈峰表〉

PDA Ch1 204nm

峰号	保留时间	面积	高度	浓度	浓度单位	标记	化合物名
1	7.828	47160067	3327781	0.000		M	
2	10.266	1243221	67405	0.000		M	
总计		48403288	3395186				

peak number　retention time　area　height

Supplementary Figure 70. HPLC spectrum of (*3aS,6R,7aR,Z*)-3mi

($3aR,6S,7aR,Z$)-7a-hydroxy-6-(methoxymethyl)-5-(1-(4-(methylsulfonyl)phenyl) ethylidene)hexahydro-3a,6-methanoinden-3(2*H*)-one (**3ni**)

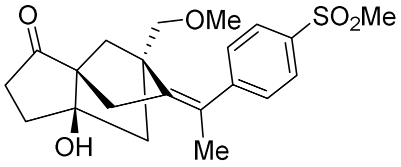

Chemical Formula: $C_{21}H_{26}O_5S$
Exact Mass: 390.1501

3ni was prepared according to general procedure using **1n** (0.1 mmol, 23.4 mg) and **2i** and was purified by silica gel column chromatography (petroleum ether/ethyl acetate = 5/1~1/2) to obtain **3ni** (27.3 mg, 70% yield). ^{1}H NMR (400 MHz, $CDCl_3$) δ 7.88 (d, *J* = 8.0 Hz, 2H), 7.36 (d, *J* = 8.0 Hz, 2H), 3.06 (s, 3H), 2.78 (s, 3H), 2.70 (s, 2H), 2.67-2.50 (m, 4H), 2.29-2.12 (m, 3H), 1.93 (s, 3H), 1.91-1.78 (m, 1H), 1.86 (dd, *J* = 10.0, 2.4 Hz, 1H), 1.74 (dd, *J* = 10.0, 3.6 Hz, 1H), 1.66 (dd, *J* = 12.4, 3.6 Hz, 1H);

^{13}C NMR (101 MHz, $CDCl_3$) δ 216.7, 150.1, 138.5, 137.9, 129.4, 127.1, 126.8, 85.8, 72.8, 63.9, 58.5, 58.2, 50.3, 48.2, 44.6, 38.0, 35.8, 33.5, 23.1;

HRMS: (ESI) calcd for $C_{21}H_{27}O_5S^+$[M+H]$^+$ 391.1574; found 391.1566.

The enantiomeric purity was established by HPLC analysis using a chiral column: AD-H column, 30 °C, *n*-Hexane/*i*-Propanol = 80/20 as eluent, 254 nm, 1 mL/min. tR = 9.8 min (minor), 10.3 min (major).

Optical Rotation: $[\alpha]_D^{25}$ -71.7 (c 0.9, ^{i}PrOH) for 98% ee.

Absolute stereochemistry was determined through analogy with **3aa**.

〈色谱图〉

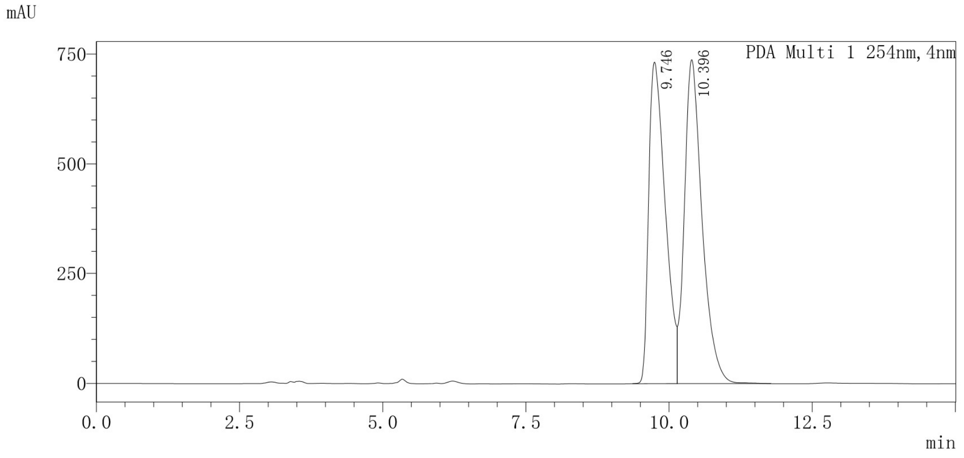

〈峰表〉

PDA Ch1 254nm

峰号	保留时间	面积	高度	浓度	浓度单位	标记	化合物名
1	9.746	14740784	732363	0.000		M	
2	10.396	15881443	737747	0.000		V M	
总计		30622226	1470110				

peak number　retention time　area　height

Supplementary Figure 71. HPLC spectrum of 3ni

〈色谱图〉

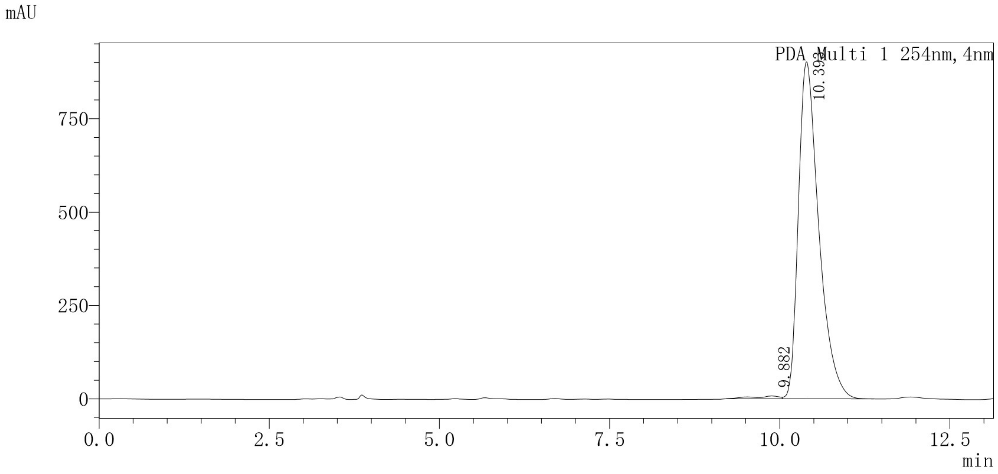

〈峰表〉

PDA Ch1 254nm

峰号	保留时间	面积	高度	浓度	浓度单位	标记	化合物名
1	9.882	206187	7581	0.000		M	
2	10.393	18832168	901487	0.000		V M	
总计		19038356	909068				

peak number　retention time　area　height

Supplementary Figure 72. HPLC spectrum of (*3aS,6R,7aR,Z*)-3ni

Methyl (*3aR,6S,7aR,Z*)-7a-hydroxy-5-(1-(4-(methylsulfonyl)phenyl)ethylidene)-3-oxohexahydro-3a,6-methanoindene-6(1*H*)-carboxylate (**3oi**)

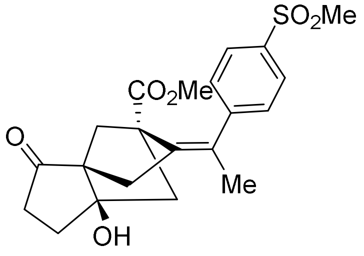

3oi was prepared according to general procedure using **1o** (0.1 mmol, 24.8 mg) and **2i** and was purified by silica gel column chromatography (petroleum ether/ethyl acetate = 5/1~1/1) to obtain **3oi** (20.2 mg, 50% yield). ^{1}H NMR (400 MHz, $CDCl_3$) δ 7.86 (d, *J* = 8.0 Hz, 2H), 7.29 (d, *J* = 8.0 Hz, 2H), 3.04 (s, 3H), 2.91 (s, 3H), 2.79-2.52 (m, 5H), 2.35-2.26 (m, 1H), 2.23-2.14 (m, 1H), 2.09-2.02 (m, 3H), 2.00 (s, 3H), 1.88 (bs, 1H);

^{13}C NMR (101 MHz, $CDCl_3$) δ 215.2, 172.1, 148.1, 138.7, 138.1, 129.0, 128.4, 127.1, 84.9, 64.9, 60.9, 51.2, 49.9, 49.3, 44.5, 38.1, 36.0, 32.3, 21.9;

HRMS: (ESI) calcd for $C_{21}H_{25}O_6S^+$[M+H]$^+$ 405.1366; found 405.1358.

The enantiomeric purity was established by HPLC analysis using a chiral column: AD-H column, 30 °C, *n*-Hexane/*i*-Propanol = 70/30 as eluent, 254 nm, 1 mL/min. tR = 7.4 min (minor), 8.6 min (major).

Optical Rotation: $[\alpha]_D^{25}$ 133.5 (c 0.5, ^{i}PrOH) for 99% ee.

Absolute stereochemistry was determined through analogy with **3aa**.

<色谱图>

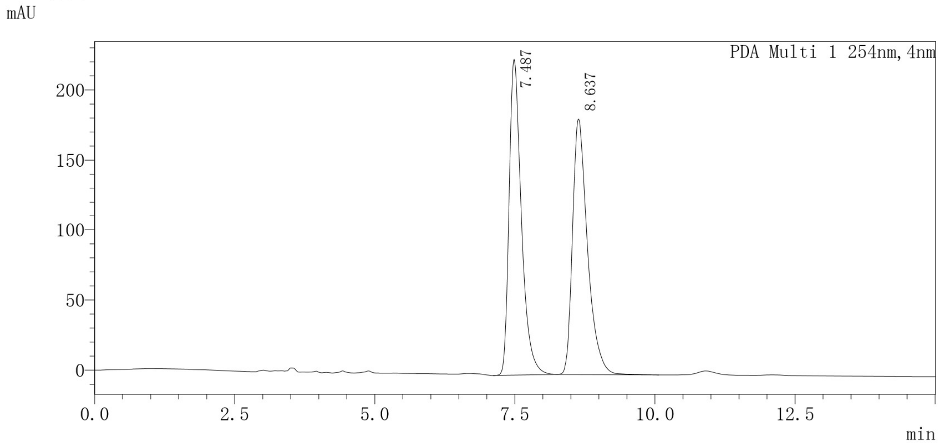

<峰表>

PDA Ch1 254nm

峰号	保留时间	面积	高度	浓度	浓度单位	标记	化合物名
1	7.487	3431987	225403	0.000		M	
2	8.637	3388139	182392	0.000		M	
总计		6820126	407795				

peak number
retention time
area
height

Supplementary Figure 73. HPLC spectrum of 3oi

<色谱图>

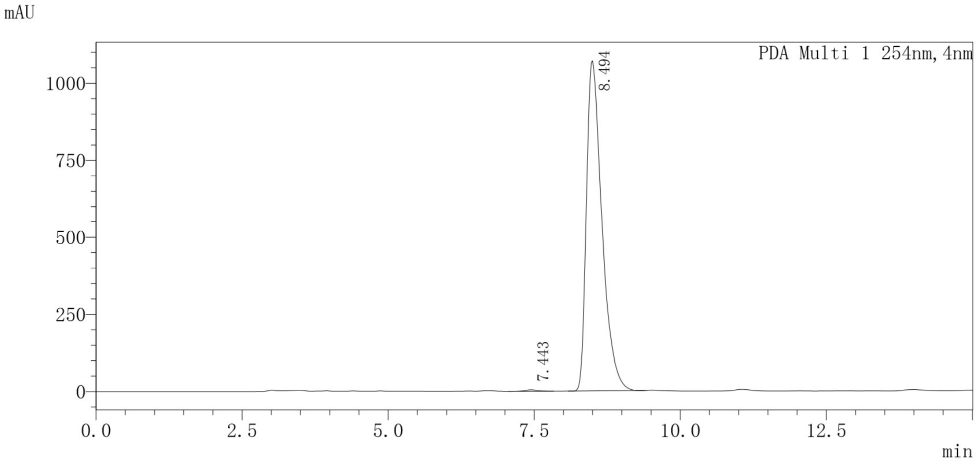

<峰表>

PDA Ch1 254nm

峰号	保留时间	面积	高度	浓度	浓度单位	标记	化合物名
1	7.443	68352	4704	0.000		M	
2	8.494	19758013	1070471	0.000		M	
总计		19826365	1075175				

peak number
retention time
area
height

Supplementary Figure 74. HPLC spectrum of (*3aS,6R,7aR,Z*)-3oi

1-((*1R,2R,4R,Z*)-2-hydroxy-2,4-dimethyl-5-(1-(4-(methylsulfonyl)phenyl)ethylidene)bicyclo[2.2.1]heptan-1-yl)ethan-1-one (**3pi**)

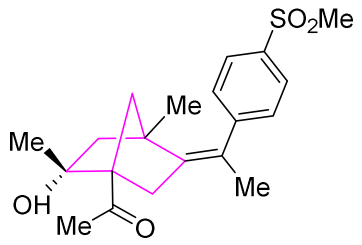

Chemical Formula: $C_{20}H_{26}O_4S$
Exact Mass: 362.1552

3pi was prepared according to general procedure using **1p** (0.1 mmol, 20.6 mg) and **2i** and was purified by silica gel column chromatography (petroleum ether/ethyl acetate = 10/1~2/1) to obtain **3pi** (18.4 mg, 51% yield). ^{1}H NMR (400 MHz, $CDCl_3$) δ 7.85 (d, *J* = 8.4 Hz, 2H), 7.32 (d, *J* = 8.4 Hz, 2H), 3.14-3.08 (m, 1H), 3.07 (s, 3H), 2.38 (d, *J* = 14.0 Hz, 1H), 2.23 (s, 3H), 1.89 (s, 3H), 1.81 (dd, *J* = 10.1, 3.2 Hz, 1H), 1.76-1.63 (m, 3H), 1.30 (s, 3H), 0.53 (s, 3H);

^{13}C NMR (101 MHz, $CDCl_3$) δ 213.0, 150.4, 141.1, 138.3, 129.6, 126.9, 124.5, 78.5, 63.7, 54.4, 49.3, 48.3, 44.6, 38.1, 29.0, 28.3, 22.8, 20.7;

HRMS: (ESI) calcd for $C_{20}H_{27}O_4S^+$[M+H]$^+$ 363.1625; found 363.1613.

The enantiomeric purity was established by HPLC analysis using a chiral column: AD-H column, 30 °C, *n*-Hexane/*i*-Propanol = 85/15 as eluent, 254 nm, 1 mL/min. tR = 10.5 min (major), 11.7 min (minor).

Optical Rotation: $[\alpha]_D^{25}$ -25.8 (c 0.5, ^{i}PrOH) for 99% ee.

Absolute stereochemistry was determined through analogy with **3aa**.

〈色谱图〉

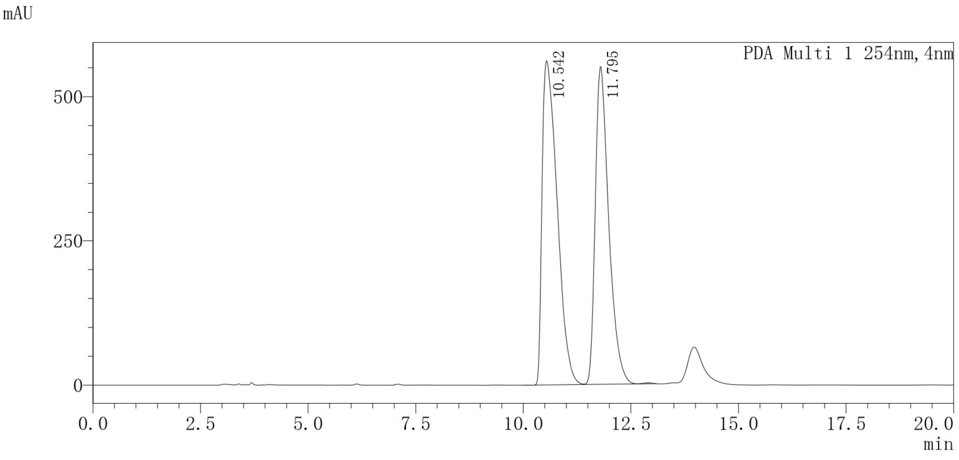

〈峰表〉

PDA Ch1 254nm

峰号	保留时间	面积	高度	浓度	浓度单位	标记	化合物名
1	10.542	13645584	561690	0.000		M	
2	11.795	12020693	550743	0.000		V M	
总计		25666277	1112433				

peak number
area
height
retention time

Supplementary Figure 75. HPLC spectrum of 3pi

〈色谱图〉

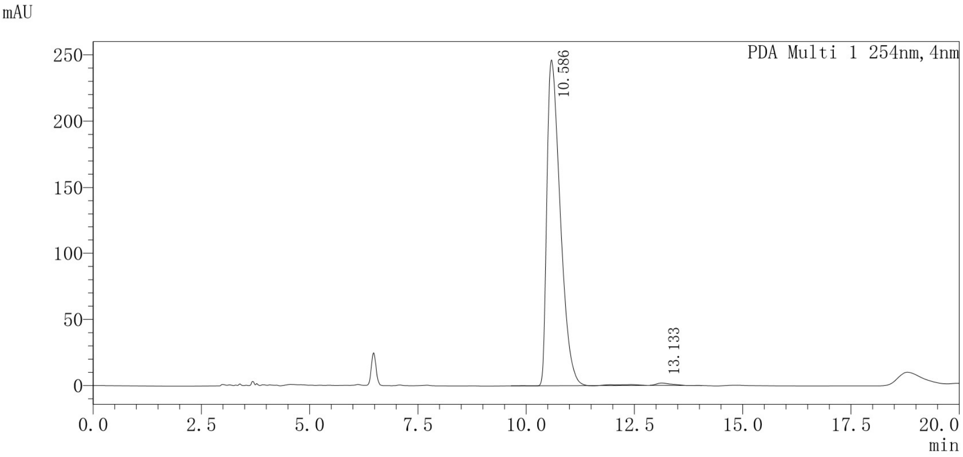

〈峰表〉

PDA Ch1 254nm

峰号	保留时间	面积	高度	浓度	浓度单位	标记	化合物名
1	10.586	5321838	246344	0.000		M	
2	13.133	42319	1797	0.000		M	
总计		5364157	248141				

peak number
area
height
retention time

Supplementary Figure 76. HPLC spectrum of (*3aS,6R,7aR,Z*)-3pi

(*Z*)-7a-hydroxy-7-methylene-5-(1-(4-(methylsulfonyl)phenyl)ethylidene) hexahydro-3a,6-methanoinden-3(2*H*)-one (**3qi**)

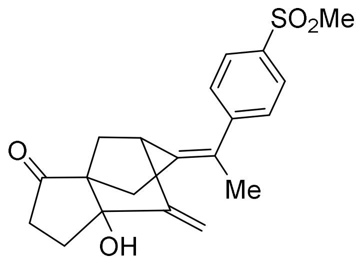

Chemical Formula: $C_{20}H_{22}O_4S$
Exact Mass: 358.1239

3qi was prepared according to general procedure using **1q** (0.1 mmol, 20.2 mg) and **2i** and was purified by silica gel column chromatography (petroleum ether/ethyl acetate = 10/1~2/1) to obtain **3qi** (12.5 mg, 35% yield). ^{1}H NMR (400 MHz, $CDCl_3$) δ 7.95-7.89 (m, 2H), 7.45-7.39 (m, 2H), 5.15 (s, 1H), 4.99 (s, 1H), 3.36 (s, 1H), 3.08 (s, 3H), 2.75-2.55 (m, 4H), 2.37-2.31 (m, 1H), 2.23-2.14 (m, 1H), 1.99 (s, 3H), 1.97 (s, 1H), 1.88-1.83 (m, 1H), 1.69-1.67 (m, 1H);

^{13}C NMR (151 MHz, $CDCl_3$) δ 215.9, 158.1, 149.1, 139.1, 138.3, 128.8, 127.3, 105.8, 86.1, 65.3, 53.6, 44.5, 43.4, 38.1, 33.4, 29.8, 20.2;

HRMS: (ESI) calcd for $C_{20}H_{23}O_4S^+$[M+H]$^+$ 358.1239; found. 358.1228.

(*3S,3aR,6R,7aR,Z*)-6-methyl-5-(1-phenylethylidene)hexahydro-3a,6-methanoindene-3,7*a*(1*H*)-diol (**5**)

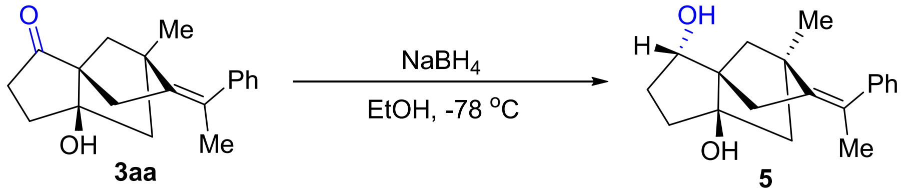

Supplementary Figure 77. Derivatization 1. Reduction of ketone

To a solution of **3aa** (28.2 mg, 0.1 mmol) in EtOH (2 mL) were added $NaBH_4$ (10.0 mg, 0.3 mmol) at −78 °C. The reaction mixture was stirred for 2 hours at this temperature. Then, the reaction mixture was quenched with saturated NH_4Cl aqueous solution and extracted with EtOAc. The combined organic layers were washed with brine, dried over anhydrous Na_2SO_4 and filtered. The solvent was concentrated under reduced pressure and purified by silica-gel column chromatography, eluting with petroleum ether/ethyl acetate 3/1~1/1 (v/v) to provide the alcohol **5** (26.4 mg, 93%, d.r. > 20/1).

^{1}H NMR (400 MHz, $CDCl_3$) δ 7.30-7.17 (m, 3H), 7.15-7.08 (m, 2H), 4.32 (t, *J* = 8.0 Hz, 1H), 2.58-2.45 (m, 2H), 2.42-2.28 (m, 1H), 2.09-1.94 (m, 2H), 1.93 (s, 3H), 1.91-1.73 (m, 2H), 1.71 (dd, *J* = 12.6, 3.8 Hz, 1H), 1.59-1.47 (m, 3H), 1.38 (dd, *J* = 9.7, 2.9 Hz, 1H), 0.57 (s, 3H);

^{13}C NMR (101 MHz, $CDCl_3$) δ 144.6, 140.3, 128.5, 127.8, 127.6, 126.1, 87.5, 72.8, 61.7, 57.2, 51.5, 48.3, 38.1, 35.0, 33.2, 23.3, 20.8;

HRMS: (ESI) calcd for $C_{19}H_{24}O_2Na^+$[M+Na]$^+$ 307.1669; found 307.1670.

Optical Rotation: $[\alpha]_D^{20}$ -264.6 (c 0.7, ^{i}PrOH).

(*3S,3aR,6R,Z*)-3-((4-methoxyphenyl)amino)-6-methyl-5-(1-phenylethylidene) hexahydro-3a,6-methanoinden-7a(1*H*)-ol (**6**)

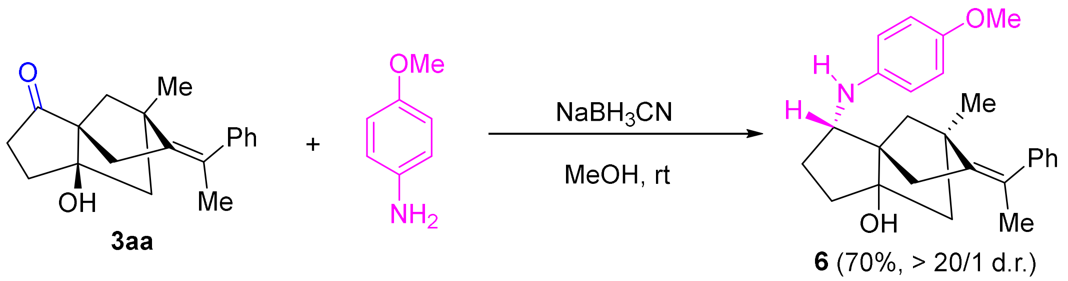

Supplementary Figure 78. Derivatization 2. Reduction amination of ketone

To a solution of **3aa** (28.2 mg, 0.1 mmol) in MeOH (2 mL) was added 4-methoxyaniline (36.9 mg, 0.3 mmol) at room temperature. The reaction mixture was stirred for 2 hours and then $NaBH_3CN$ (20.0 mg, 0.3 mmol) was added. Then the mixture was stirred at room temperature until the reaction was complete (monitored by TLC). The reaction mixture was quenched with saturated NH_4Cl aqueous solution and extracted with EtOAc. The combined organic layers were washed with brine, dried over anhydrous Na_2SO_4 and filtered. The solvent was concentrated under reduced pressure and purified by silica-gel column chromatography, eluting with petroleum ether/ethyl acetate 3/1~1/1 (v/v) to provide the amine **6** (27.2 mg, 70%, d.r. > 20/1). ^{1}H NMR (400 MHz, $CDCl_3$) δ 7.27 (d, *J* = 6.6 Hz, 1H), 7.26-7.17 (m, 2H), 7.13-7.08 (m, 2H), 6.82-6.75 (m, 2H), 6.64 (d, *J* = 8.4 Hz, 2H), 3.92 (t, *J* = 8.1 Hz, 1H), 3.76 (s, 3H), 2.59-2.44 (m, 3H), 2.03-1.93 (m, 3H), 1.89 (t, *J* = 1.4 Hz, 3H), 1.74 (dd, *J* = 12.7, 3.6 Hz, 1H), 1.61 (dd, *J* = 11.2, 5.4 Hz, 1H), 1.54 (dd, *J* = 9.4, 3.6 Hz, 1H), 1.38 (dd, *J* = 9.4, 3.2 Hz, 1H), 0.57 (s, 3H);

^{13}C NMR (151 MHz, $CDCl_3$) δ 144.5, 140.1, 128.5, 127.8, 127.6, 126.1, 114.9, 114.5, 87.4, 61.2, 56.8, 55.8, 54.5, 51.5, 48.2, 38.7, 35.7, 31.8, 23.3, 20.8;

HRMS: (ESI) calcd for $C_{26}H_{32}NO_2^+$[M+H]$^+$ 390.2428; found. 390.2424.

(*3R,3aR,6R,7aR,Z*)-3-allyl-6-methyl-5-(1-phenylethylidene)hexahydro-3a,6-methanoindene-3,7*a*(1*H*)-diol (**7**)

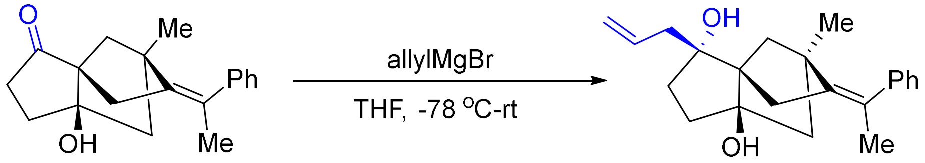

3aa **7**

Supplementary Figure 79. Derivatization 3. Allylation of ketone

To a solution of **3aa** (28.2 mg, 0.1 mmol) in anhydrous THF (2 mL) was added allylmagnesium bromide (1 mol/L in Et_2O, 0.3 mL, 0.3 mmol) at −78 °C. The reaction mixture was allowed to warm up to room temperature and was stirred for 24 hours. Then, the reaction mixture was quenched with saturated NH_4Cl aqueous solution and extracted with EtOAc. The combined organic layers were washed with brine, dried over anhydrous Na_2SO_4 and filtered. The solvent was concentrated under reduced pressure and purified by silica-gel column chromatography, eluting with petroleum ether/ethyl acetate 3/1~1/1 (v/v) to provide the allyl alcohol **7** (21.7 mg, 67%, d.r. > 20/1).

^{1}H NMR (400 MHz, $CDCl_3$) δ 7.32-7.23 (m, 2H), 7.21-7.16 (m, 1H), 7.15-7.10 (m, 2H), 6.03-5.88 (m, 1H), 5.21 (s, 1H), 5.20-5.13 (m, 1H), 2.80 (bs, 1H), 2.77-2.68 (m, 1H), 2.49-2.32 (m, 2H), 2.30-2.10 (m, 3H), 2.06-1.94 (m, 3H), 1.93 (s, 3H), 1.89-1.76 (m, 2H), 1.52 (dd, *J* = 9.5, 2.5 Hz, 1H), 1.15 (dd, *J* = 9.5, 3.7 Hz, 1H), 0.55 (s, 3H);

^{13}C NMR (101 MHz, $CDCl_3$) δ 144.7, 140.1, 133.6, 128.6, 127.6, 127.2, 126.0, 119.0, 89.2, 81.6, 63.3, 55.3, 51.3, 51.1, 41.7, 40.0, 37.9, 32.5, 23.2, 20.7;

HRMS: (ESI) calcd for $C_{22}H_{28}O_2Na^+[M+Na]^+$ 347.1982; found 347.1973.

Optical Rotation: $[\alpha]_D^{20}$ -220.6 (c 0.5, ^{i}PrOH).

(*3aR,6R,7aR,Z*)-6-methyl-3-methylene-5-(1-phenylethylidene)hexahydro-3a,6-methanoinden-7a(1*H*)-ol (**8**)

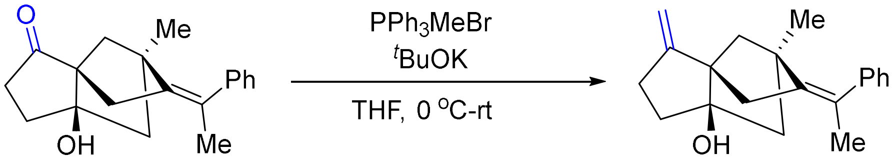

3aa **8**

Supplementary Figure 80. Derivatization 4. Vinylation of ketone

To a stirred solution of methyltriphenylphosphonium bromide (168.6 g, 0.3 mmol) in THF was added ^{t}BuOK (1.0 M in THF, 0.3 mL, 0.3 mmol) at 0 °C. After stirred for 40 min at the same temperature, a solution of **3aa** (28.2 mg, 0.1 mmol) in THF (2 mL) was added to the mixture at 0 °C. After stirred for 12 hours at the same temperature, the reaction was quenched with saturated aqueous $NaHCO_3$. The aqueous phase was extracted with hexane and the combined organic extracts were dried over Na_2SO_4 and filtered. The solvent was concentrated under reduced pressure and purified by silica-gel column chromatography, eluting with petroleum ether/ethyl acetate 10/1~2/1 (v/v) to provide the alkene **8** (25.2 mg, 90%, d.r. > 20/1).

^{1}H NMR (400 MHz, $CDCl_3$) δ 7.30-7.23 (m, 2H), 7.22-7.17 (m, 1H), 7.16-7.10 (m, 2H), 5.02-4.99 (m, 1H), 4.98-4.96 (m, 1H), 2.78-2.62 (m, 2H), 2.58 (s, 2H), 2.02-1.90 (m, 1H), 1.96 (s, 3H), 1.92 (s, 1H), 1.89 (s, 1H), 1.79-1.71 (m, 1H), 1.54-1.45 (m, 3H), 0.54 (s, 3H);

^{13}C NMR (101 MHz, $CDCl_3$) δ 151.6, 144.7, 140.3, 128.6, 127.6, 127.5, 126.0, 106.1, 88.8, 61.2, 56.4, 54.5, 53.1, 38.7, 36.2, 32.0, 23.2, 20.6;

HRMS: (ESI) calcd for $C_{20}H_{25}O^+[M+H]^+$ 281.1900; found 281.1898.

Optical Rotation: $[\alpha]_D^{20}$ -234.6 (c 0.6, ^{i}PrOH).

(*4aS,6R,8aS,Z*)-4a-hydroxy-6-methyl-7-(1-phenylethylidene)hexahydro-1*H*,3*H*-6,8a-methanoisochromen-1-one (**9**)

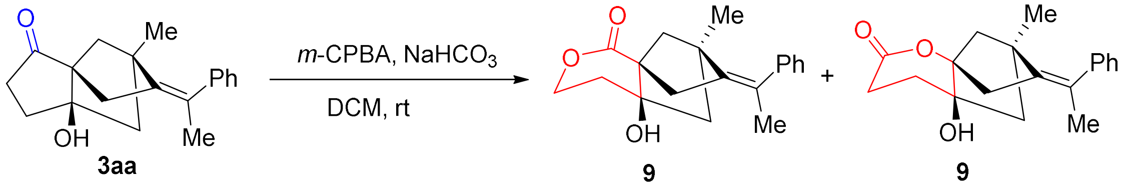

Supplementary Figure 81. Derivatization 5. Baeyer-Villiger oxidation

To a solution of **3aa** (28.2 mg, 0.1 mmol) and $NaHCO_3$ (33.6 mg, 0.4 mmol, 4 equiv) in DCM (3 mL) was added *m*-CPBA (29.3 mg, 0.2 mmol, 2 equiv). The reaction mixture was stirred at room temperature for 1 hour. The reaction was then quenched with water (5 mL). The aqueous phase was extracted with DCM and the combined organic extracts were dried over Na_2SO_4 and filtered. The solvent was concentrated under reduced pressure and purified by silica-gel column chromatography, eluting with petroleum ether/ethyl acetate 20/1~4/1 (v/v) to provide the tricyclic lactones **9** and **9’** (25.9 mg, 87% yield, **9**/**9’** = 3/1).

^{1}H NMR (400 MHz, acetone-D_6) δ 7.43-7.38 (m, 2.6H), 7.35-7.29 (m, 2.6H), 7.28-7.22 (m, 1.3H), 4.60 (ddd, *J* = 12.9, 11.4, 2.8 Hz, 1H), 4.56 (s, 1H), 4.26 (ddd, *J* = 11.3, 5.1, 1.7 Hz, 1H), 3.08 (dd, *J* = 13.4, 3.3 Hz, 0.3H), 2.84 (dd, *J* = 13.9, 3.4 Hz, 1H), 2.85 (s, 3H), 2.82-2.68 (m, 0.3H), 2.38 (ddd, *J* = 17.7, 5.3, 1.8 Hz, 0.3H), 2.30-2.13 (m, 2.6H), 2.03-1.75 (m, 5H), 1.62-1.56 (m, 1.2H), 1.55 (s, 3H), 1.54 (s, 1H), 0.26 (s, 3H), 0.21 (s, 1H);

^{13}C NMR (101 MHz, acetone-D_6) δ 172.6, 169.0, 141.6, 141.4, 127.8, 127.0, 126.9, 126.6, 89.0, 74.4, 74.3, 73.8, 70.0, 66.0, 64.7, 54.4, 52.2, 49.6, 49.0, 47.7, 47.4, 43.4, 37.6, 36.2, 35.7, 26.0, 24.5, 16.1;

HRMS: (ESI) calcd for $C_{19}H_{23}O_3^+$[M+H]$^+$ 299.1642; found 299.1634.

(*4aR,6R,8aS,Z*)-4a-hydroxy-6-methyl-7-(1-phenylethylidene)hexahydro-1*H*-6,8a-methanoquinolin-2(3*H*)-one (**10**)

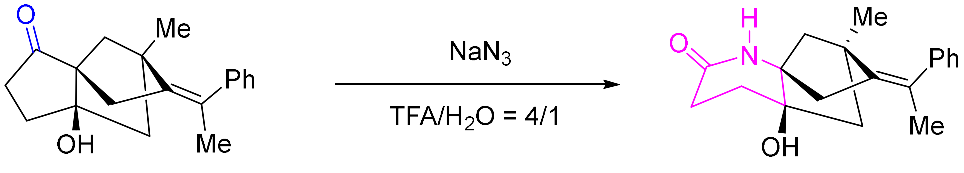

Supplementary Figure 82. Derivatization 6. Schmidt reaction with NaN_3

To a solution of **3aa** (28.2 mg, 0.1 mmol) in THF/H_2O (4/1) (3 mL) was added NaN_3 (13.0 mg, 0.2 mmol). The reaction mixture was stirred at 70 °C until the reaction was complete (monitored by TLC). The reaction was then quenched with water (5 mL). The aqueous phase was extracted with DCM and the combined organic extracts were dried over Na_2SO_4 and filtered. The solvent was concentrated under reduced pressure and purified by silica-gel column chromatography, eluting with petroleum ether/ethyl acetate 10/1~2/1 (v/v) to provide the tricyclic lactam **10** (23.8 mg, 80% yield, d.r. > 20/1). ^{1}H NMR (400 MHz, $CDCl_3$) δ 7.29-7.27 (m, 1H), 7.26-7.18 (m, 2H), 7.11-7.07 (m, 2H), 6.51 (s, 1H), 2.95 (dd, *J* = 15.4, 2.7 Hz, 1H), 2.75-2.61 (m, 1H), 2.32 (dt, *J* = 17.6, 3.7 Hz, 2H), 2.05-1.98 (m, 3H), 1.90 (s, 3H), 1.82-1.69 (m, 3H), 1.53 (dd, *J* = 9.5, 3.5 Hz, 1H), 0.51 (s, 3H);

^{13}C NMR (151 MHz, $CDCl_3$) δ 172.5, 144.0, 137.6, 128.4, 128.0, 127.7, 126.3, 73.5, 65.6, 54.4, 54.2, 47.7, 39.7, 33.2, 27.5, 23.3, 20.3;

HRMS: (ESI) calcd for $C_{19}H_{24}NO_2^+$[M+H]$^+$ 298.1802; found.298.1786.

(*3aS,6R,7aR*)-7a-hydroxy-6-methyltetrahydro-3a,6-methanoindene-3,5(2*H*,4*H*)-dione (**11**)

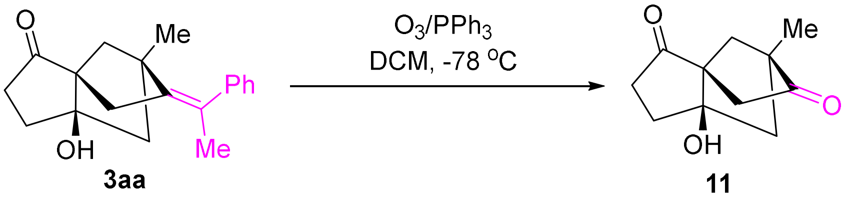

Supplementary Figure 83. Derivatization 7. Ozonolysis with O_3

To a solution of **3aa** (28.2 mg, 0.1 mmol) in CH_2Cl_2 (3 mL), O_3 was bubbled at -78 °C until the reaction was complete (monitored by TLC). Argon was bubbled into the solution for 5 min to remove the excess O_3. PPh_3 was added at -78 °C and the mixture was kept stirring for another 1 hour. The reaction mixture was passed through a short pad of silica gel, and eluted with EtOAc. The filtrate was concentrated and the residue was purified by flash column chromatography on silica gel (petroleum ether/ethyl acetate = 10/1 to 2/1) to afford the ketone **11** (18.6 mg, 96% yield).

^{1}H NMR (400 MHz, $CDCl_3$) δ 2.76-2.55 (m, 3H), 2.47 (d, *J* = 17.8 Hz, 1H), 2.36-2.21 (m, 2H), 2.15 (d, *J* = 13.4 Hz, 1H), 2.10 (dd, *J* = 10.6, 4.6 Hz, 1H), 2.07-1.92 (bs, 1H), 1.75 (dd, *J* = 13.4, 3.4 Hz, 1H), 1.68 (dd, *J* = 10.5, 3.4 Hz, 1H), 1.14 (s, 3H);

^{13}C NMR (101 MHz, $CDCl_3$) δ 215.4, 214.9, 84.8, 63.7, 59.3, 50.0, 47.7, 39.5, 38.1, 36.4, 14.5;

HRMS: (ESI) calcd for $C_{11}H_{15}O_3^+$[M+H]$^+$ 195.1016; found 195.1013.

Optical Rotation: $[\alpha]_D^{19}$ -7.8 (c 0.4, ^{i}PrOH).

Scheme 4b

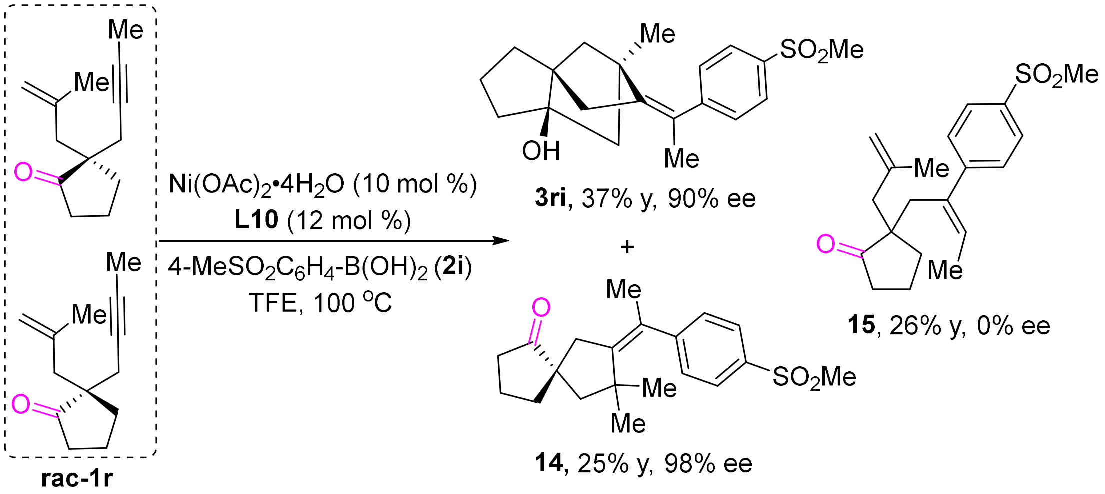

Supplementary Figure 84. Reaction with monocarbonyl substrate

Experimental procedure: An oven-dried sealed tube equipped with a PTFE-coated stir bar was charged with $Ni(OAc)_2 \cdot 4H_2O$ (0.01 mmol, 2.5 mg, 10 mol%), *1R,1'R,2S,2'S*-Duanphos (**L10**) (0.012 mmol, 4.6 mg, 12 mol%) and TFE (1 mL). This reaction mixture was stirred at room temperature for 15 minutes in an argon-filled glovebox. Substrate **1r** (0.1 mmol, 19.0 mg) and **2i** (0.2 mmol, 40.0 mg) was then added. The sealed tube was sealed and removed from the glovebox. Then the mixture was stirred at 100 °C until the reaction was complete (monitored by TLC). The resulting mixture was concentrated under reduced pressure and purified by column chromatography on silica gel, eluting with petroleum ether/ethyl acetate 5/1~1/1 (v/v) to afford the corresponding product **3ri** (12.8 mg, 37% yield, 90% ee), **14** (8.7 mg, 25% yield, 98% ee) and **15** (9.0 mg, 26% yield, 0% ee).

(3aR,6R,7aR,Z)-6-methyl-5-(1-(4-(methylsulfonyl)phenyl)ethylidene)hexahydro-3a,6-methanoinden-7a(1H)-ol (**3ri**)

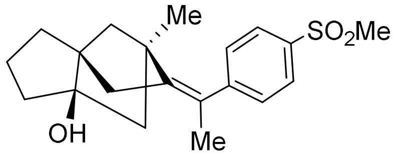

Chemical Formula: $C_{20}H_{26}O_3S$
Exact Mass: 346.1603

^{1}H NMR (400 MHz, $CDCl_3$) δ 7.83 (d, *J* = 8.5 Hz, 2H), 7.33 (d, *J* = 8.5 Hz, 2H), 3.06 (s, 3H), 2.59 (dd, *J* = 15.7, 3.0 Hz, 1H), 2.33 (d, *J* = 15.7 Hz, 1H), 1.96-1.85 (m, 8H), 1.75-1.62 (m, 3H), 1.40-1.33 (m, 2H), 0.49 (s, 3H);

^{13}C NMR (151 MHz, $CDCl_3$) δ 151.0, 144.0, 138.0, 129.6, 126.8, 124.9, 88.3, 57.7, 54.7, 53.9, 51.8, 44.6, 41.2, 37.5, 27.0, 23.9, 22.9, 21.1;

HRMS: (ESI) calcd for $C_{20}H_{27}O_3S^+$[M+H]$^+$ 347.1675; found. 347.1668.

The enantiomeric purity was established by HPLC analysis using a chiral column: AD-H column, 30 °C, *n*-Hexane/*i*-Propanol = 85/15 as eluent, 254 nm, 1 mL/min. tR = 8.7 min (major), 13.2 min (minor).

Optical Rotation: $[\alpha]_D^{11}$ -94.5 (c 0.4, ^{i}PrOH) for 99% ee.

Absolute stereochemistry was determined through analogy with **3aa**.

<色谱图>

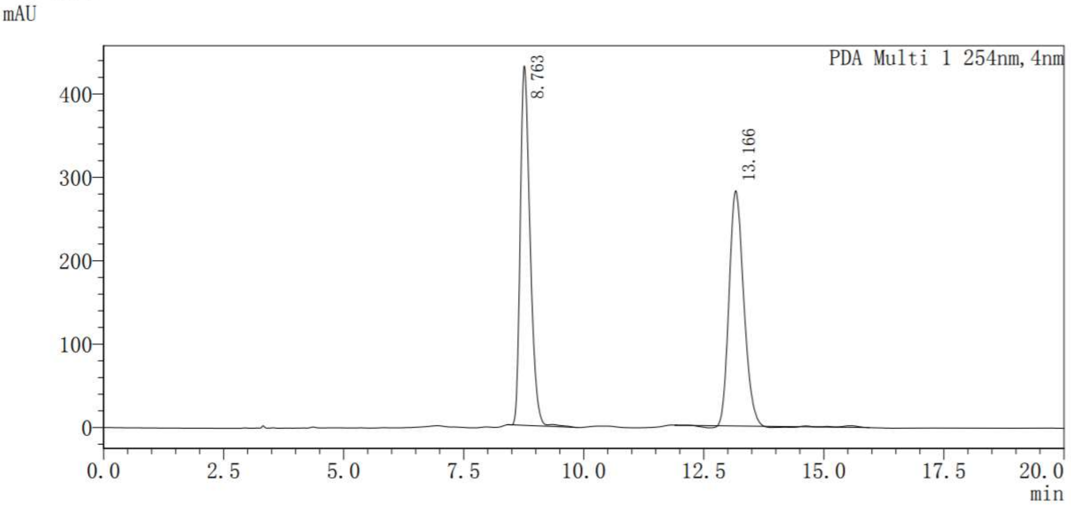

<峰表>

PDA Ch1 254nm

峰号	保留时间	面积	高度	浓度	浓度单位	标记	化合物名
1	8.763	6133123	431019	0.000		M	
2	13.166	5977208	282106	0.000		M	
总计		12110331	713125				

Supplementary Figure 85. HPLC spectrum of 3ri

<色谱图>

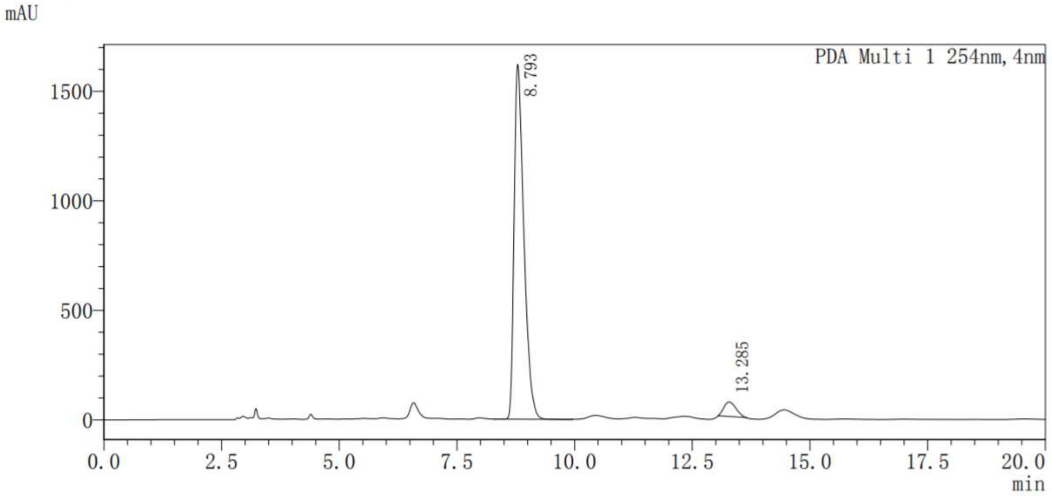

<峰表>

PDA Ch1 254nm

峰号	保留时间	面积	高度	浓度	浓度单位	标记	化合物名
1	8.793	23329226	1620002	0.000		M	
2	13.285	1195279	66489	0.000		M	
总计		24524505	1686491				

Supplementary Figure 86. HPLC spectrum of (3aR,6R,7aR,Z)-3ri

(S,Z)-7,7-dimethyl-8-(1-(4-(methylsulfonyl)phenyl)ethylidene)spiro[4.4]nonan-1-one (**14**)

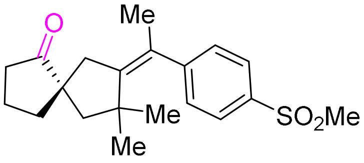

Chemical Formula: $C_{20}H_{26}O_3S$
Exact Mass: 346.1603

^{1}H NMR (400 MHz, $CDCl_3$) δ 7.91-7.82 (m, 2H), 7.35-7.29 (m, 2H), 3.08 (s, 3H), 2.73 (dd, *J* = 16.7, 1.6 Hz, 1H), 2.50 (d, *J* = 16.7 Hz, 1H), 2.32-2.19 (m, 2H), 2.13-1.88 (m, 5H), 1.86-1.82 (m, 3H), 1.45 (dd, *J* = 13.0, 1.7 Hz, 1H), 0.96 (s, 3H), 0.77 (s, 3H);

^{13}C NMR (151 MHz, $CDCl_3$) δ 222.6, 149.7, 144.5, 137.2, 128.9, 126.2, 125.9, 52.8, 52.1, 43.6, 42.5, 42.4, 37.6, 36.4, 29.6, 29.0, 23.5, 18.5;

HRMS: (ESI) calcd for $C_{20}H_{27}O_3S^+$[M+H]$^+$ 347.1675; found. 347.1671.

The enantiomeric purity was established by HPLC analysis using a chiral column: AD-H column, 30 °C, *n*-Hexane/*i*-Propanol = 80/20 as eluent, 254 nm, 1 mL/min. tR = 6.5 min (minor), 7.4 min (major).

Optical Rotation: $[\alpha]_D^{13}$ 10.7 (c 0.1, ^{i}PrOH) for 98% ee.

Absolute stereochemistry was determined through analogy with **3aa**.

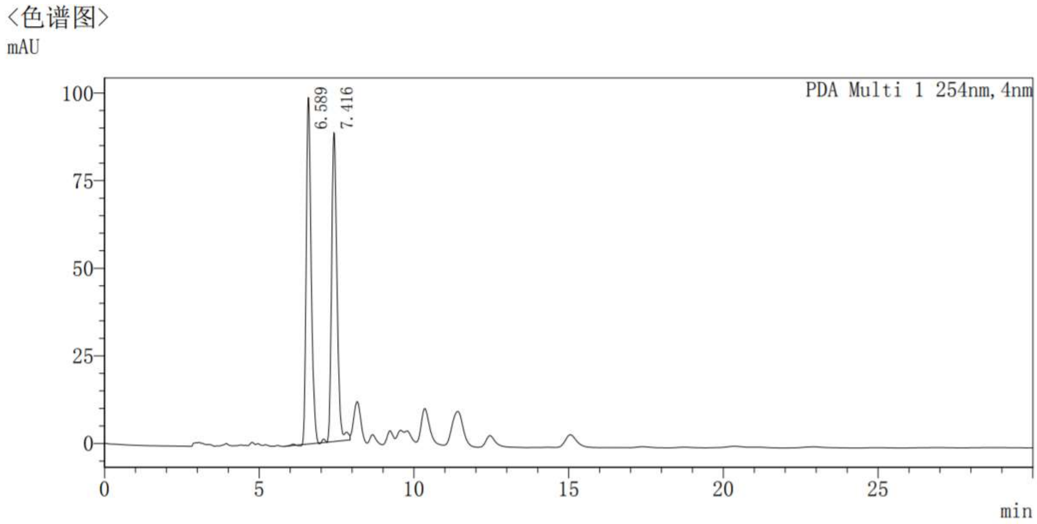

〈峰表〉
PDA Ch1 254nm

峰号	保留时间	面积	高度	浓度	浓度单位	标记	化合物名
1	6.589	1104527	98854	0.000		M	
2	7.416	1072835	88151	0.000		M	
总计		2177362	187005				

Supplementary Figure 87. HPLC spectrum of 14

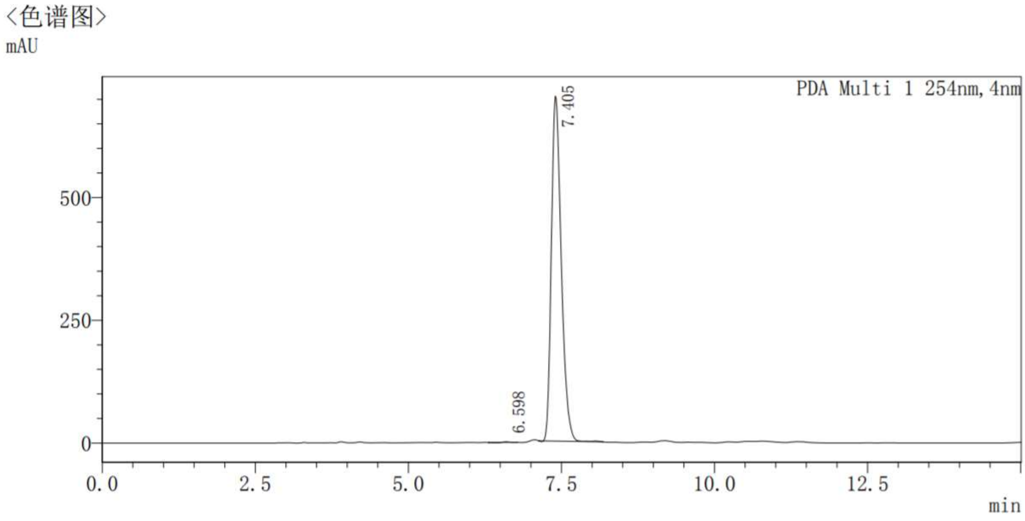

〈峰表〉
PDA Ch1 254nm

峰号	保留时间	面积	高度	浓度	浓度单位	标记	化合物名
1	6.598	5977	1255	0.000		M	
2	7.405	7876528	702489	0.000		M	
总计		7882504	703744				

Supplementary Figure 88. HPLC spectrum of (S,Z)-14

(*E*)-2-(2-methylallyl)-2-(2-(4-(methylsulfonyl)phenyl)but-2-en-1-yl)cyclopentan-1-one (**15**)

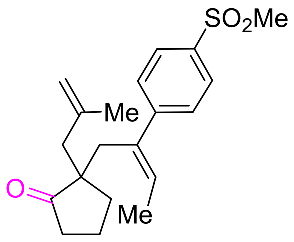

Chemical Formula: $C_{20}H_{26}O_3S$
Exact Mass: 346.1603

^{1}H NMR (400 MHz, $CDCl_3$) δ 7.87-7.82 (m, 2H), 7.48-7.43 (m, 2H), 5.86 (d, *J* = 7.0 Hz, 1H), 4.86-4.79 (m, 1H), 4.64 (s, 1H), 3.06 (s, 3H), 2.74 (s, 2H), 2.24 (d, *J* = 13.4 Hz, 1H), 2.15-2.01 (m, 2H), 1.93 (d, *J* = 7.0 Hz, 1H), 1.82 (d, *J* = 7.0 Hz, 3H), 1.80-1.63 (m, 4H), 1.61 (s, 3H);

^{13}C NMR (151 MHz, $CDCl_3$) δ 222.7, 150.2, 142.0, 138.4, 136.9, 131.4, 127.6, 127.3, 115.6, 52.8, 44.6, 44.6, 38.2, 35.0, 31.3, 24.4, 18.9, 15.2;

HRMS: (ESI) calcd for $C_{20}H_{27}O_3S^+$[M+H]$^+$ 347.1675; found. 347.1671.

Supplementary Table 2. Crystal data and structure refinement for 3aa.

Identification code	mo_190918e_0m	
Empirical formula	C19 H22 O2	
Formula weight	282.36	
Temperature	273.15 K	
Wavelength	0.71073 Å	
Crystal system	Orthorhombic	
Space group	$P2_12_12_1$	
Unit cell dimensions	a = 7.0530(19) Å	= 90°.
	b = 9.622(3) Å	= 90°.
	c = 22.688(6) Å	= 90°.
Volume	1539.7(7) $Å^3$	
Z	4	
Density (calculated)	1.218 Mg/m^3	
Absorption coefficient	0.077 mm^{-1}	
F(000)	608	
Crystal size	0.12 x 0.1 x 0.1 mm^3	
Theta range for data collection	1.795 to 32.031°.	
Index ranges	-10<=h<=10, -13<=k<=13, -32<=l<=29	
Reflections collected	16542	
Independent reflections	4947 [R(int) = 0.0339]	
Completeness to theta = 25.242°	99.8 %	
Absorption correction	Semi-empirical from equivalents	
Max. and min. transmission	0.7463 and 0.7254	
Refinement method	Full-matrix least-squares on F^2	
Data / restraints / parameters	4947 / 0 / 193	
Goodness-of-fit on F^2	1.040	
Final R indices [I>2sigma(I)]	R1 = 0.0399, wR2 = 0.1056	
R indices (all data)	R1 = 0.0484, wR2 = 0.1114	
Absolute structure parameter	0.2(4)	
Extinction coefficient	n/a	
Largest diff. peak and hole	0.224 and -0.239 e.$Å^{-3}$	

Supplementary Table 3. Atomic coordinates (x 10^4) and equivalent isotropic displacement parameters ($Å^2$x 10^3) for 3aa.

U(eq) is defined as one third of the trace of the orthogonalized U^{ij} tensor.

	x	y	z	U(eq)
C(1)	2852(2)	4738(2)	5437(1)	28(1)
C(2)	3100(3)	3884(2)	4946(1)	40(1)
C(3)	4475(4)	4195(3)	4528(1)	51(1)
C(4)	5578(3)	5369(3)	4586(1)	54(1)
C(5)	5315(3)	6251(2)	5062(1)	47(1)
C(6)	3956(3)	5932(2)	5484(1)	36(1)
C(7)	1399(2)	4434(2)	5898(1)	26(1)
C(8)	-556(3)	4958(2)	5748(1)	37(1)
C(9)	1798(2)	3810(1)	6411(1)	23(1)
C(10)	428(2)	3690(1)	6934(1)	25(1)
C(11)	1765(2)	3217(1)	7424(1)	22(1)
C(12)	2968(2)	2135(1)	7088(1)	25(1)
C(13)	3684(2)	3186(2)	6617(1)	24(1)
C(14)	4617(2)	4289(2)	7024(1)	28(1)
C(15)	3313(2)	4320(1)	7570(1)	24(1)
C(16)	1029(2)	2775(1)	8016(1)	27(1)
C(17)	2530(3)	3087(2)	8476(1)	36(1)
C(18)	4182(2)	3770(2)	8149(1)	29(1)
C(19)	5005(2)	2556(2)	6164(1)	33(1)
O(1)	2538(2)	5643(1)	7712(1)	32(1)
O(2)	-514(2)	2273(1)	8124(1)	39(1)

Supplementary Table 4. Bond lengths [Å] and angles [°] for 3aa.

C(1)-C(2)	1.396(2)
C(1)-C(6)	1.392(2)
C(1)-C(7)	1.493(2)
C(2)-H(2)	0.9300
C(2)-C(3)	1.389(3)
C(3)-H(3)	0.9300
C(3)-C(4)	1.378(4)
C(4)-H(4)	0.9300
C(4)-C(5)	1.387(4)
C(5)-H(5)	0.9300
C(5)-C(6)	1.388(3)
C(6)-H(6)	0.9300
C(7)-C(8)	1.507(2)
C(7)-C(9)	1.339(2)
C(8)-H(8A)	0.9600
C(8)-H(8B)	0.9600
C(8)-H(8C)	0.9600
C(9)-C(10)	1.534(2)
C(9)-C(13)	1.533(2)
C(10)-H(10A)	0.9700
C(10)-H(10B)	0.9700
C(10)-C(11)	1.528(2)
C(11)-C(12)	1.5453(19)
C(11)-C(15)	1.5577(19)
C(11)-C(16)	1.501(2)
C(12)-H(12A)	0.9700
C(12)-H(12B)	0.9700
C(12)-C(13)	1.554(2)
C(13)-C(14)	1.553(2)
C(13)-C(19)	1.514(2)
C(14)-H(14A)	0.9700
C(14)-H(14B)	0.9700
C(14)-C(15)	1.543(2)
C(15)-C(18)	1.544(2)

C(15)-O(1)	1.4222(17)
C(16)-C(17)	1.517(2)
C(16)-O(2)	1.216(2)
C(17)-H(17A)	0.9700
C(17)-H(17B)	0.9700
C(17)-C(18)	1.530(2)
C(18)-H(18A)	0.9700
C(18)-H(18B)	0.9700
C(19)-H(19A)	0.9600
C(19)-H(19B)	0.9600
C(19)-H(19C)	0.9600
O(1)-H(1)	0.8200

C(2)-C(1)-C(7)	122.07(15)
C(6)-C(1)-C(2)	118.44(16)
C(6)-C(1)-C(7)	119.45(15)
C(1)-C(2)-H(2)	119.8
C(3)-C(2)-C(1)	120.38(19)
C(3)-C(2)-H(2)	119.8
C(2)-C(3)-H(3)	119.8
C(4)-C(3)-C(2)	120.4(2)
C(4)-C(3)-H(3)	119.8
C(3)-C(4)-H(4)	120.0
C(3)-C(4)-C(5)	120.1(2)
C(5)-C(4)-H(4)	120.0
C(4)-C(5)-H(5)	120.2
C(4)-C(5)-C(6)	119.6(2)
C(6)-C(5)-H(5)	120.2
C(1)-C(6)-H(6)	119.4
C(5)-C(6)-C(1)	121.11(18)
C(5)-C(6)-H(6)	119.4
C(1)-C(7)-C(8)	113.82(13)
C(9)-C(7)-C(1)	123.51(14)
C(9)-C(7)-C(8)	122.61(15)
C(7)-C(8)-H(8A)	109.5
C(7)-C(8)-H(8B)	109.5

C(7)-C(8)-H(8C)	109.5
H(8A)-C(8)-H(8B)	109.5
H(8A)-C(8)-H(8C)	109.5
H(8B)-C(8)-H(8C)	109.5
C(7)-C(9)-C(10)	124.96(13)
C(7)-C(9)-C(13)	128.57(13)
C(13)-C(9)-C(10)	106.31(11)
C(9)-C(10)-H(10A)	111.5
C(9)-C(10)-H(10B)	111.5
H(10A)-C(10)-H(10B)	109.3
C(11)-C(10)-C(9)	101.38(12)
C(11)-C(10)-H(10A)	111.5
C(11)-C(10)-H(10B)	111.5
C(10)-C(11)-C(12)	100.27(11)
C(10)-C(11)-C(15)	112.60(11)
C(12)-C(11)-C(15)	100.32(11)
C(16)-C(11)-C(10)	121.52(13)
C(16)-C(11)-C(12)	116.19(12)
C(16)-C(11)-C(15)	104.23(11)
C(11)-C(12)-H(12A)	112.8
C(11)-C(12)-H(12B)	112.8
C(11)-C(12)-C(13)	94.58(11)
H(12A)-C(12)-H(12B)	110.3
C(13)-C(12)-H(12A)	112.8
C(13)-C(12)-H(12B)	112.8
C(9)-C(13)-C(12)	100.52(12)
C(9)-C(13)-C(14)	106.33(11)
C(14)-C(13)-C(12)	100.04(12)
C(19)-C(13)-C(9)	118.93(12)
C(19)-C(13)-C(12)	113.96(12)
C(19)-C(13)-C(14)	114.59(13)
C(13)-C(14)-H(14A)	111.0
C(13)-C(14)-H(14B)	111.0
H(14A)-C(14)-H(14B)	109.0
C(15)-C(14)-C(13)	103.76(11)
C(15)-C(14)-H(14A)	111.0

C(15)-C(14)-H(14B)	111.0
C(14)-C(15)-C(11)	103.55(11)
C(14)-C(15)-C(18)	116.11(12)
C(18)-C(15)-C(11)	103.02(11)
O(1)-C(15)-C(11)	112.84(12)
O(1)-C(15)-C(14)	115.34(12)
O(1)-C(15)-C(18)	105.48(11)
C(11)-C(16)-C(17)	108.56(13)
O(2)-C(16)-C(11)	127.04(15)
O(2)-C(16)-C(17)	124.39(14)
C(16)-C(17)-H(17A)	110.4
C(16)-C(17)-H(17B)	110.4
C(16)-C(17)-C(18)	106.43(13)
H(17A)-C(17)-H(17B)	108.6
C(18)-C(17)-H(17A)	110.4
C(18)-C(17)-H(17B)	110.4
C(15)-C(18)-H(18A)	110.8
C(15)-C(18)-H(18B)	110.8
C(17)-C(18)-C(15)	104.94(13)
C(17)-C(18)-H(18A)	110.8
C(17)-C(18)-H(18B)	110.8
H(18A)-C(18)-H(18B)	108.8
C(13)-C(19)-H(19A)	109.5
C(13)-C(19)-H(19B)	109.5
C(13)-C(19)-H(19C)	109.5
H(19A)-C(19)-H(19B)	109.5
H(19A)-C(19)-H(19C)	109.5
H(19B)-C(19)-H(19C)	109.5
C(15)-O(1)-H(1)	109.5

Symmetry transformations used to generate equivalent atoms:

Supplementary Table 5. Anisotropic displacement parameters (Å^{2}x 10^3) for 3aa.

The anisotropic displacement factor exponent takes the form: -2 ^{2}[h^2 a*^{2}U^{11} + ... + 2 h k a* b* U^{12}]

	U^{11}	U^{22}	U^{33}	U^{23}	U^{13}	U^{12}
C(1)	28(1)	31(1)	26(1)	5(1)	-6(1)	4(1)
C(2)	47(1)	43(1)	29(1)	-1(1)	-4(1)	4(1)
C(3)	54(1)	67(1)	30(1)	2(1)	3(1)	13(1)
C(4)	41(1)	83(2)	38(1)	23(1)	3(1)	7(1)
C(5)	36(1)	55(1)	51(1)	22(1)	-8(1)	-7(1)
C(6)	38(1)	35(1)	36(1)	7(1)	-8(1)	-2(1)
C(7)	26(1)	27(1)	27(1)	-2(1)	-5(1)	1(1)
C(8)	29(1)	46(1)	37(1)	3(1)	-8(1)	7(1)
C(9)	21(1)	22(1)	27(1)	-3(1)	-3(1)	1(1)
C(10)	20(1)	25(1)	28(1)	-2(1)	-2(1)	-1(1)
C(11)	21(1)	20(1)	25(1)	-2(1)	-1(1)	-1(1)
C(12)	28(1)	21(1)	28(1)	-1(1)	-1(1)	3(1)
C(13)	21(1)	27(1)	25(1)	0(1)	-1(1)	3(1)
C(14)	22(1)	32(1)	30(1)	4(1)	-3(1)	-4(1)
C(15)	23(1)	21(1)	28(1)	-1(1)	-4(1)	-1(1)
C(16)	30(1)	23(1)	28(1)	-1(1)	2(1)	-2(1)
C(17)	37(1)	45(1)	25(1)	1(1)	-1(1)	-6(1)
C(18)	28(1)	30(1)	29(1)	1(1)	-7(1)	-2(1)
C(19)	30(1)	38(1)	32(1)	2(1)	4(1)	11(1)
O(1)	38(1)	21(1)	35(1)	-5(1)	-9(1)	1(1)
O(2)	37(1)	43(1)	36(1)	0(1)	4(1)	-14(1)

Supplementary Table 6. Hydrogen coordinates (x 10^4) and isotropic displacement parameters ($Å^2$x 10^3) for 3aa.

	x	y	z	U(eq)
H(2)	2341	3102	4898	48
H(3)	4652	3608	4207	61
H(4)	6500	5570	4306	65
H(5)	6045	7052	5100	57
H(6)	3780	6526	5802	43
H(8A)	-512	5945	5688	56
H(8B)	-1409	4749	6066	56
H(8C)	-992	4513	5394	56
H(10A)	-155	4577	7025	30
H(10B)	-554	3007	6859	30
H(12A)	2209	1394	6919	31
H(12B)	3985	1752	7325	31
H(14A)	5895	4017	7131	33
H(14B)	4657	5191	6833	33
H(17A)	2944	2236	8666	43
H(17B)	2031	3709	8775	43
H(18A)	5174	3098	8069	35
H(18B)	4712	4526	8380	35
H(19A)	4312	1918	5921	50
H(19B)	6011	2071	6361	50
H(19C)	5530	3281	5924	50
H(1)	1939	5940	7429	47

Supplementary Table 7. Torsion angles [°] for 3aa.

C(1)-C(2)-C(3)-C(4)	-1.6(3)
C(1)-C(7)-C(9)-C(10)	169.70(13)
C(1)-C(7)-C(9)-C(13)	-5.2(2)
C(2)-C(1)-C(6)-C(5)	-1.9(2)
C(2)-C(1)-C(7)-C(8)	-83.81(19)
C(2)-C(1)-C(7)-C(9)	98.97(19)
C(2)-C(3)-C(4)-C(5)	-0.3(3)
C(3)-C(4)-C(5)-C(6)	1.0(3)
C(4)-C(5)-C(6)-C(1)	0.1(3)
C(6)-C(1)-C(2)-C(3)	2.6(3)
C(6)-C(1)-C(7)-C(8)	93.93(17)
C(6)-C(1)-C(7)-C(9)	-83.29(19)
C(7)-C(1)-C(2)-C(3)	-179.60(17)
C(7)-C(1)-C(6)-C(5)	-179.75(15)
C(7)-C(9)-C(10)-C(11)	-167.06(13)
C(7)-C(9)-C(13)-C(12)	-156.70(14)
C(7)-C(9)-C(13)-C(14)	99.47(17)
C(7)-C(9)-C(13)-C(19)	-31.6(2)
C(8)-C(7)-C(9)-C(10)	-7.3(2)
C(8)-C(7)-C(9)-C(13)	177.80(15)
C(9)-C(10)-C(11)-C(12)	-42.39(12)
C(9)-C(10)-C(11)-C(15)	63.43(13)
C(9)-C(10)-C(11)-C(16)	-171.99(11)
C(9)-C(13)-C(14)-C(15)	68.17(13)
C(10)-C(9)-C(13)-C(12)	27.64(13)
C(10)-C(9)-C(13)-C(14)	-76.19(13)
C(10)-C(9)-C(13)-C(19)	152.71(13)
C(10)-C(11)-C(12)-C(13)	58.85(12)
C(10)-C(11)-C(15)-C(14)	-70.36(14)
C(10)-C(11)-C(15)-C(18)	168.28(12)
C(10)-C(11)-C(15)-O(1)	55.03(16)
C(10)-C(11)-C(16)-C(17)	-151.03(13)
C(10)-C(11)-C(16)-O(2)	27.8(2)
C(11)-C(12)-C(13)-C(9)	-51.96(12)

C(11)-C(12)-C(13)-C(14)	56.90(13)
C(11)-C(12)-C(13)-C(19)	179.65(13)
C(11)-C(15)-C(18)-C(17)	-33.84(15)
C(11)-C(16)-C(17)-C(18)	1.56(17)
C(12)-C(11)-C(15)-C(14)	35.43(13)
C(12)-C(11)-C(15)-C(18)	-85.94(13)
C(12)-C(11)-C(15)-O(1)	160.82(12)
C(12)-C(11)-C(16)-C(17)	86.64(15)
C(12)-C(11)-C(16)-O(2)	-94.57(19)
C(12)-C(13)-C(14)-C(15)	-36.01(14)
C(13)-C(9)-C(10)-C(11)	8.80(13)
C(13)-C(14)-C(15)-C(11)	0.50(14)
C(13)-C(14)-C(15)-C(18)	112.61(13)
C(13)-C(14)-C(15)-O(1)	-123.27(13)
C(14)-C(15)-C(18)-C(17)	-146.25(13)
C(15)-C(11)-C(12)-C(13)	-56.62(12)
C(15)-C(11)-C(16)-C(17)	-22.67(15)
C(15)-C(11)-C(16)-O(2)	156.12(15)
C(16)-C(11)-C(12)-C(13)	-168.21(12)
C(16)-C(11)-C(15)-C(14)	156.02(11)
C(16)-C(11)-C(15)-C(18)	34.66(14)
C(16)-C(11)-C(15)-O(1)	-78.58(14)
C(16)-C(17)-C(18)-C(15)	20.45(17)
C(19)-C(13)-C(14)-C(15)	-158.32(12)
O(1)-C(15)-C(18)-C(17)	84.68(14)
O(2)-C(16)-C(17)-C(18)	-177.27(15)

Symmetry transformations used to generate equivalent atoms:

Supplementary Table 8. Hydrogen bonds for 3aa [Å and °].

D-H...A	d(D-H)	d(H...A)	d(D...A)	<(DHA)
O(1)-H(1)...O(2)#1	0.82	2.06	2.8445(18)	161.3

Symmetry transformations used to generate equivalent atoms:

#1 -x,y+1/2,-z+3/2

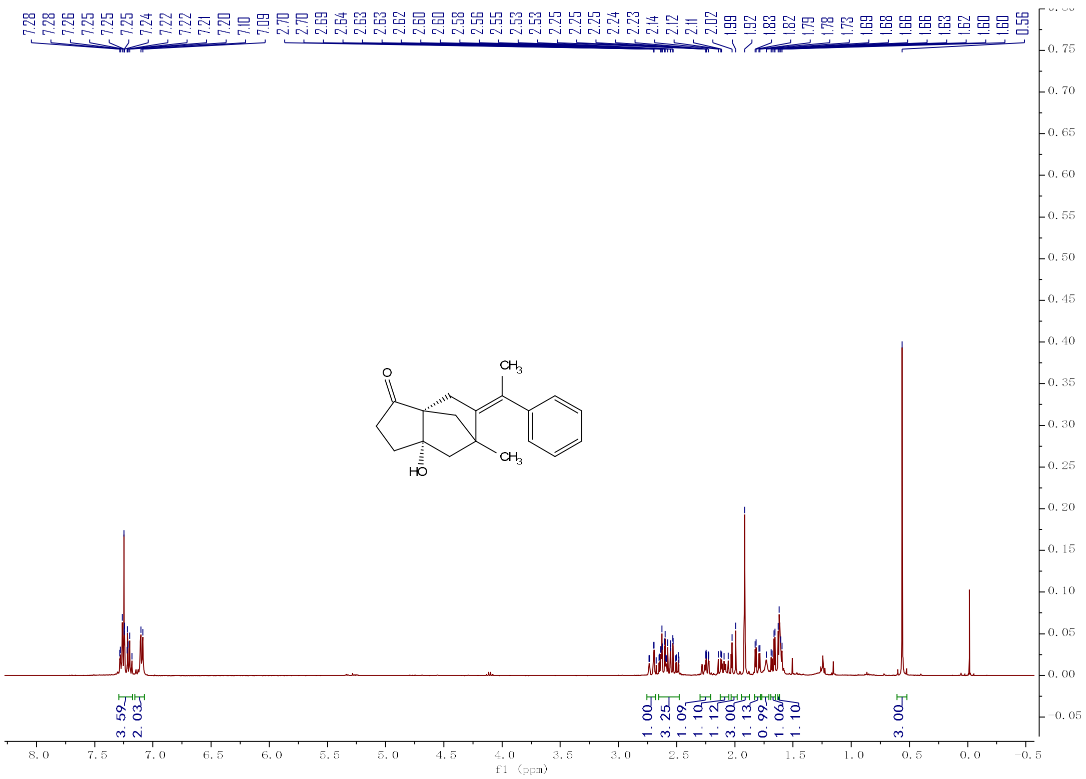

Supplementary Figure 89. ^{1}H NMR spectrum of 3aa

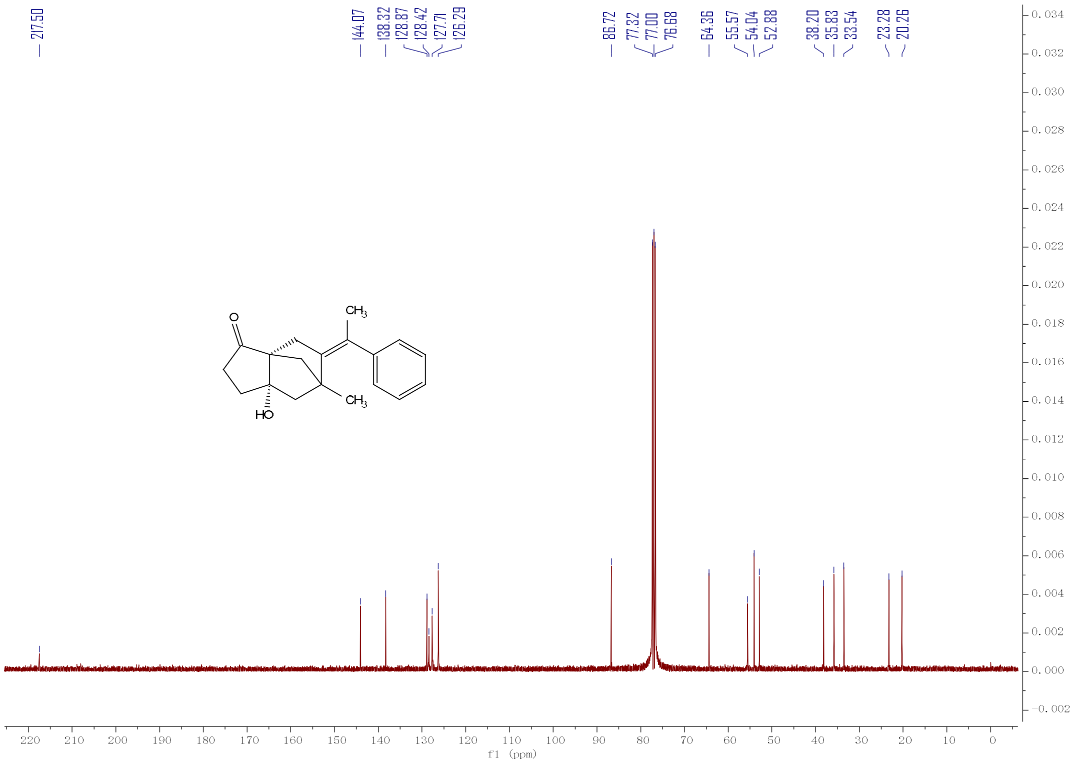

Supplementary Figure 90. ^{13}C NMR spectrum of 3aa

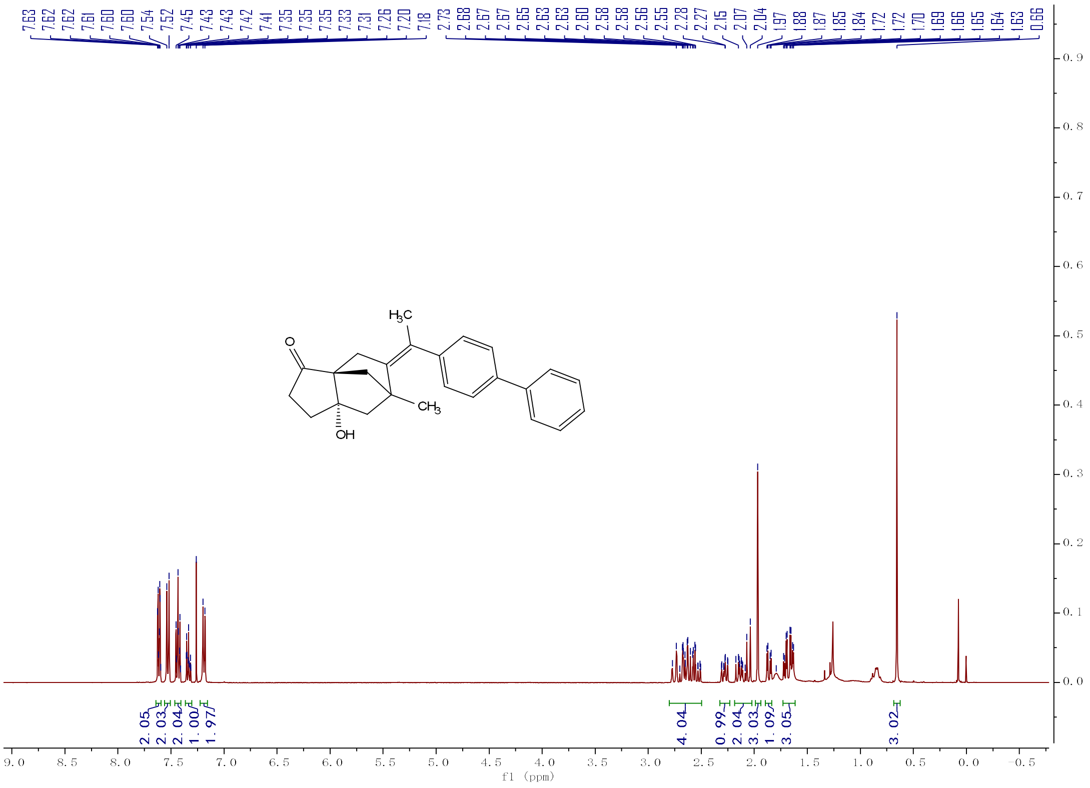

Supplementary Figure 91. ^{1}H NMR spectrum of 3ab

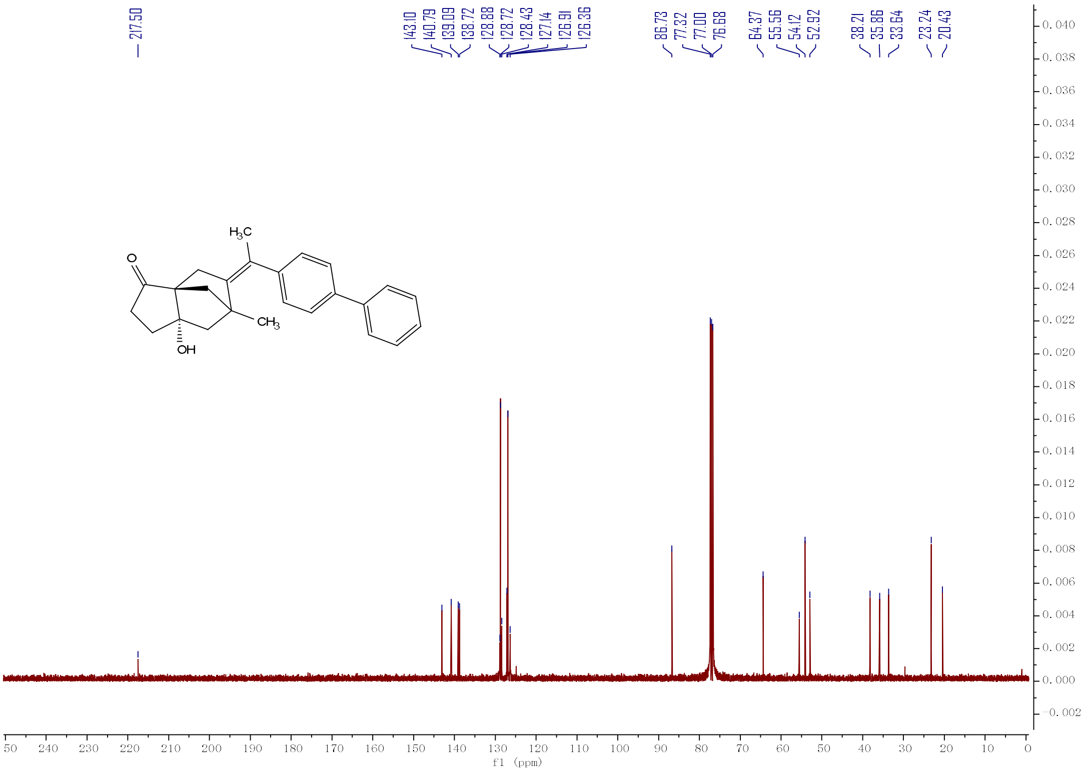

Supplementary Figure 92. ^{13}C NMR spectrum of 3ab

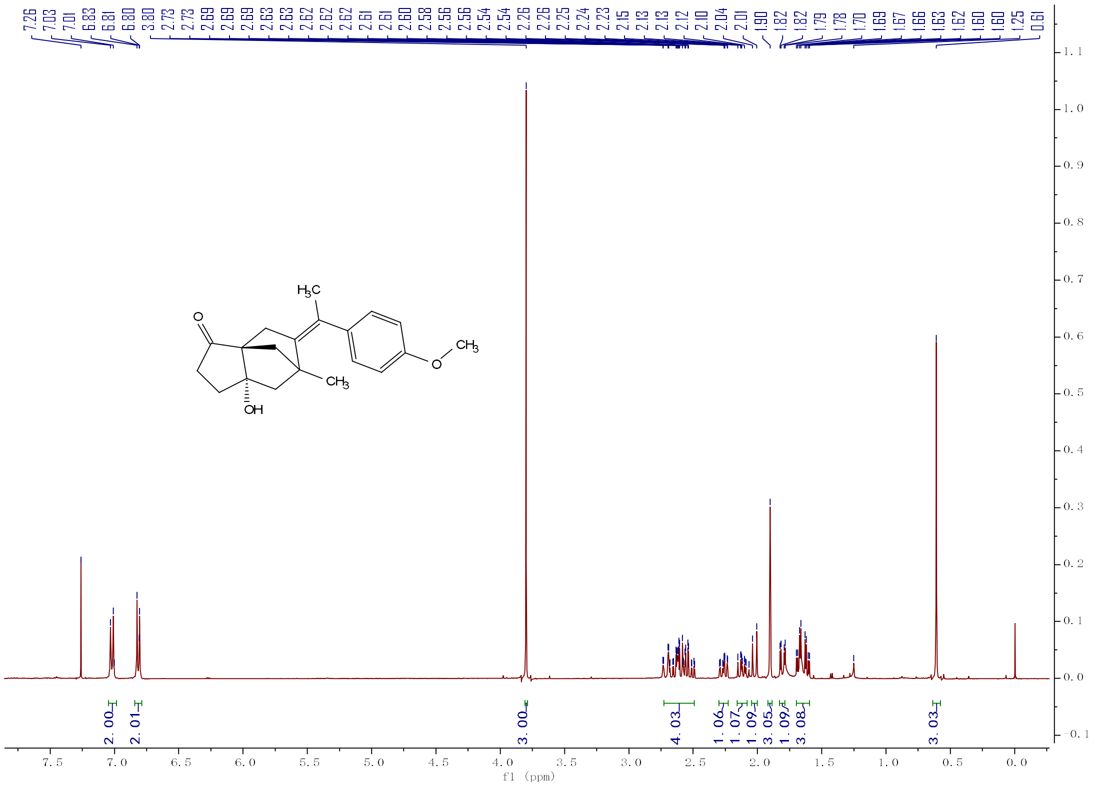

Supplementary Figure 93. ^{1}H NMR spectrum of 3ac

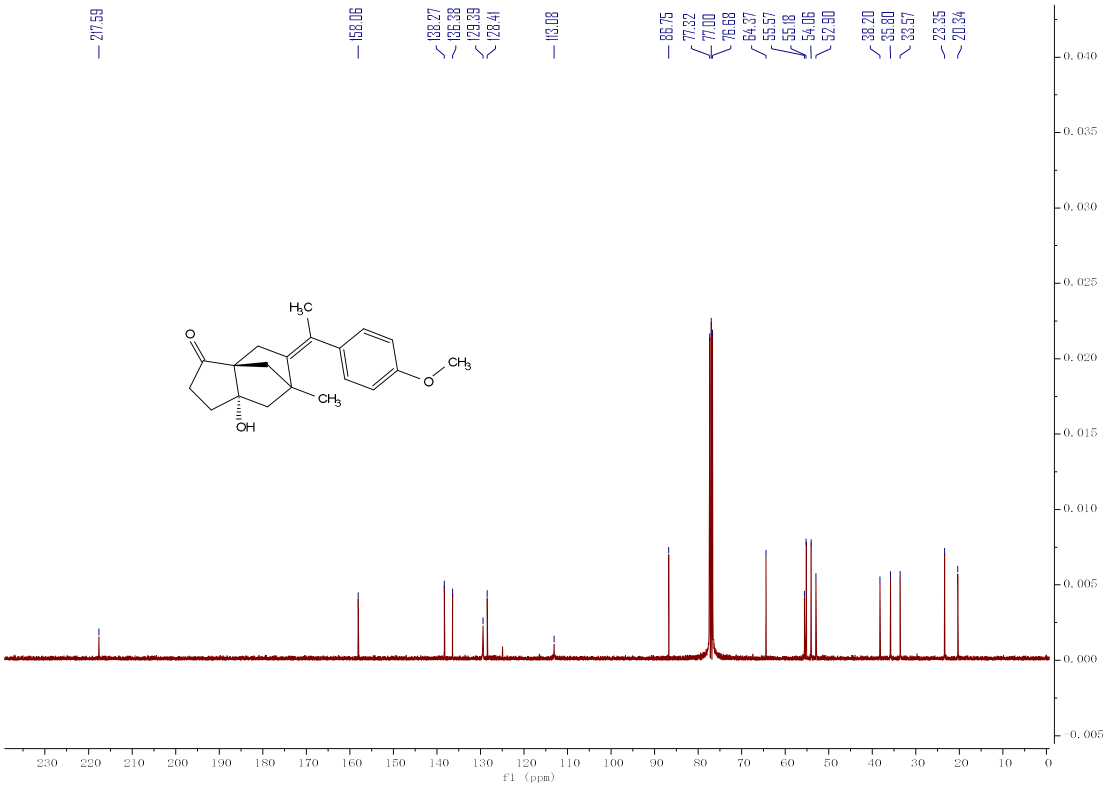

Supplementary Figure 94. ^{13}C NMR spectrum of 3ac

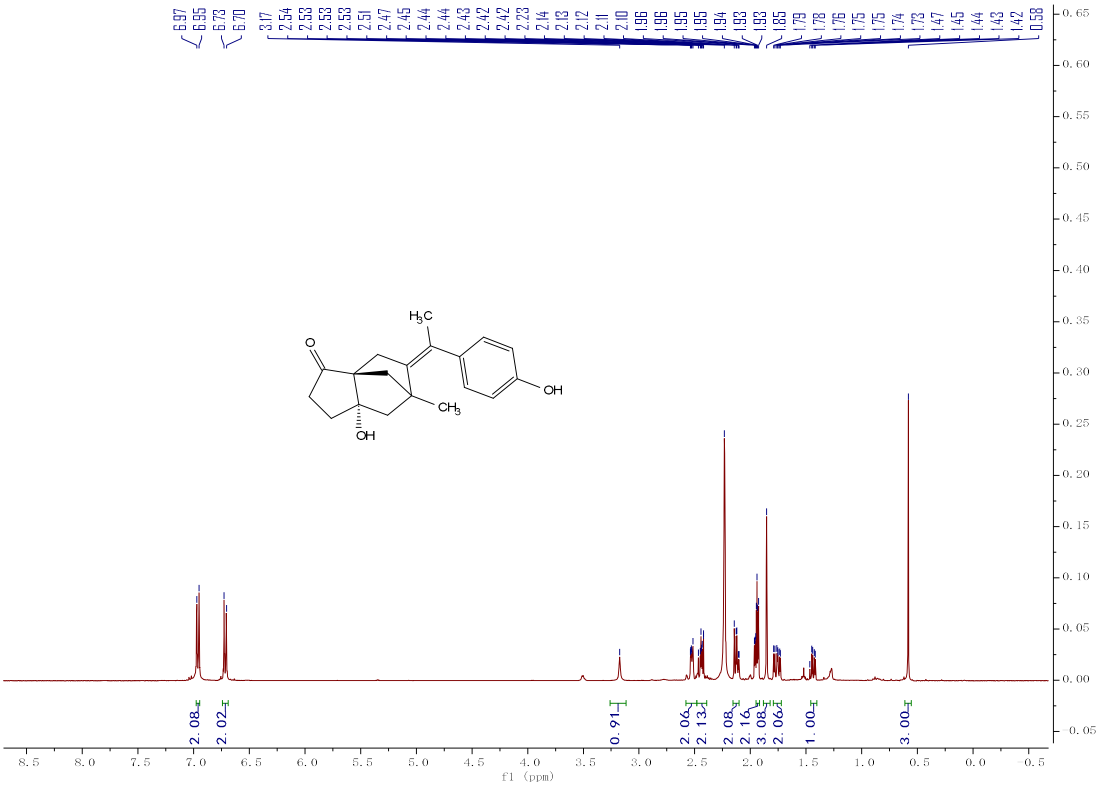

Supplementary Figure 95. ^{1}H NMR spectrum of 3ad

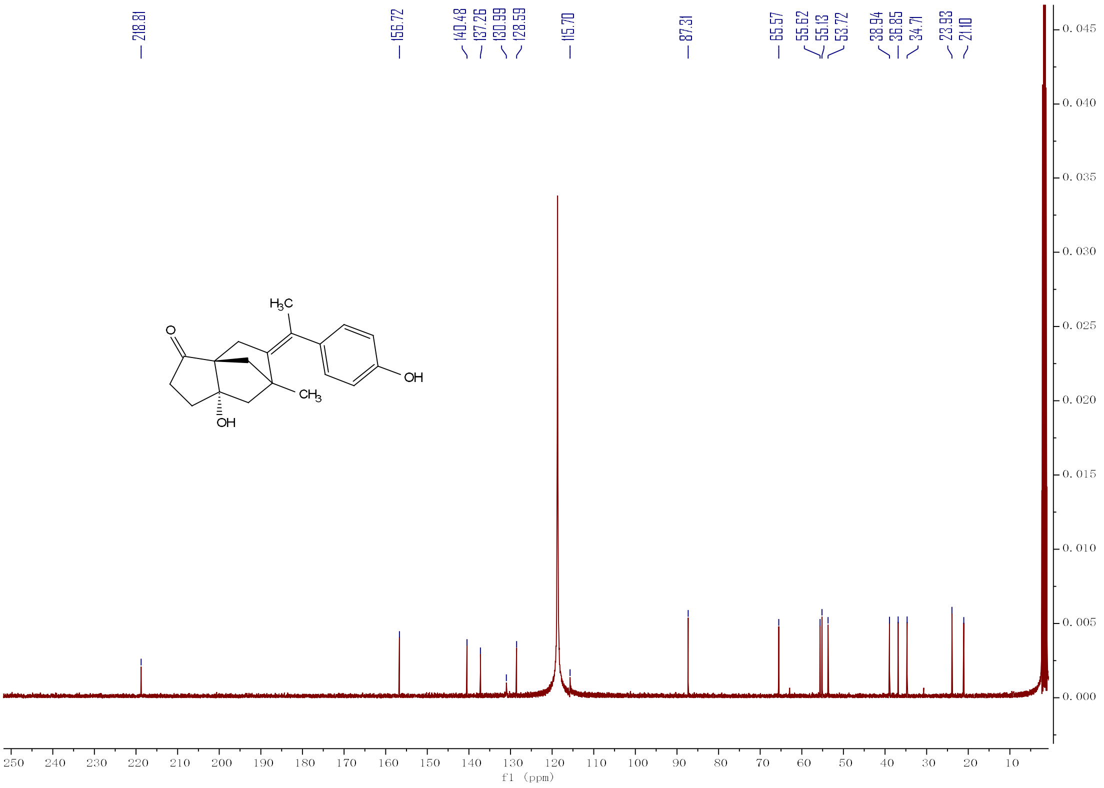

Supplementary Figure 96. ^{13}C NMR spectrum of 3ad

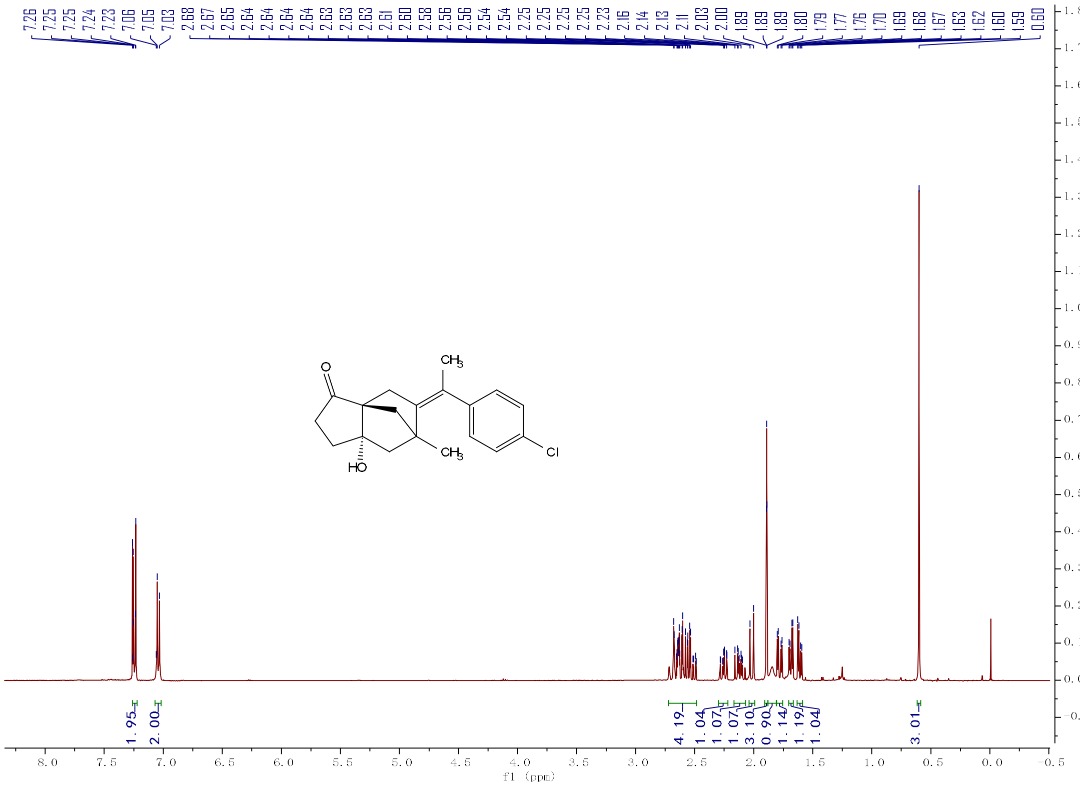

Supplementary Figure 97. ^{1}H NMR spectrum of 3ae

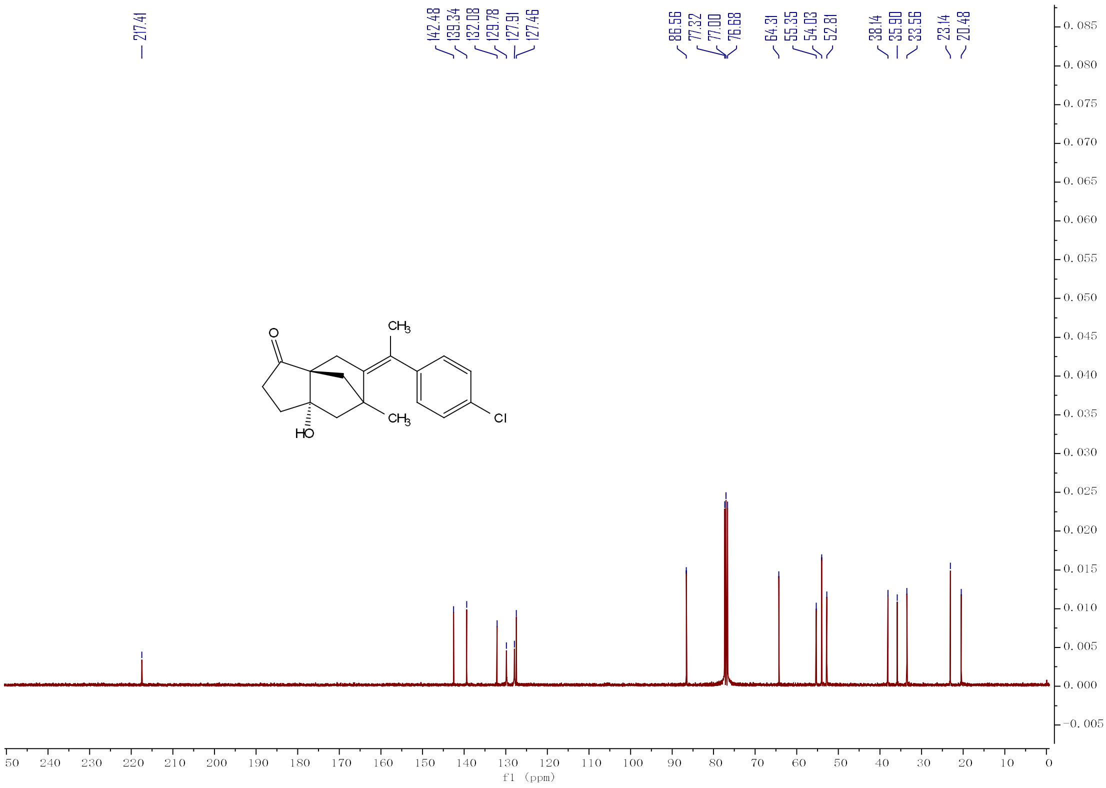

Supplementary Figure 98. ^{13}C NMR spectrum of 3ae

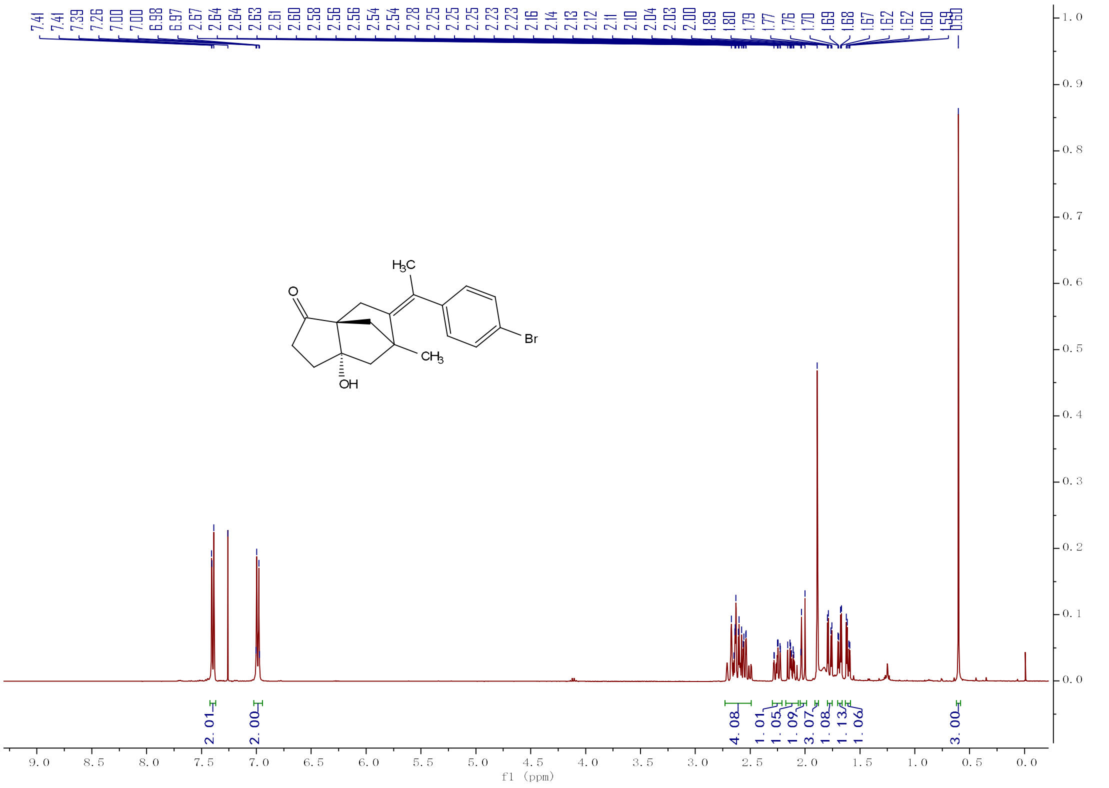

Supplementary Figure 99. ^{1}H NMR spectrum of 3af

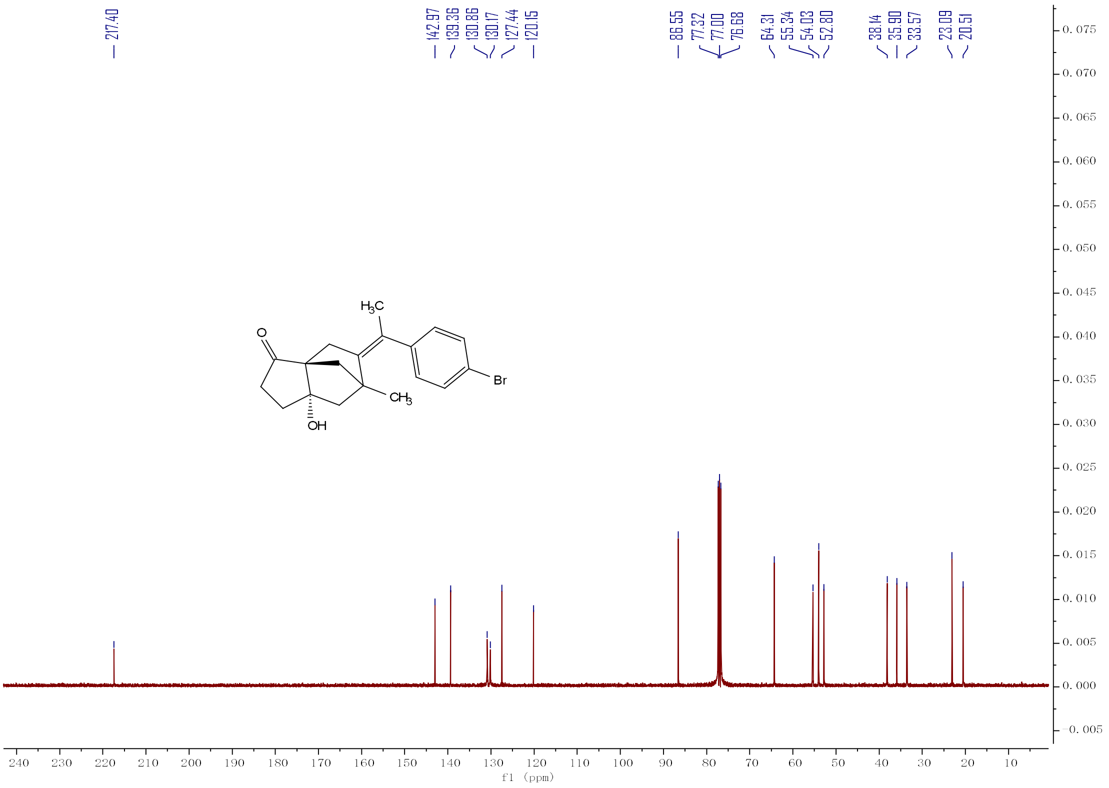

Supplementary Figure 100. ^{13}C NMR spectrum of 3af

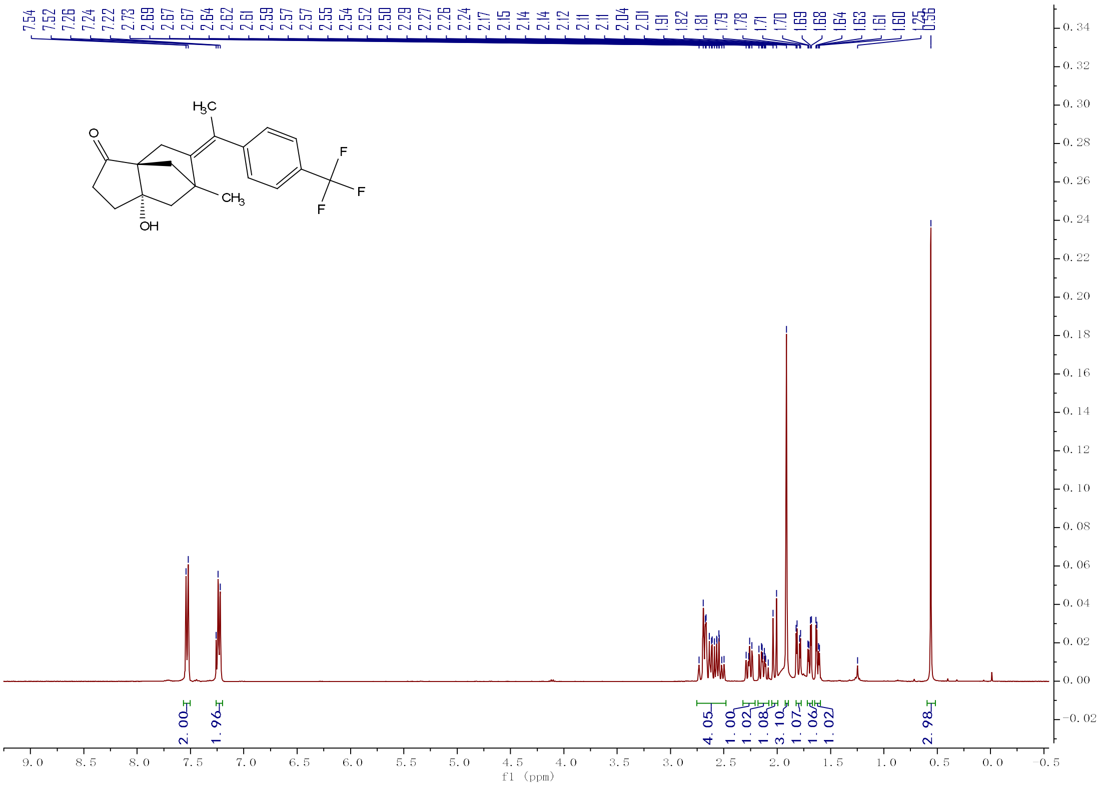

Supplementary Figure 101. ^{1}H NMR spectrum of 3ag

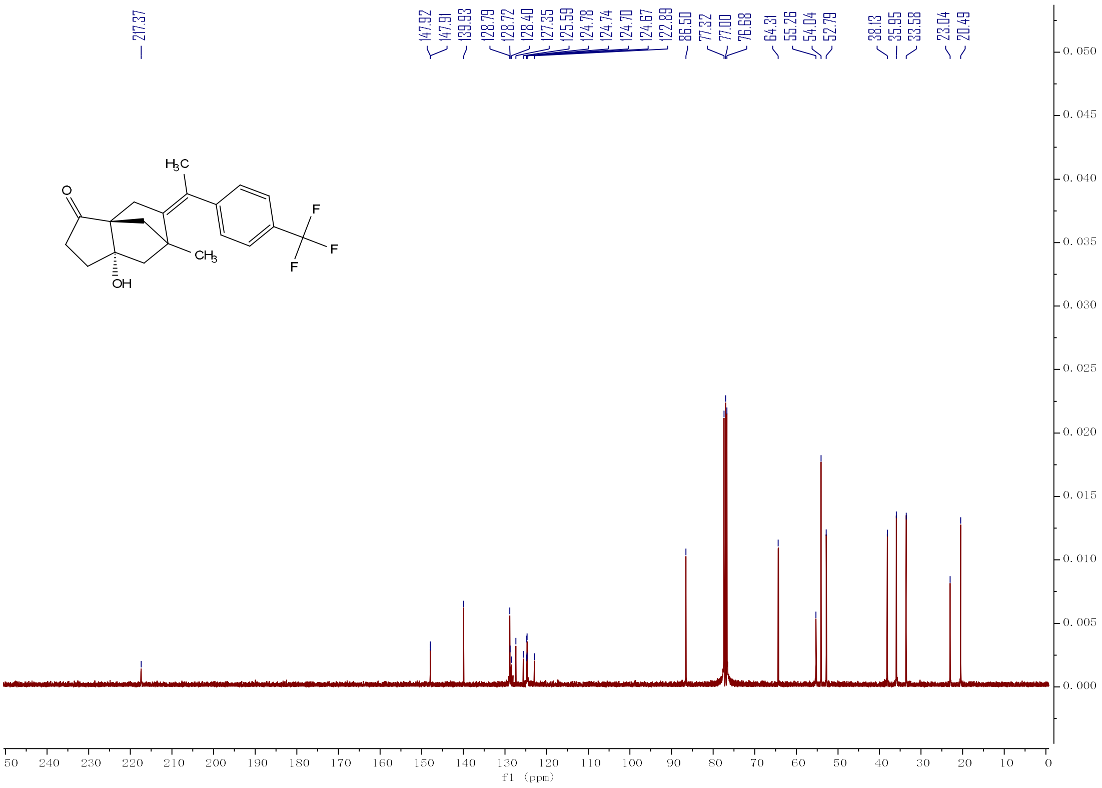

Supplementary Figure 102. ^{13}C NMR spectrum of 3ag

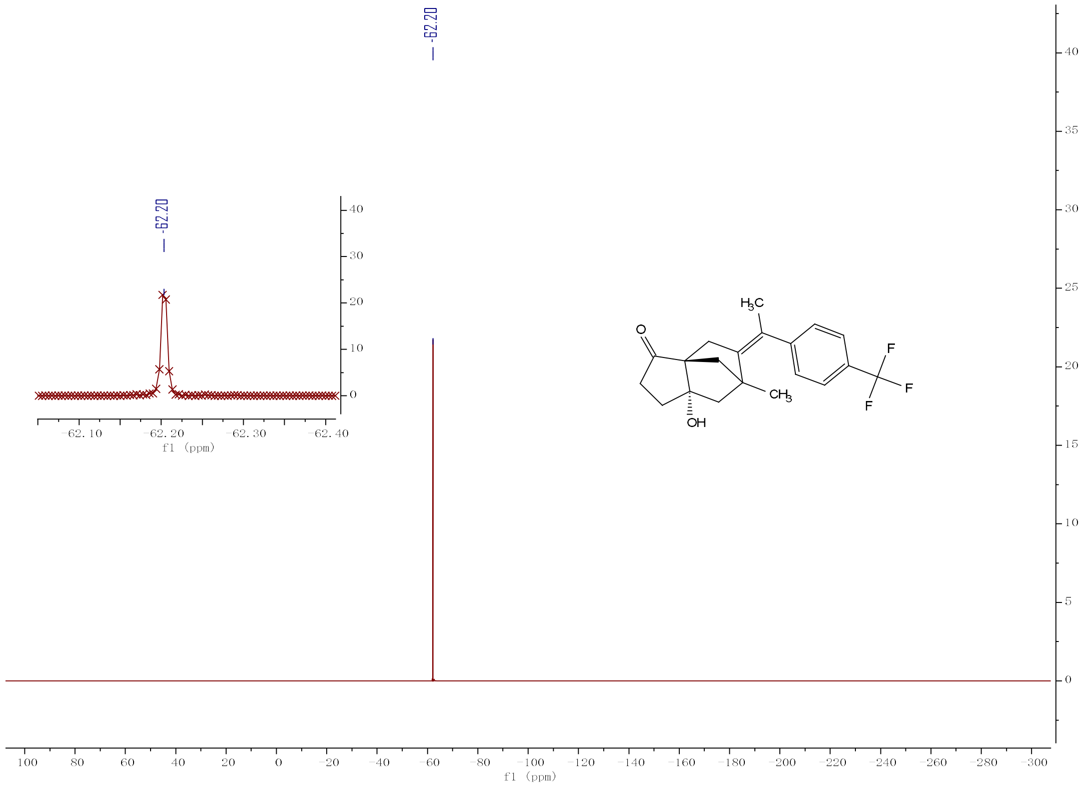

Supplementary Figure 103. ^{19}F NMR spectrum of 3ag

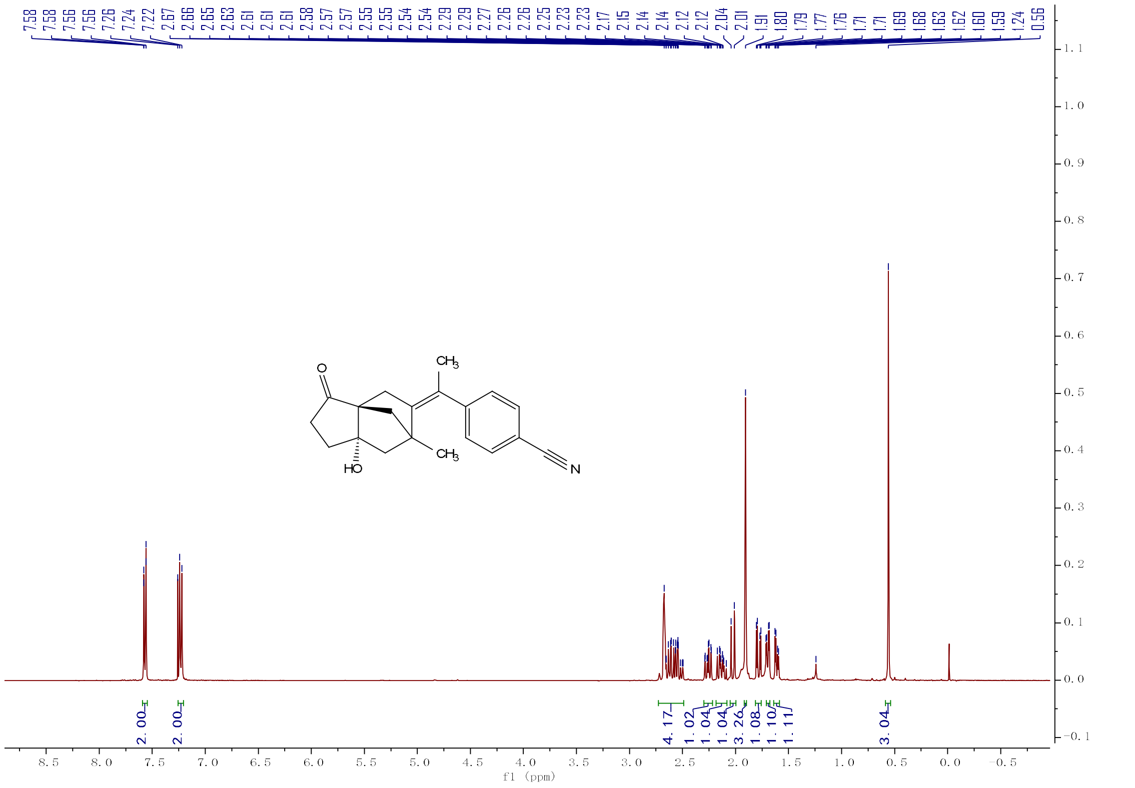

Supplementary Figure 104. ^{1}H NMR spectrum of 3ah

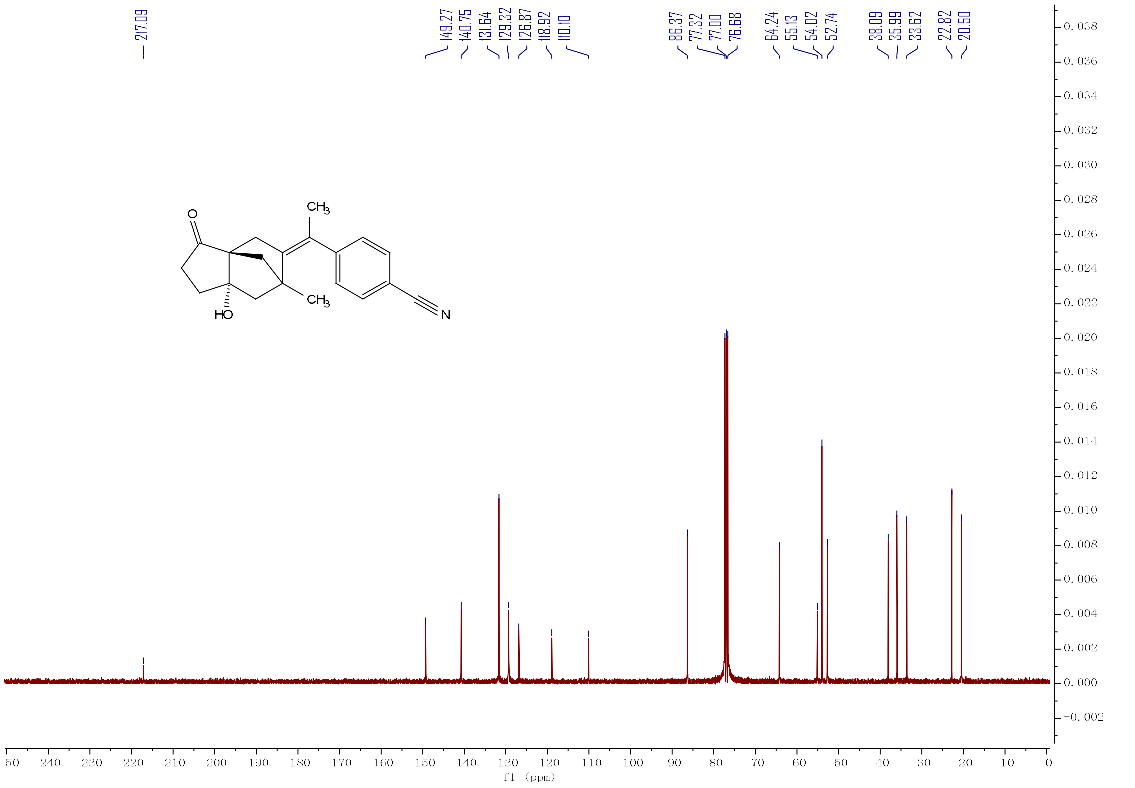

Supplementary Figure 105. ^{13}C NMR spectrum of 3ah

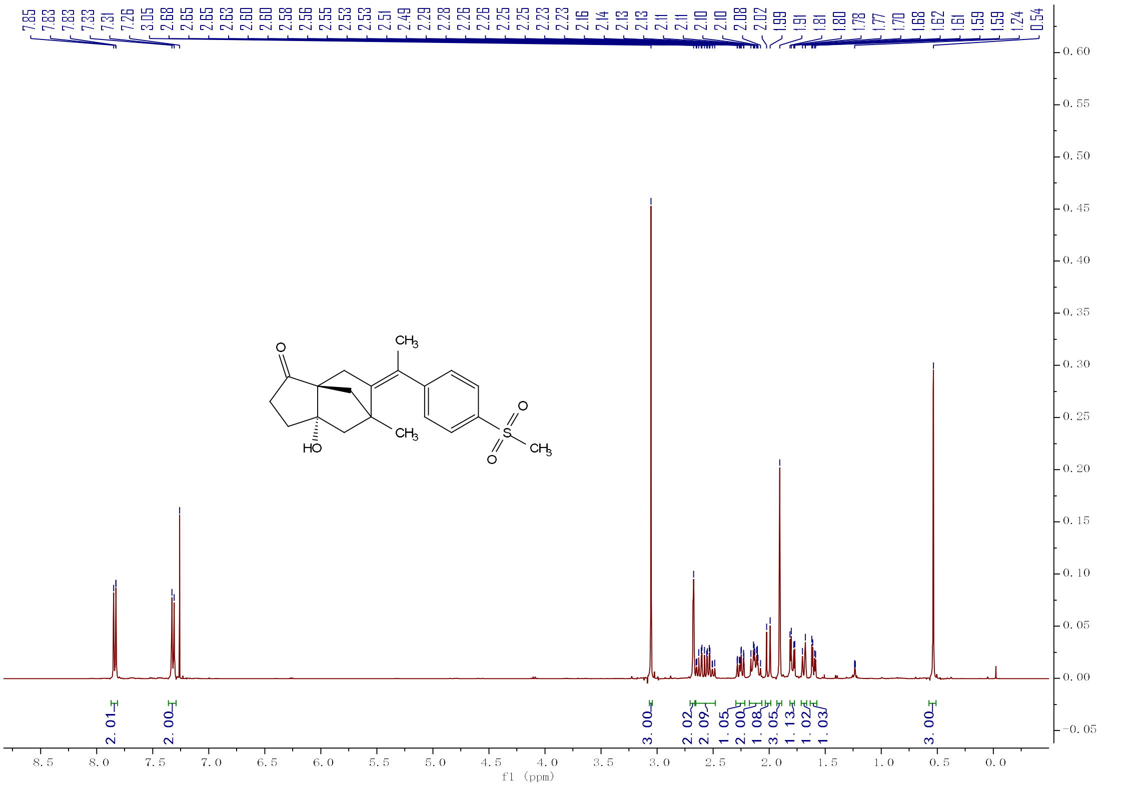

Supplementary Figure 106. ^{1}H NMR spectrum of 3ai

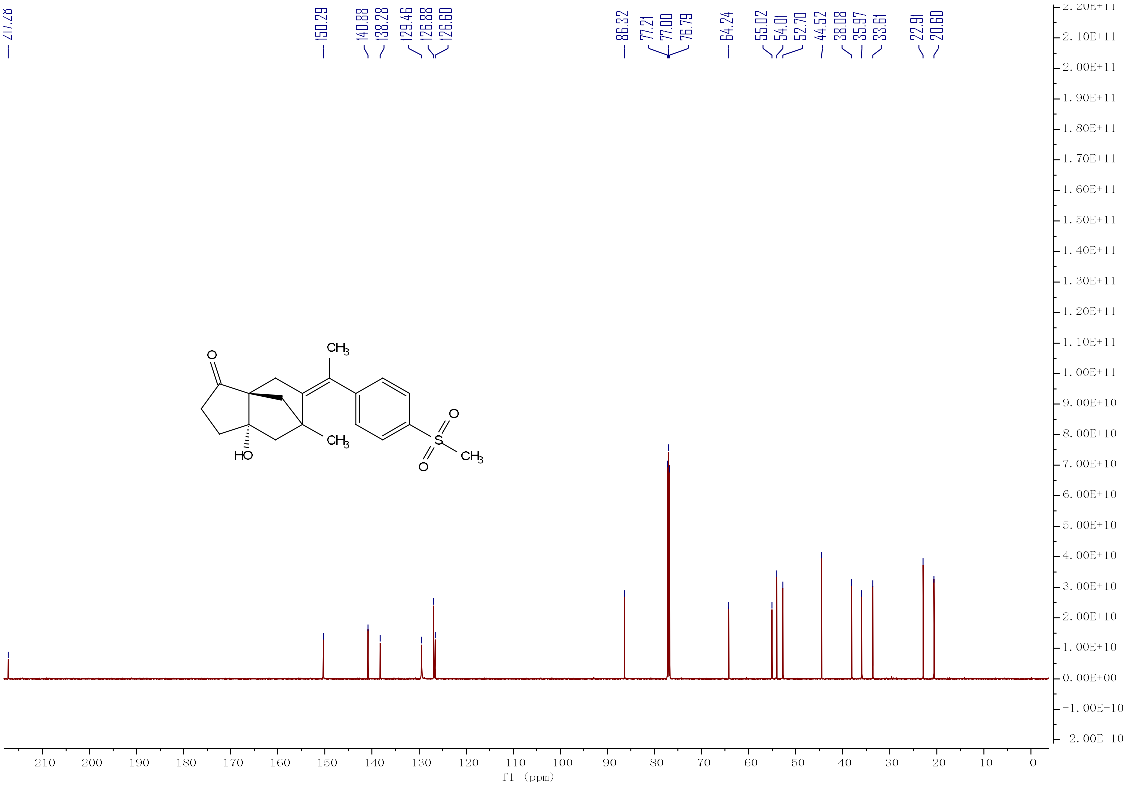

Supplementary Figure 107. ^{13}C NMR spectrum of 3ai

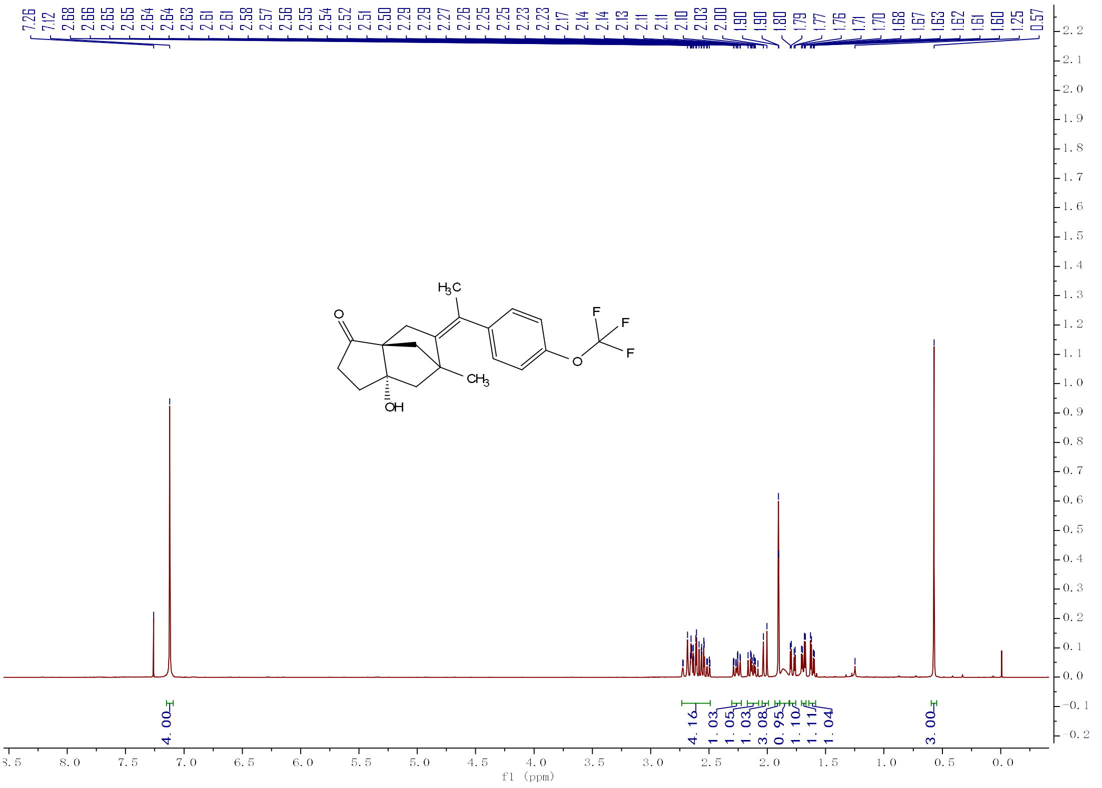

Supplementary Figure 108. ^{1}H NMR spectrum of 3aj

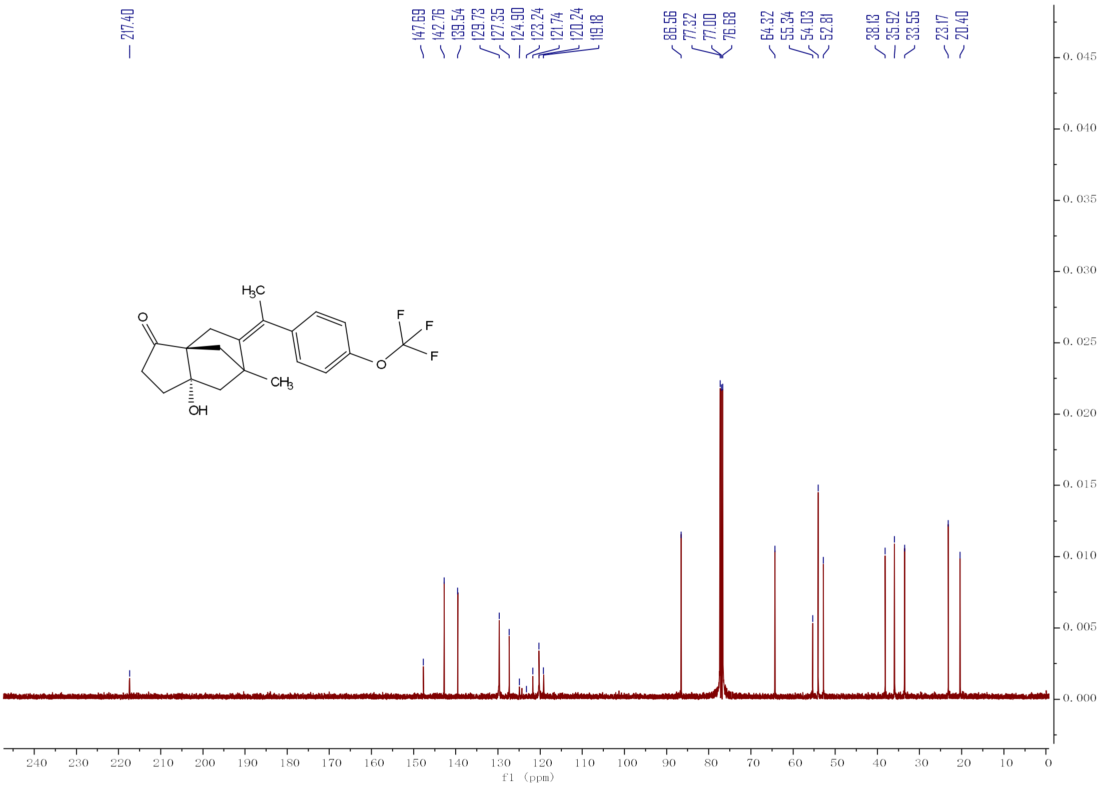

Supplementary Figure 109. ^{13}C NMR spectrum of 3aj

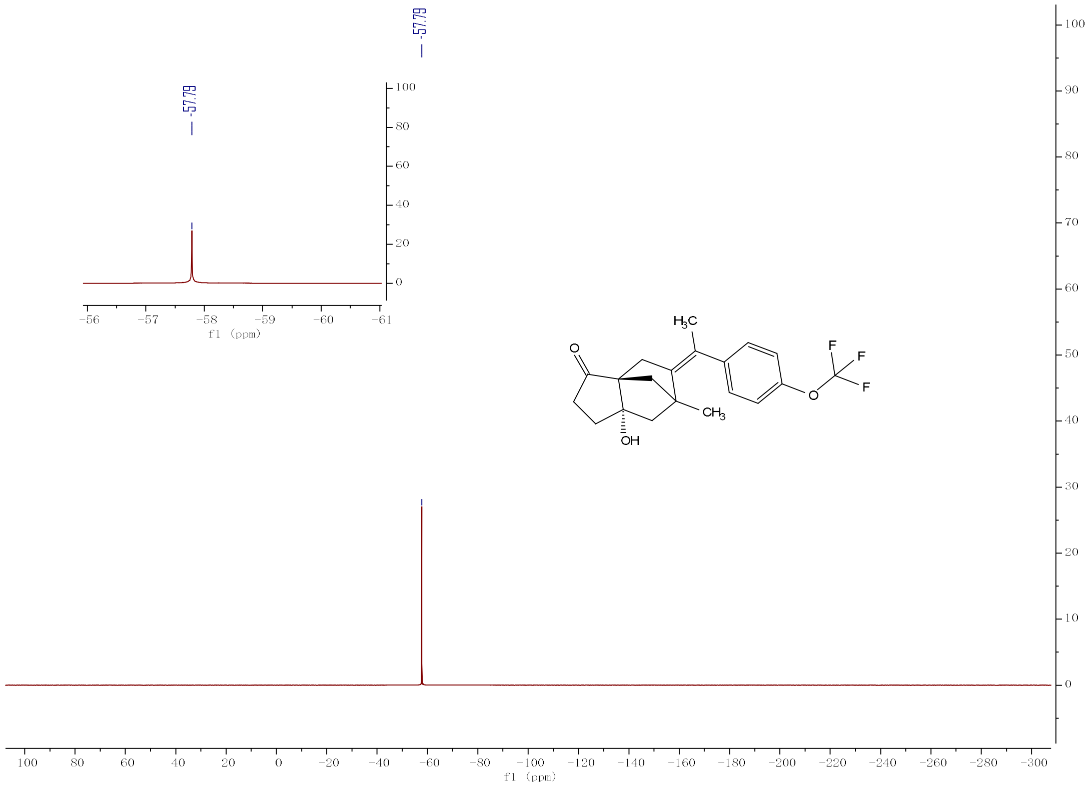

Supplementary Figure 110. ^{19}F NMR spectrum of 3aj

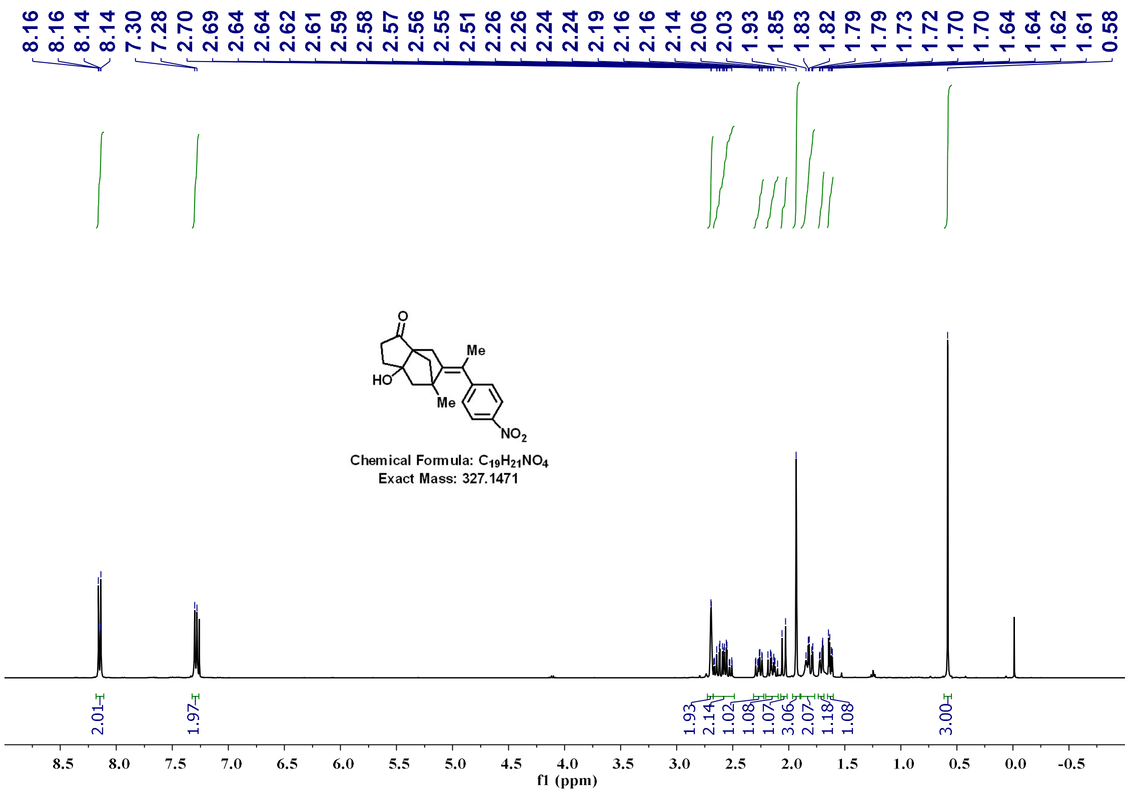

Supplementary Figure 111. ^{1}H NMR spectrum of 3ak

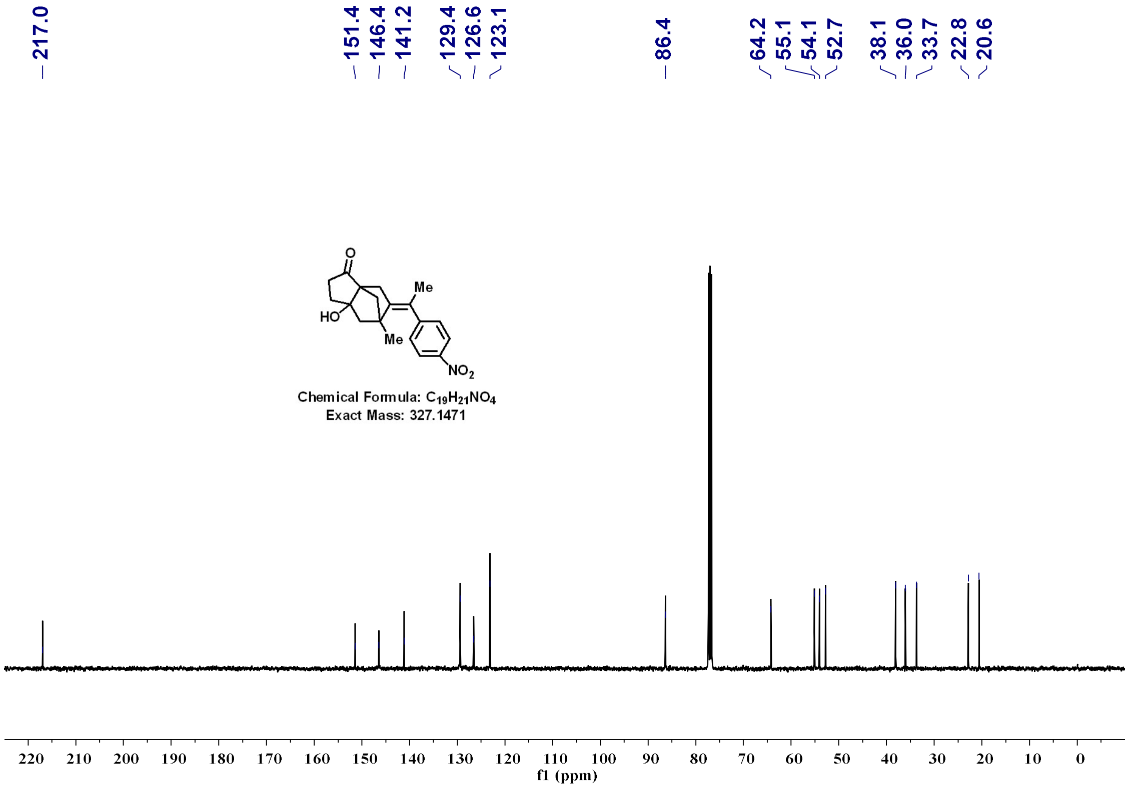

Supplementary Figure 112. ^{13}C NMR spectrum of 3ak

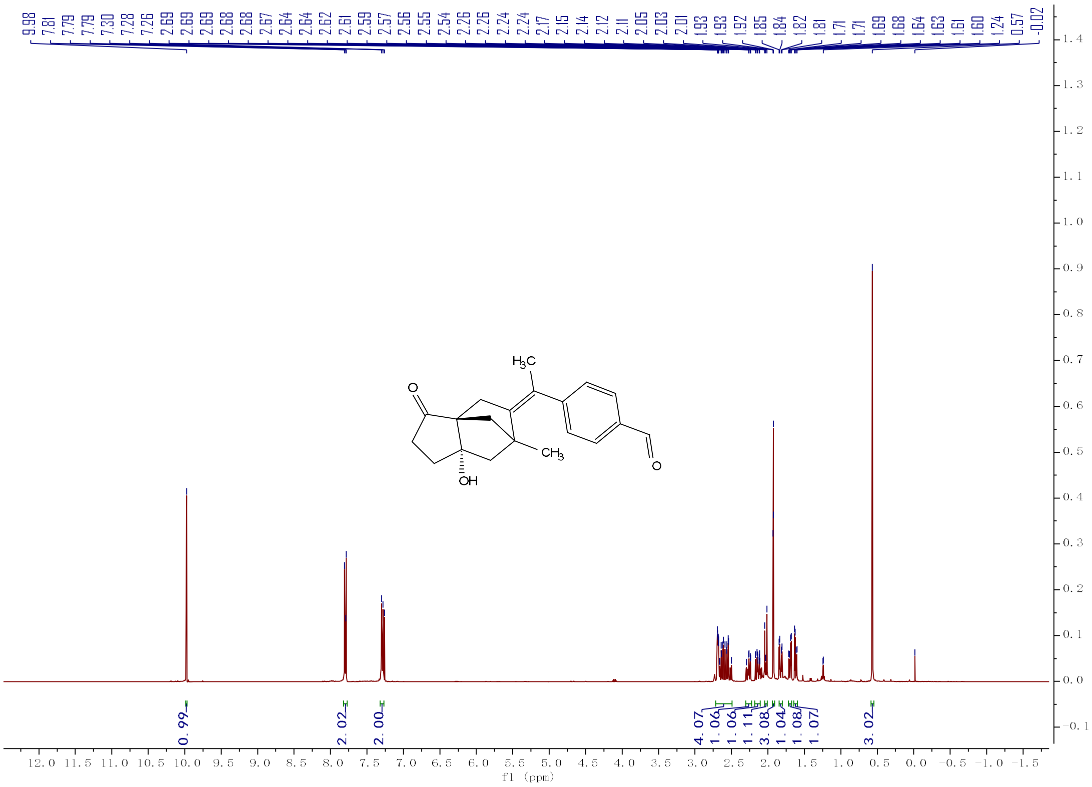

Supplementary Figure 113. ^{1}H NMR spectrum of 3al

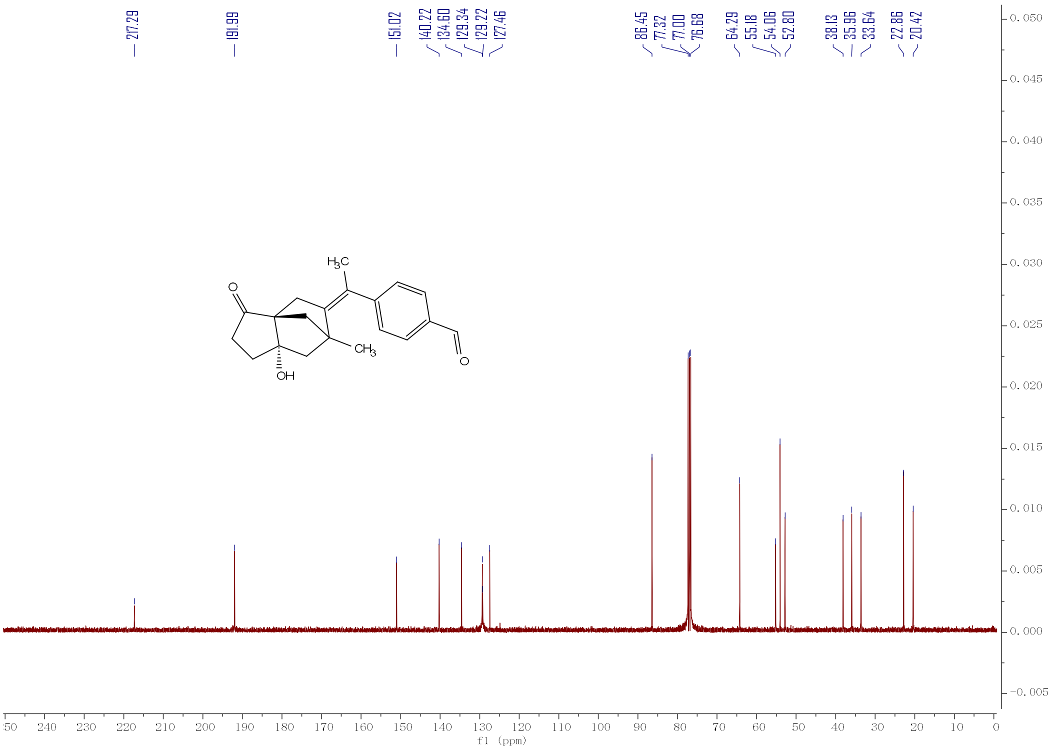

Supplementary Figure 114. ^{13}C NMR spectrum of 3al

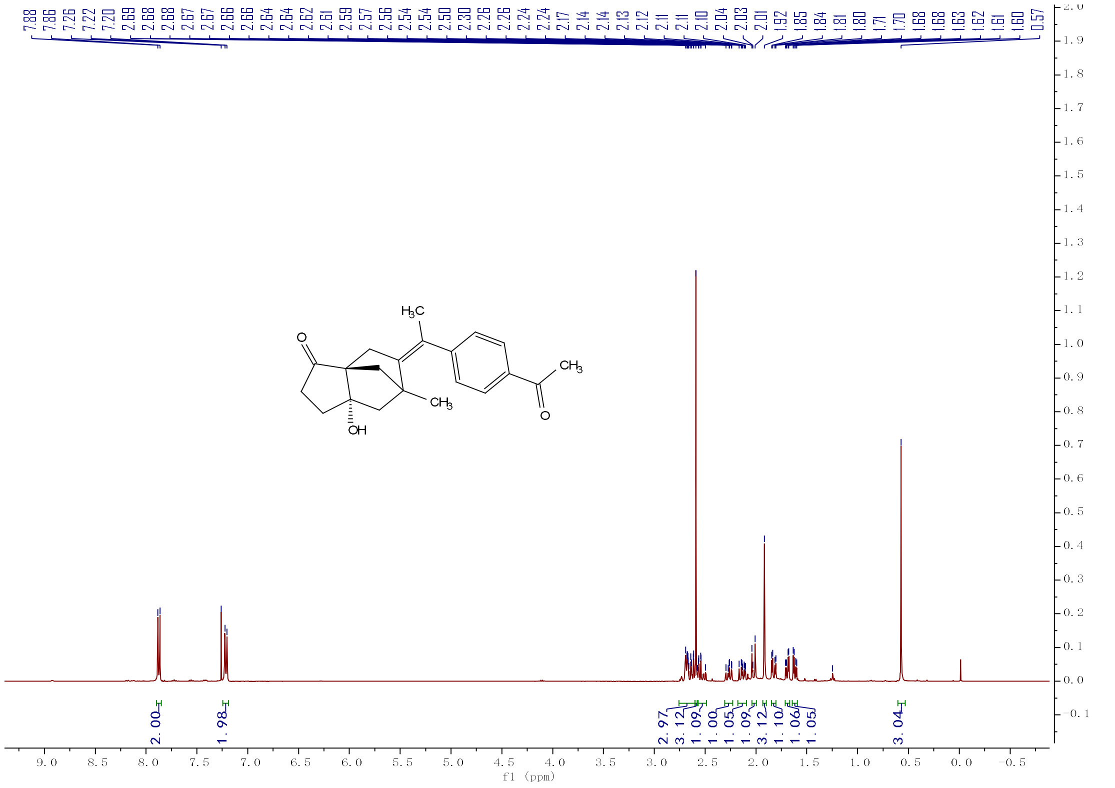

Supplementary Figure 115. ^{1}H NMR spectrum of 3am

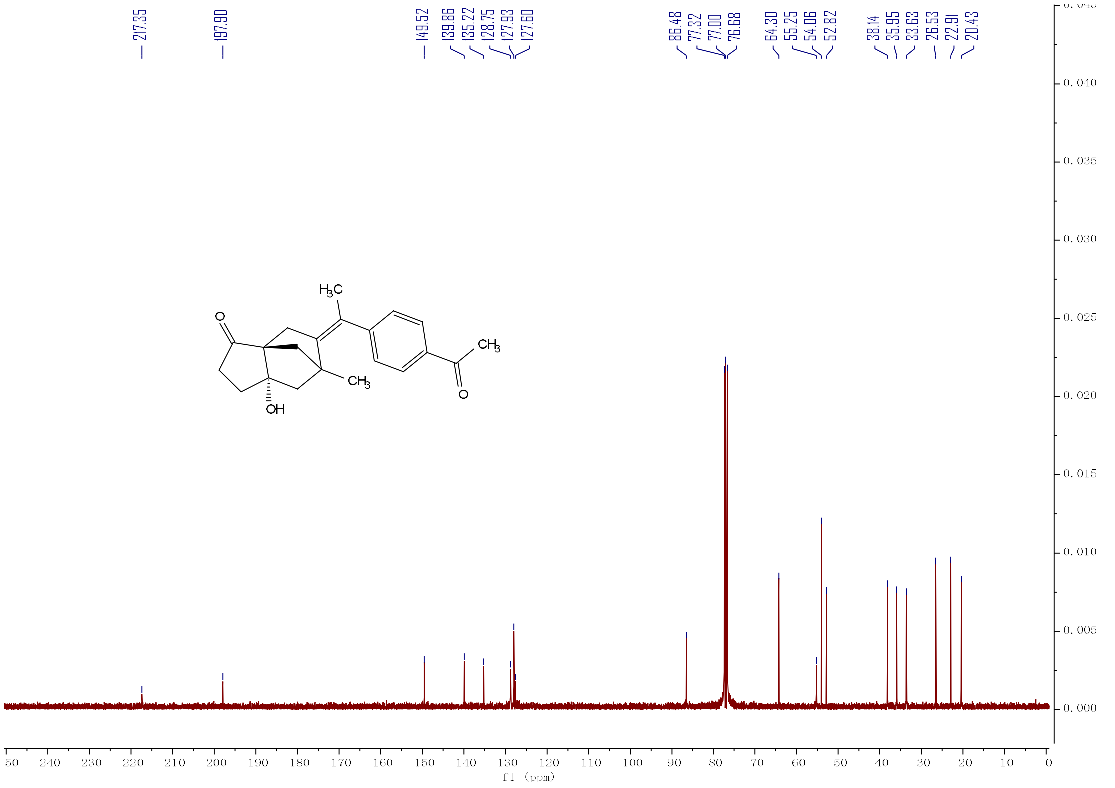

Supplementary Figure 116. ^{13}C NMR spectrum of 3am

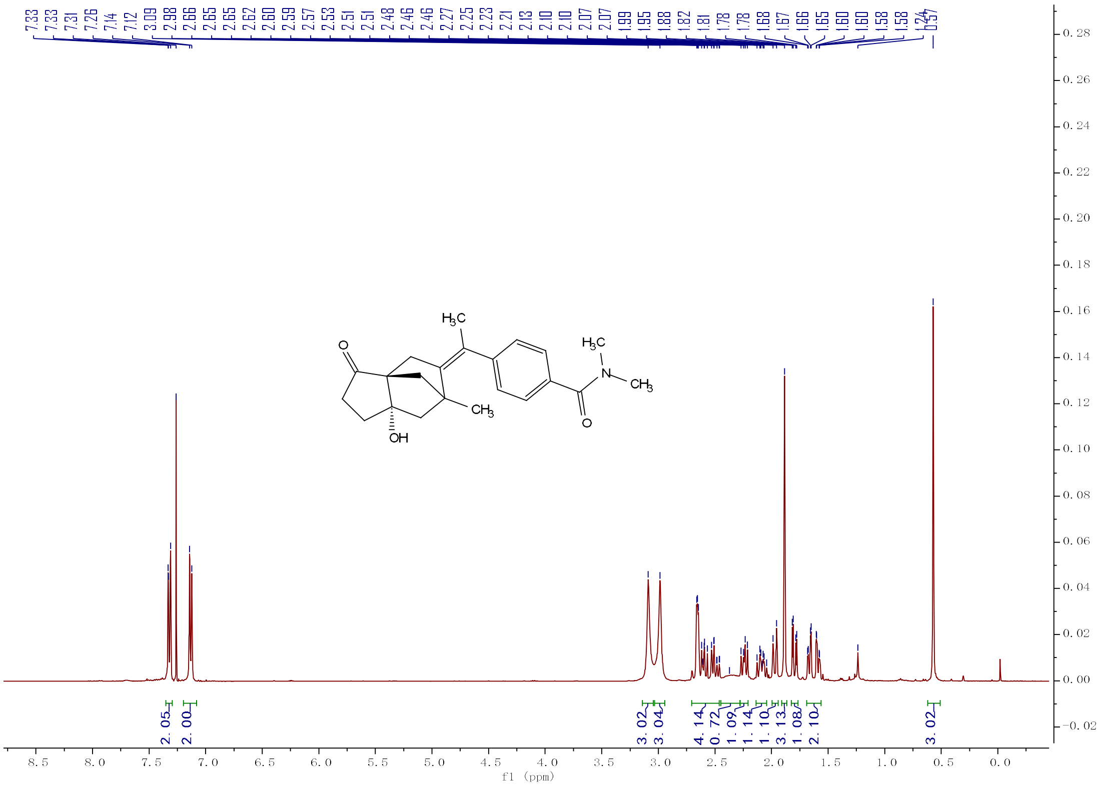

Supplementary Figure 117. ^{1}H NMR spectrum of 3an

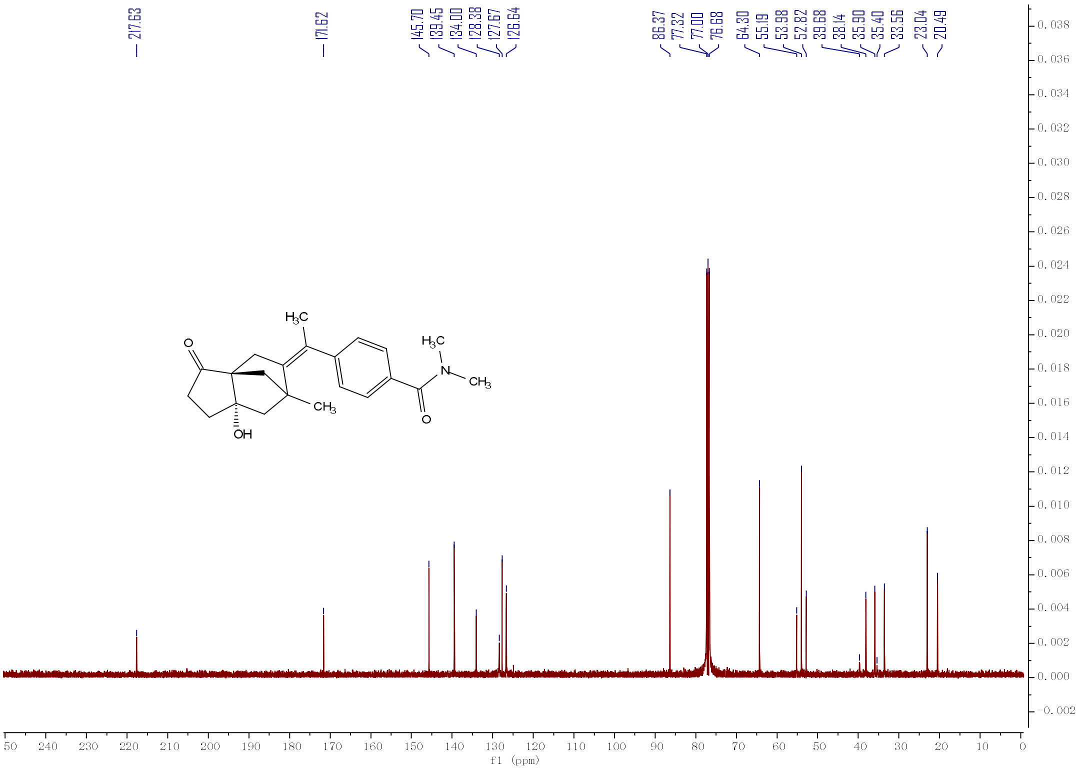

Supplementary Figure 118. ^{13}C NMR spectrum of 3an

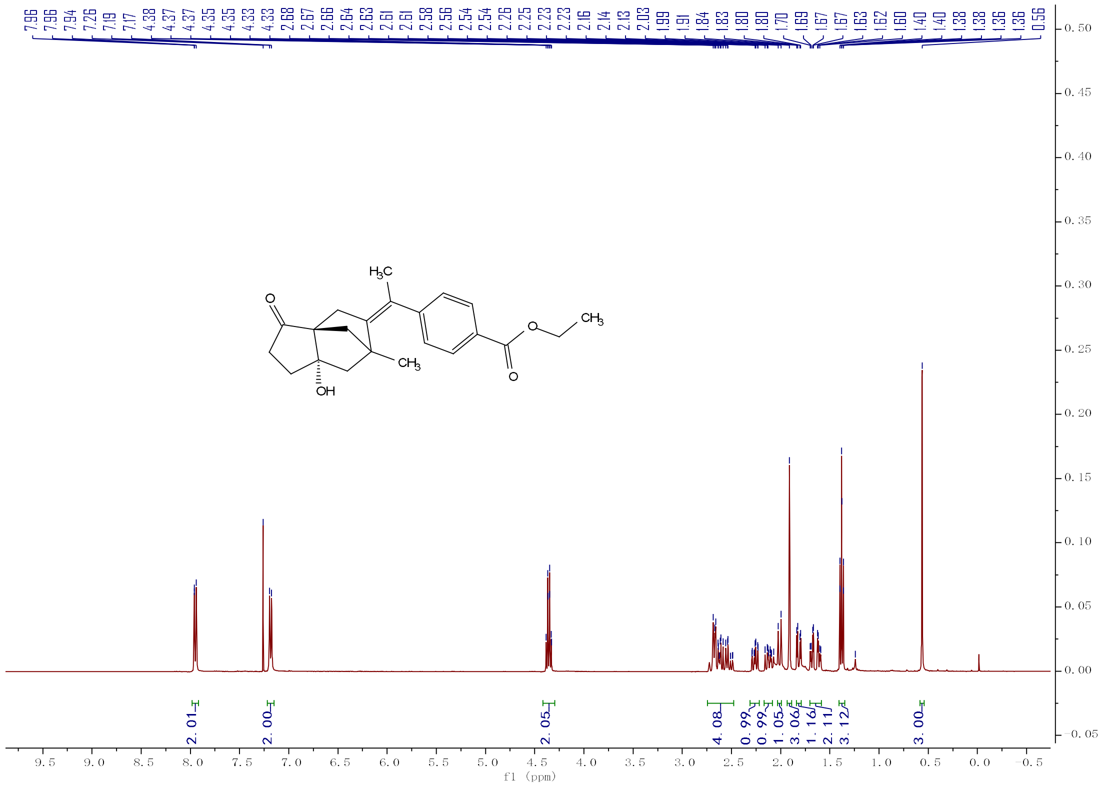

Supplementary Figure 119. ^{1}H NMR spectrum of 3ao

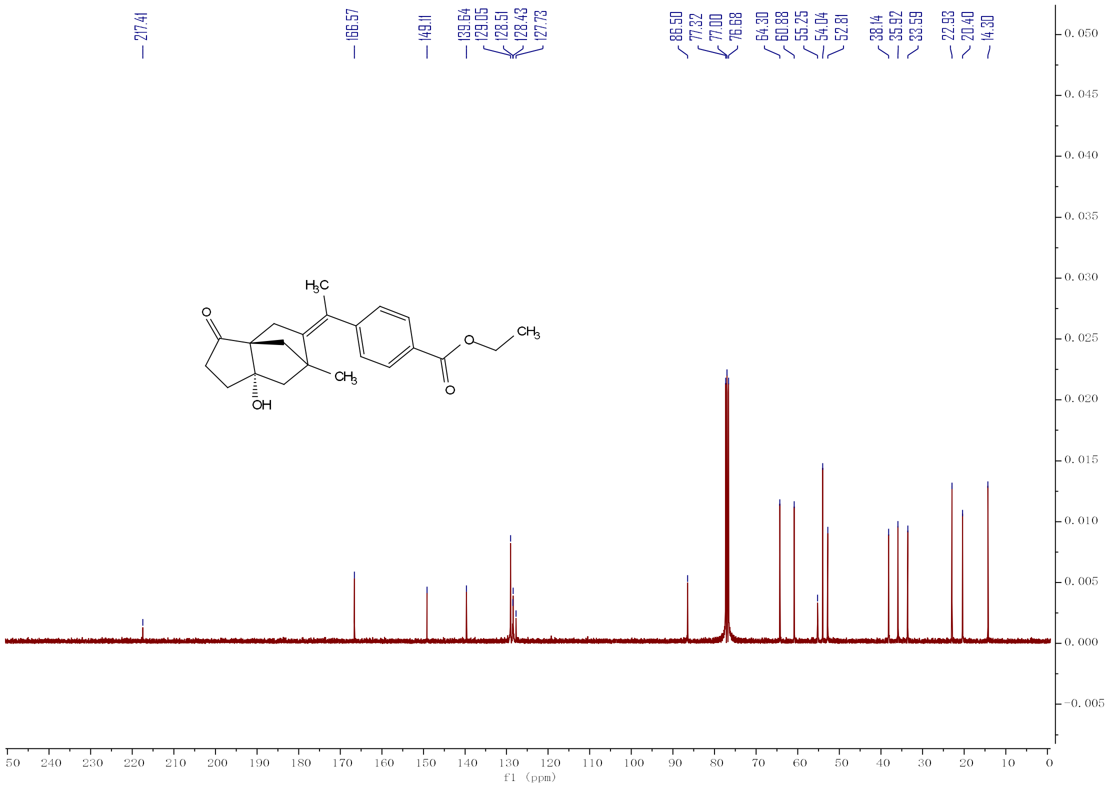

Supplementary Figure 120. ^{13}C NMR spectrum of 3ao

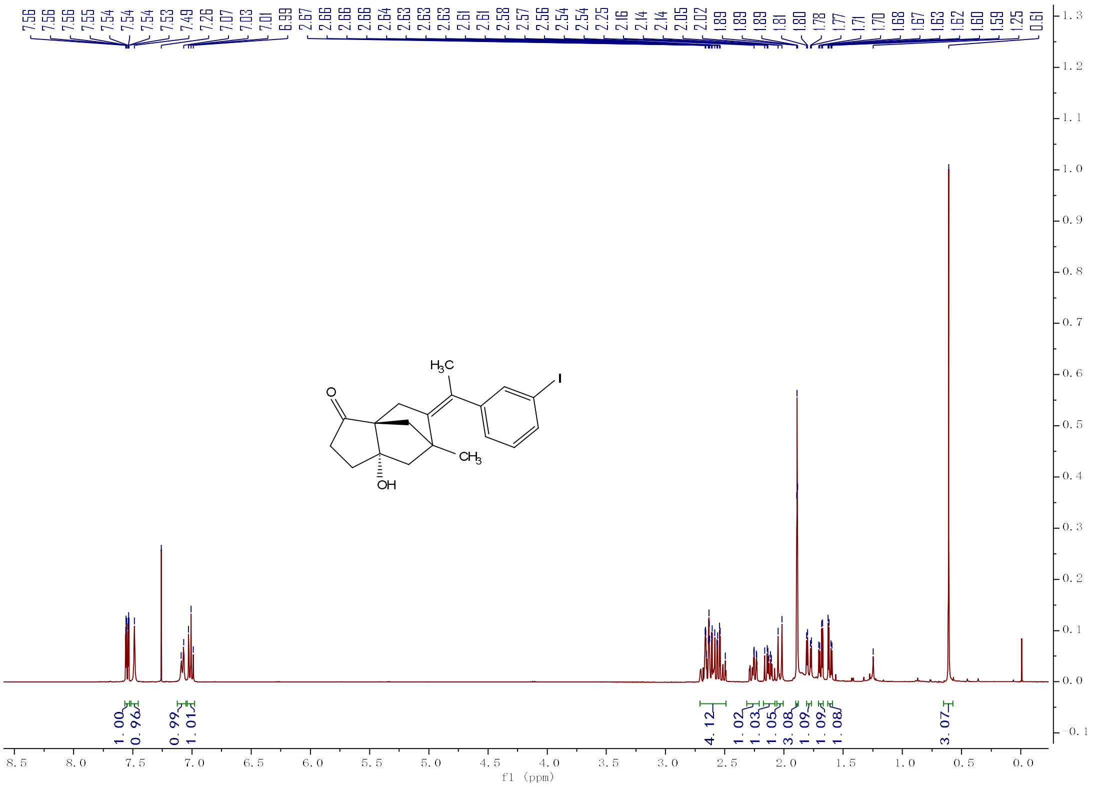

Supplementary Figure 121. ^{1}H NMR spectrum of 3ap

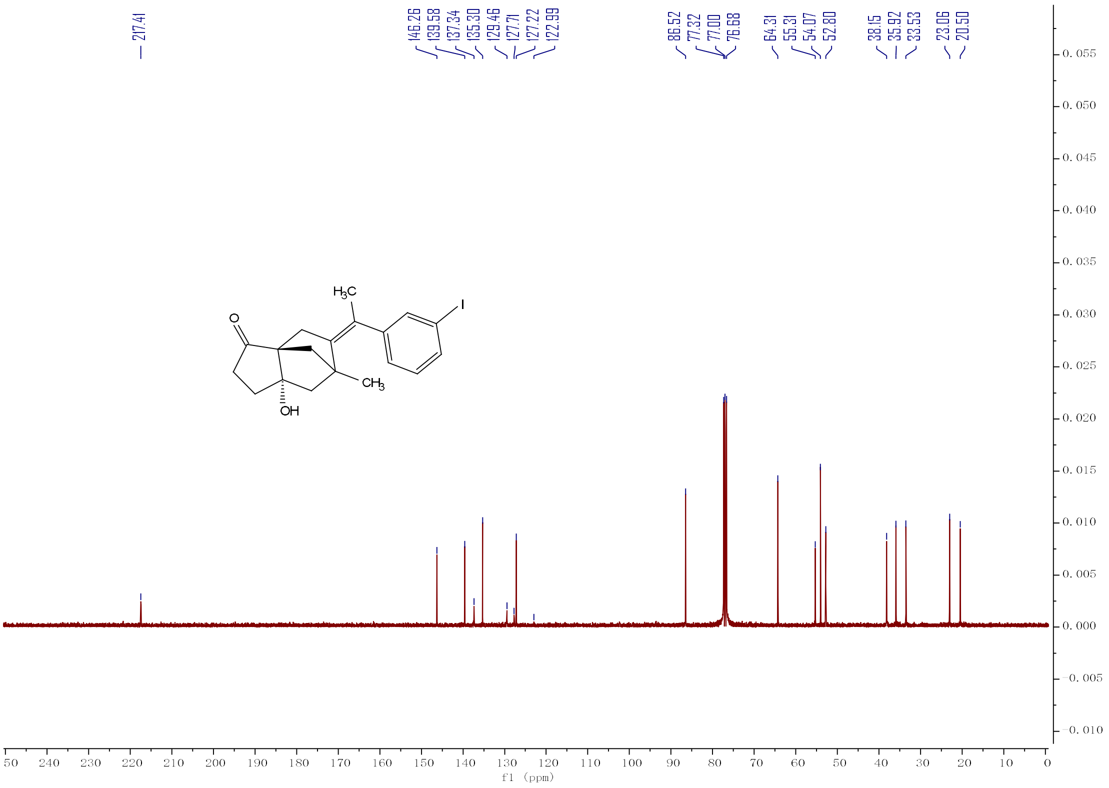

Supplementary Figure 122. ^{13}C NMR spectrum of 3ap

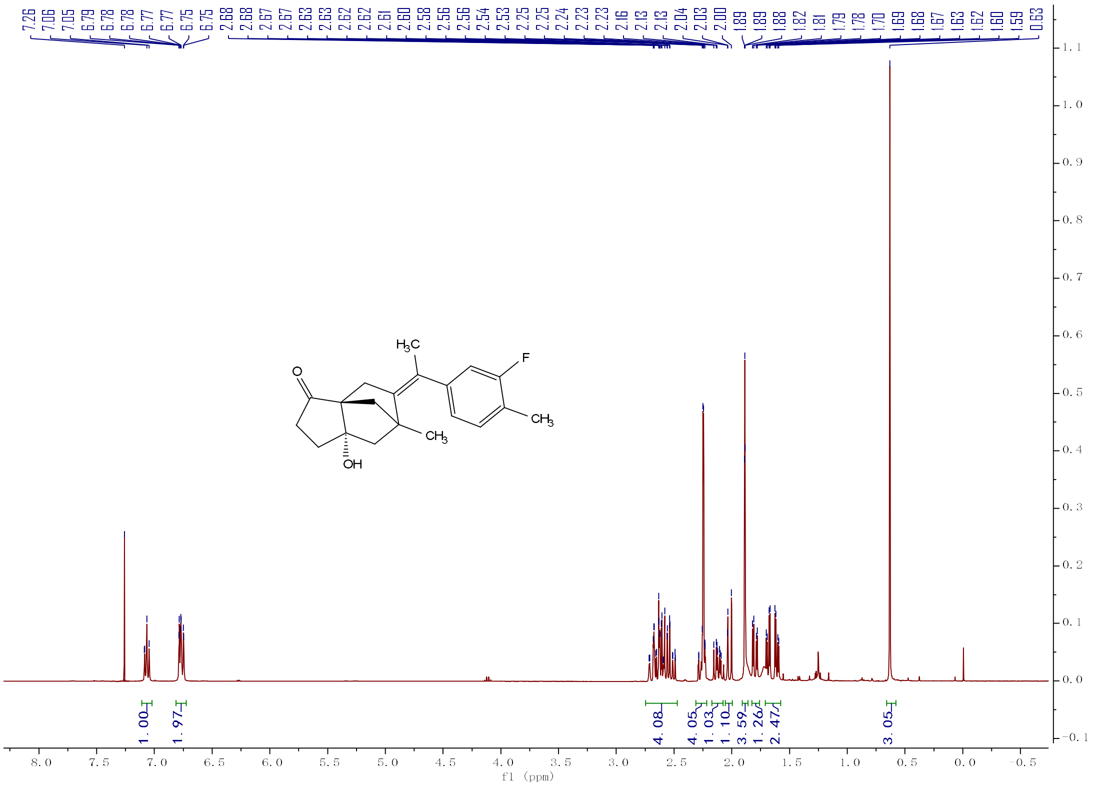

Supplementary Figure 123. ^{1}H NMR spectrum of 3aq

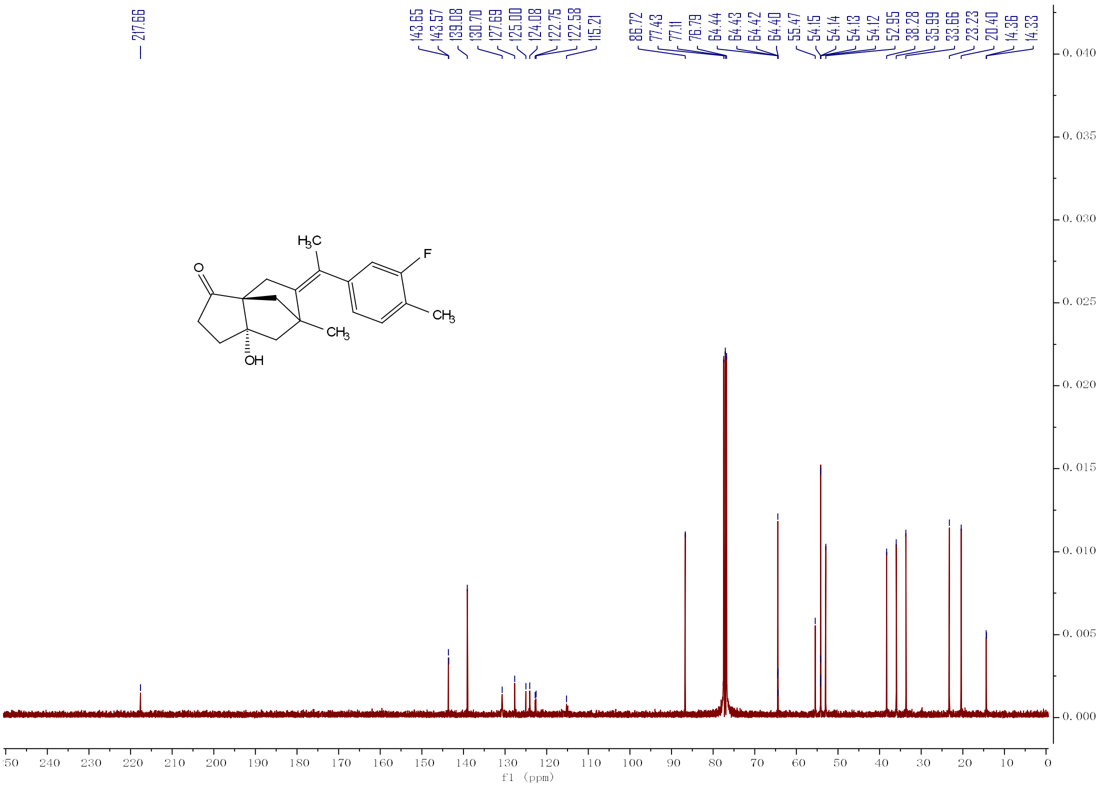

Supplementary Figure 124. ^{13}C NMR spectrum of 3aq

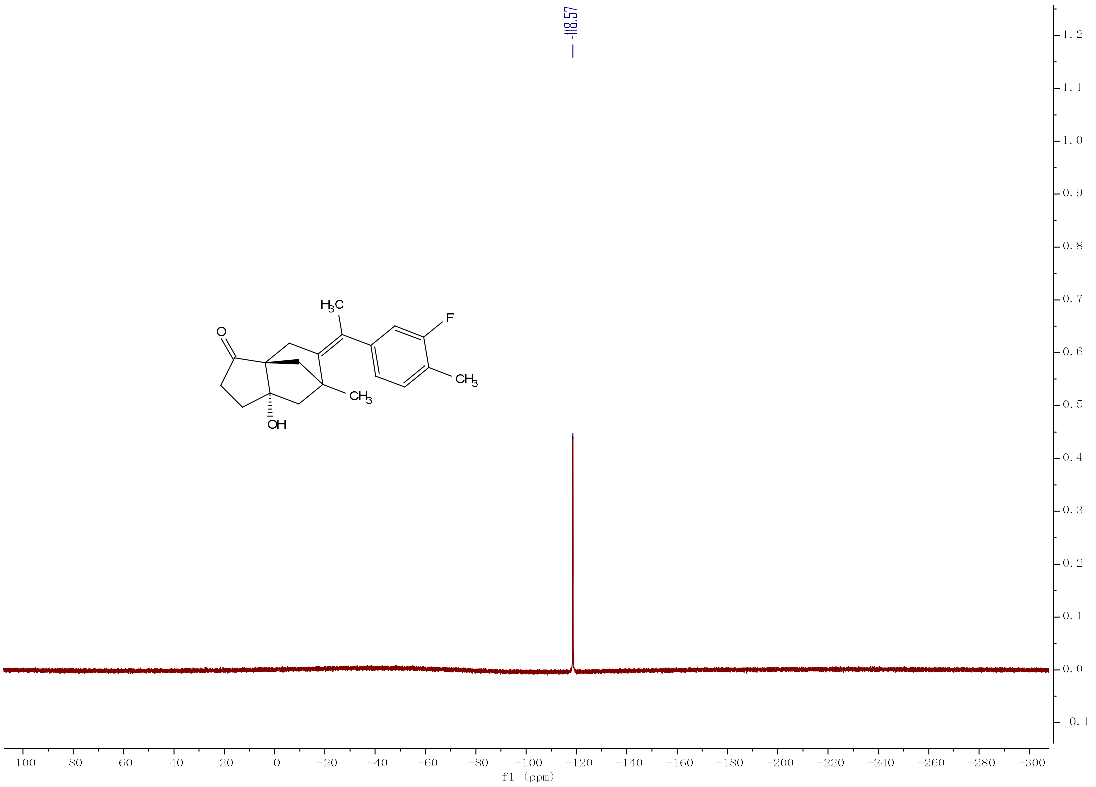

Supplementary Figure 125. ^{19}F NMR spectrum of 3aq

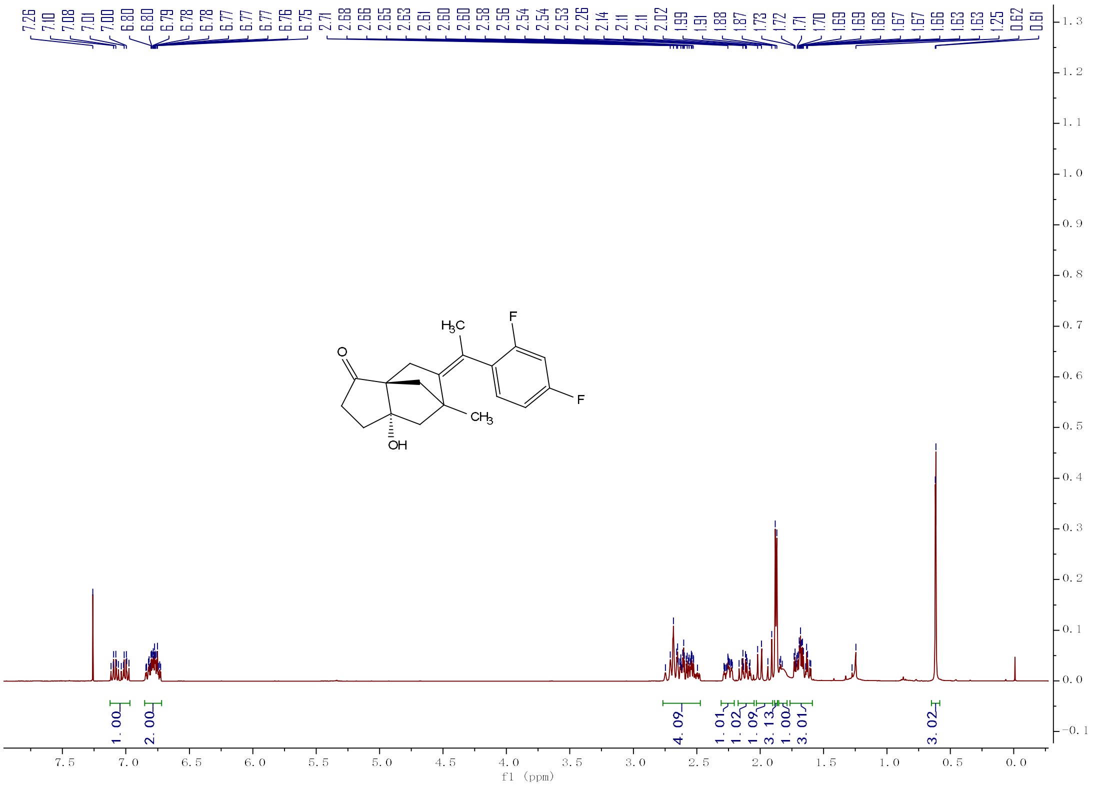

Supplementary Figure 126. ^{1}H NMR spectrum of 3ar

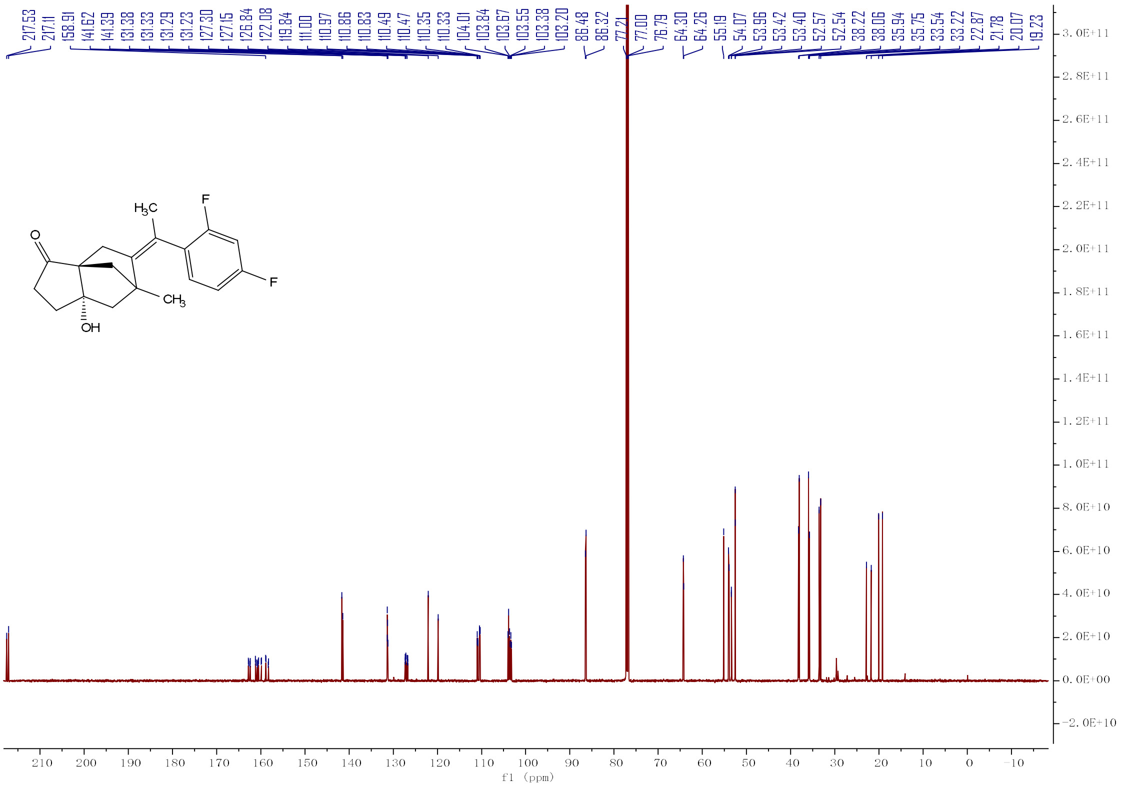

Supplementary Figure 127. ^{13}C NMR spectrum of 3ar

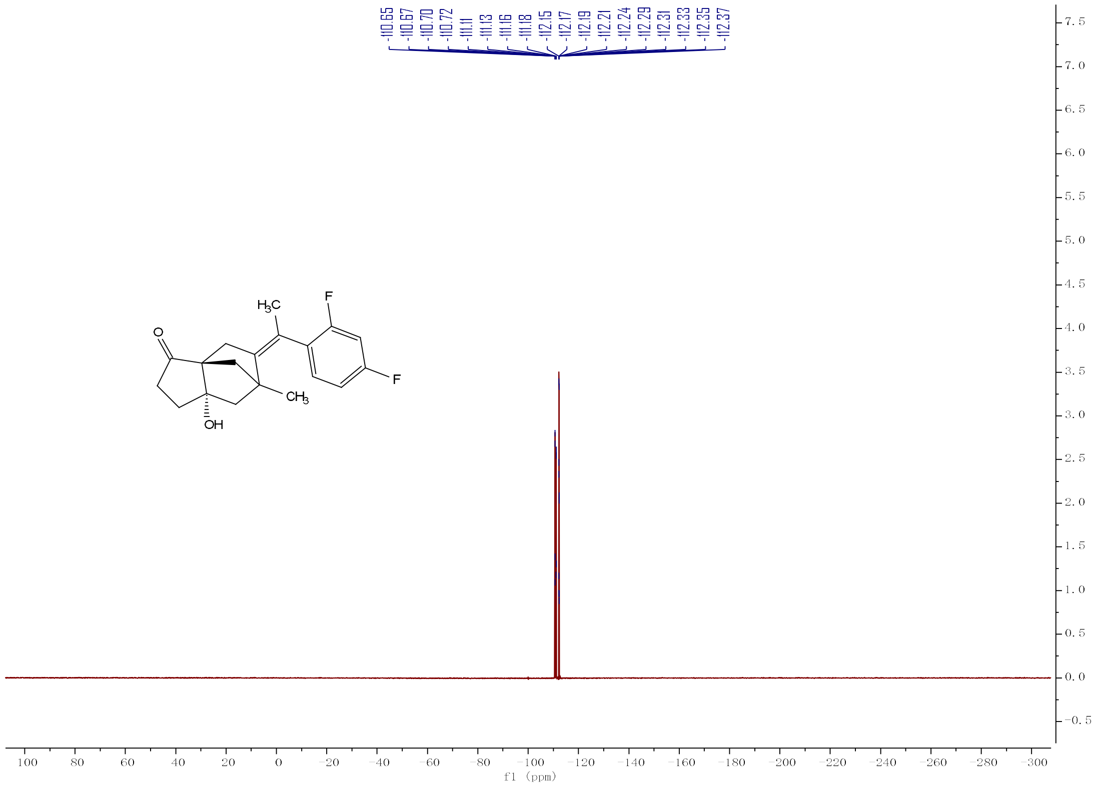

Supplementary Figure 128. ^{19}F NMR spectrum of 3ar

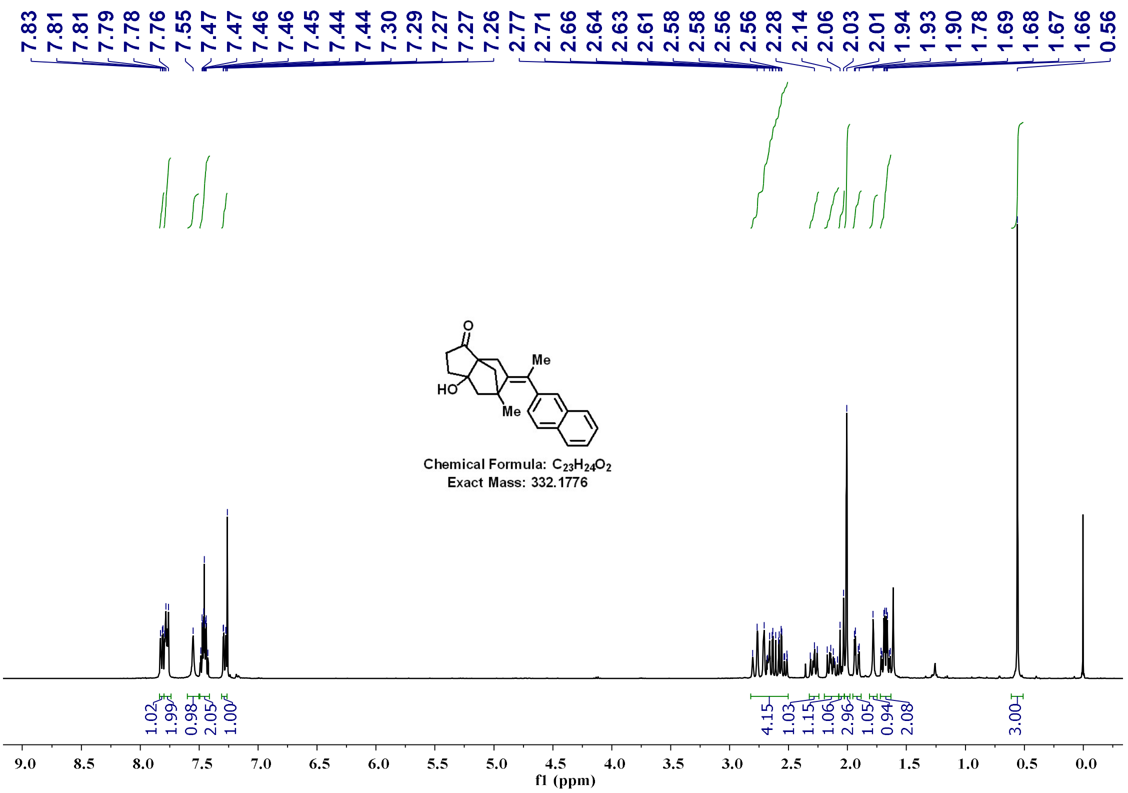

Supplementary Figure 129. ^{1}H NMR spectrum of 3as

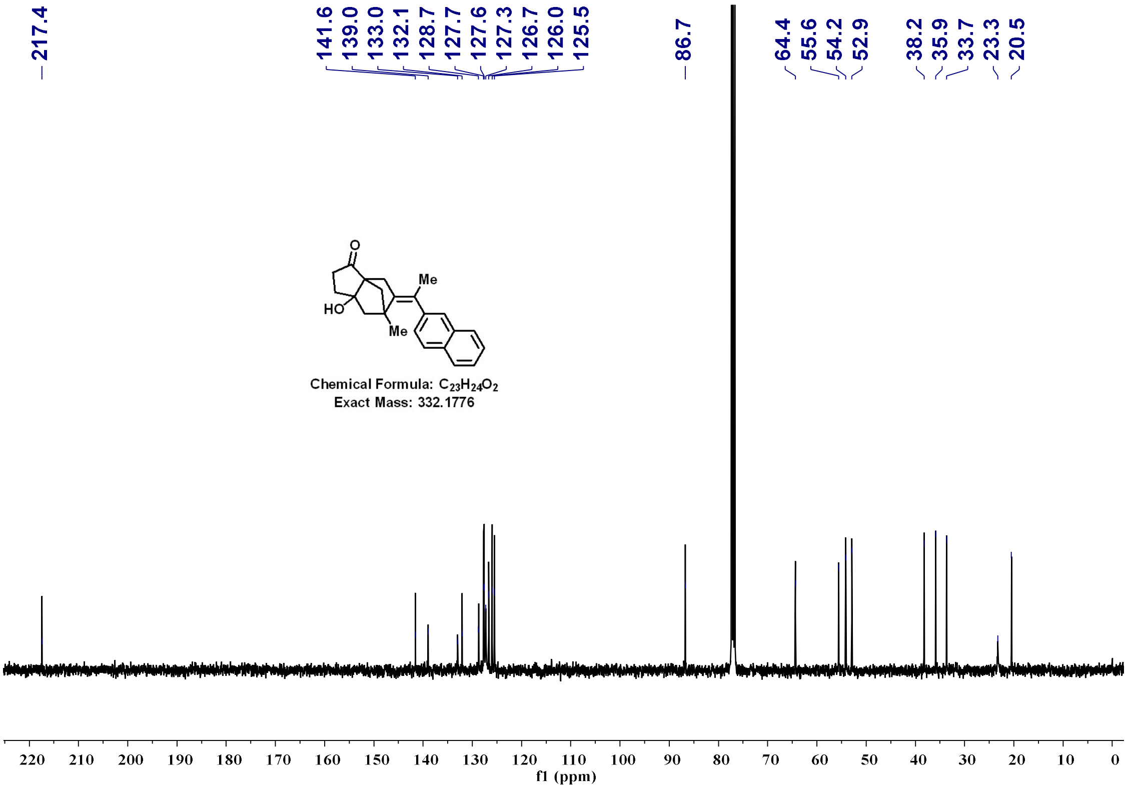

Supplementary Figure 130. ^{13}C NMR spectrum of 3as

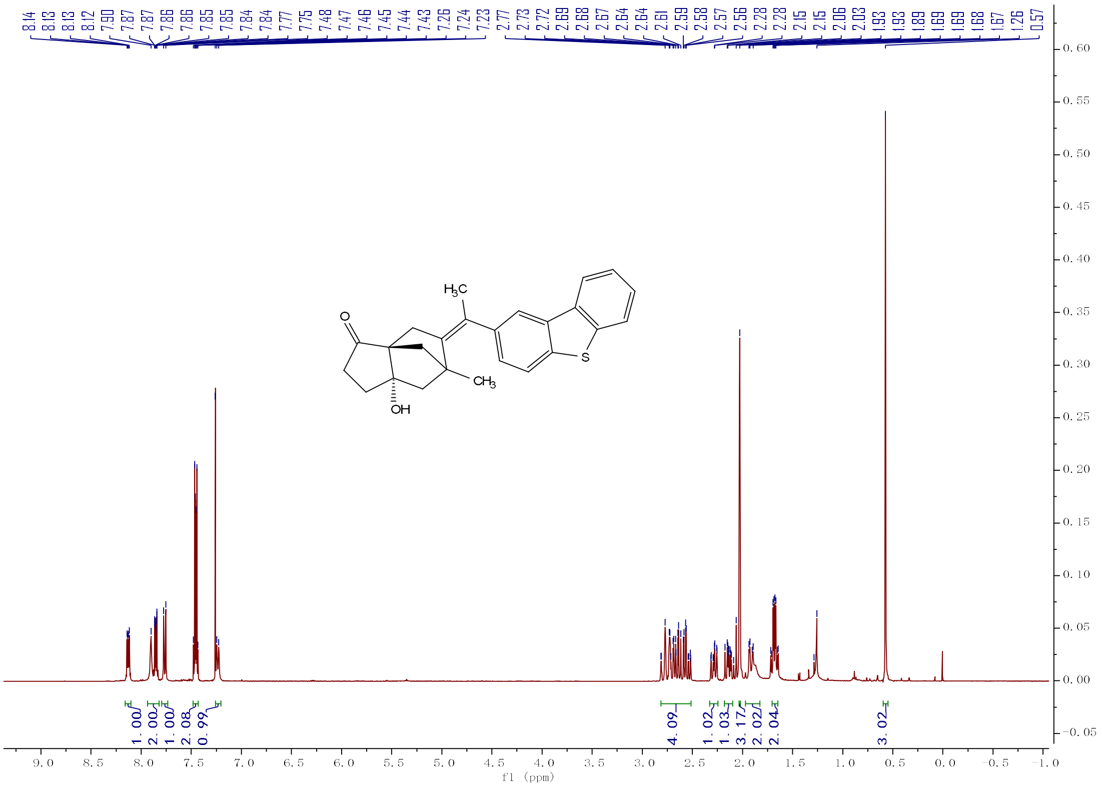

Supplementary Figure 131. ^{1}H NMR spectrum of 3at

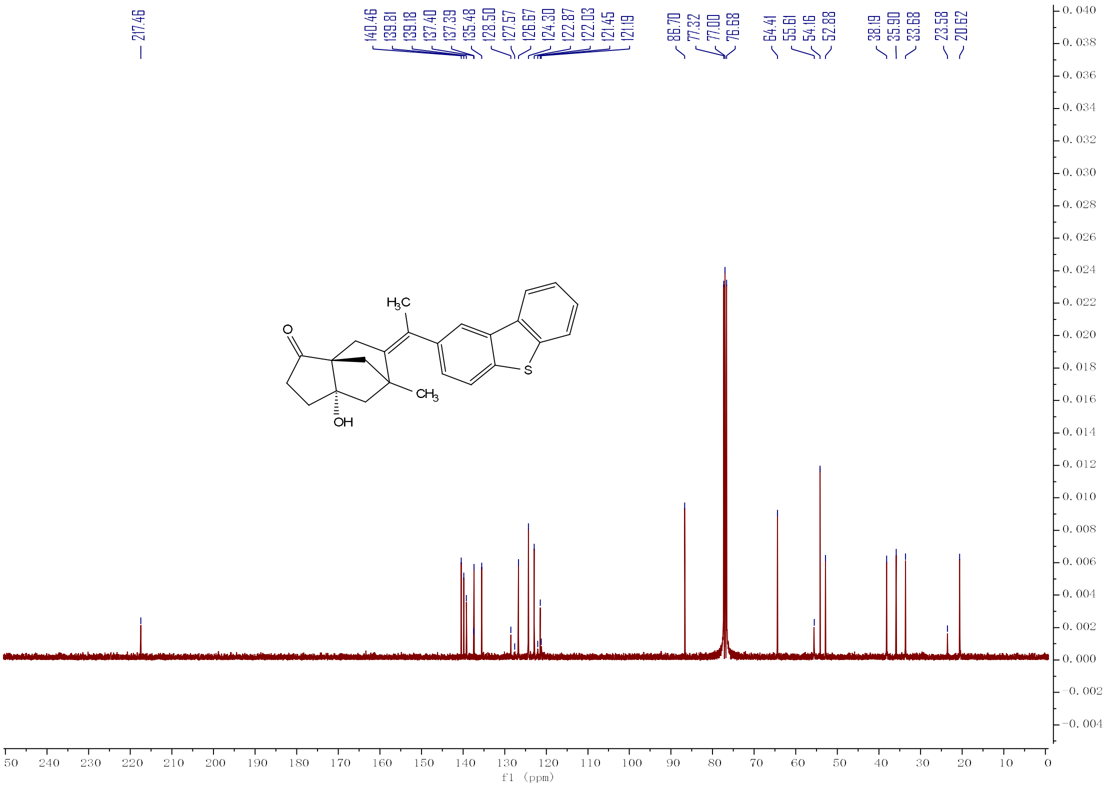

Supplementary Figure 132. ^{13}C NMR spectrum of 3at

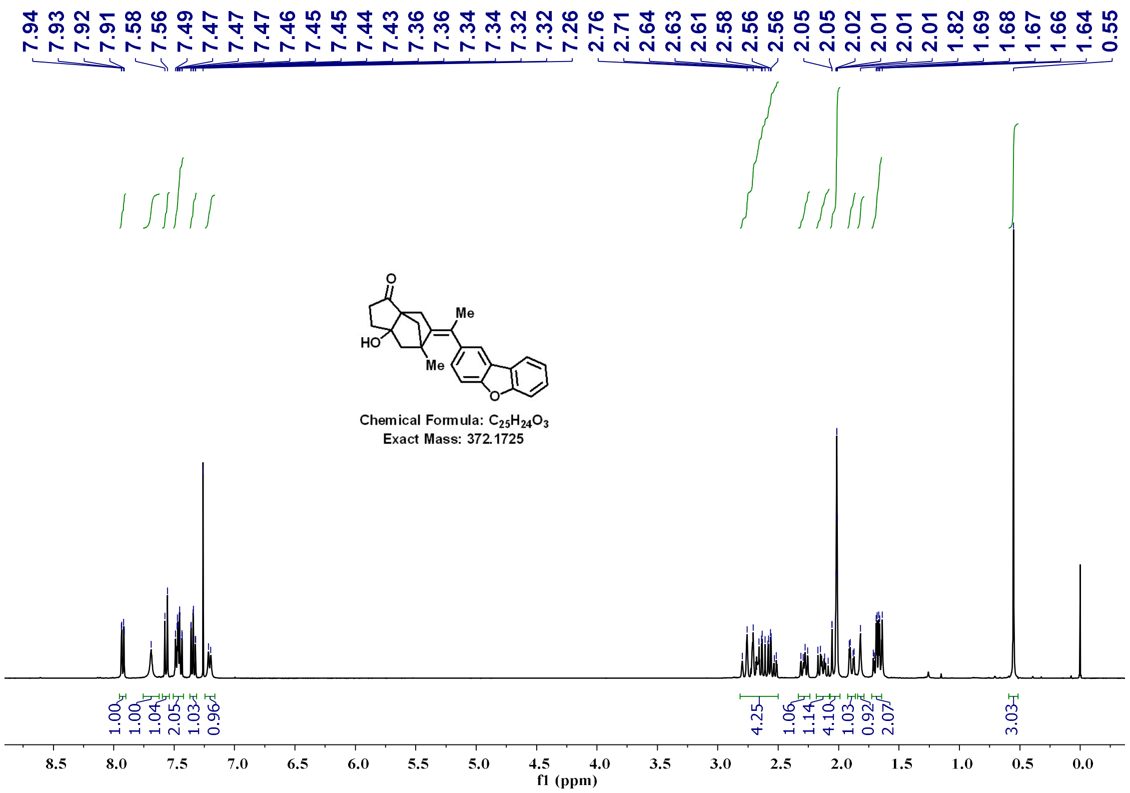

Supplementary Figure 133. ^{1}H NMR spectrum of 3au

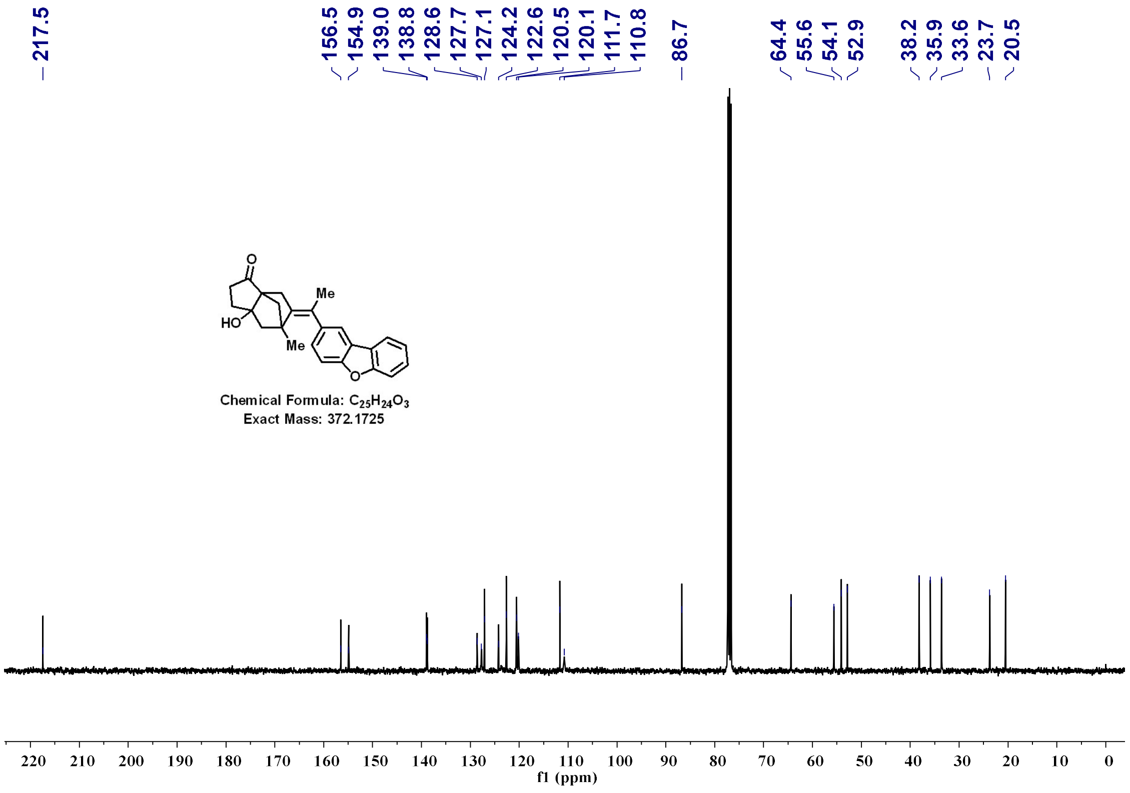

Supplementary Figure 134. ^{13}C NMR spectrum of 3au

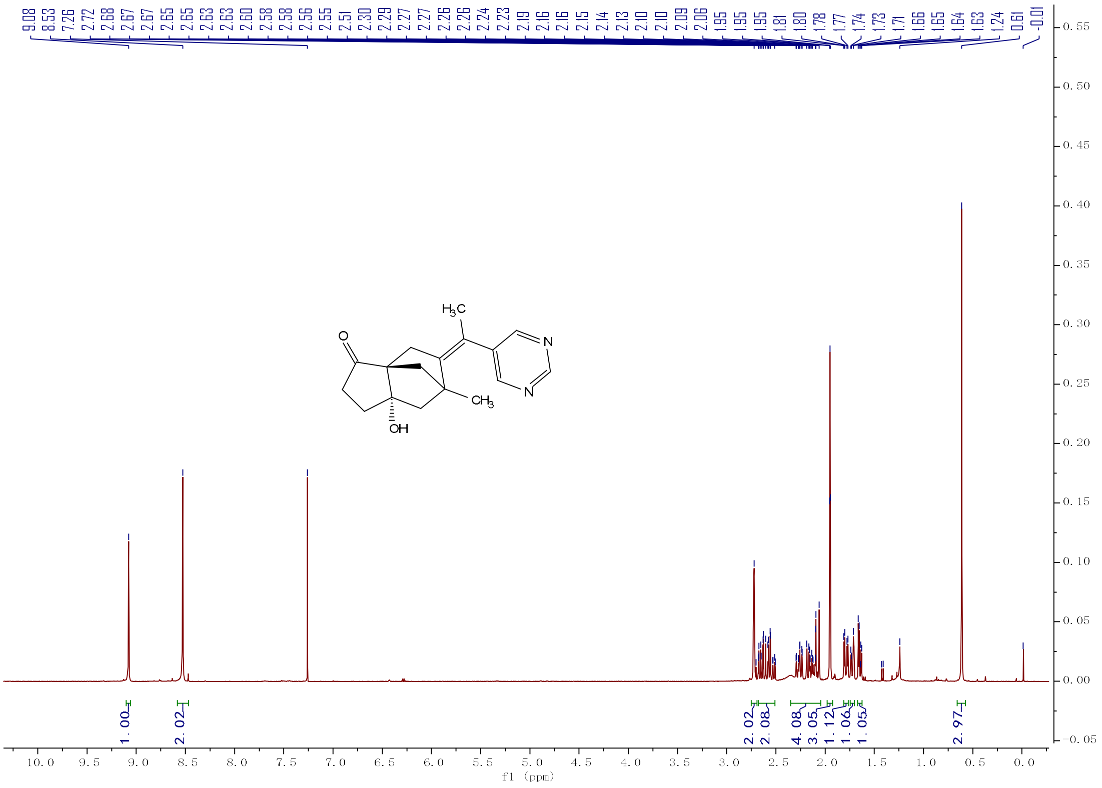

Supplementary Figure 135. ^{1}H NMR spectrum of 3av

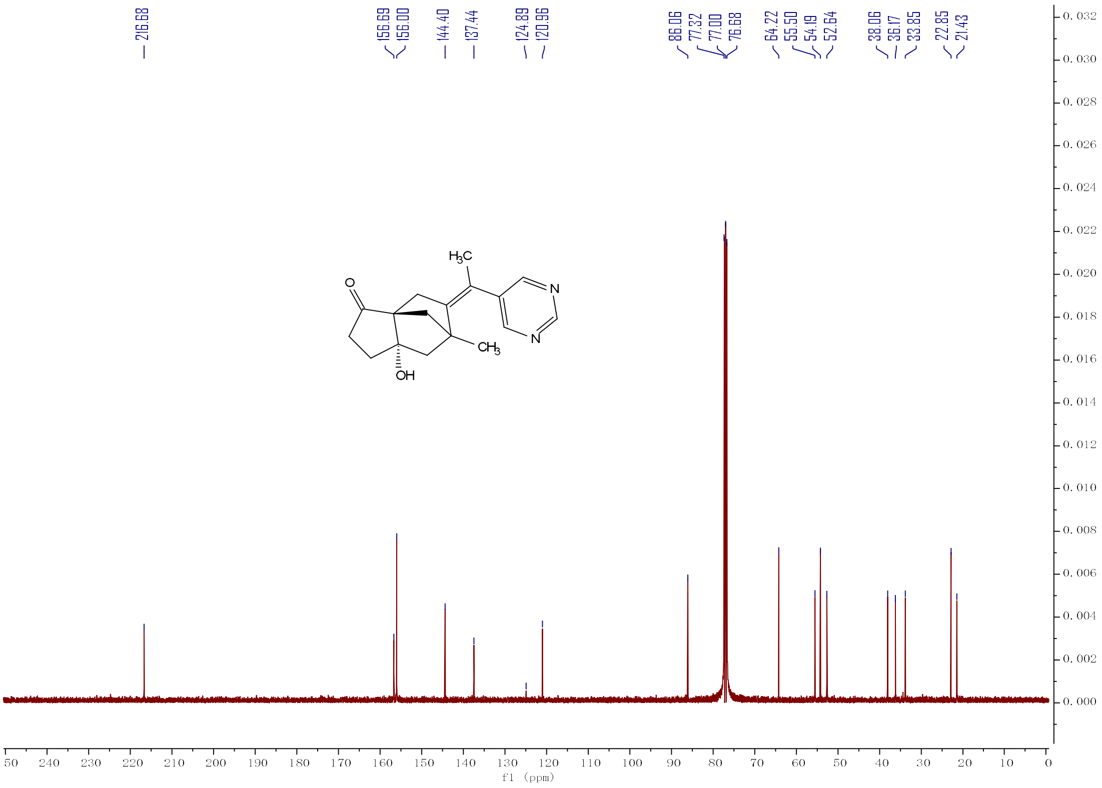

Supplementary Figure 136. ^{13}C NMR spectrum of 3av

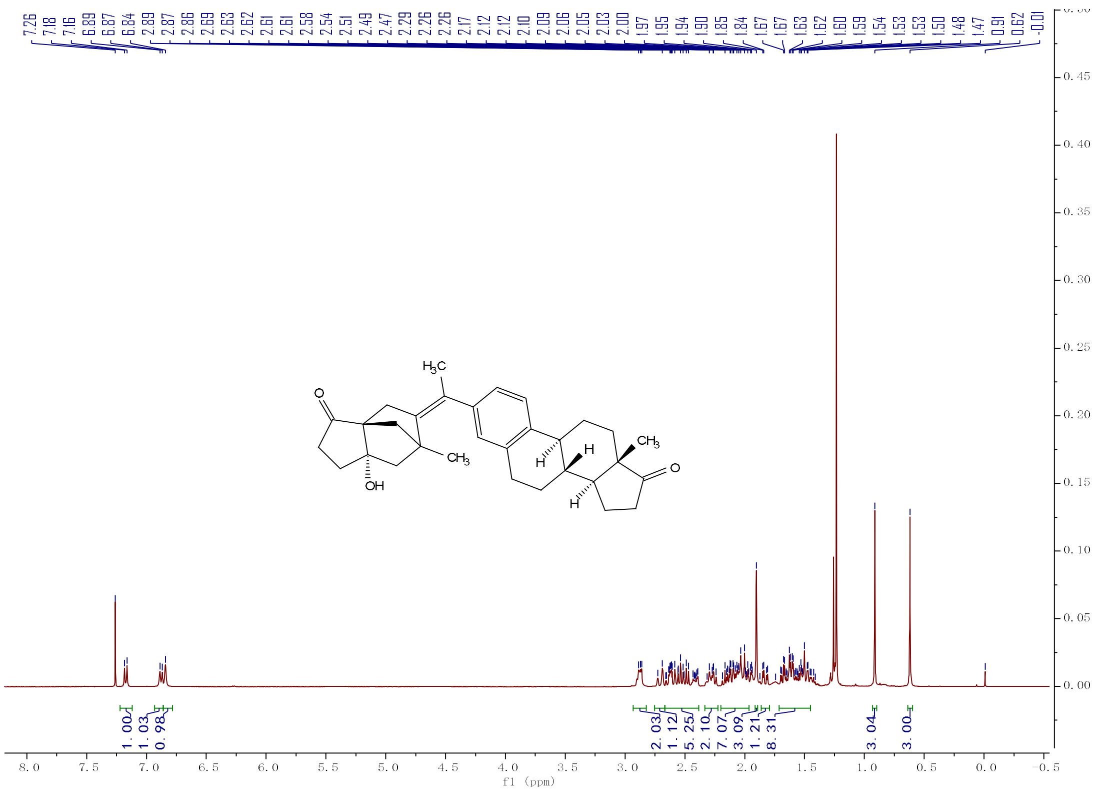

Supplementary Figure 137. ^{1}H NMR spectrum of 3aw

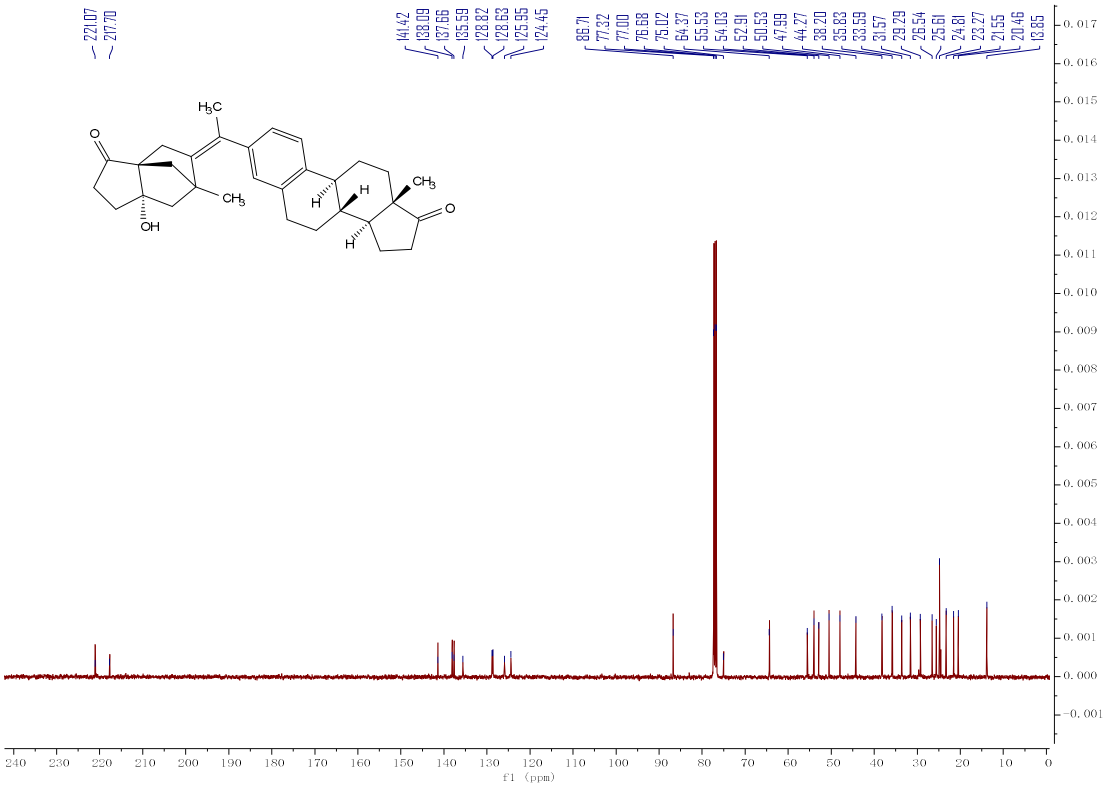

Supplementary Figure 138. ^{13}C NMR spectrum of 3aw

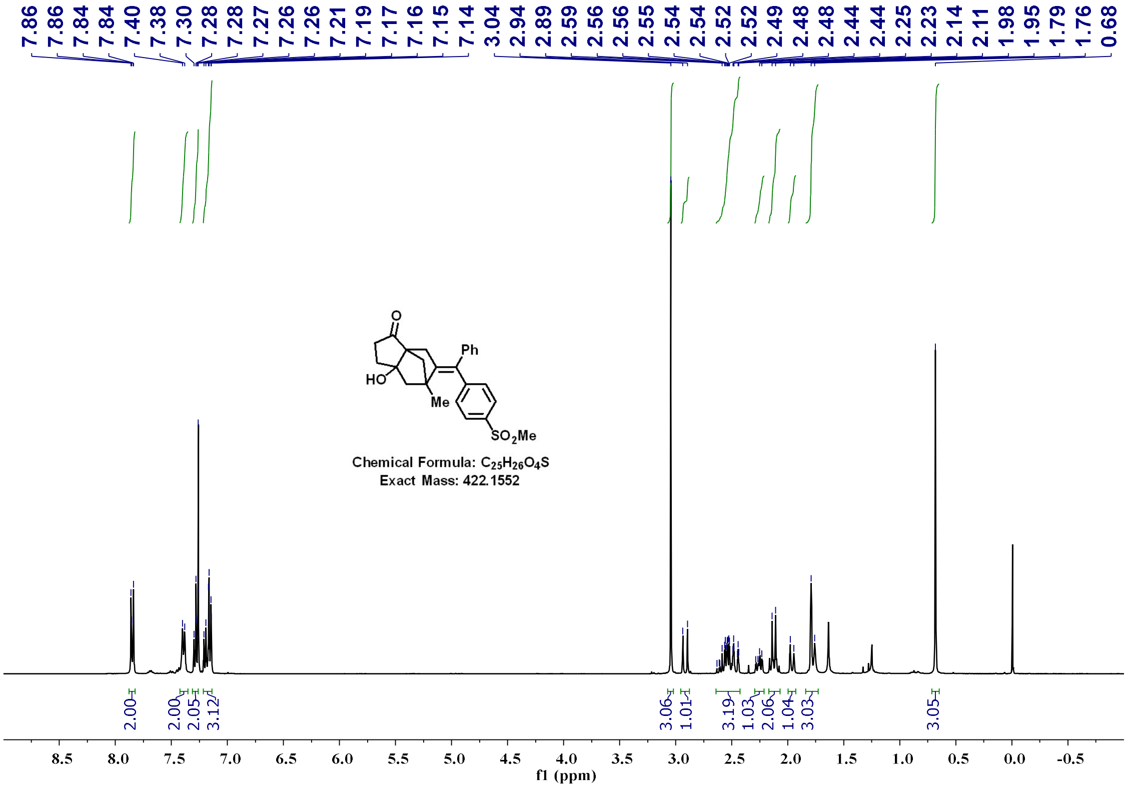

Supplementary Figure 139. ^{1}H NMR spectrum of 3bi

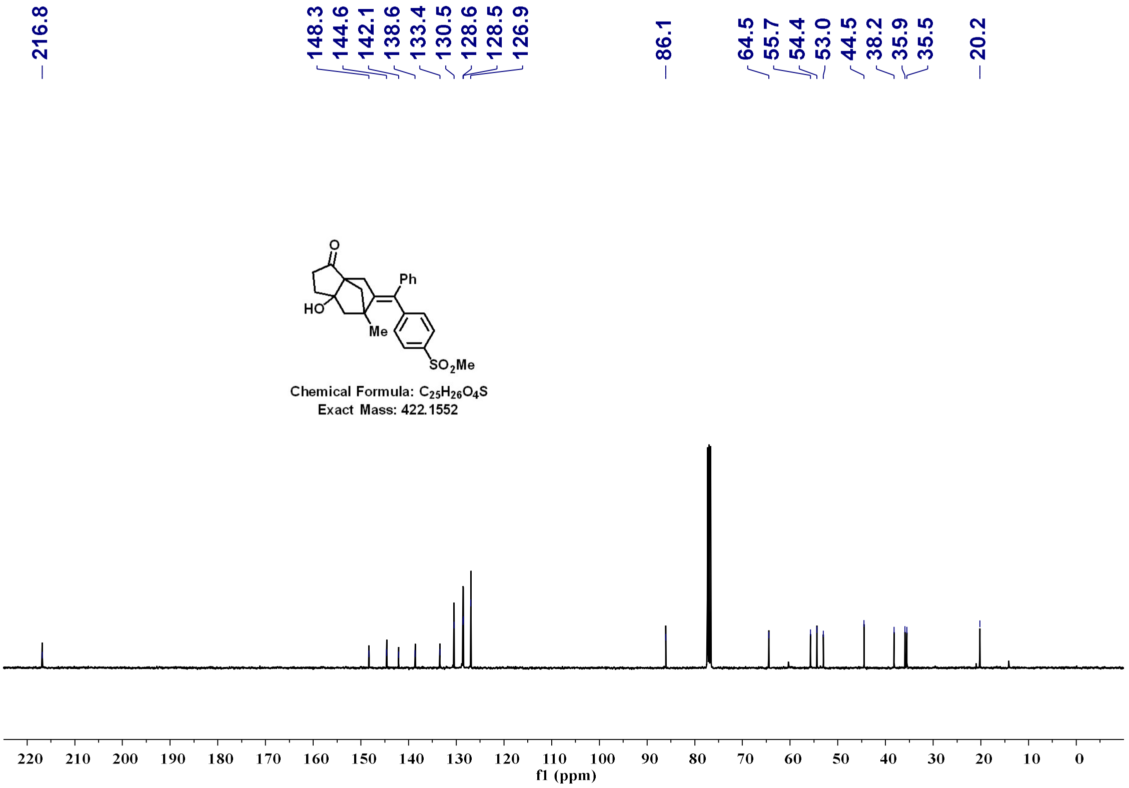

Supplementary Figure 140. ^{13}C NMR spectrum of 3bi

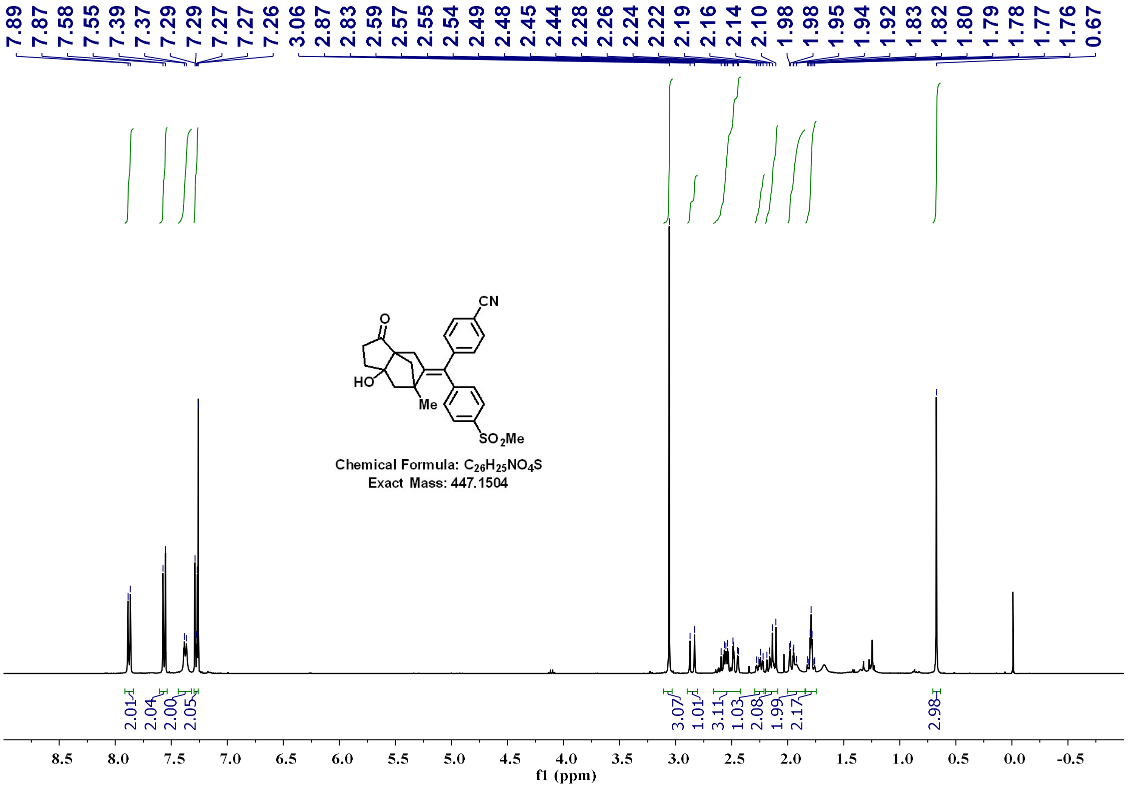

Supplementary Figure 141. ^{1}H NMR spectrum of 3ci

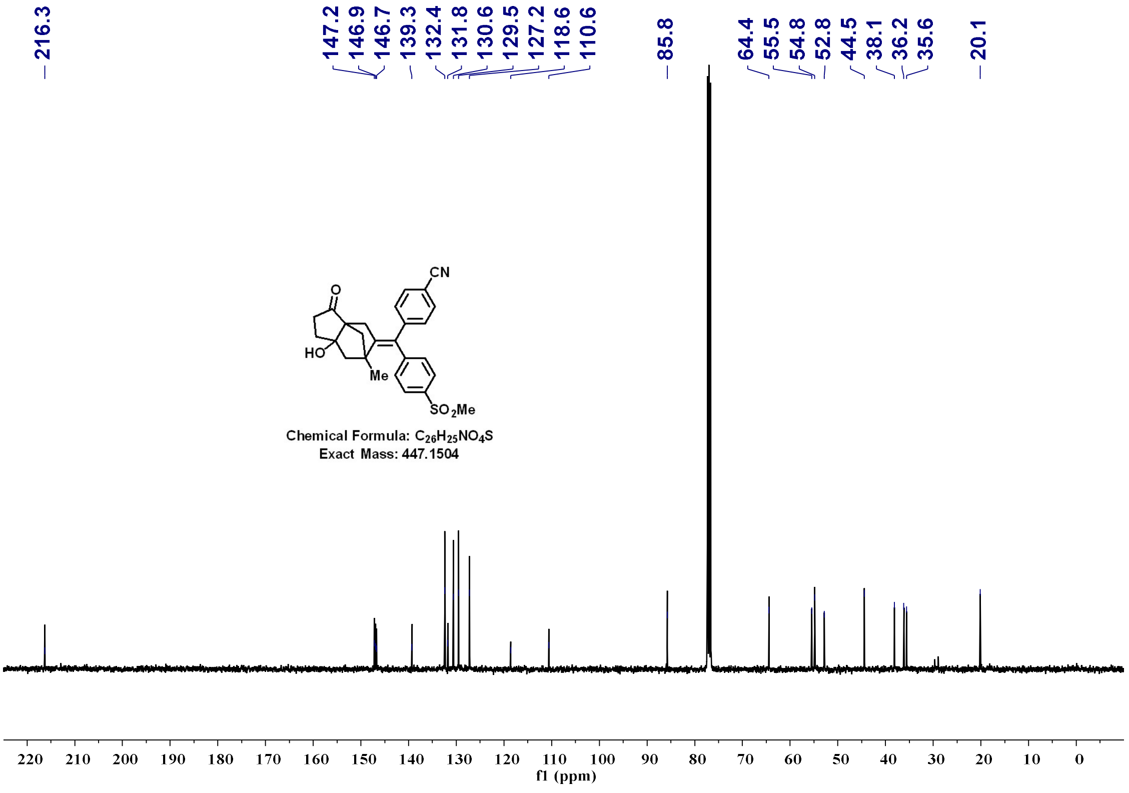

Supplementary Figure 142. ^{13}C NMR spectrum of 3ci

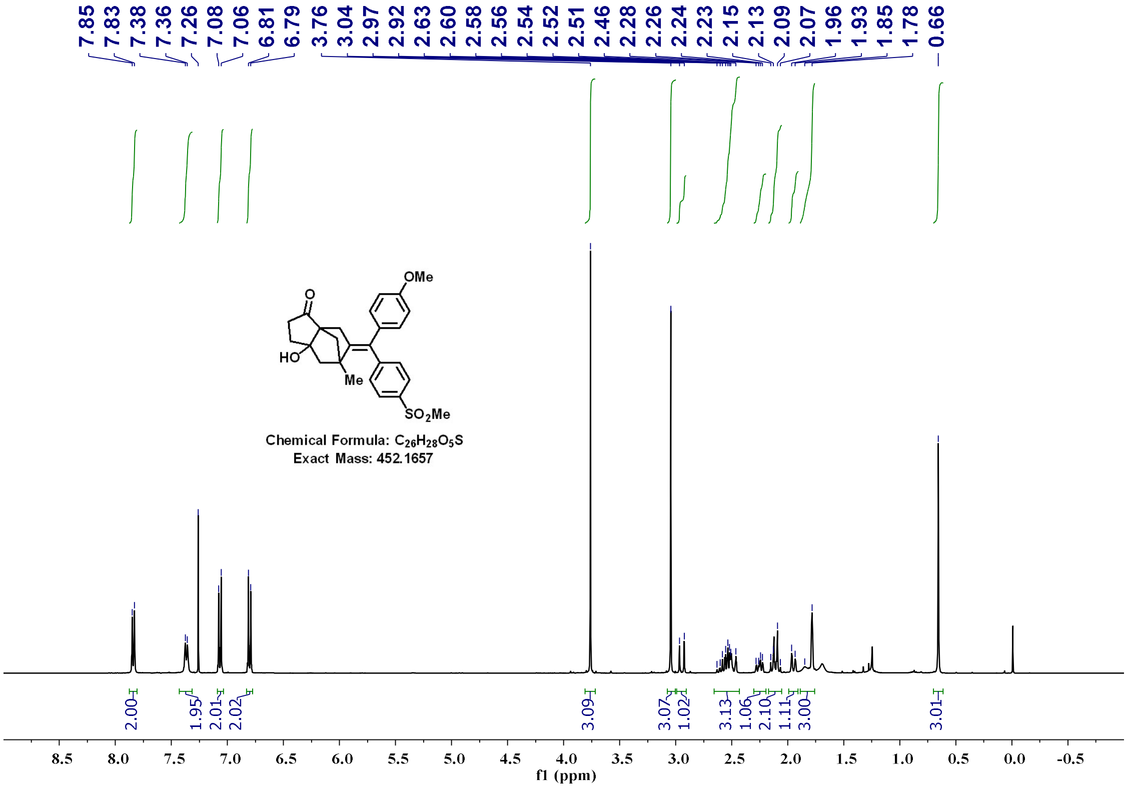

Supplementary Figure 143. ^{1}**H NMR spectrum of 3di**

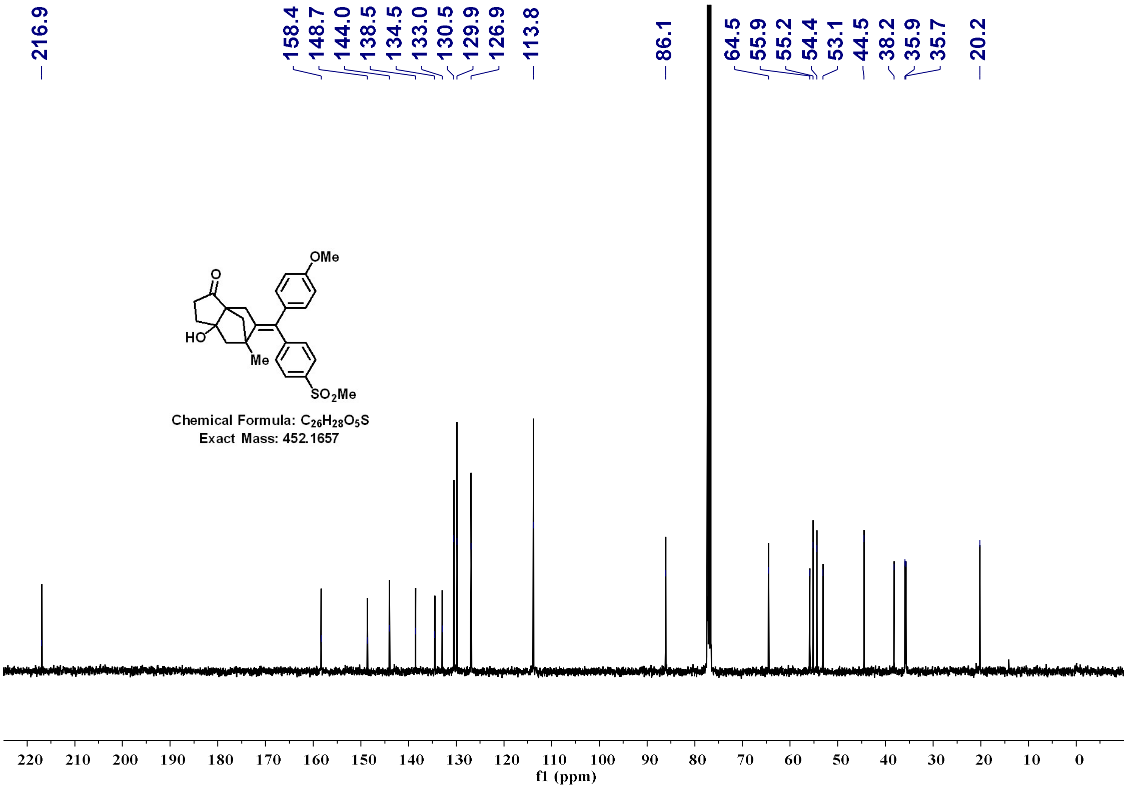

Supplementary Figure 144. ^{13}**C NMR spectrum of 3di**

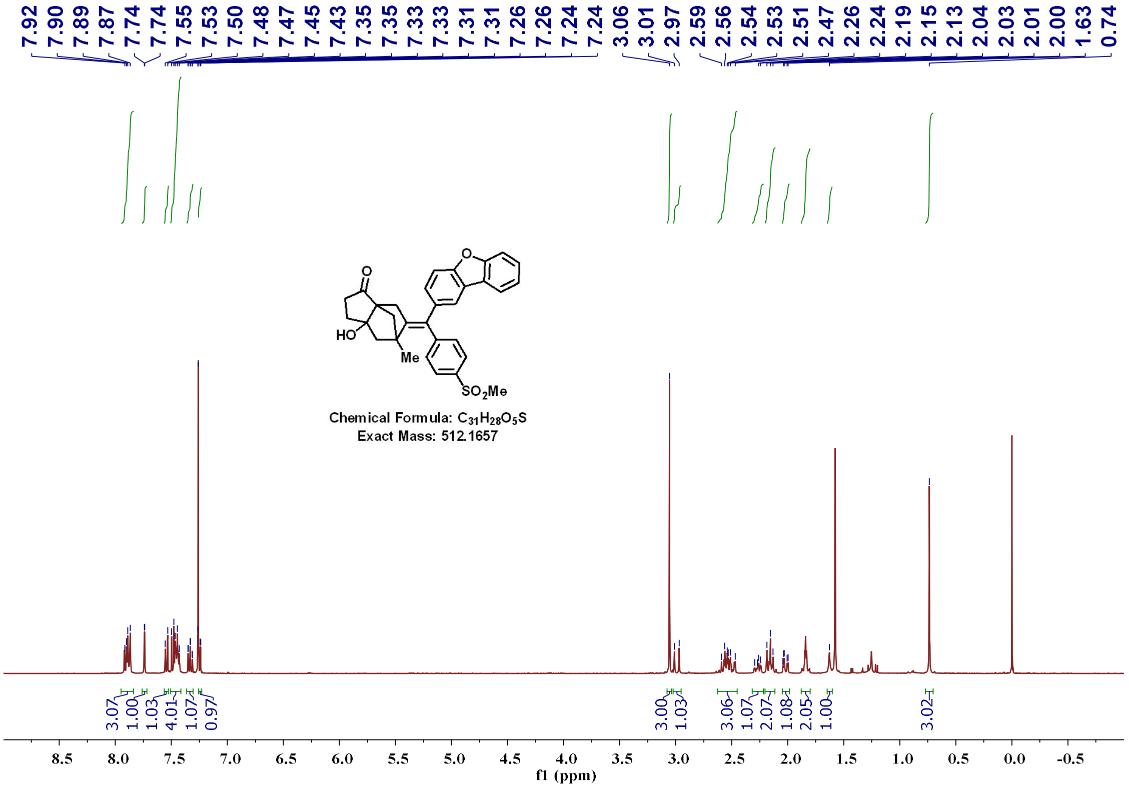

Supplementary Figure 145. ^{1}H NMR spectrum of 3ei

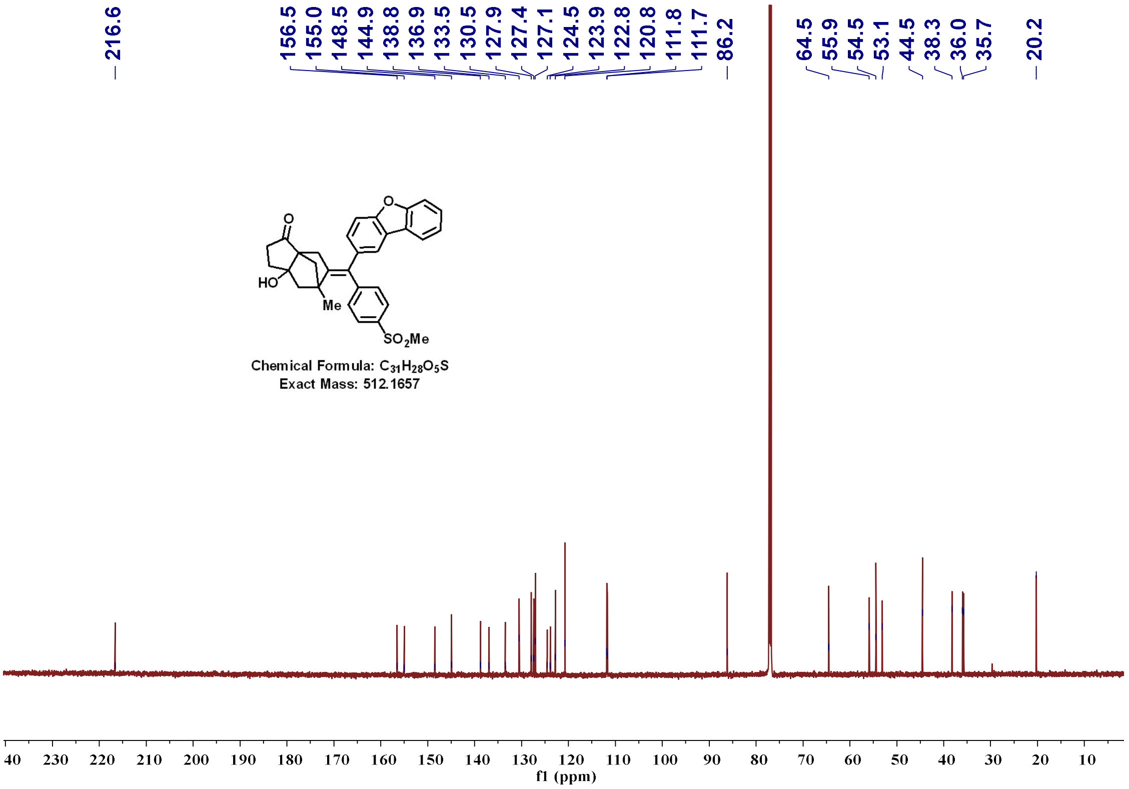

Supplementary Figure 146. ^{13}C NMR spectrum of 3ei

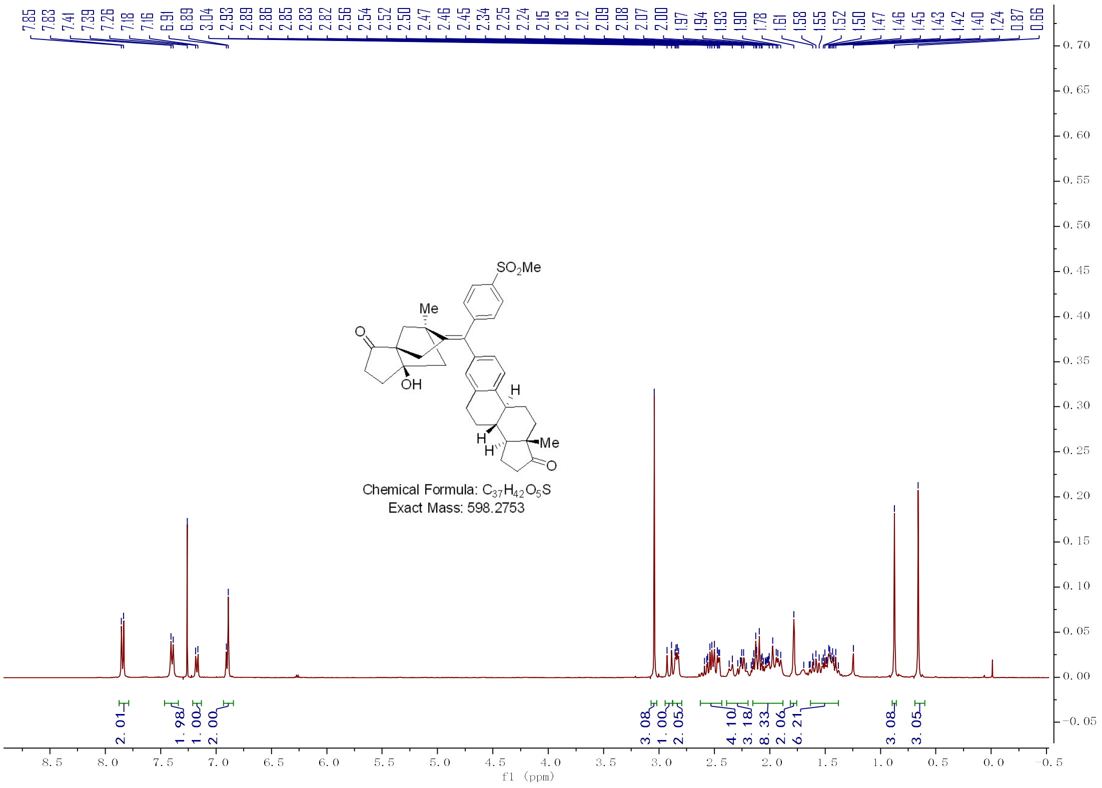

Supplementary Figure 147. ^{1}H NMR spectrum of 3fi

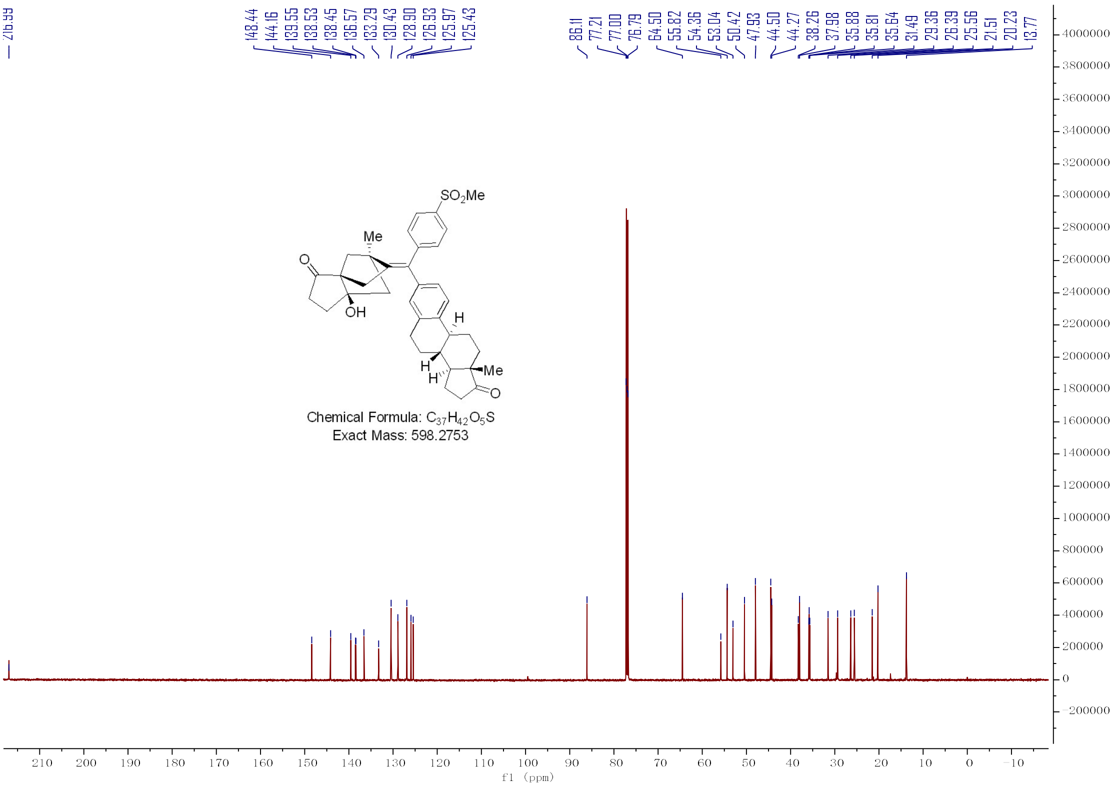

Supplementary Figure 148. ^{13}C NMR spectrum of 3fi

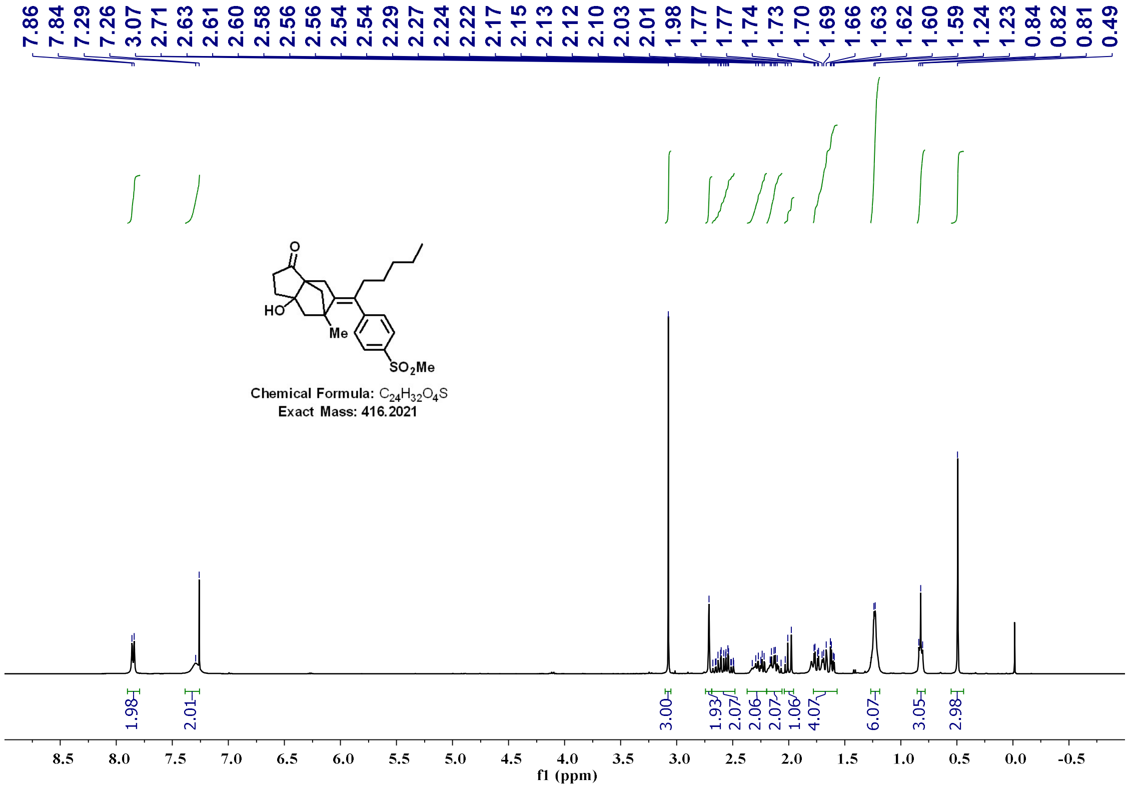

Supplementary Figure 149. ^{1}H NMR spectrum of 3gi

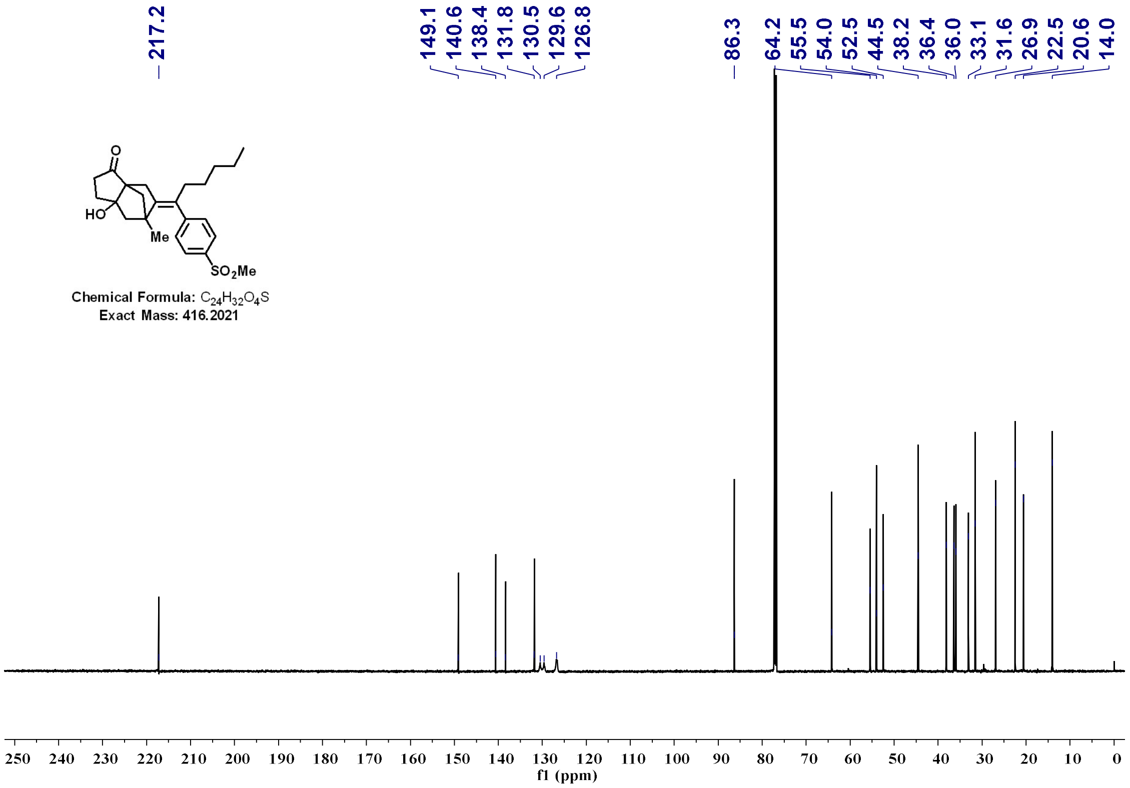

Supplementary Figure 150. ^{13}C NMR spectrum of 3gi

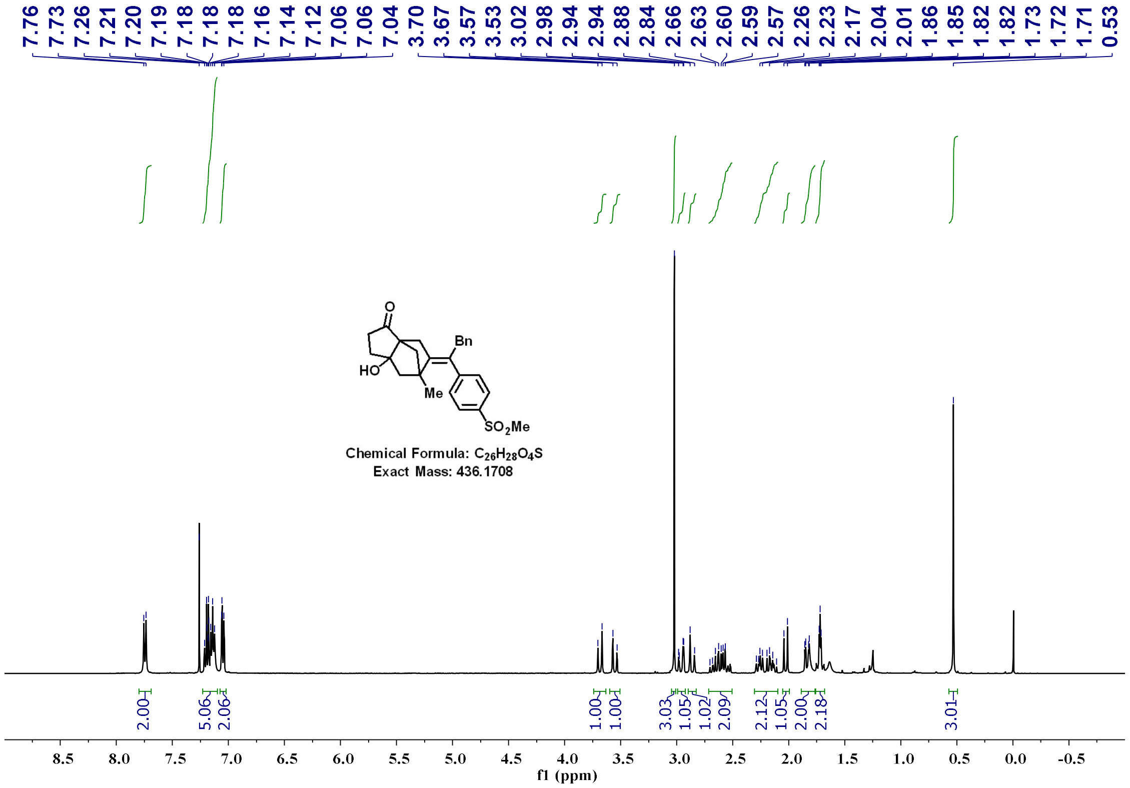

Supplementary Figure 151. ^{1}H NMR spectrum of 3hi

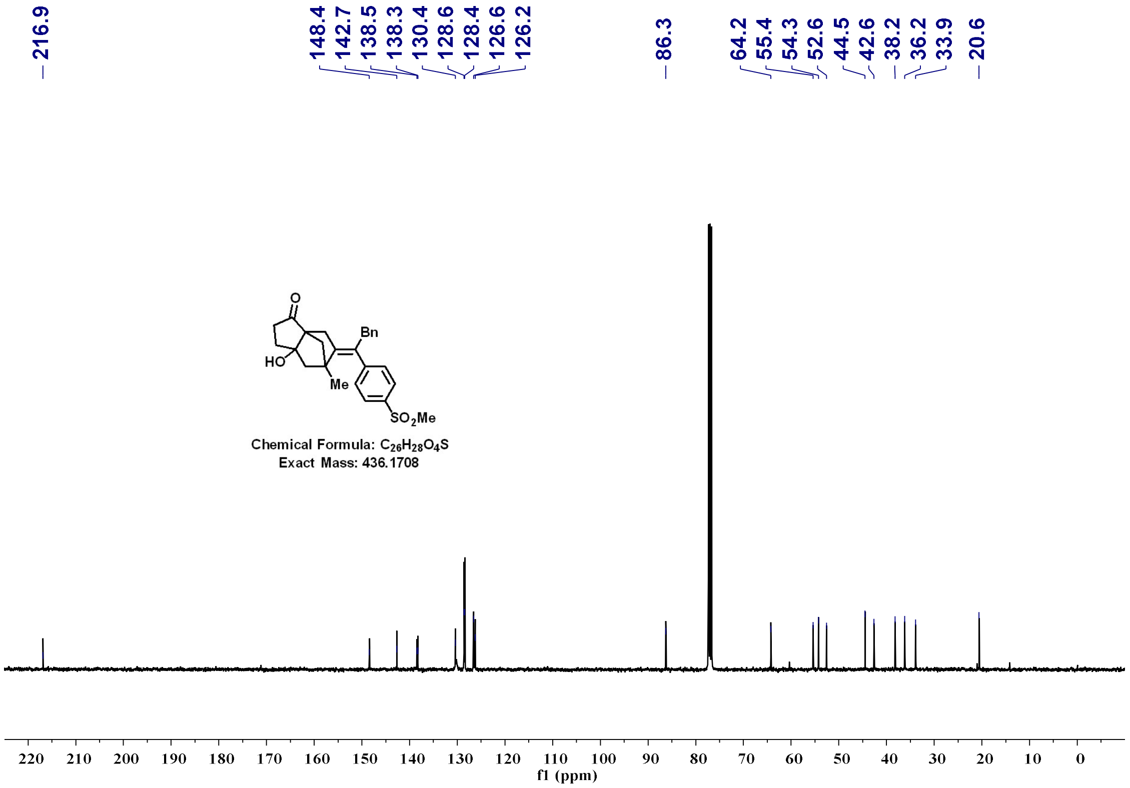

Supplementary Figure 152. ^{13}C NMR spectrum of 3hi

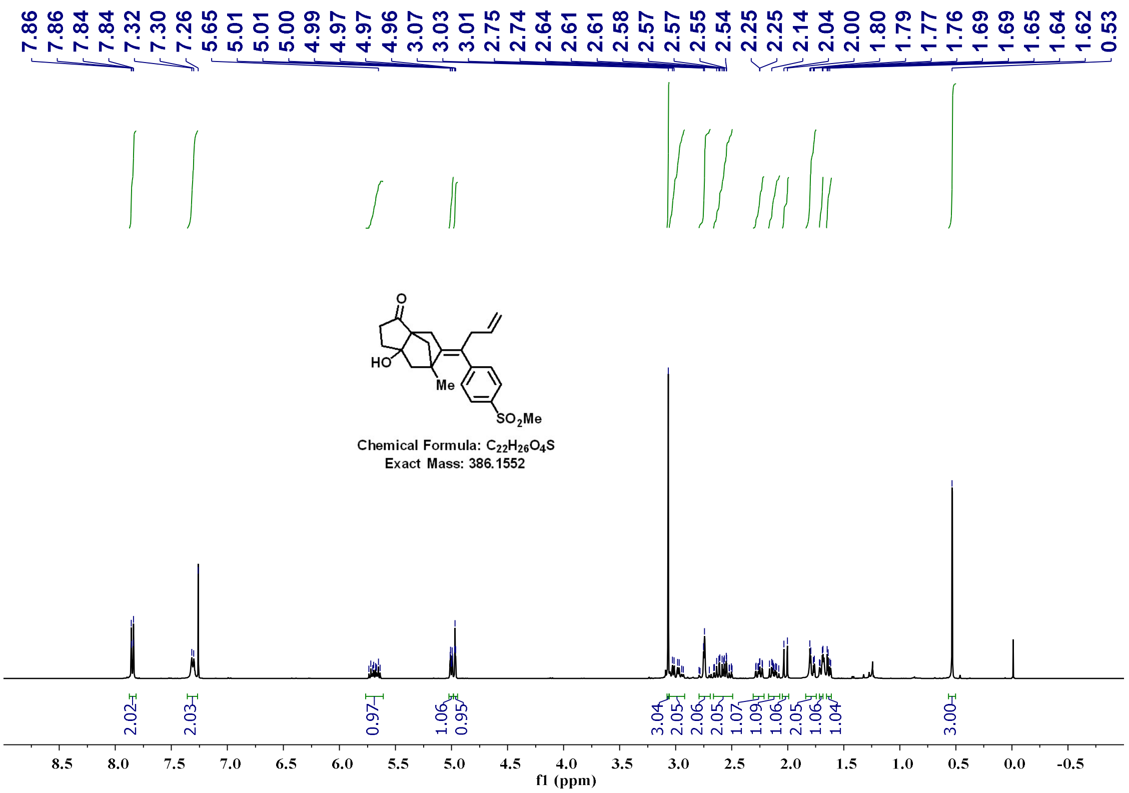

Supplementary Figure 153. ^{1}**H NMR spectrum of 3ii**

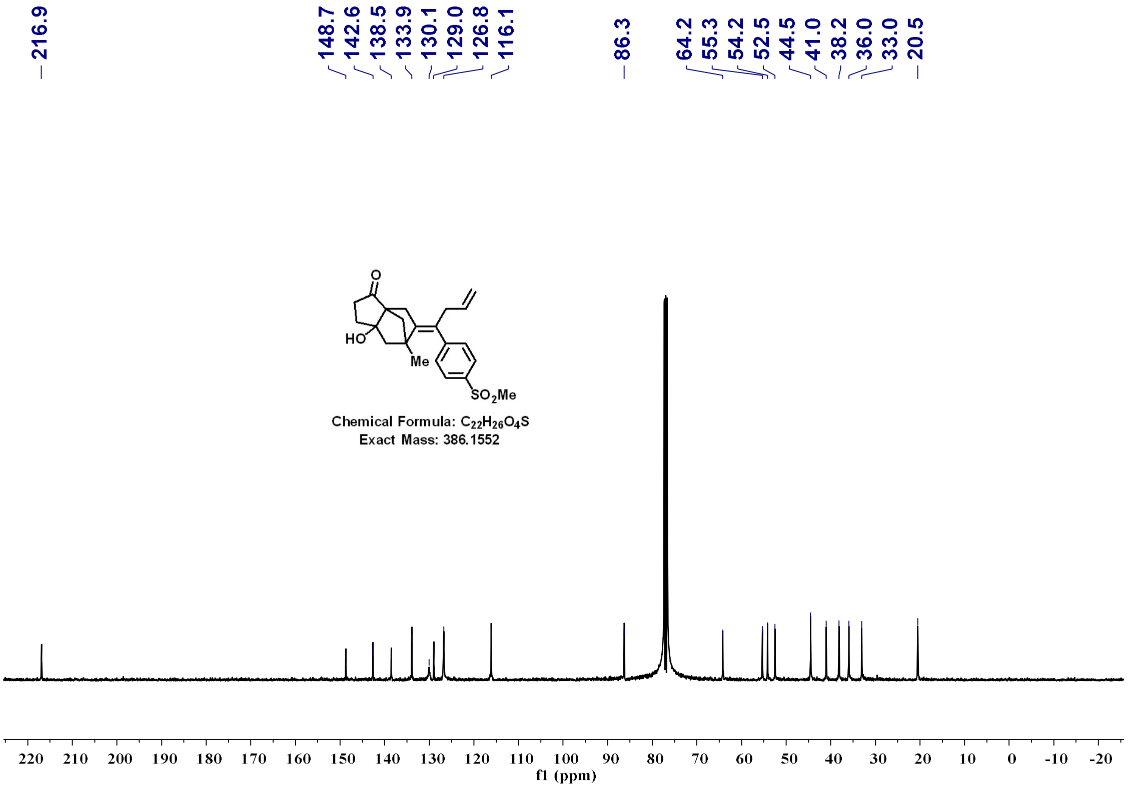

Supplementary Figure 154. ^{13}**C NMR spectrum of 3ii**

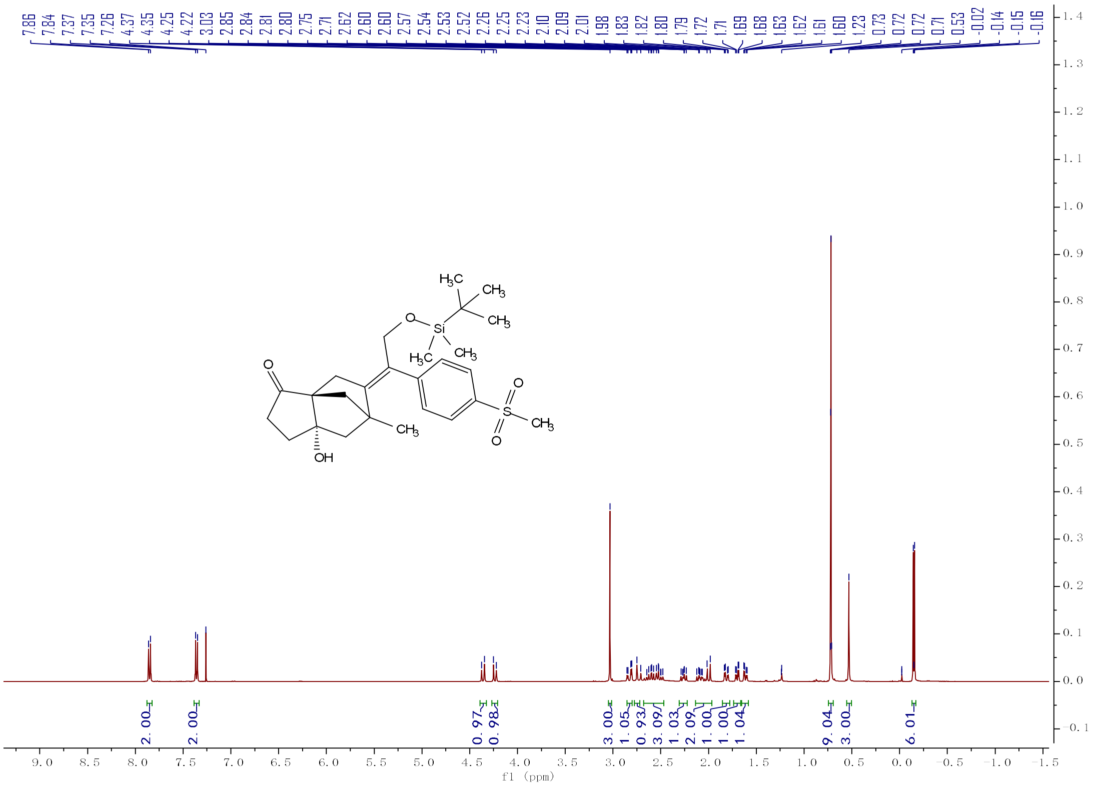

Supplementary Figure 155. ^{1}H NMR spectrum of 3ji

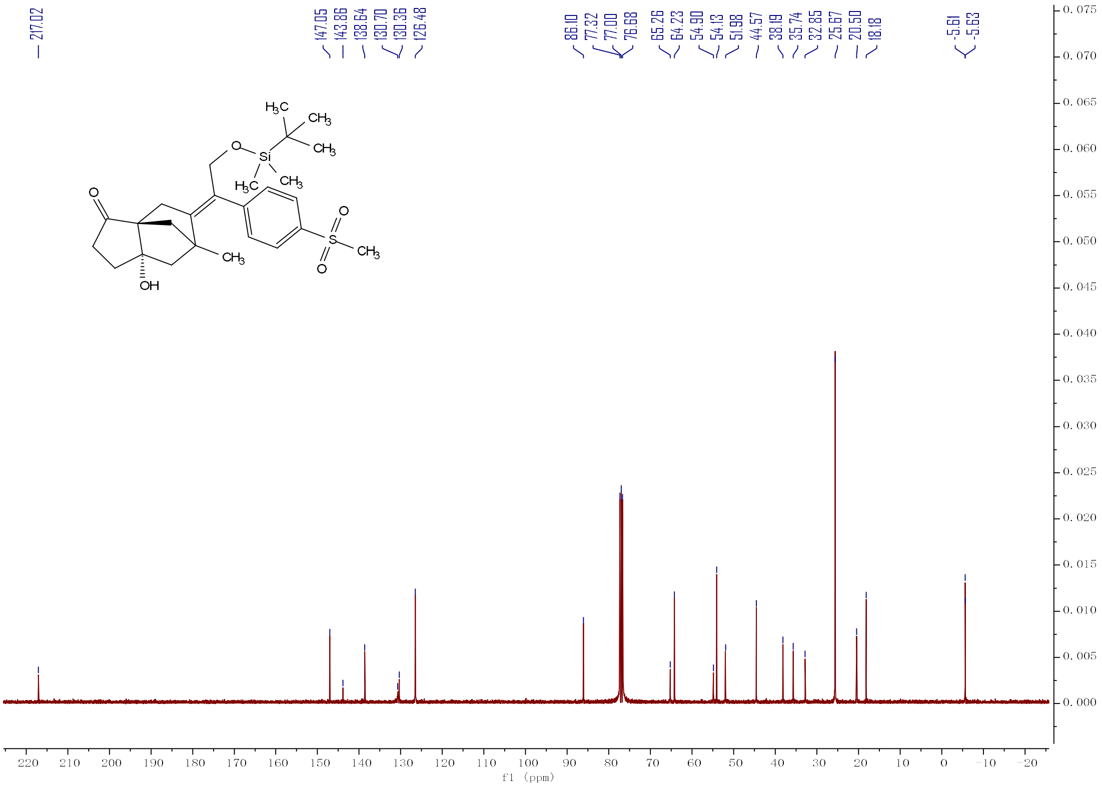

Supplementary Figure 156. ^{13}C NMR spectrum of 3ji

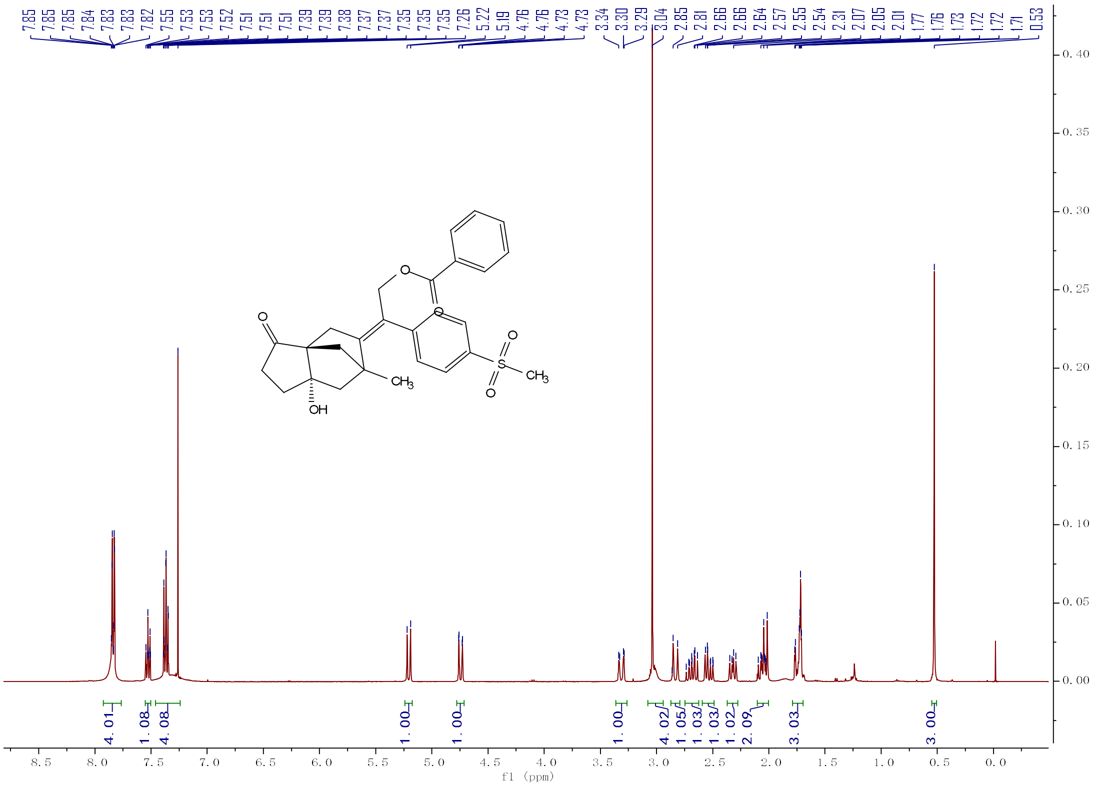

Supplementary Figure 157. ^{1}H NMR spectrum of 3ki

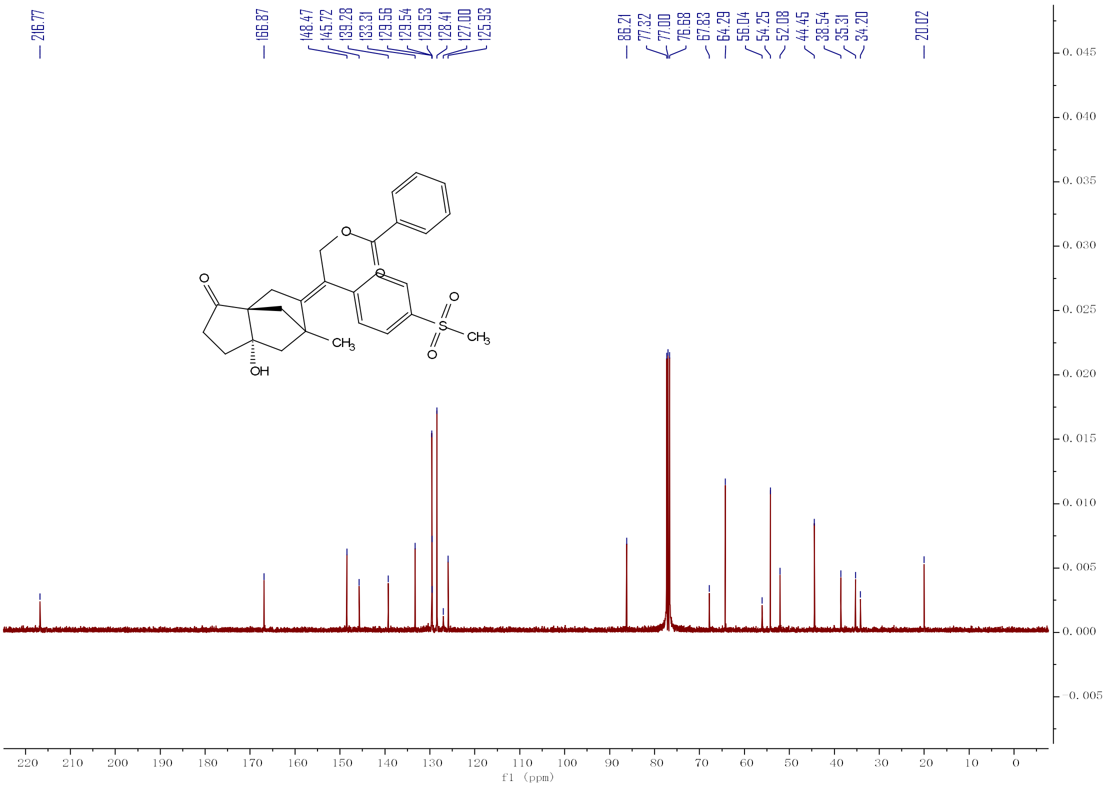

Supplementary Figure 158. ^{13}C NMR spectrum of 3ki

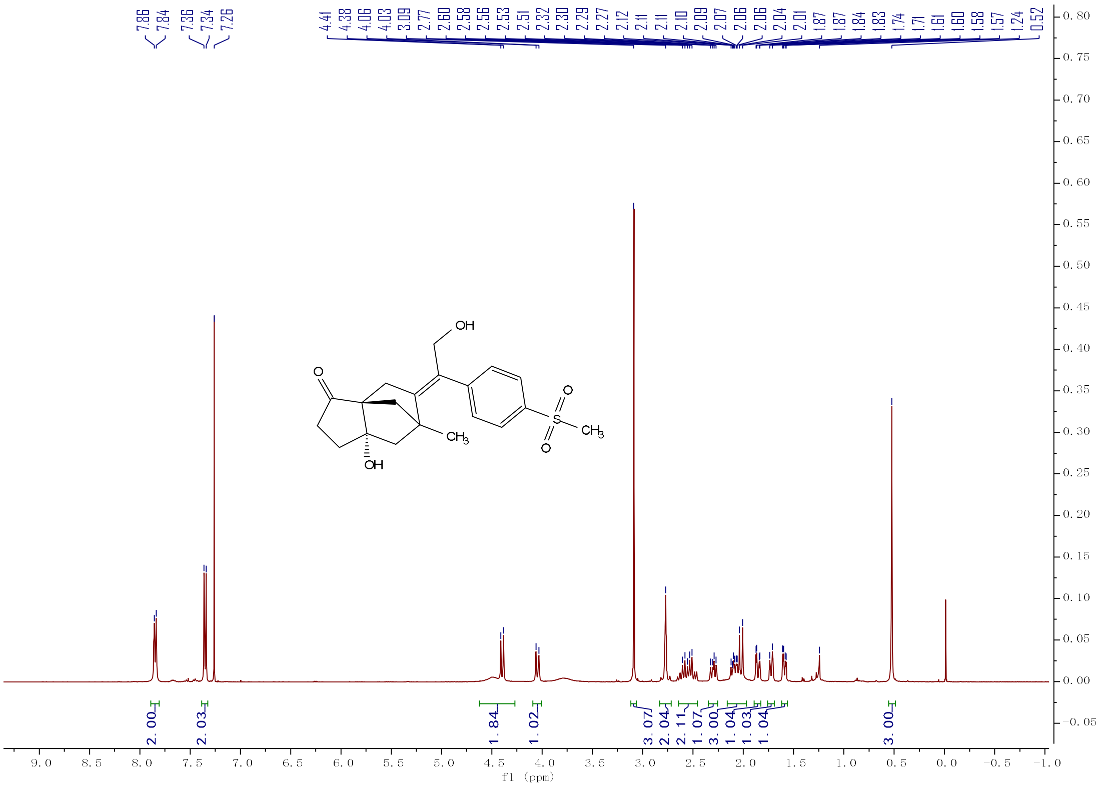

Supplementary Figure 159. ^{1}H NMR spectrum of 3li

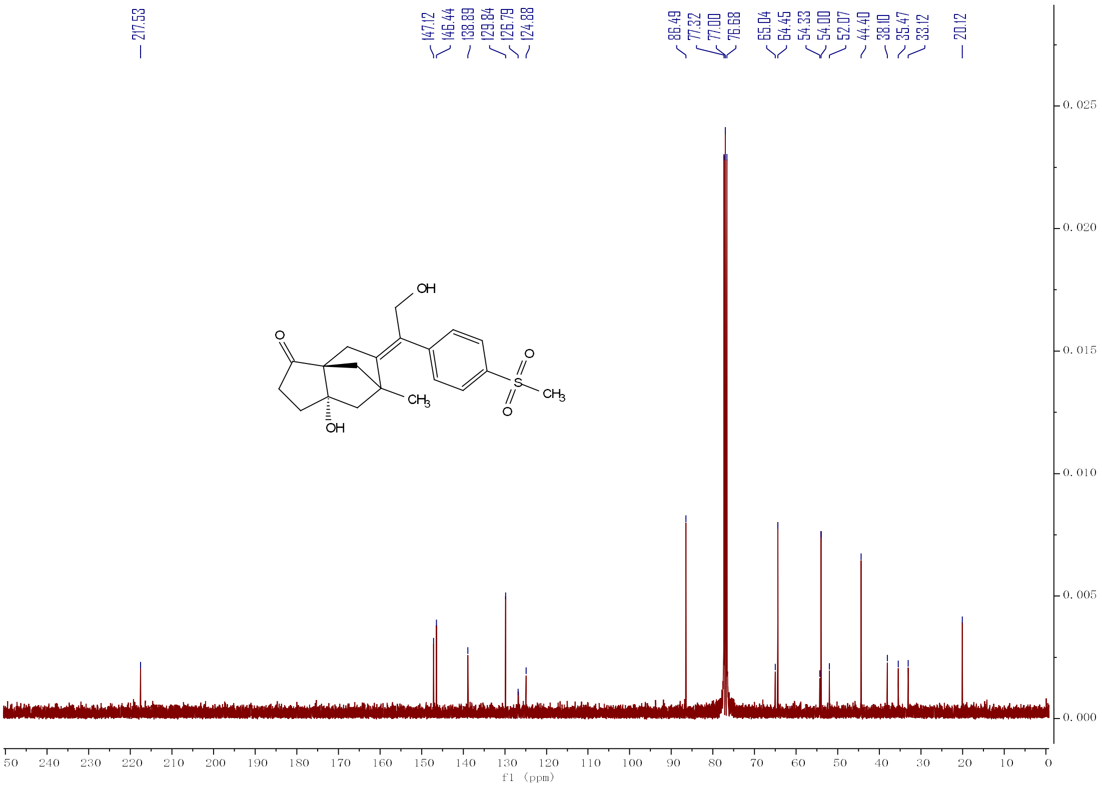

Supplementary Figure 160. ^{13}C NMR spectrum of 3li

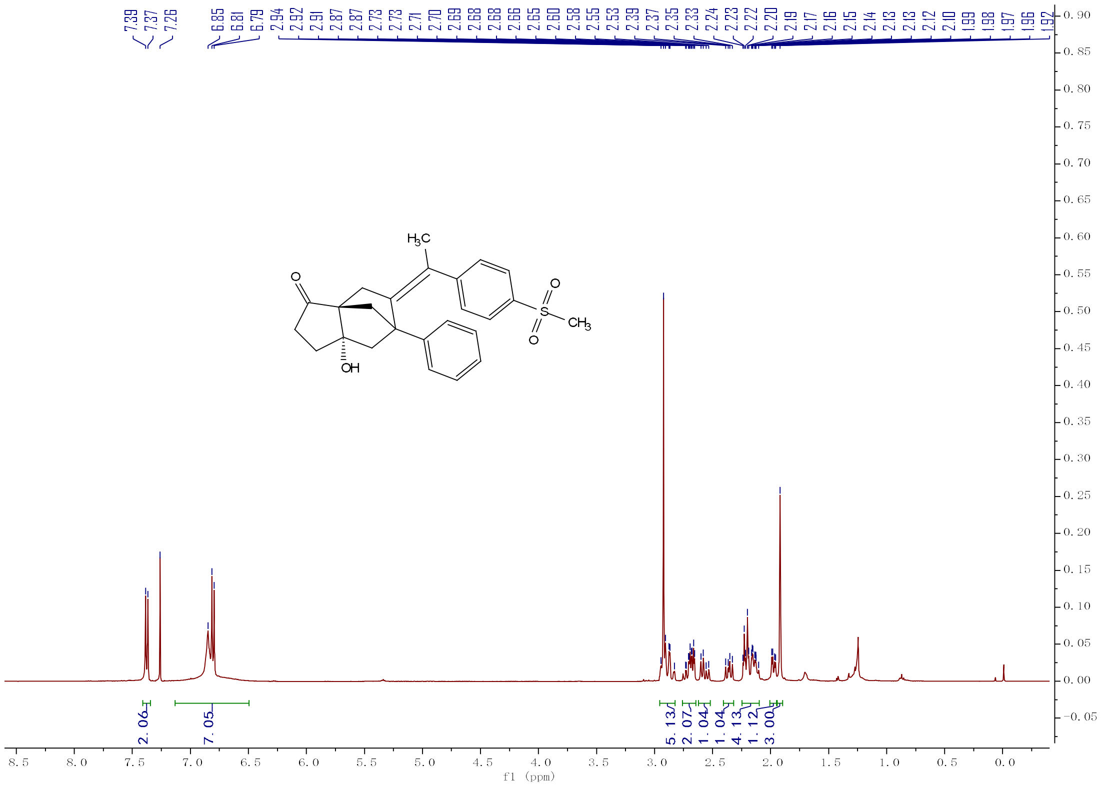

Supplementary Figure 161. ^{1}H NMR spectrum of 3mi

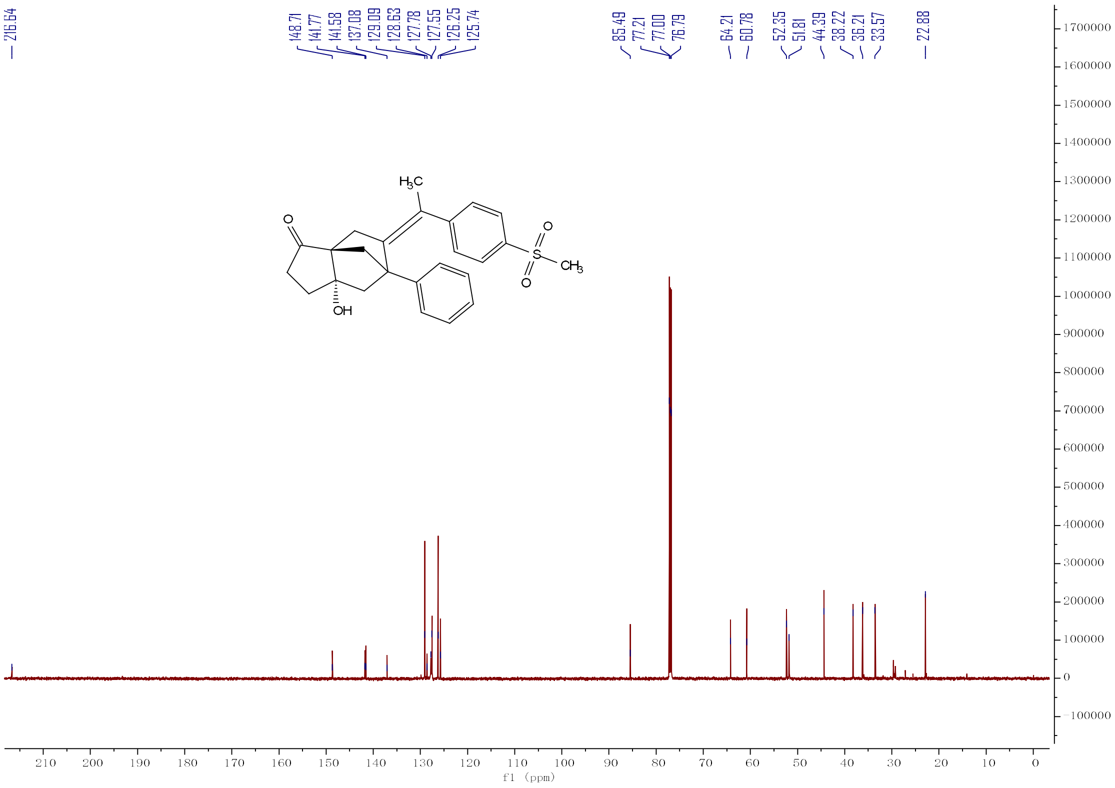

Supplementary Figure 162. ^{13}C NMR spectrum of 3mi

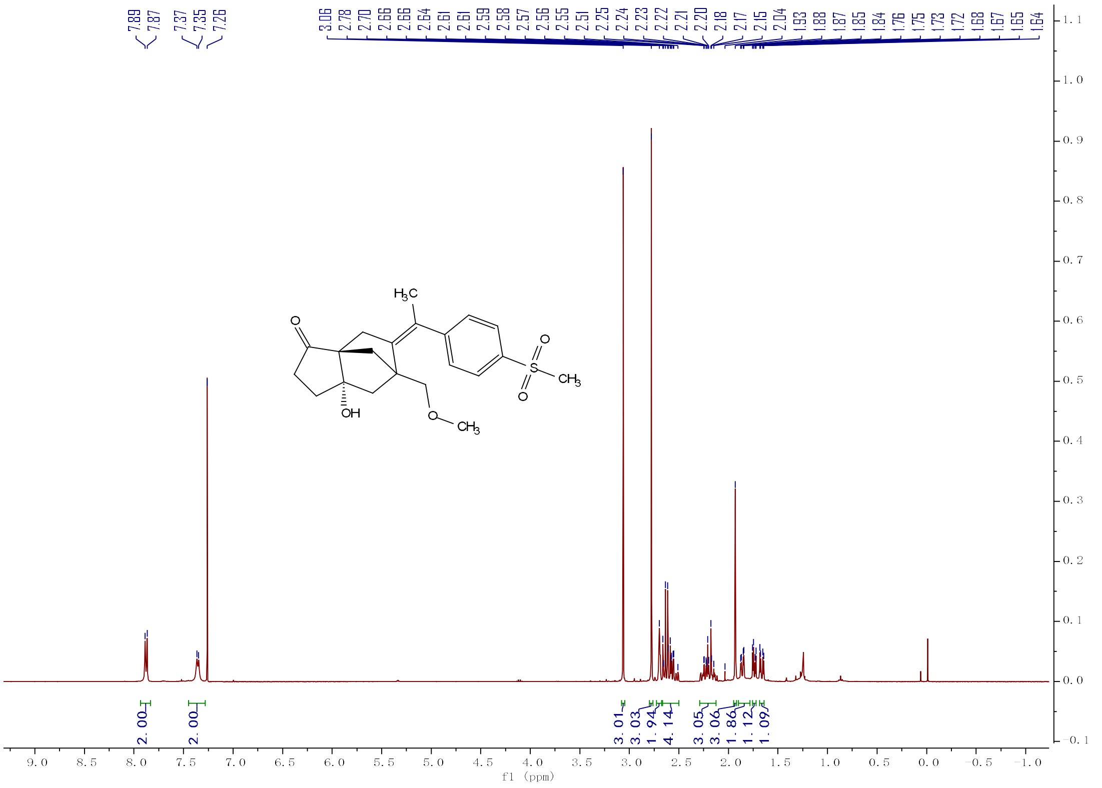

Supplementary Figure 163. ^{1}H NMR spectrum of 3ni

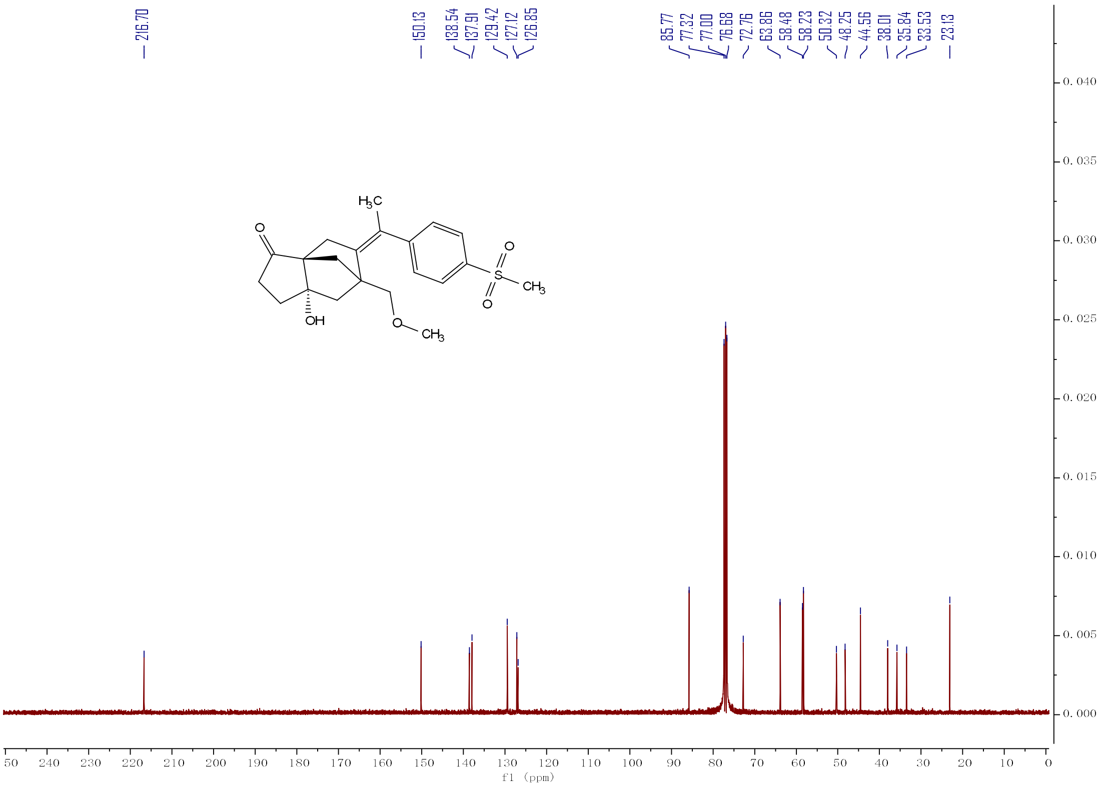

Supplementary Figure 164. ^{13}C NMR spectrum of 3ni

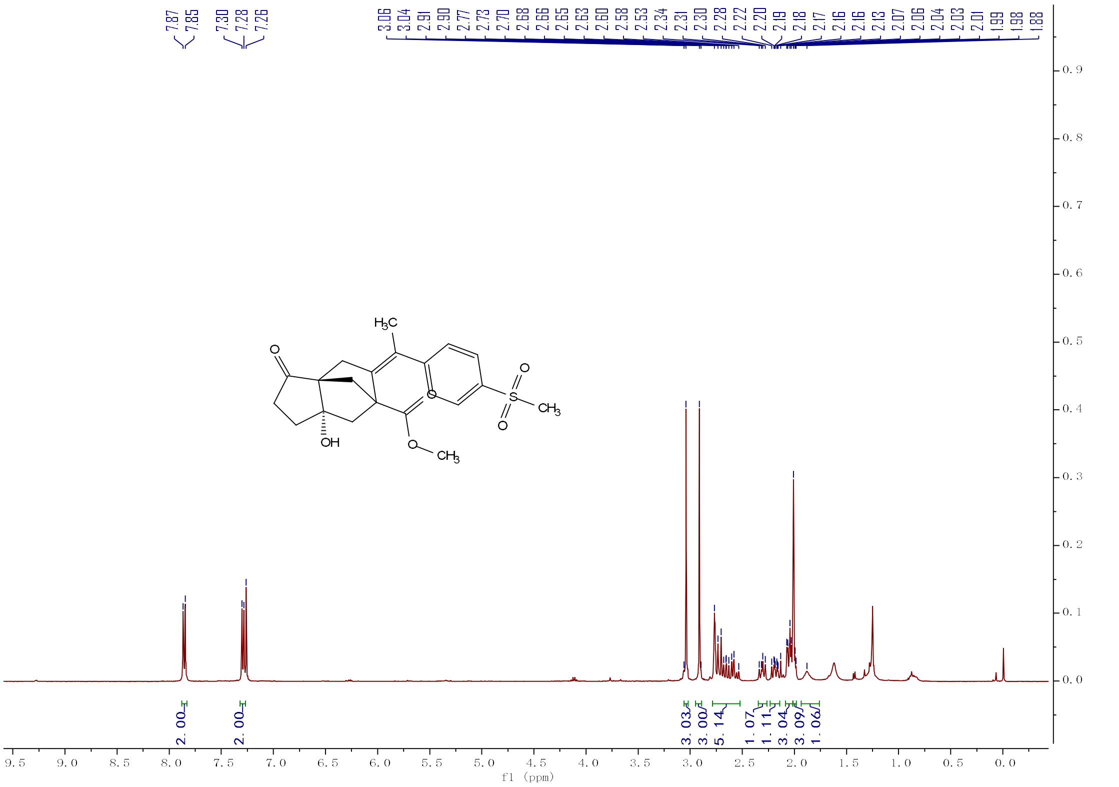

Supplementary Figure 165. ^{1}H NMR spectrum of 3oi

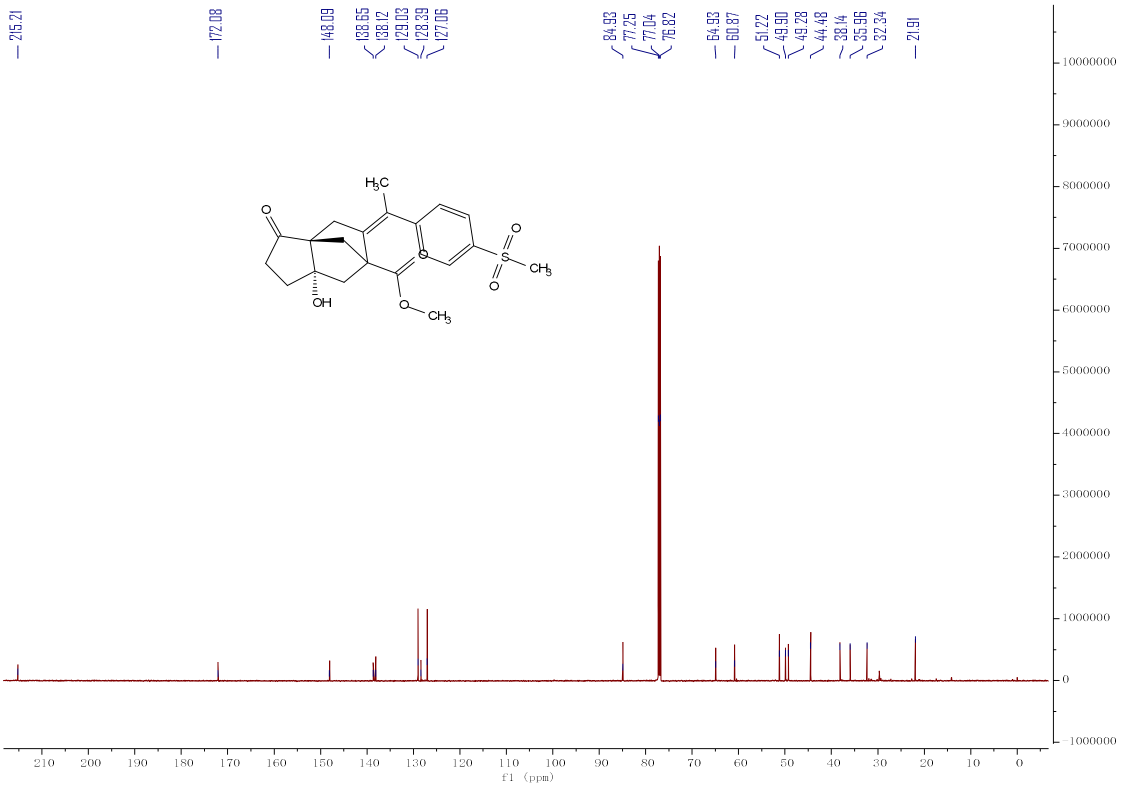

Supplementary Figure 166. ^{13}C NMR spectrum of 3oi

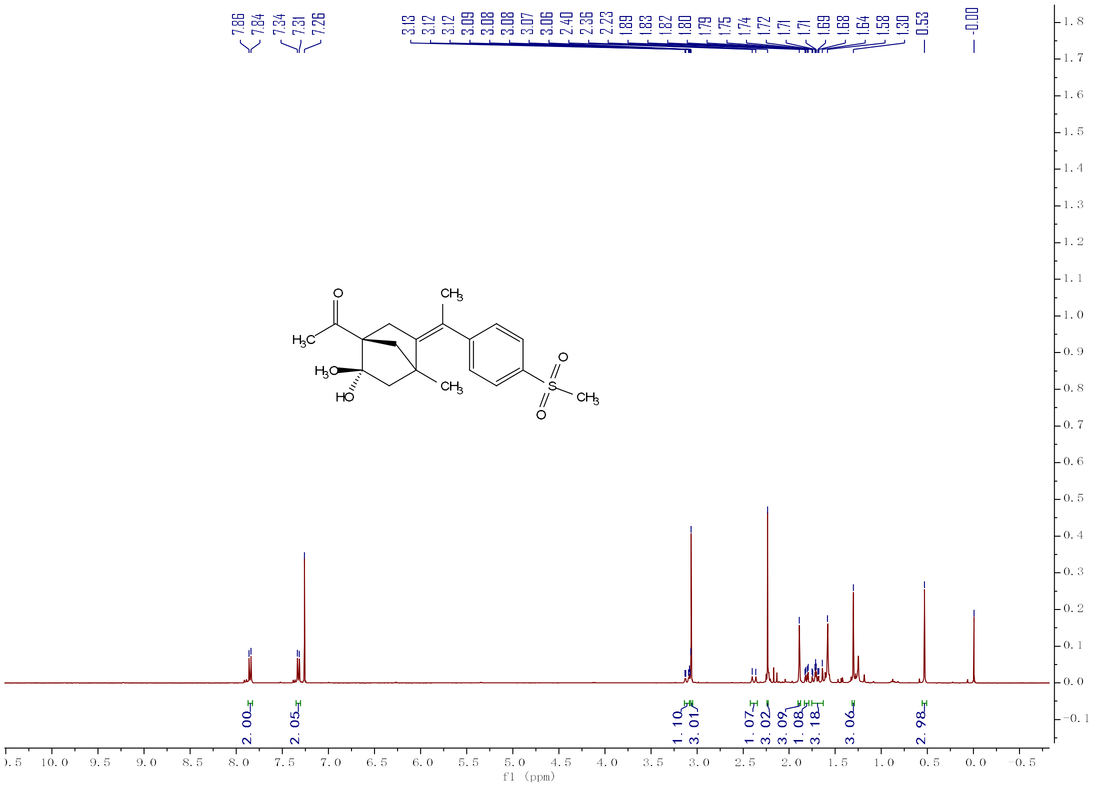

Supplementary Figure 167. ^{1}H NMR spectrum of 3pi

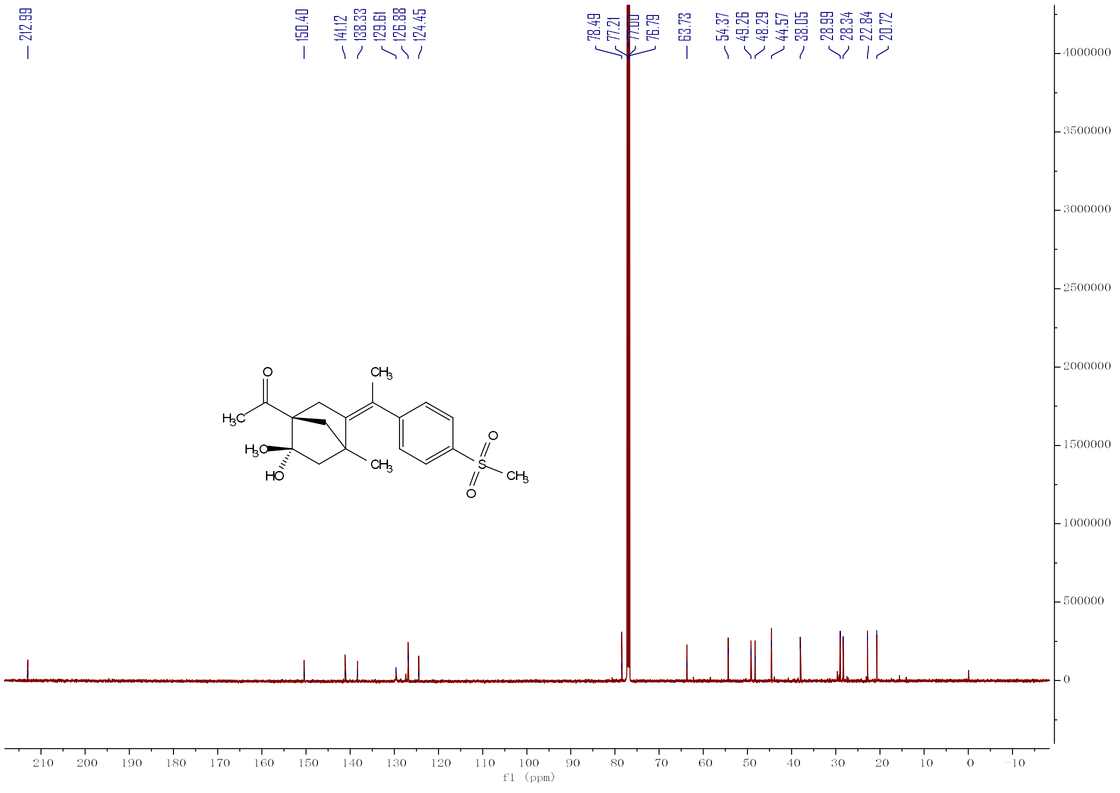

Supplementary Figure 168. ^{13}C NMR spectrum of 3pi

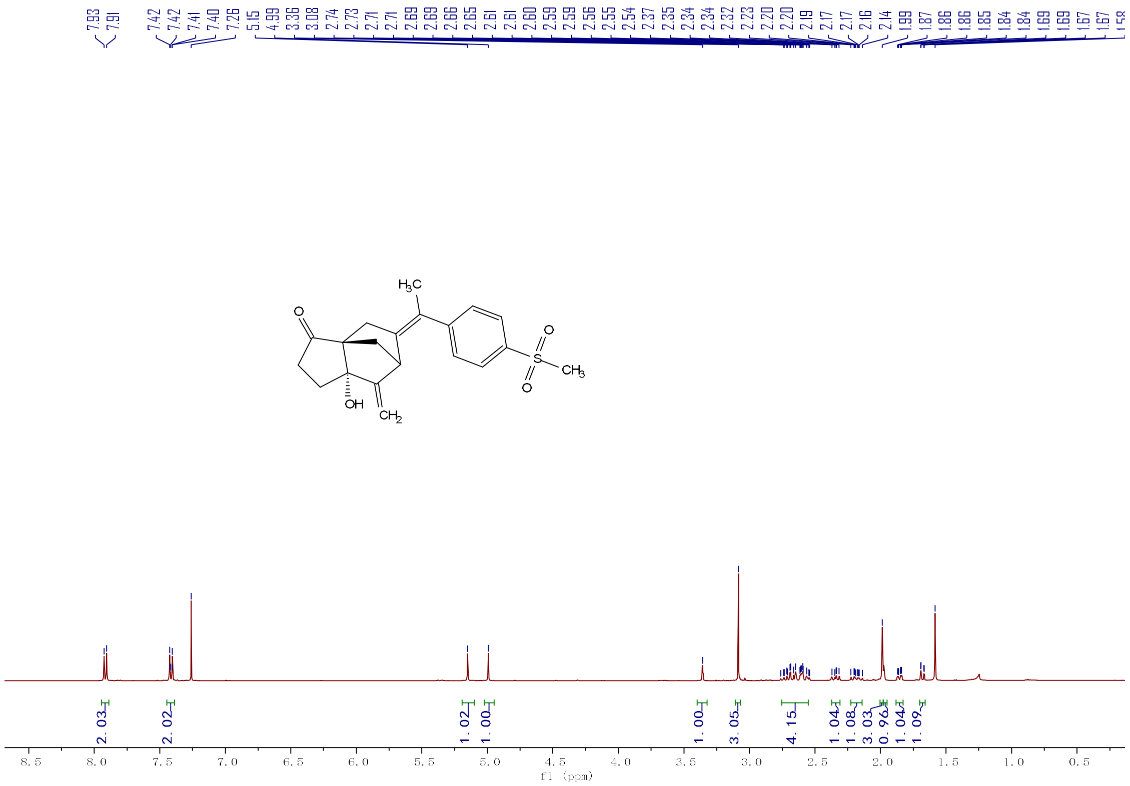

Supplementary Figure 169. ^{1}H NMR spectrum of 3qi

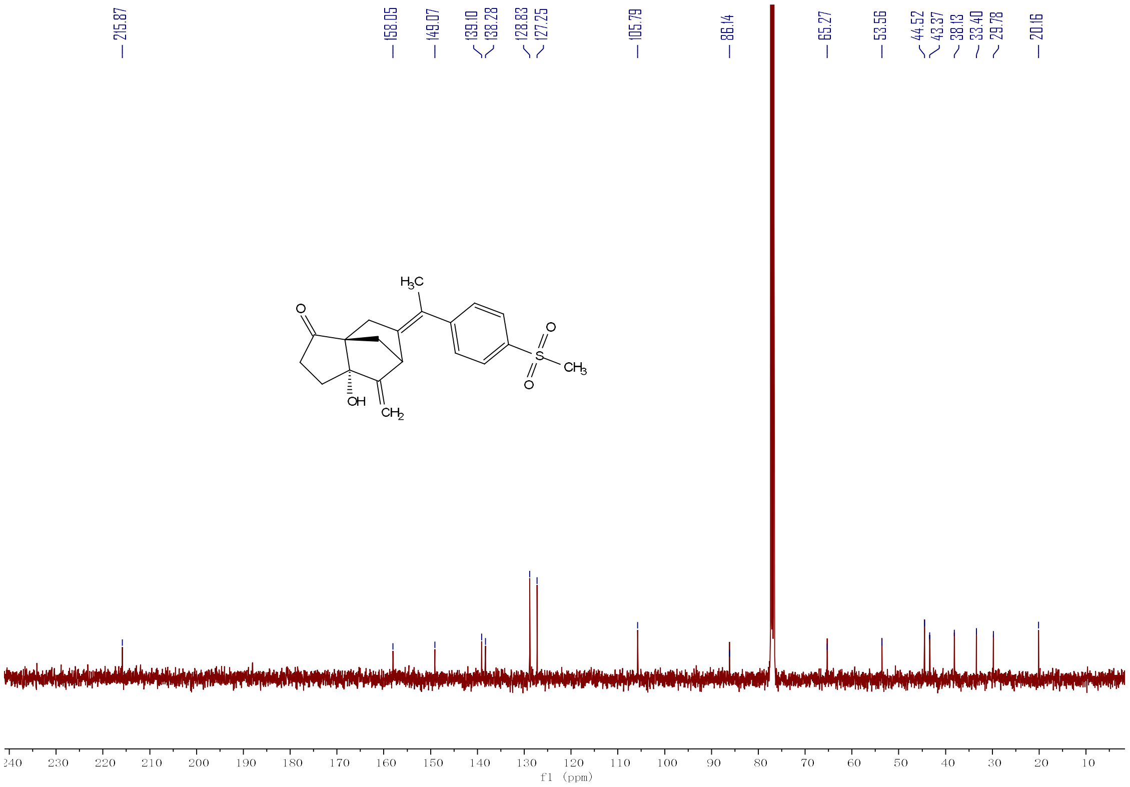

Supplementary Figure 170. ^{13}C NMR spectrum of 3qi

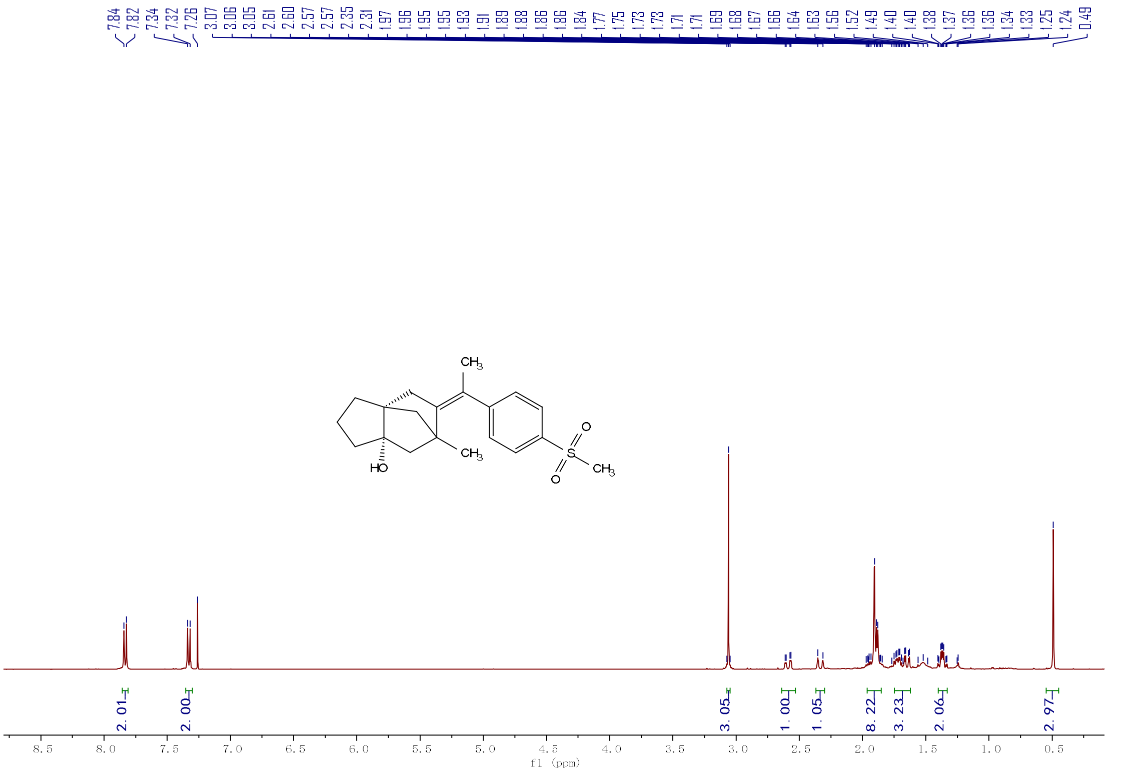

Supplementary Figure 171. ^{1}H NMR spectrum of 3ri

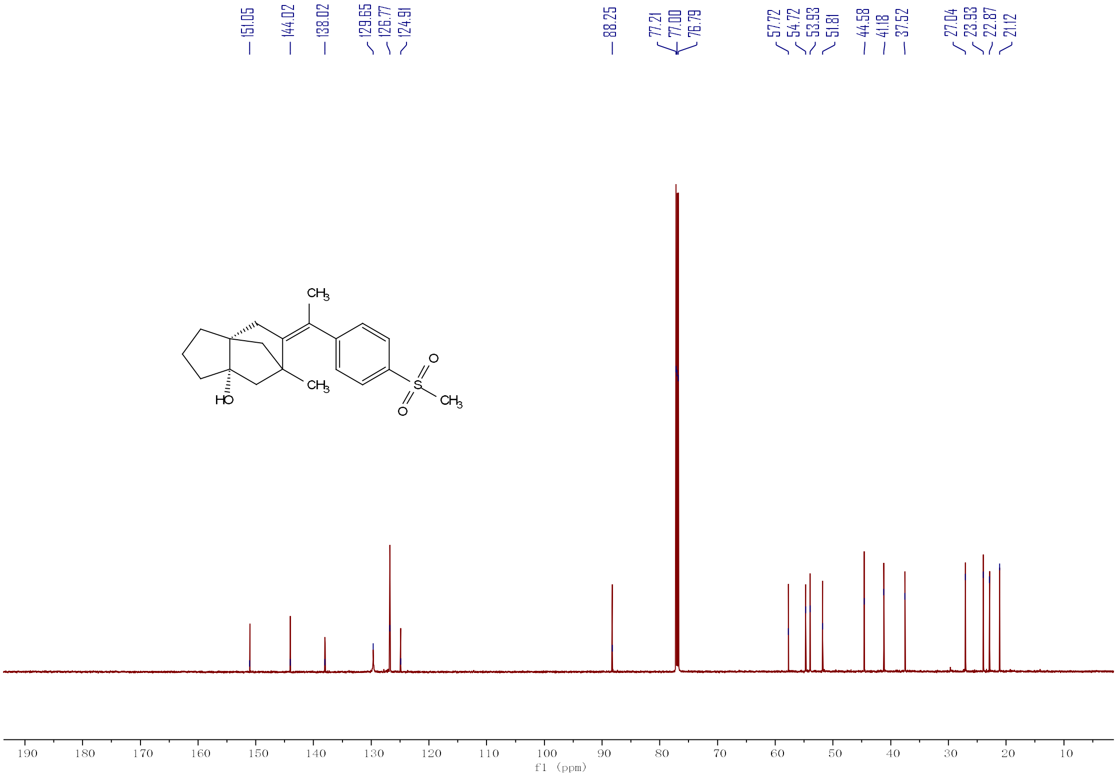

Supplementary Figure 172. ^{13}C NMR spectrum of 3ri

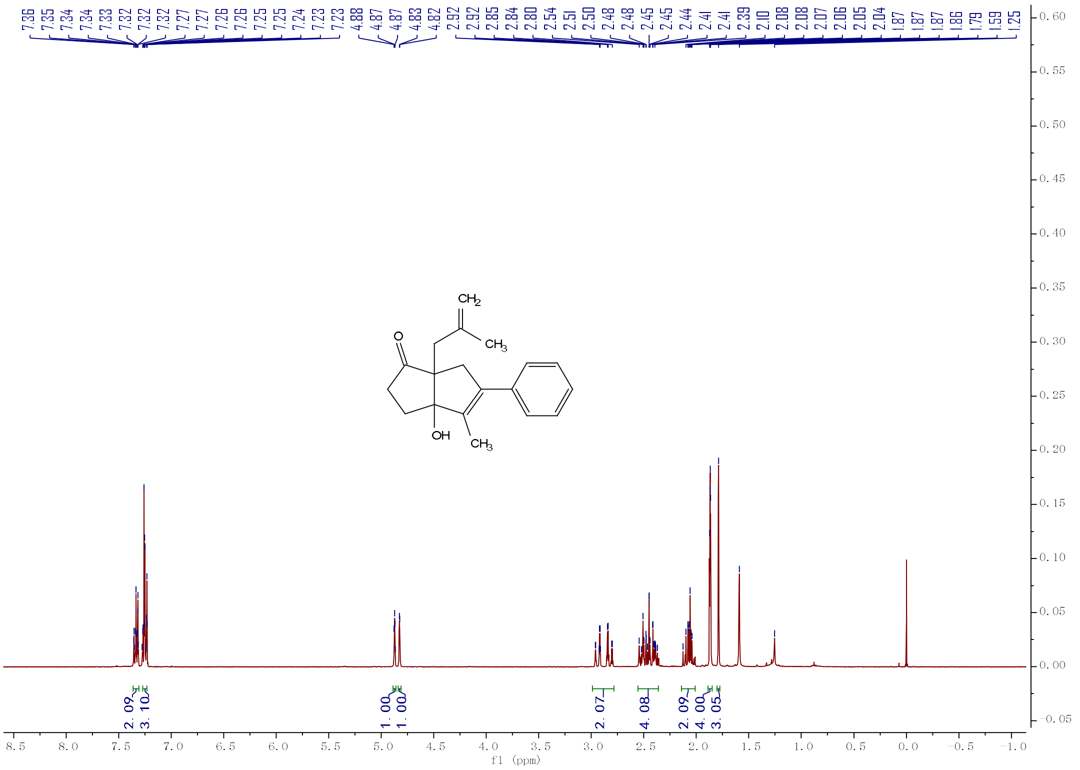

Supplementary Figure 173. ^{1}H NMR spectrum of 4aa

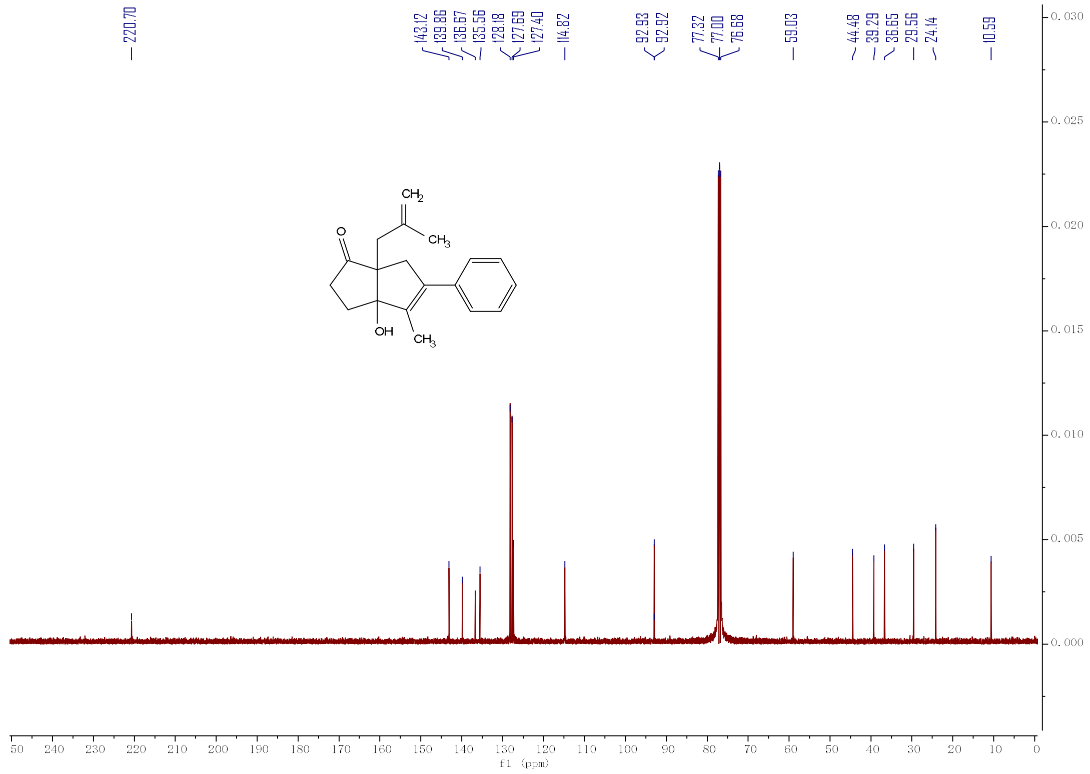

Supplementary Figure 174. ^{13}C NMR spectrum of 4aa

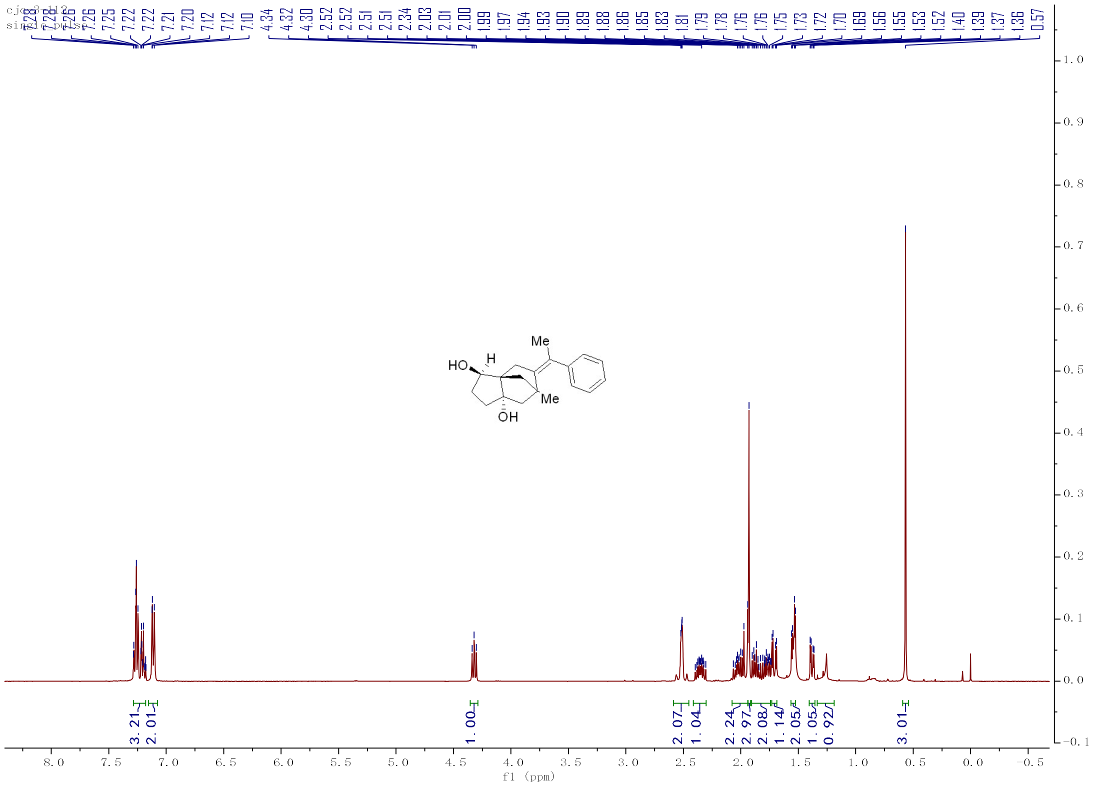

Supplementary Figure 175. ^{1}H NMR spectrum of product 5

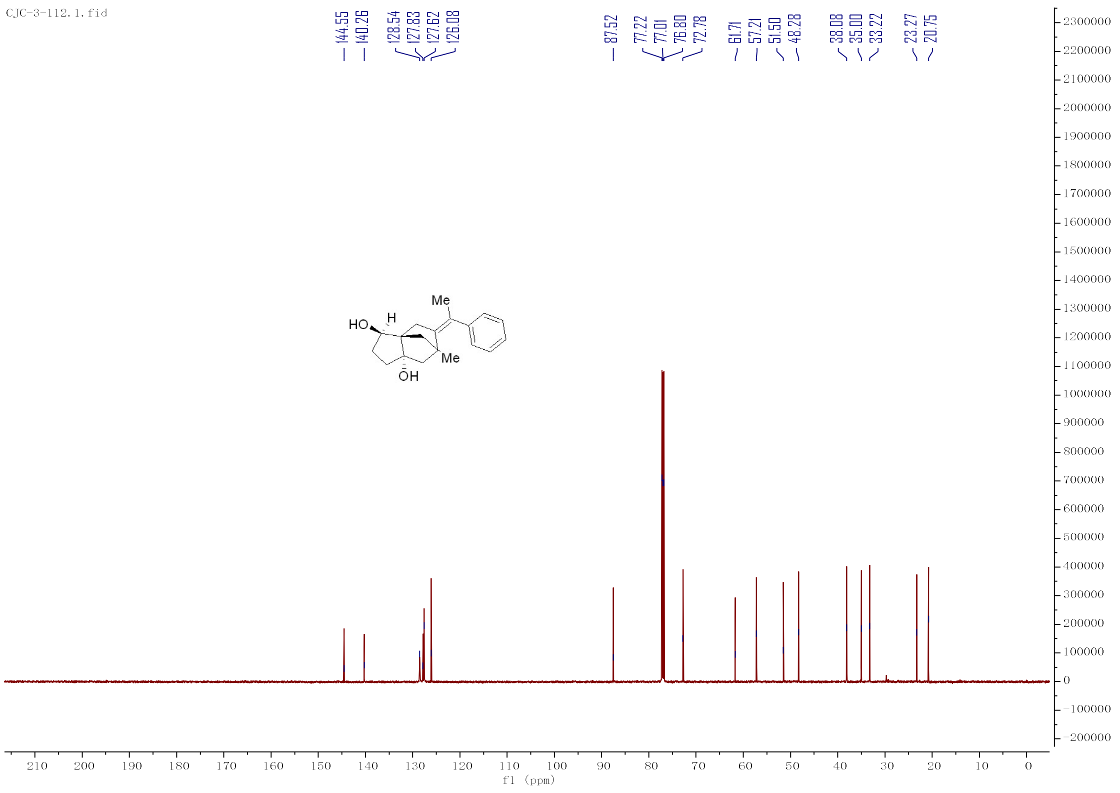

Supplementary Figure 176. ^{13}C NMR spectrum of product 5

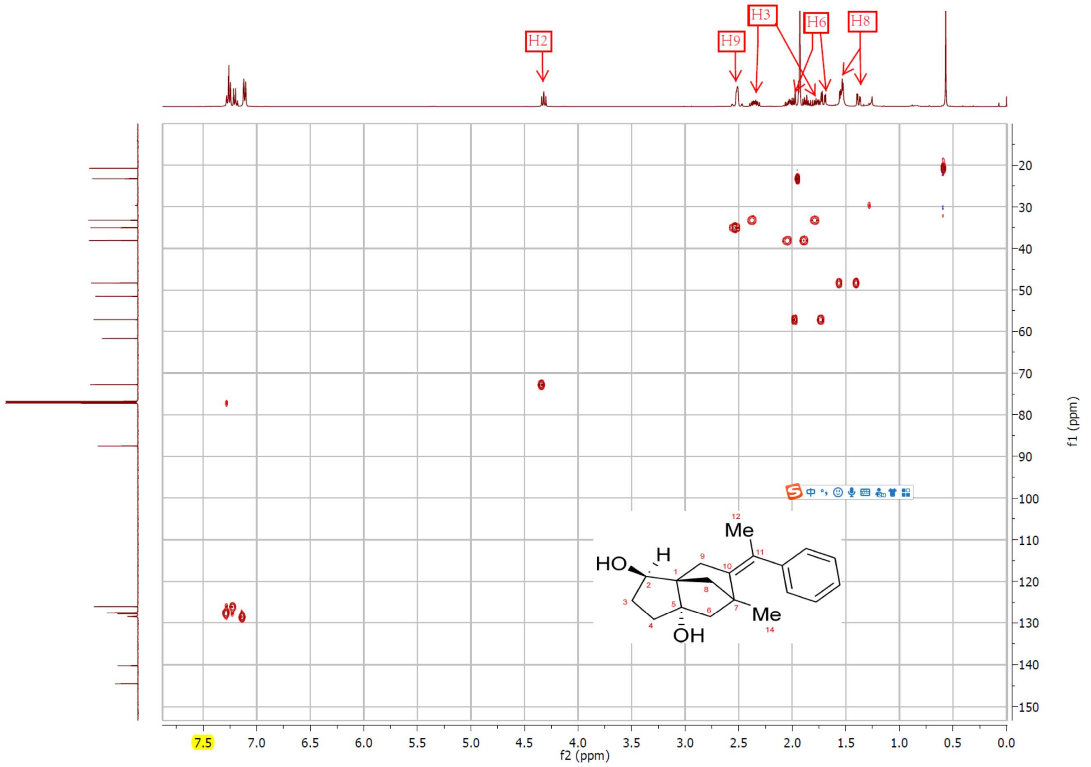

Supplementary Figure 177. HSQC spectrum of product 5

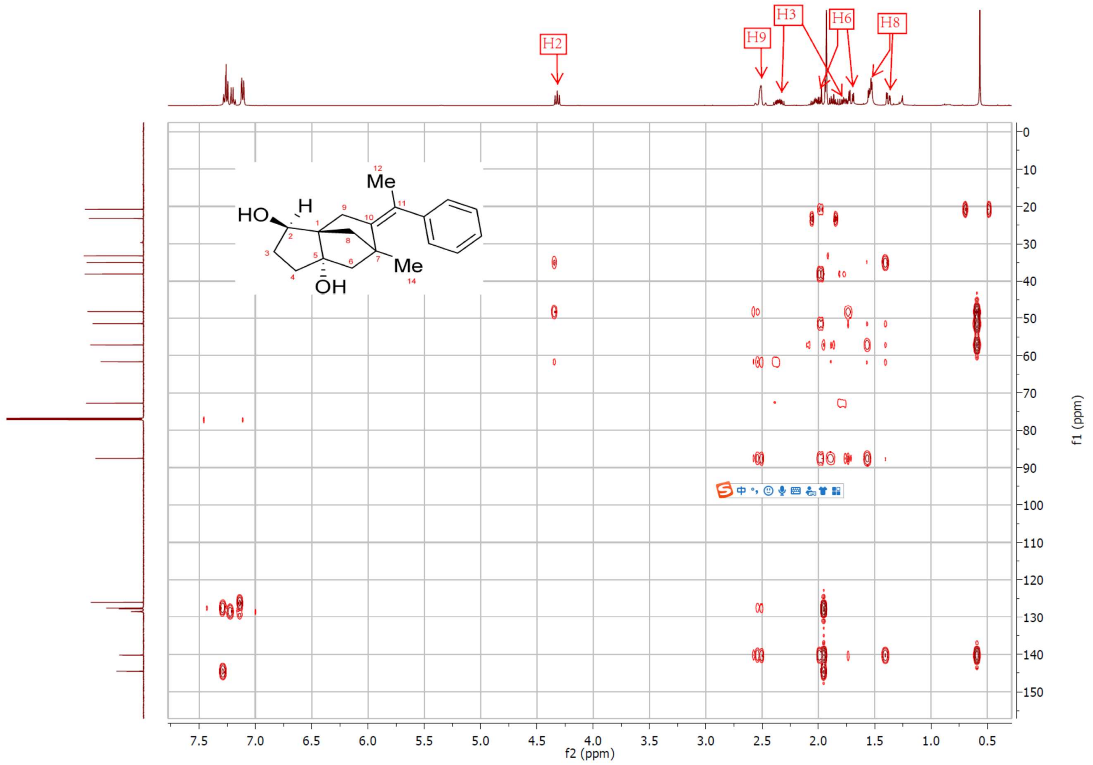

Supplementary Figure 178. HMBC spectrum of product 5

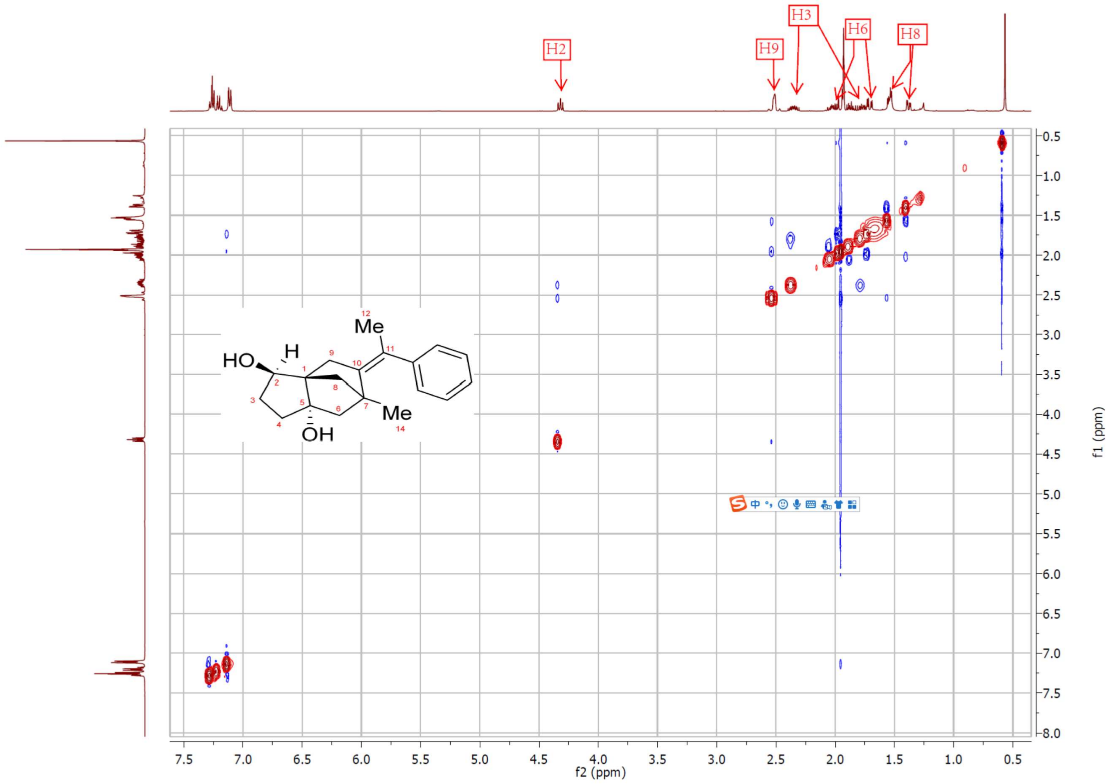

Supplementary Figure 179. NOE spectrum of product 5

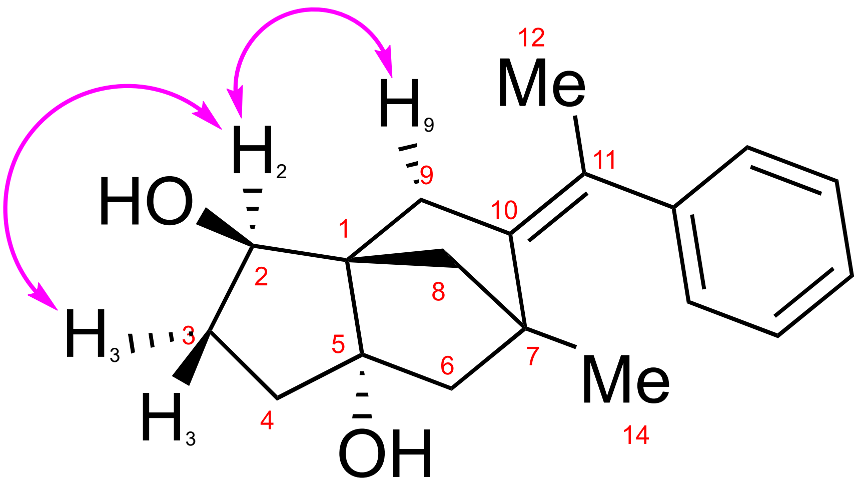

Supplementary Figure 180. Structure of product 5

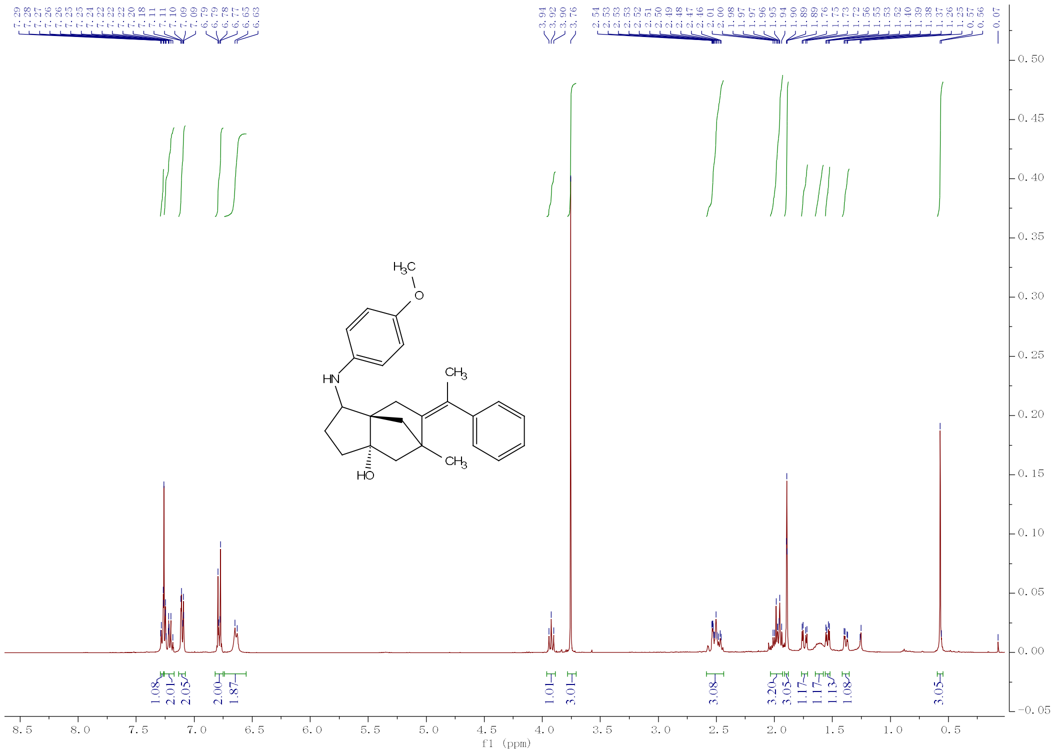

Supplementary Figure 181. ^{1}H NMR spectrum of product 6

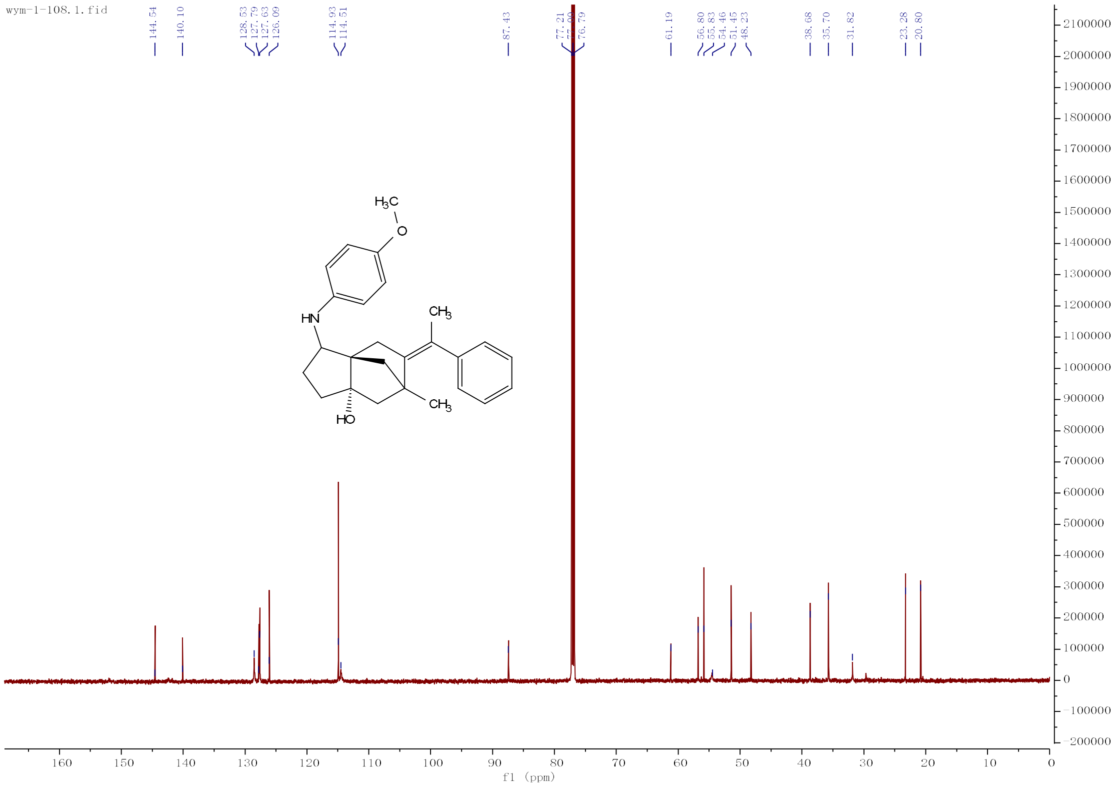

Supplementary Figure 182. ^{13}C NMR spectrum of product 6

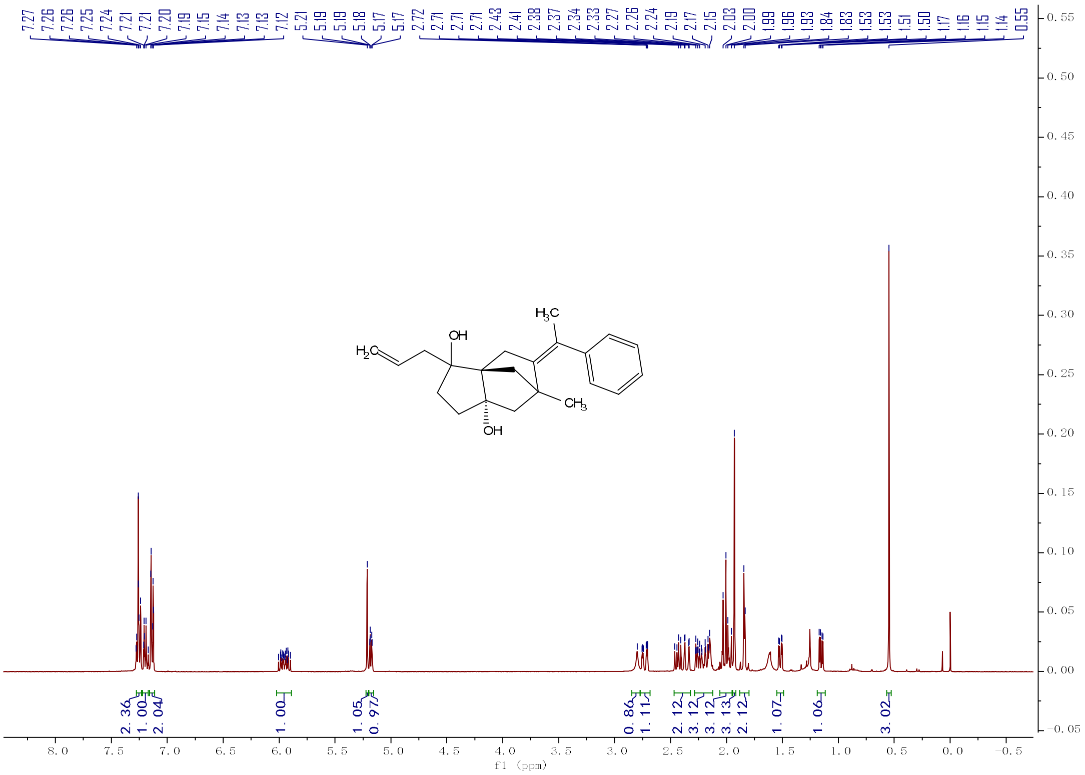

Supplementary Figure 183. ^{1}H NMR spectrum of product 7

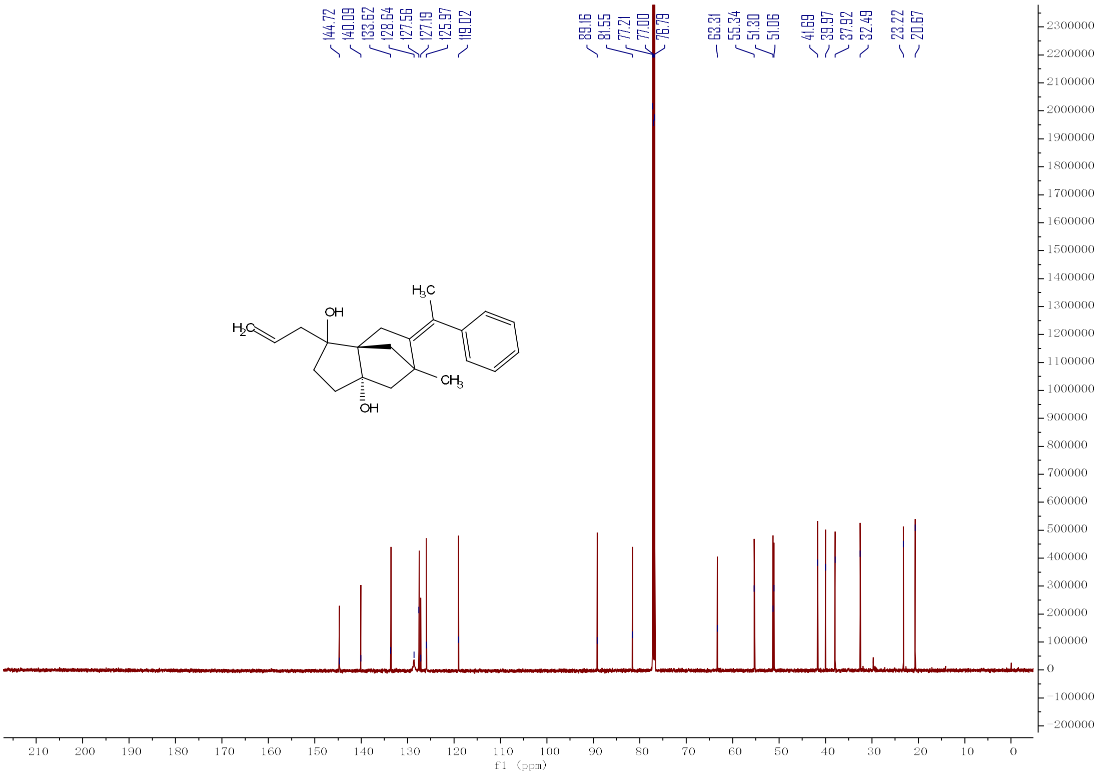

Supplementary Figure 184. ^{13}C NMR spectrum of product 7

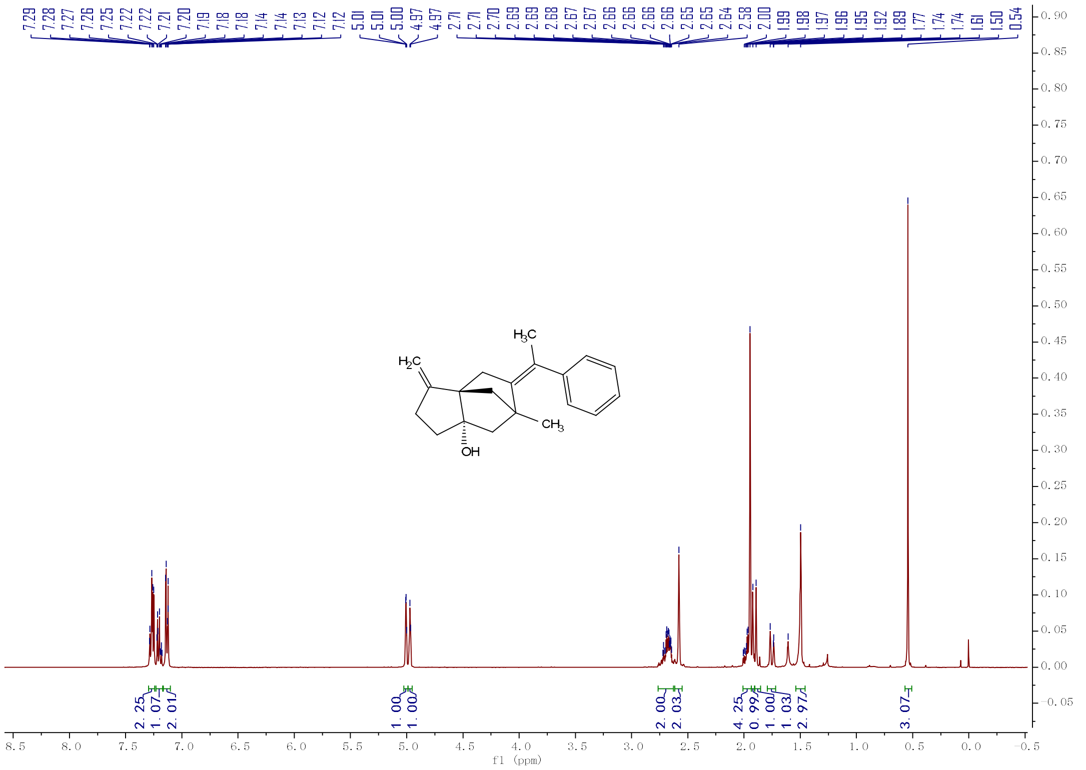

Supplementary Figure 185. ^{1}H NMR spectrum of product 8

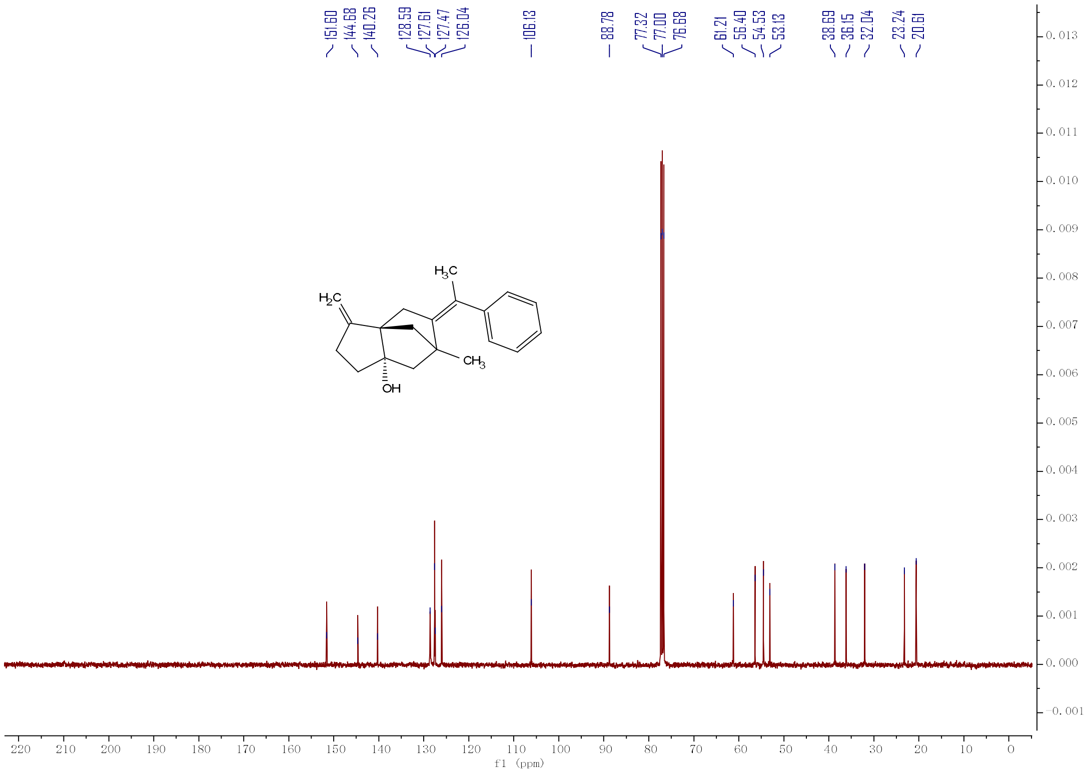

Supplementary Figure 186. ^{13}C NMR spectrum of product 8

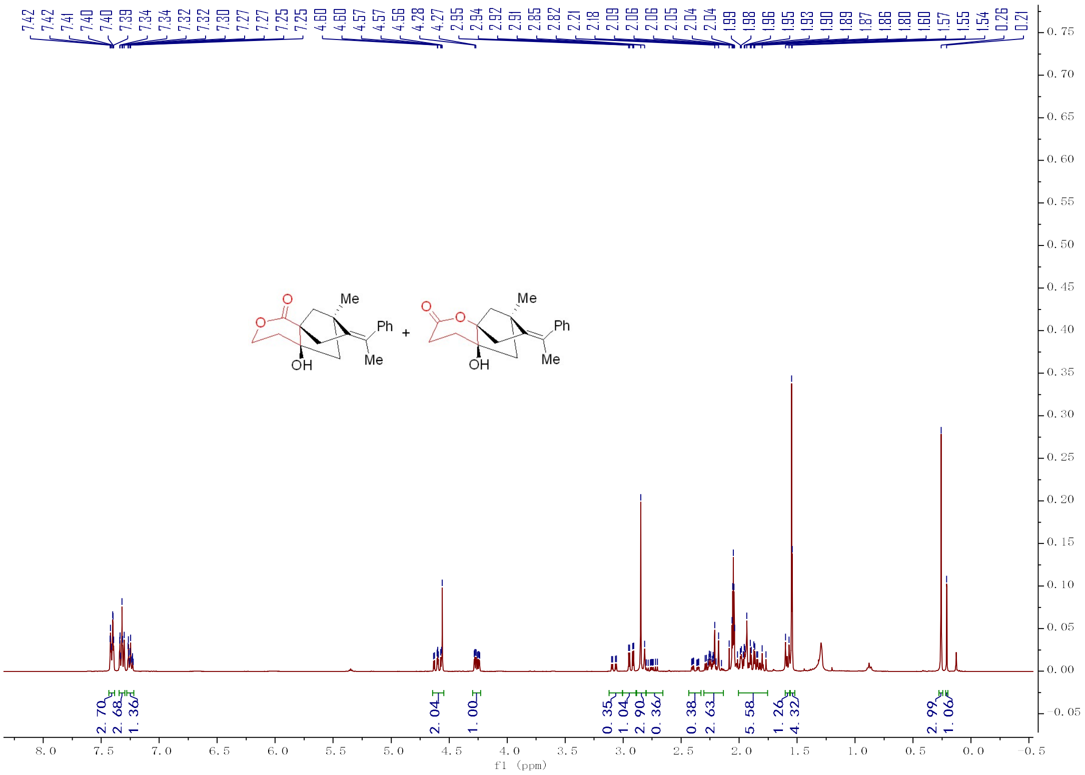

Supplementary Figure 187. ^{1}H NMR spectrum of product 9

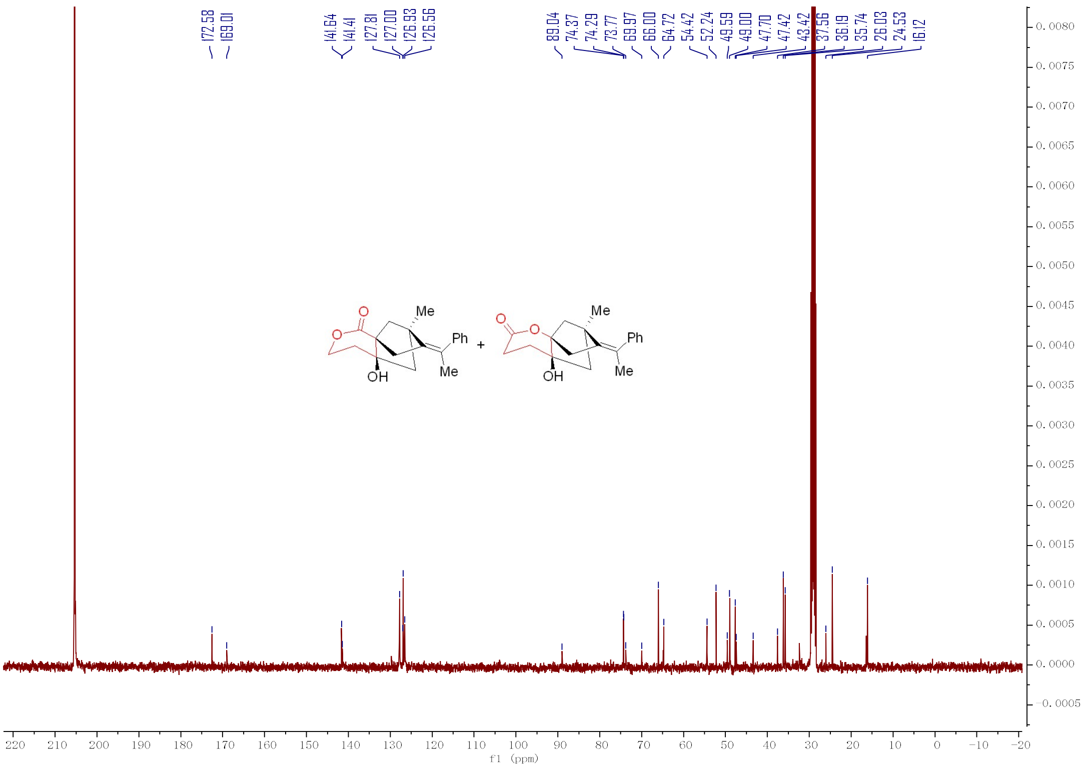

Supplementary Figure 188. ^{13}C NMR spectrum of product 9

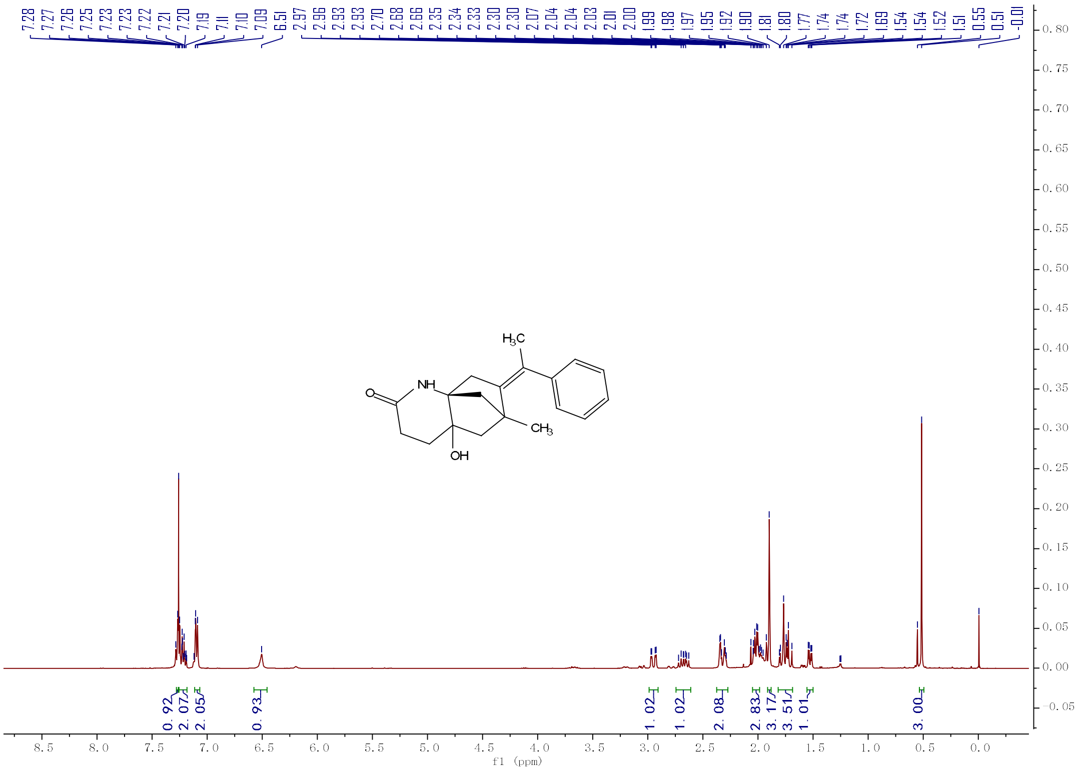

Supplementary Figure 189. ^{1}H NMR spectrum of product 10

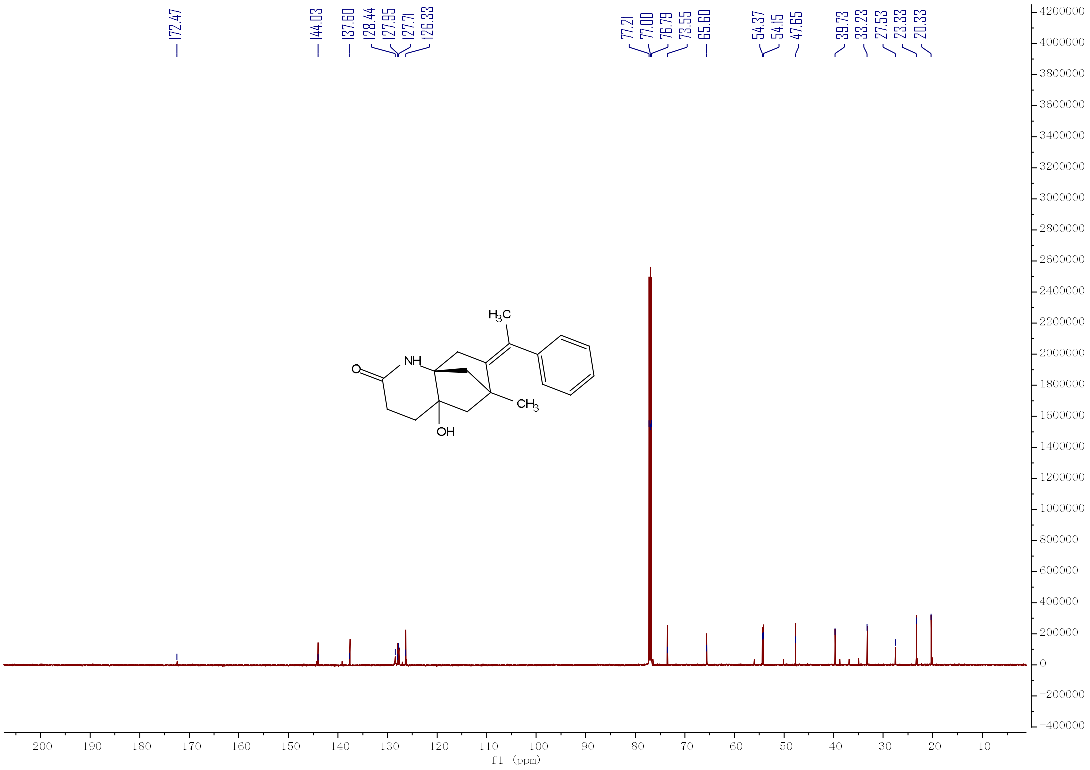

Supplementary Figure 190. ^{13}C NMR spectrum of product 10

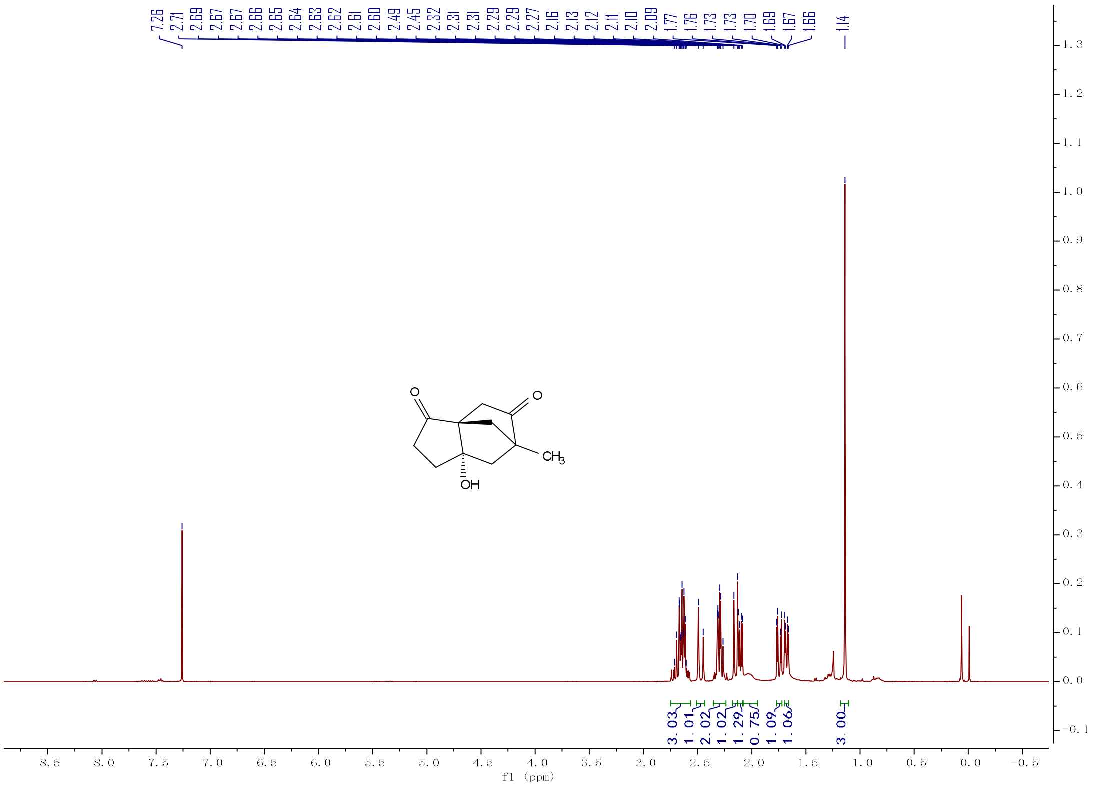

Supplementary Figure 191. ^{1}H NMR spectrum of product 11

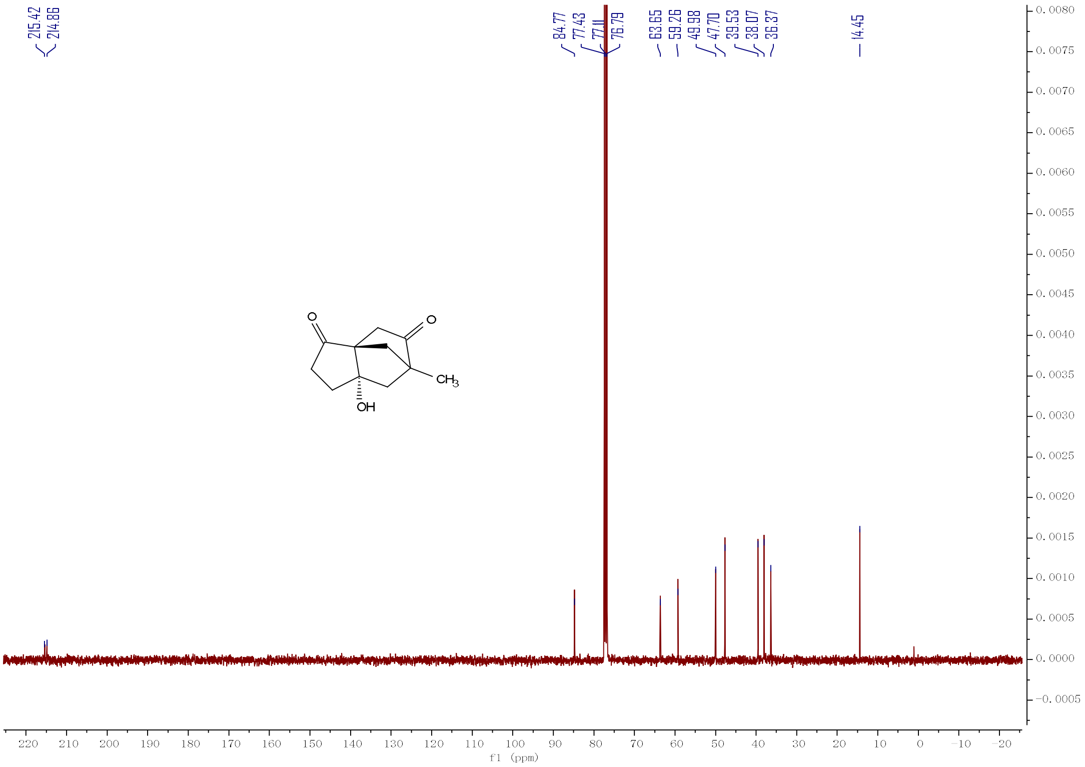

Supplementary Figure 192. ^{13}C NMR spectrum of product 11

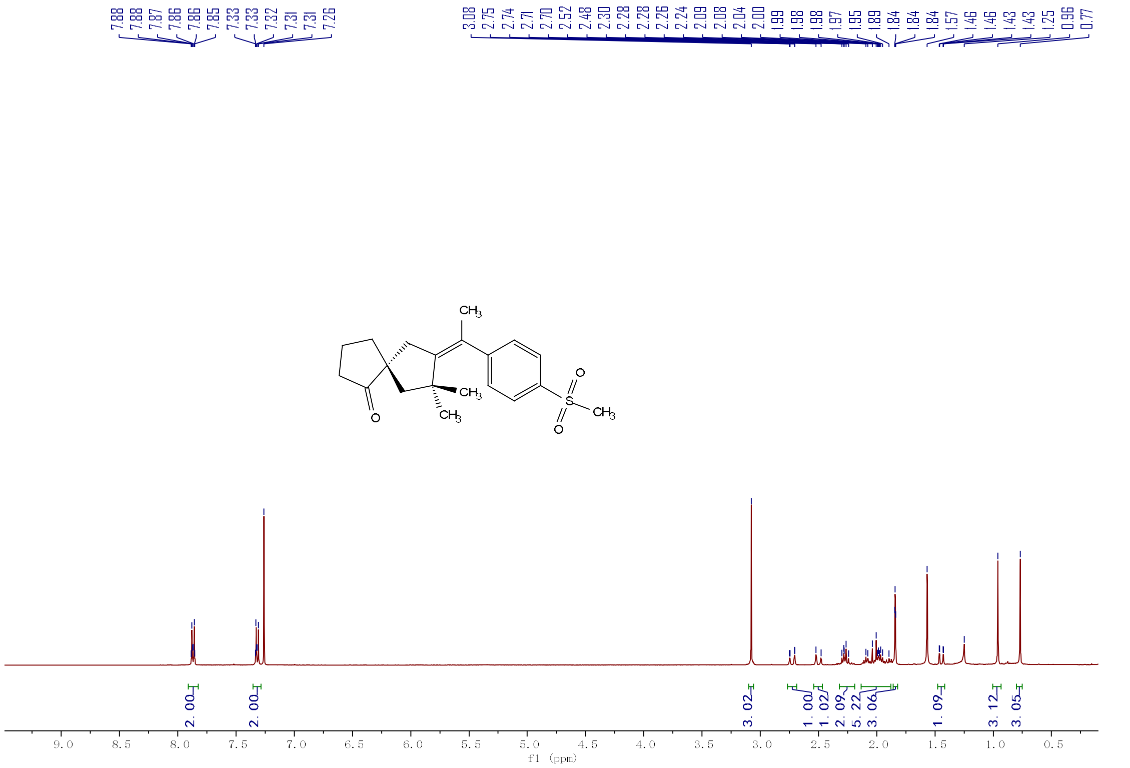

Supplementary Figure 193. ^{1}H NMR spectrum of product 14

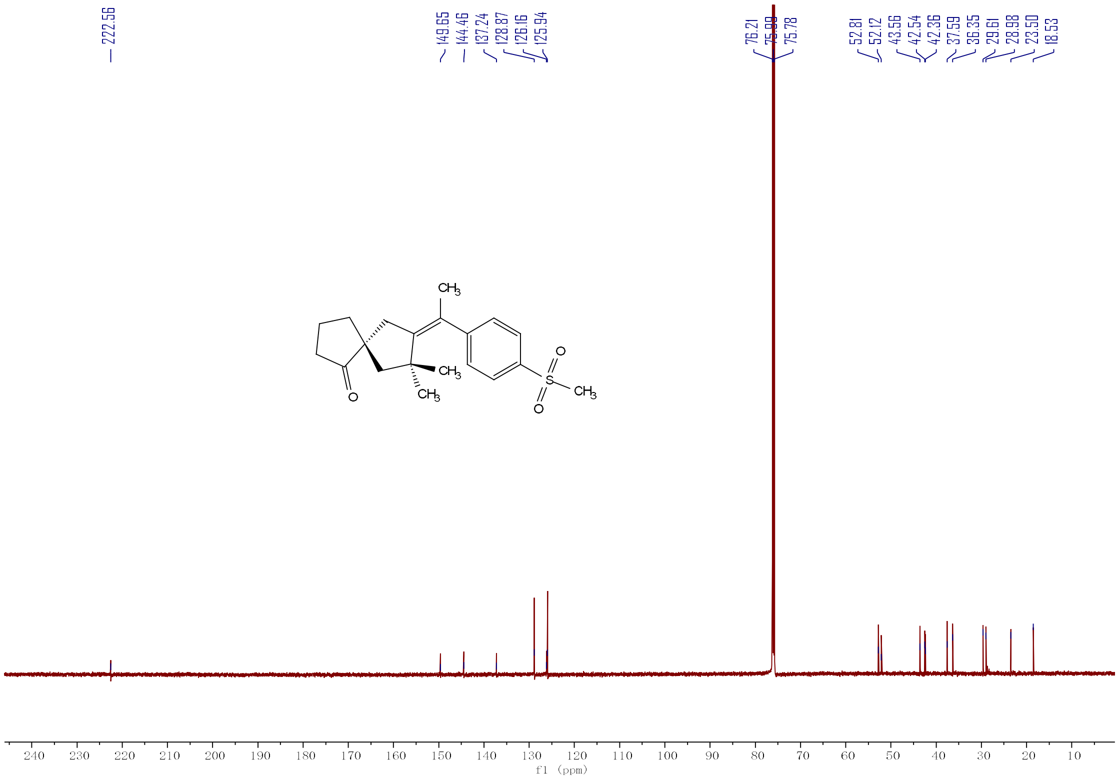

Supplementary Figure 194. ^{13}C NMR spectrum of product 14

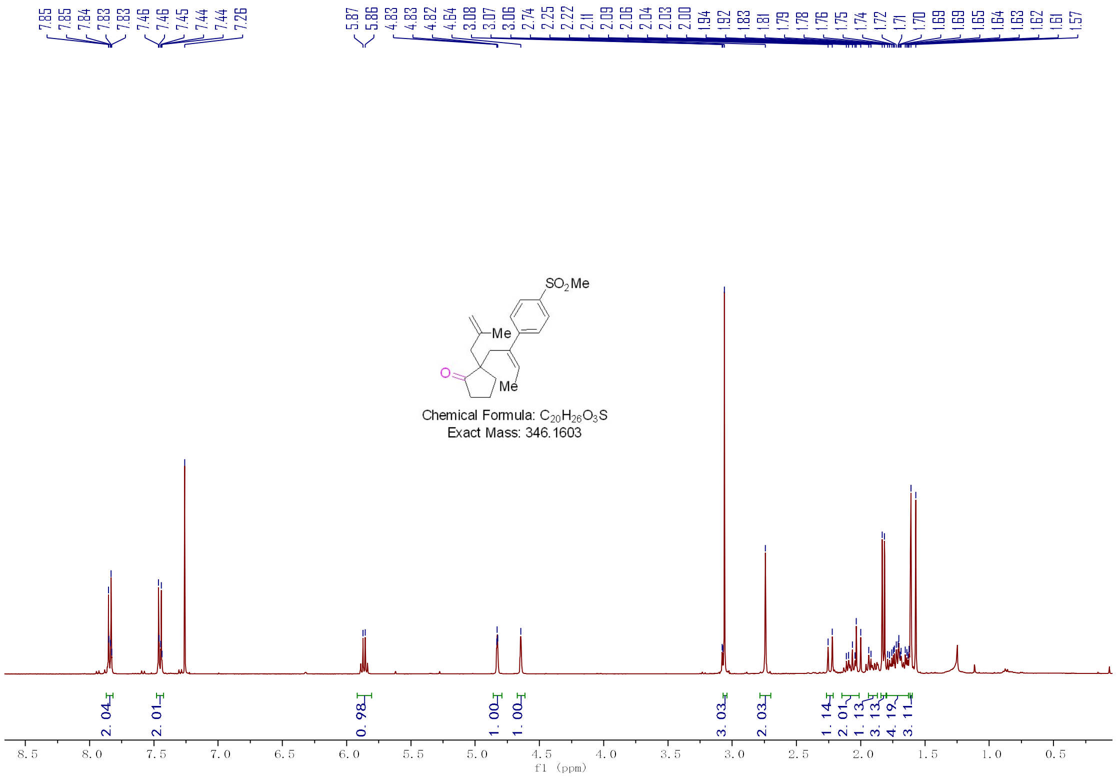

Supplementary Figure 195. ^{1}H NMR spectrum of product 15

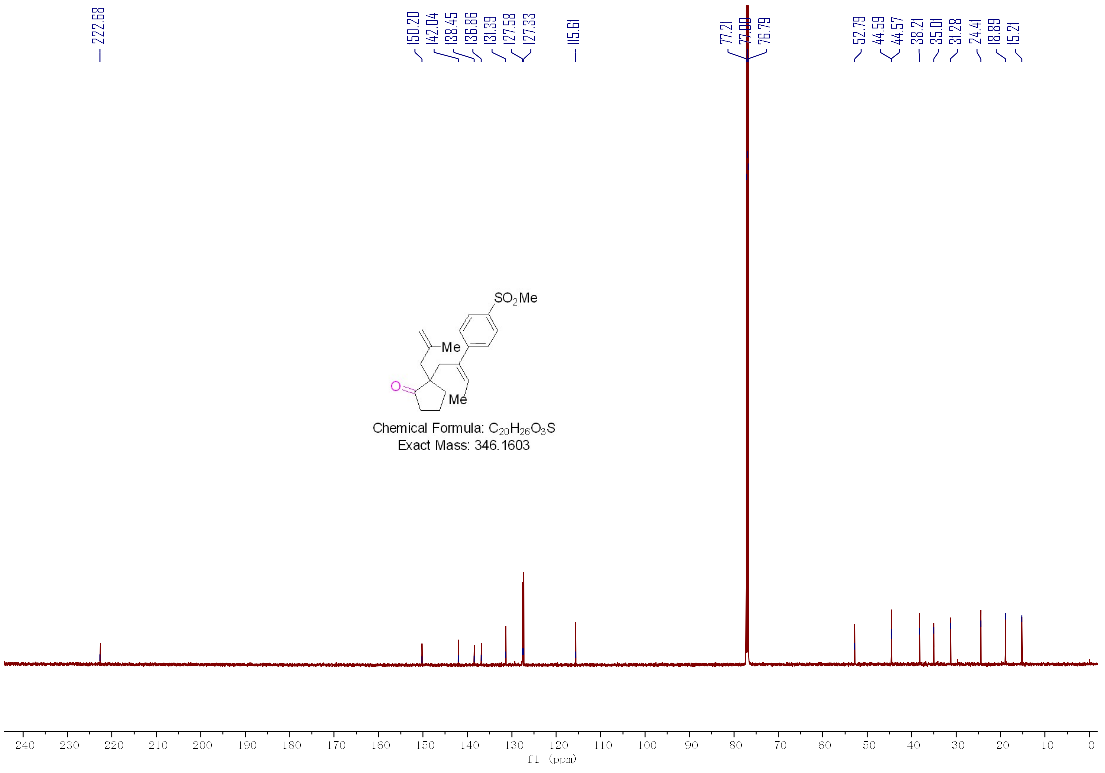

Supplementary Figure 196. ^{13}C NMR spectrum of product 15

Supplementary References

1. Ramachary, D. B., Kishor, M. Direct amino acid-catalyzed cascade biomimetic reductive alkylations: application to the asymmetric synthesis of Hajos-Parrish ketone analogues. *Org. Biomol. Chem*. **6**, 4176-4187 (2008);
2. Cuadros, S., Dell'Amico, L., Melchiorre, P. Forging Fluorine-Containing Quaternary Stereocenters by a Light-Driven Organocatalytic Aldol Desymmetrization Process. *Angew. Chem. Int. Ed*. **56**, 11875-11879 (2017).
